# A chemoproteomic biotechnological toolkit for resolving xylanase specificity in decorated xylan

**DOI:** 10.1038/s41467-026-74484-0

**Published:** 2026-06-22

**Authors:** Thamy L. R. Corrêa, Zirui Li, Olga Moroz, Isabelle B. Pickles, Andrey A. Lebedev, Saeed Akkad, Lianne I. Willems, Jeroen D. C. Codée, Herman S. Overkleeft, Gideon J. Davies

**Affiliations:** 1 York Structural Biology Laboratory, Department of Chemistry, University of York, York, North Yorkshire UK; 2 Department of Bio-organic Synthesis, Leiden Institute of Chemistry, Leiden University, Einsteinweg 55 Leiden, The Netherlands; 3 CCP4, STFC Rutherford Appleton Laboratory, Harwell Oxford, Didcot, UK; 4 York Biomedical Research Institute, Department of Chemistry, University of York, North Yorkshire, UK

**Keywords:** Enzymes, Enzymes

## Abstract

Xylanases are central to lignocellulosic biomass degradation, yet current methods lack the specificity to resolve how enzymes distinguish complex xylan structures decorated with arabinofuranose (Ara*f*) and 4-*O*-methyl-glucuronic acid (MeGlcA). Here, we report a suite of chemically-defined activity-based probes (ABPs) that enable the selective detection of arabinoxylan- and glucuronoxylan-specific xylanases (AXXs and GXXs). These cyclophellitol-derived ABPs covalently label retaining xylanases at their active sites, allowing precise mapping of substrate specificity across diverse glycoside hydrolase families. Crystallographic and mass spectrometric analyses reveal the molecular basis of probe selectivity, while in-gel and pull-down assays demonstrate their effectiveness in profiling xylanase activities in complex bacterial and fungal proteomes, including cellulosomes. By integrating activity-based protein profiling (ABPP) with sequence similarity networks (SSNs), we further show that xylanase specificity can be predicted from sequence alone, enabling rapid functional annotation of uncharacterized xylanases. This chemoproteomic strategy provides a powerful platform for discovering and engineering substrate-specific enzymes for biomass valorisation, microbial ecology, and biotechnological applications.

## Abstract (web summary)

Xylan is a complex plant polysaccharide with chemical decorations that shape its degradation. Here, the authors develop selective probes for xylanases targeting these structures in biological samples, offering a powerful approach for enzyme discovery and optimization for biotechnology.

## Introduction

Despite advances in genomics and structural prediction, the functional annotation of carbohydrate-active enzymes (CAZymes) remains a major bottleneck in enzyme discovery^1^. Many predicted proteins lack clear substrate assignments, particularly within glycoside hydrolase (GH) families, where subtle structural differences dictate specificity for decorated polysaccharides such as xylan. Sequence-based tools, including AlphaFold, are limited in their ability to predict enzymatic activity or substrate scope when decorations such as, in the case of xylan, arabinofuranose or glucuronic acid are present^2,3^. Activity-based protein profiling (ABPP) offers a powerful alternative: by covalently tagging active enzymes in their catalytic site, ABPP bypasses the need for prior annotation and enables functional screening, even in complex proteomes^4,5^. However, existing ABPs lack the substrate discrimination required to resolve activities tailored to one of nature’s most complex and challenging substrates - natural, decorated xylan structures.

The design and application of activity-based probes (ABPs) targeting glycan-processing enzymes - particularly retaining GHs - have attracted growing interest for their utility in biotechnology and human health^6^. Inspired by the mechanism-based inhibition of β-glucosidases by cyclophellitol^7^, we have developed cyclophellitol-derived ABPs, modified at the sugar ring to selectively target key enzymes for biomass degradation: retaining cellulases, xylanases, xyloglucanases, α-L-arabinofuranosidases, and amylases^8–14^. The use of ABPs to profile enzyme activities useful in the biotechnological arena holds great appeal for advancing our understanding on the complex consortia involved in, and dynamics of, polysaccharide degradation.

**Fig. 1:**
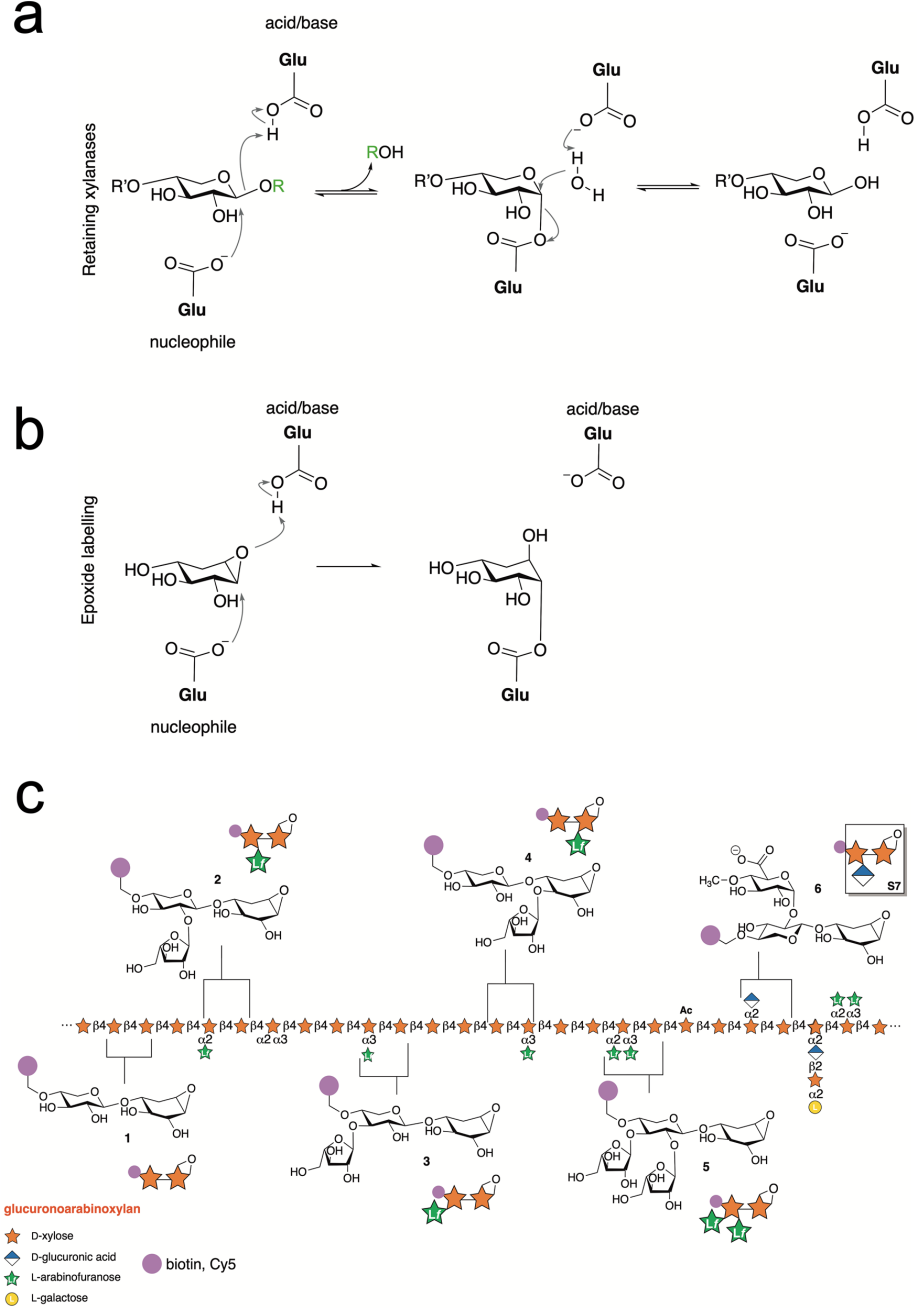
Mechanism of action of xylanases, overview of xylan structure and chemical probes used in this study. **a** Mechanism of action of xylanases involves nucleophilic attack by the glutamate residue, resulting in the formation of a covalent intermediate. **b** Covalent inhibition of retaining xylanases by cyclophellitol epoxides. Activation by the catalytic acid/base residue enables nucleophilic attack by the catalytic carboxylate, forming a non-productive covalent adduct that blocks water-mediated turnover and results in irreversible inhibition. **c** Schematic overview of xylan structure, highlighting key decorations: arabinofuranose (Ara*f*), 4-*O*-methyl-glucuronic acid (MeGlcA), and acetyl groups (Ac). Structure of cyclophellitol-derived activity-based probes and inhibitor (ABPs **1-6,** **S7**) designed to mimic native arabinoxylan or glucuronoxylan oligosaccharides. Probe design incorporates strategic decoration patterns to target specific xylanase activities. Reporter tags (Cy5 or biotin) are installed at the non-reducing end to preserve enzyme binding and enable visualization or enrichment. This probe suite enables selective covalent labelling of AXXs and GXXs at their -1 and -2 subsites, resolving xylanase specificity in complex biological samples. **S7** was used in crystallographic studies of GXXs. See Supplementary Information for full synthetic schemes (Supplementary Figs. 1–3) and chemical characterization. Symbols for sugars were obtained from https://grtc.ucsd.edu/education/textbook/slides.html).

Among these enzymes, xylanases play a central role in the degradation of xylan. Xylanases classified across the families GH5, 10, 11, and 30 in CAZy database cleave glycosidic bonds between xylosyl units via a retaining double-displacement mechanism. In this mechanism, the nucleophilic residue - targeted by the ABPP technology - forms a covalent glycosyl-enzyme intermediate^15^. Subsequently, the acid/base catalytic residue protonates the glycosidic oxygen, stabilizing the leaving group. In the second step, the same residue acts as a base to deprotonate a water molecule, which then attacks the intermediate, leading to cleavage of the glycosidic bond and release of the product^16^ (Fig. 1a).

Prior studies have demonstrated that mono- and disaccharide-based ABPs bearing electrophilic epoxide or aziridine warheads can effectively label β-xylanases (GH10) and β-xylosidases (GH3) by covalently reacting with their catalytic nucleophile - typically a glutamate residue - forming a stable adduct that resembles the natural glycosyl-enzyme intermediate^10,17^ (Fig. 1b). Cyclophellitol epoxides and aziridines are weak electrophiles that are protonated by the catalytic acid/base residue. This protonation stabilises the developing positive charge and facilitates nucleophilic attack by the catalytic carboxylate. The resulting covalent adduct is not a productive glycosyl-enzyme intermediate and blocks water-mediated turnover, rendering these probes highly selective, mechanism-based, irreversible inhibitors^18^.

These early ABPs were instrumental in probing xylan-degrading activities in fungal systems and assessing enzyme performance. However, these probes target enzymes acting primarily on non-decorated xylan backbones and do not distinguish between enzymes specific for decorated substrates such as arabinoxylan or glucuronoxylan^10,19^. Thus, they lack the structural resolution required to functionally annotate arabinoxylan- and glucuronoxylan-specific xylanases in biological or biotechnological enzyme mixtures.

Xylan is a linear polymer made of xylobiose units. The structural complexity of xylan arises from its decoration with α-1,2/α-1,3 (O)-linked acetyl (Ac), α-1,2/α-1,3 (O)-linked arabinofuranosyl (Ara*f*), and α-1,2(O)-linked 4-*O*-methyl glucuronic acid (MeGlcA) (Fig. 1c).

The distribution and abundance of these decorations vary across plant species, tissues, and plant developmental stages^20^. For example, in grasses, xylan can range from evenly substituted arabinoxylan to highly substituted glucuronoarabinoxylan (GAX). Although the precise biological roles of these decorations remain unclear, they are thought to mediate interactions with other cell wall components, influencing cell wall architecture and biomass recalcitrance^21^.

β-xylanases and β-xylosidases act in concert to deconstruct xylan into its xylose units, with the xylooligosaccharides (XOs) produced by the former enzyme serving as substrates for the latter^22^. β-xylanase activity is often challenged by the structural complexity of xylan. The hindrance caused by the decorations on β-xylanases activity can be alleviated by the action of accessory enzymes, such as acetyl xylan esterases, α-L-arabinofuranosidases, and α-glucuronidases, which specifically remove these decorations through distinct mechanisms^22^. Crucially, efficient degradation of xylan (especially relevant for industry) also relies on the activity of specialised xylanases, such as arabinoxylan-specific xylanases (AXXs) and glucuronoxylan-specific xylanases (GXXs), which make productive interactions with Ara*f* and MeGlcA groups, respectively, releasing decorated XOs from these complex challenging substrates^23–26^.

AXXs and GXXs are retaining GHs classified within the CAZy^27^ GH5_34/GH10 and GH30 (GH30_7 and GH30_8)^28,29^ families/subfamilies, respectively. Like “traditional” xylanases, AXXs and GXXs operate via the double displacement mechanism, in which a glutamate residue within the catalytic site serves as nucleophile. Their substrate specificity arises from molecular determinants that govern interactions with xylan decorations. For instance, GH5 AXXs are strictly dependent on the presence of Ara*f*, whereas GH10 AXXs can accommodate and cleave non-decorated xylan and regions decorated with groups other than Ara*f*^30,31^. Turning to GXXs, GH30_7 and GH30_8 share low sequence identity (around 20%), with GH30_7 consisting exclusively of fungal members, while GH30_8 harbours the bacterial strains^32^. Notably, GH30_8 exhibits significantly higher activity on MeGlcA-decorated xylan, in contrast to GH30_7^33^. Therefore, carefully balancing the properties of each enzyme is essential for producing XOs from specific substrates.

While existing ABPs target xylanases acting on linear xylan backbones, natural xylans are far more complex, decorated with chemical groups that has driven the co-evolution and diversification of enzymes capable of recognizing, modifying, or degrading these decorated polymers. Here, we develop a suite of chemically tailored, cyclophellitol-derived ABPs that selectively and covalently label endo-acting retaining AXX and GXX specific xylanases. Through structural, biochemical, and proteomic analyses, we reveal the molecular determinants of probe selectivity and demonstrate their utility in complex microbial systems, including both free enzymes and cellulosomes. Furthermore, by integrating ABPP with sequence similarity network (SSN) analysis, we establish a predictive platform for enzyme functional annotation directly from sequence data. This chemoproteomic strategy offers a powerful approach for discovering, profiling, and engineering substrate-specific xylanases for biotechnological applications.

## Results and discussion

### Design and synthesis of specificity-resolving xylanase probes

To dissect substrate-specific xylanase activity across decorated xylan structures, we designed a suite of ABPs that mimic the key molecular features of arabinoxylan and glucuronoxylan. These probes were engineered to covalently bind retaining xylanases that require Ara*f* or MeGlcA decorations for substrate recognition. This selective covalent labelling strategy enablesthe chemoproteomic distinction of AXXs and GXXs within complex biological mixtures.

Previous probes lacked the ability to discriminate xylanase activity based on xylan decorations^10^. To address this, we designed six ABPs by modifying a xylobiose-configured cyclophellitol epoxide scaffold with strategically positioned Ara*f* or MeGlcA decorations. Ara*f* decorations were introduced at (O2), (O3), or both positions on the terminal xylose, reflecting their natural heterogeneity in arabinoxylan, whereas MeGlcA was placed at (O2), consistent with its occurrence in glucuronoxylan. By incorporating detection handles at the non-reducing end - mimicking the natural glycan extension direction - we further optimized the probes for compatibility with both fluorescent (Cy5) and affinity-based (biotin) detection. The resulting toolkit comprises ABPs **1-6** (Fig. 1c), each with defined decoration patterns, offering a comprehensive platform to resolve xylanase specificity at the molecular level.

**Fig. 2:**
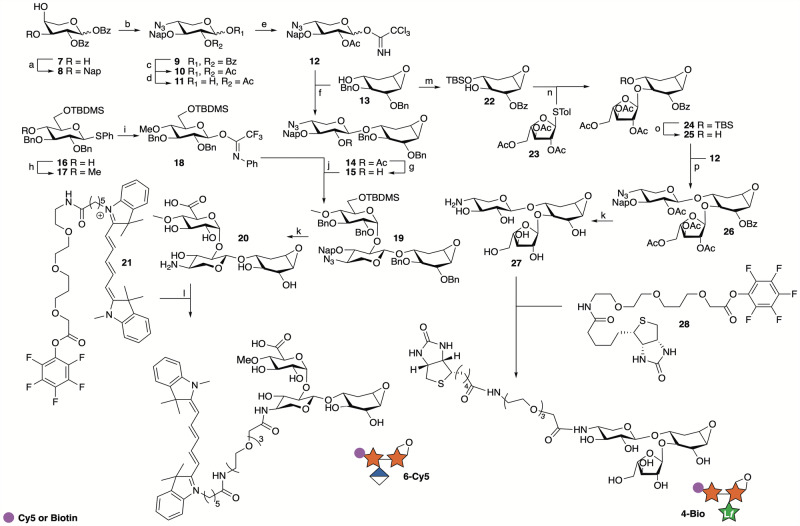
Chemical probe synthesis exemplified for ABPs 4-Bio and 6-Cy5 (see Supplementary Information for full experimental and analytical details of all chemical probe syntheses). Reagents and conditions: a) Bu_2_SnO, toluene, 90 °C, then NapBr, CsF, DMF (56%); b) (i) Tf_2_O, pyridine, CH_2_Cl_2_, (ii) NaN_3_, DMF (90%); c) (i) NaOMe, MeOH, CH_2_Cl_2_, (ii) Ac_2_O, pyridine (88%); d) piperidine, THF (68%); e) Cl_3_CCN, K_2_CO_3_, CH_2_Cl_2_ (90%); f) TMSOTf, CH_2_Cl_2_, -30 °C (79%); g) NaOMe, MeOH, CH_2_Cl_2_ (90%); h) MeI, NaH, DMF (99%); i) (i) NIS, acetone/H_2_O, (ii) N-phenyltrifluoroacetimidoyl chloride, Cs_2_CO_3_, acetone (78%); j) TfOH, CH_2_Cl_2_, -78 °C to 0 °C (67%); k) (i) TBAF, THF, Δ (90%), (ii) TEMPO, BAIB, CH_2_Cl_2_/tBuOH/H_2_O 4/4/1 (79%), (iii) H_2_, Pd(OH_2_)/C, TFA, dioxane/H_2_O 4/1 (78%); l) (1) **21**, pentafluorophenyl trifluoroacetate, DiPEA, DMF, (ii) add 20, (iii) reversed phase HPLC purification (23%); m) (i) TBSOTf, 2,6-lutidine, CH_2_Cl_2_, -78 °C (95%), (ii) H_2_, Pd(OH)_2_/C, MeOH/H_2_O/dioxane 1/1/1 (93%), (iii) BzCl (1 equiv.), pyridine, 0 °C to rt (81%); n) N-iodosuccinimide, TMSOTf, CH_2_Cl_2_, -40 °C (70%); o) TBAF, AcOH, THF (79%); p) TMSOTf, CH_2_Cl_2_, -30 °C (86%); q) (i) NaOMe, MeOH/CH_2_Cl_2_ 1/1, (ii) H_2_, Pd(OH)_2_/C, TFA, dioxane/H_2_O 3/1 (81%); r) (i) 28, pentafluorophenyl trifluoroacetate, DiPEA, DMF, (ii) add 27, (iii) reversed phase HPLC purification (30%).

A key design improvement addressed a limitation in earlier ABPs: susceptibility to exoglycosidase degradation due to exposed non-reducing ends^10^. To overcome this, we installed the reporter group (Cy5 or biotin) at the non-reducing end (O4) of the terminal xylose, mimicking the native oligosaccharide extension and ensuring compatibility with endo-xylanase binding. This configuration preserved probe integrity and enabled dual utility - fluorescence-based detection and affinity-based enrichment - in complex proteomes. Figure 2 illustrates the representative synthetic pathways for ABPs **4-Bio** and **6-Cy5**, which exemplify the core strategies applied across the full probe suite.

The synthesis of **6-Cy5** commences with partially protected D-arabinopyranoside **7**^34^, which was selectively naphthylated at O3 to allow S_N_2 substitution of the remaining alcohol at C4 for an azide (**7** to **9**). Saponification of the two benzoates in **9** followed by acetylation of O1 and O2 in the resulting compound **10** and then selective removal of the anomeric acetate (**10** to **11**) allowed for transformation of hemi-acetal **11** into donor tricholoacetimidate **12**. Condensation of 12 with beta-D-*xylo*-cyclophellitol **13** under the agency of TMS triflate provided orthogonally protected xylobiose-configured cyclophellitol **14** with O’2 and O’3 available for further modification. In the depicted example, the acetate at O’2 is removed and the thus liberated alcohol in **15** condensed with orthogonally protected 4-OMe-glucopyranoside 18, activated as the N-phenyl-trifluoroacetimidate donor, and which for this purpose was prepared in three steps (methylation of the free OH-4, then removal of the anomeric thio-ether and finally introduction of the imidate) from the known partially protected phenylthioglucopyranoside **16**^35^. The silyl protective group in the obtained trisaccharidic cyclophellitol **19** was then selectively removed and the resulting free alcohol oxidized to the carboxylic acid using catalytic 2,2,6,6-Tetramethylpiperidine 1-oxyl (TEMPO) free radical and bisacetoxy-iodobenzene (BAIB) as co-oxidant. Catalytic hydrogenolysis of the benzyl- and naphthyl ethers then provided compound **20** which we condensed with triethylene glycol spacered Cy5 **21** by first in situ activating the carboxylic acid in **21** to the pentafluorophenyl ester and then adding carboxylic acid **20** in an adaptation of the strategy we previously used for the synthesis of cellobiose-configured ABPs^8^. Reversed phase HPLC purification then yielded **6-Cy5** in milligram quantities in analytically pure form.

For the synthesis of **4-Bio**, partially protected beta-D-*xylo*-cyclophellitol **13** was transformed into partially and orthogonally protected beta-D-*xylo*-cyclophellitol **22** by first silylation of the free OH in **13**, then catalytic hydrogenolysis of the two benzyl ethers and finally selective benzoylation of OH-2. Compound **22** was then glycosylated with 1-*S*-tolyl-2,3,5-tri-*O*-acetyl-alpha-L-arabinofuranoside **23**^36,37^ to give disaccharidic cyclophellitol **24**. Removal of the silyl protective group and then condensation of the resultant **25** with acceptor *xylo*-cyclophellitol **12** afforded fully and orthogonally protected trisaccharidic cyclophellitol 26. Saponification of the acetate and benzoate esters in **26** and then hydrogenolysis of the naphthyl ether and azide gave amine **27** which was then condensed with biotin, in situ transformed into the pentafluorophenyl ester, to give after RP-HPLC purification, ABP milligram quantities of **4-Bio** in analytically pure form.

### Functional profiling of AXX and GXX xylanases via chemoselective probes

Having developed substrate-mimicking ABPs tailored for arabinoxylan and glucuronoxylan, we next tested their ability to resolve xylanase specificity in a family- (and sub-family) and substrate-dependent manner. We selected recombinant *Ct*Xyl5A (GH5_34) and *Ct*Xyn30A (GH30_8), two well-characterized enzymes from *Clostridium thermocellum* with strict specificity for Ara*f*- and MeGlcA-decorated xylan, respectively. These models enabled direct assessment of whether individual ABPs can selectively label AXXs or GXXs based on their substrate-recognition profiles - an unresolved challenge in functional enzyme annotation^24,26,38^.

**Fig. 3:**
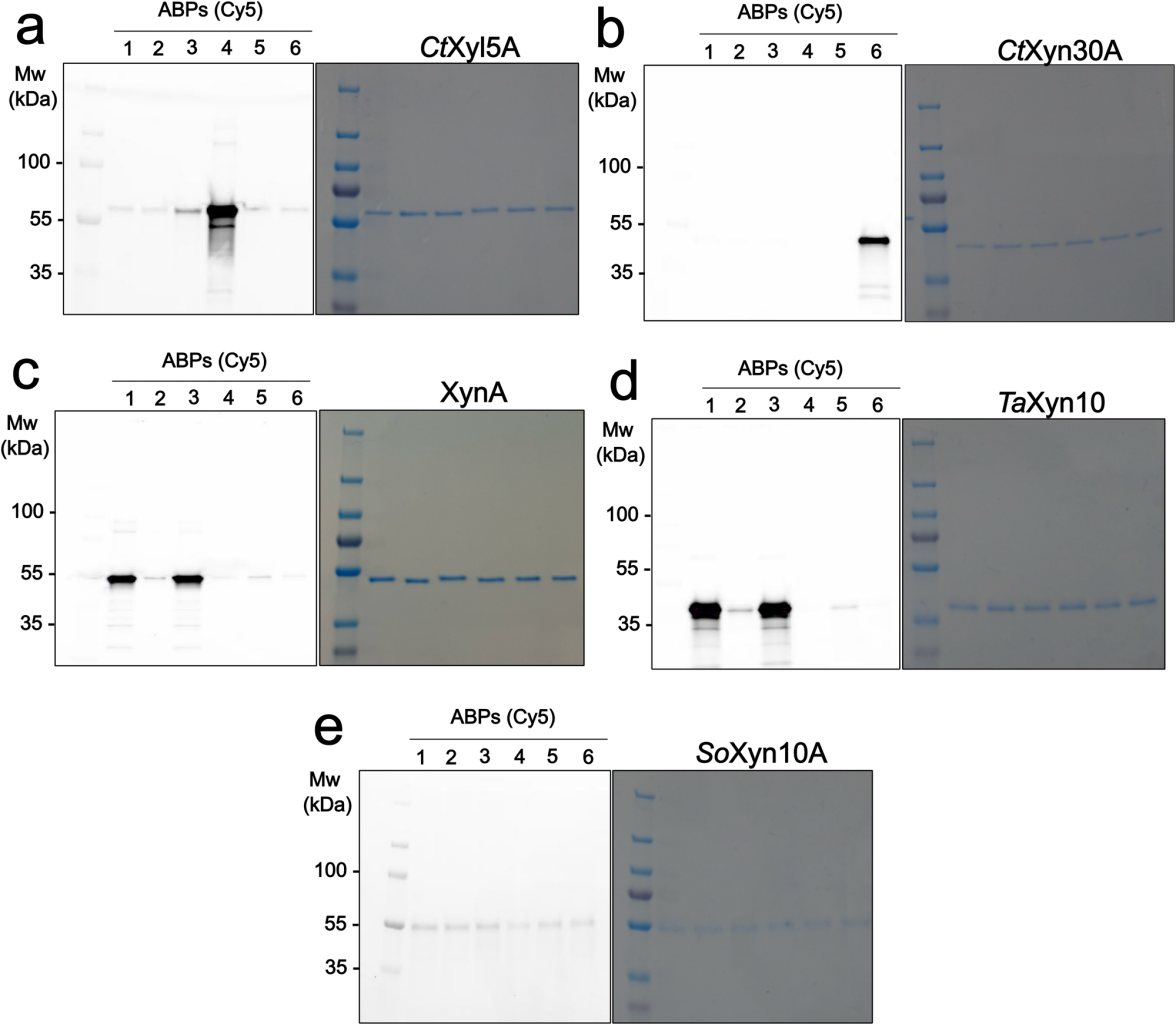
Selective labelling of xylanases with substrate-decorated ABPs. **a** *Ct*Xyl5A (GH5_34), a strict arabinoxylan-specific xylanase (AXX), is selectively labelled by ABP **4-Cy5**, which presents an α−1,3-arabinofuranose (Ara*f*) decoration. **b** *Ct*Xyn30A (GH30_8), a glucuronoxylan-specific xylanase (GXX), reacts exclusively with ABP **6-Cy5**, bearing a 4-*O*-methyl-D-glucuronic acid (MeGlcA) group. **c**, **d** GH10 AXXs - XynA and *Ta*Xyn10- are labelled by both ABP **1-Cy5** (non-decorated xylobiose) and ABP 3-Cy5 (α−1,3-Ara*f*), reflecting broader substrate promiscuity. **e** *So*Xyn10A, a known broad-specificity xylanase, reacts with multiple ABPs including both Ara*f* - and MeGlcA-decorated variants. The identification of ABPs **1-6** follows the same numbering as in Fig. 1c. These results demonstrate that ABPs **1–6-Cy5** can resolve xylanase specificity patterns across GH families, enabling functional profiling of strict and promiscuous activities toward decorated xylans. The predicted molecular masses (kDa) for each protein are: *Ct*Xyl5A, 54.1; *Ct*Xyn30A, 46.8; XynA, 49.9; *Ta*Xyn10, 35.5; *So*Xyn10A, 48.1 (Supplementary Table 1). The Coomassie blue-stained gels are shown on the right. The raw scans and gels data are provided in Source Data. The in-gel assays were performed in triplicate. Mw, molecular weight.

We tested each ABP (**1–6-Cy5**) for its ability to selectively label *Ct*Xyl5A and *Ct*Xyn30A using fluorescence gel-based assays. Clear and distinct labelling patterns emerged: *Ct*Xyl5A reacted exclusively with ABP **4-Cy5**, while *Ct*Xyn30A showed selective labelling by ABP **6-Cy5** (Fig. 3a, b). These patterns directly reflect the substrate specificity encoded by each enzyme - *Ct*Xyl5A recognizes Ara*f*-decorated xylan, while *Ct*Xyn30A is selective for MeGlcA. This study demonstrates that this set of ABPs can resolve xylanase specificity based on substrate decorations, validating their potential for substrate-guided enzyme profiling.

To explore the broader mechanistic diversity of AXXs, we next applied our ABP panel to enzymes from the GH10 family, which exhibit more flexible recognition of Ara*f*-decorated xylans than their GH5 counterparts. Unlike MeGlcA, which occurs at a single defined position of the sugar ring (O2), Ara*f* can be linked at multiple positions within the xylobiosyl unit, reflected in our ABP designs (Fig. 1c). We selected two well-characterized GH10 enzymes - XynA from *Bacillus* sp. KW1 and *Ta*Xyn10 from *Thermoascus aurantiacus* - both of which accommodate Ara*f*-decorated xylans^25,39,40^. Gel-based labelling assays showed that these enzymes reacted with both ABP **1-Cy5** (non-decorated xylobiose) and ABP **3-Cy5** (α−1,3-Ara*f* xylobiose), reflecting both their substrate promiscuity and the ability of our ABPs to capture these functional differences (Fig. 3c, d). These results demonstrate that the ABP suite can distinguish subtle differences in decoration-dependent substrate preferences within and across xylanase families.

To validate further the discriminatory power of our ABP panel, we included *So*Xyn10A, a GH10 xylanase from *Streptomyces olivaceoviridis* E-86 with broad substrate specificity toward both Ara*f*- and MeGlcA-decorated xylans^31^. Gel-based assays revealed that *So*Xyn10A indeed reacted with multiple probes, including both decorated and non-decorated variants, consistent with its known promiscuity. This response confirms that the ABP suite can faithfully report both strict and flexible substrate recognition patterns, validating its use not only for target discrimination but also for identifying broad-spectrum xylanases in mixed systems (Fig. 3e).

### Mechanistic insights into ABP selectivity for Ara*f* decorated xylanases

**Fig. 4:**
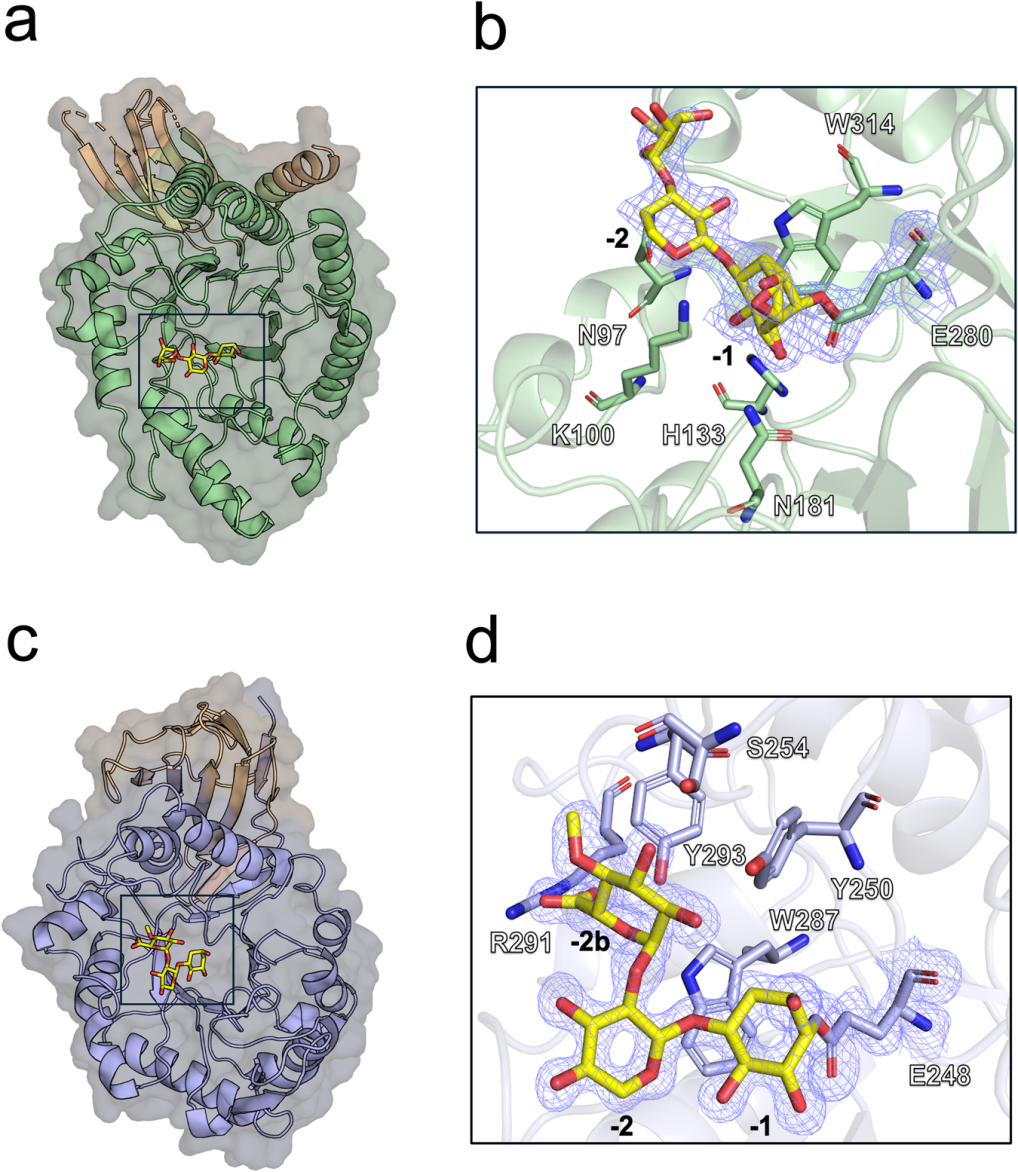
Structural characterization and covalent labelling of decorated xylanase-ABP complexes. **a** X-ray structure of XynA in complex with ABP **3-Bio** (2.0 Å). **b** Active-site view showing the cyclophellitol epoxide positioned at the catalytic nucleophile Glu280. The density is consistent with partial occupancy of a covalently linked glycosyl-enzyme intermediate (the covalent conformation is shown). Electron density for the α−1,3-Ara*f* group was observed. The electron density (2Fo-Fc) is shown for the ligand as blue mesh contoured at 1.0σ. Hydrogen bonding and hydrophobic interactions are observed between the xylobiosyl-Ara*f* moiety and residues Trp314, Asn97, and Lys100. Structural alignment with related GH10 enzymes (*Cm*Xyn10B, *So*Xyn10A) confirms conserved Ara*f* recognition, while a hydrogen bond with Asn97 appears unique to XynA. **c** X-ray structure of *Ct*Xyn30A bound to **S7** (1.5 Å). **d** MeGlcA is positioned in the −2b subsite, forming a salt bridge with Arg291 and hydrogen bonds with Tyr293, Tyr250, Ser254, and Trp287 to stabilize the complex. The electron density (2Fo-Fc) is shown for **S7** as blue mesh contoured at 1.5σ. We note that in 3D analyses, of all enzymes in this study, we do only observe labelling of the catalytic nucleophile and no off-target reactivity towards other amino acids.

To investigate the molecular basis of ABP selectivity toward decorated xylans, we pursued soaking and co-crystallization of xylanase-probe complexes. Despite repeated attempts, we were unable to obtain high-resolution crystals of the *Ct*Xyl5A-**4-Cy5** complex. However, soaking of the GH10 enzyme XynA with ABP **3-Bio** - a xylobiosyl-configured cyclophellitol probe bearing an α-1,3-Ara*f* decoration - was successful. Initial soaking experiments with the Cy5-labelled probe yielded no interpretable electron density, while co-crystallization attempts disrupted crystal formation altogether. To overcome these limitations, we employed the biotinylated version of the probe (**3-Bio**), reasoning that the smaller biotin moiety would reduce steric hindrance and, if fortunate, minimize disruption to protein-ligand interactions. This strategy proved successful and enabled structural characterization of the XynA-**3-Bio** complex at 2.0 Å resolution (Fig. 4a, Supplementary Table 2).

Initial refinement revealed electron density in the active site consistent with probe binding at the catalytic nucleophile (Glu280). However, detailed inspection of omit and weighted difference omit maps indicated that the density at the anomeric carbon is not fully consistent with a single, fully occupied covalent adduct. Instead, the maps are best explained by partial occupancy of two states: an intact epoxide and a covalently linked glycosyl–enzyme intermediate. Accordingly, both the ligand and Glu280 were modelled in alternative conformations with refined occupancies (Supplementary Fig. 4, Fig. 4b). These observations remain consistent with mechanism-based inhibition while indicating that the trapped intermediate represents a mixed population in the crystal. Importantly, both the xylobiosyl core and the Ara*f* were clearly resolved, validating that the ABP accurately mimics the natural substrate and engages key substrate-recognition elements within the binding cleft.

Structural alignment of XynA with *Cm*Xyn10B bound to Ara*f*-X2 (PDB: 1UR1) and *So*Xyn10A bound to Ara*f*-X3 (PDB: 1V6V) confirmed that these bacterial GH10 xylanases accommodate α-1,3-Ara*f* in a conserved fashion. However, XynA exhibits distinct features: a hydrogen bond between O3 of Ara*f* and Asn97 (2.8 Å), and a hydrophobic contact between Ara*f* and Glu96 (Supplementary Table 3). This interaction is analogous but not identical to those observed in *Cm*Xyn10B (Glu67–Ara*f*) and *So*Xyn10A (Glu44–Ara*f*), highlighting subtle variation in Ara*f* accommodation across GH10 enzymes (Supplementary Fig. 5). These findings confirm that ABP **3-Bio** mimics arabinoxylan binding with high fidelity while also revealing the enzyme-specific interactions that shape substrate selectivity.

While crystallographic analysis provided high-resolution insight into probe binding within a GH10 active site, we sought to further validate and generalize these interactions using mass spectrometry, a technique which also benefits from not needing purified protein. Covalent labelling by ABPs results in a defined mass shift and leaves a stable adduct at the catalytic nucleophile, enabling precise identification of probe-enzyme conjugates. We therefore performed LC-MS/MS analysis of labelled XynA, *Ta*Xyn10 and *Ct*Xyl5A to confirm covalent modification and map key protein–ligand contacts. This approach provided orthogonal confirmation of probe selectivity and extended our ability to analyse systems where crystallographic data, and ultimately purified over-expressed enzyme, are unavailable.

Mass spectrometry analysis revealed covalent labelling of XynA (49,710 Da), *Ta*Xyn10 (36,310 Da), and *Ct*Xyl5A (53,970 Da) upon reaction with ABP **3-Bio** and **4-Bio**, yielding labelled products with masses of 50,540 Da (XynA-**3-Bio**), 37,140 Da (*Ta*Xyn10-**3-Bio**), and 54,800 (*Ct*Xyl5A-**4-Bio**) - consistent with the predicted mass increase due to covalent probe attachment (Supplementary Fig. 6). In contrast, less than 10% conversion to the corresponding 1-Bio labelled species was observed, indicating that these enzymes preferentially accommodate decorated backbones, in agreement with previous reports^24,25^.

The observed selectivity corresponds with prior crystallographic data (PDB: 5LA2), which show that *Ct*Xyl5A contains a well-defined −2* subsite adjacent to the catalytic centre. This pocket forms productive interactions with the α-1,3-Ara*f* moiety linked to the xylose unit at the -1 subsite, identifying Ara*f* as a critical determinant of substrate recognition^23^. These results provide direct biochemical evidence that ABP **4** structurally mimics the native substrate required for *Ct*Xyl5A activity and selectivity.

### Structural and biochemical insights into glucuronoxylan-specific xylanase recognition

To characterize the structural basis for MeGlcA recognition, we determined the crystal structure of *Ct*Xyn30A in complex with the mechanism-based inhibitor **S7** at 1.5 Å resolution (Fig. 4c, Supplementary Table 2). Notably, this structure captures *Ct*Xyn30A bound to a uronic acid ligand in the -2b subsite. Previous structures showed only phosphate (PDB: 5A6M) or sulfate ions (PDB: 4UQC) at this position^26^, leaving the true mode of MeGlcA recognition unresolved. The inhibitor formed a covalent bond with the catalytic nucleophile, Glu248, and clear electron density was observed for the cyclophellitol core, the xylobiosyl backbone, and the 4-*O*-methyl-glucuronic acid (MeGlcA) group. The MeGlcA moiety was accommodated in the -2b subsite adjacent to the catalytic centre, forming a salt bridge with Arg291 and hydrogen bonds with Tyr293, Ser254, Tyr250, and Trp287 (Fig. 4d, Supplementary Table 4). These interactions explain the strict substrate selectivity of *Ct*Xyn30A for MeGlcA-substituted xylan, consistent with previous biochemical studies^26^ and structural predictions of GH30 enzymes^41^.

As observed for AXX, covalent binding of *Ct*Xyn30A to **S7** was confirmed by mass spectrometry, with complete conversion of *Ct*Xyn30A (46,860 Da) to *Ct*Xyn30A-S7 (47,330 Da) (Supplementary Fig. 6).

Having validated ABP specificity through crystallography and mass spectrometry, we next asked whether the probes could distinguish functional xylanase activities in far more complex biological systems.

### Resolving substrate-specific xylanase activities in cellulosomal proteomes

*C. thermocellum* was selected as a model organism due to its expression of *Ct*Xyl5A and *Ct*Xyn30A - two xylanases previously validated with our ABPs. These enzymes are localized in the cellulosome, a multi-enzyme complex central to biomass degradation^42,43^.

**Fig. 5:**
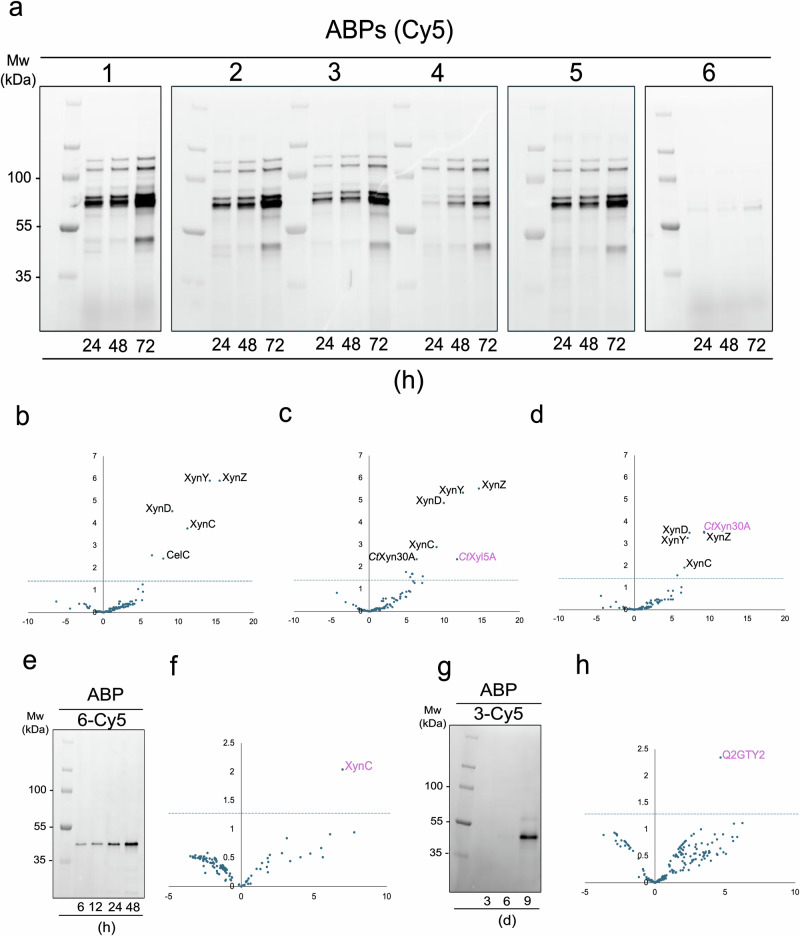
Activity-based profiling of AXX and GXX xylanases across bacterial and fungal proteomes. **a** In-gel fluorescence analysis of *C. thermocellum* secretomes (24, 48, and 72 h) incubated with ABPs **1–6-Cy5** reveals probe- and time-dependent labelling of xylanases.** b**–**d** Pull-down enrichment of ABP-labelled proteins from *C. thermocellum* secretomes using biotinylated probes **1-Bio** (non-decorated), **4-Bio** (Ara*f*), and **6-Bio** (MeGlcA), showing selective capture of xylanases including *Ct*Xyl5A and *Ct*Xyn30A. **e** In-gel fluorescence analysis of *B. subtilis* secretomes (6–48 h) following incubation with ABP **6** shows strong labelling of a ~ 47 kDa protein, consistent with GH30 xylanase XynC. **f** Pull-down enrichment from *B. subtilis* secretomes confirms selective capture of XynC by **6-Bio**. **g** In-gel labelling of *C. globosum* secretomes (3, 6, 9 days) with ABP **3-Cy5** highlights Ara*f*-responsive xylanase activity, with maximal signal at day 9. **h** Pull-down enrichment of ABP **3-Bio**-labelled proteins from *C. globosum* identifies GH10 enzyme Q2GTY2 as the top AXX candidate. Secretomes were incubated with probes **1–6-Cy5** (30 °C, 1 h) or with biotinylated analogues **1–6-Bio** (30 °C, 2 h), followed by SDS-PAGE or streptavidin-based enrichment and LC-MS/MS. In-gel assays were performed in triplicate. Pull-down assays were performed with two independent biological replicates. Proteins enriched in DMSO- and ABP-treated conditions (log_2_ fold change, *x* axis) were analysed by quantitative proteomics, and enrichment significance was defined using a Benjamini-Hochberg *p*-value ≤ 0.05 (corresponding to -log_10_ > 1.3 (*y*-axis). Raw gel images and scans are provided in Source Data. Full protein annotations are provided in Source Data 2. Mw, molecular weight.

To evaluate ABP performance under native expression conditions, *C. thermocellum* was cultured for 72 h in MTC medium supplemented with cellobiose as the carbon source^44,45^. The secretome was used for analysis, as cellulosomes are known to detach from the cell membrane between 24–72 hours of growth^46^. Samples were incubated with ABPs **1–6-Cy5** and analyzed by in-gel fluorescence. Multiple labelled bands were detected between ~48 and 120 kDa, consistent with known *C. thermocellum* xylanase molecular weights (Supplementary Table 5, Fig. 5a). To identify the labelled proteins, secretome samples were incubated with biotinylated probes (**1–6-Bio**), followed by affinity enrichment and LC-MS/MS analysis^47^.

The cellulosomal xylanases XynZ (92.3 kDa), XynY (119.7 kDa), XynD (71.6 kDa), and XynC (69.5 kDa)^48–50^ were significantly enriched under multiple probe conditions, each showing >3-fold increase over DMSO controls (Fig. 5b–d; Supplementary Fig. 7). This suggests that these enzymes can accommodate glycan decorations in their catalytic clefts - consistent with previous reports of XynY activity on arabinoxylan^49^. Critically, *Ct*Xyl5A (103.1 kDa) and *Ct*Xyn30A (70.3 kDa) were selectively enriched by the decorated probes **4-Bio** and **6-Bio**, but not by the non-decorated probe, confirming that these ABPs discriminate between AXX and GXX activities in native enzyme mixtures (Fig. 5b–d). Interestingly, *Ct*Xyn30A was also moderately enriched by **2-Bio** and **4-Bio**, despite the complete absence of corresponding labelling in purified-enzyme gel assays (Fig. 3b). One possible explanation is co-enrichment arising from the physical association of *Ct*Xyn30A with other xylanases within the cellulosomal architecture.

### Dissecting substrate-specific glycoside hydrolase activities in industrially-relevant microbial free-enzyme systems

Alongside multi-enzyme complexes, we investigated free enzyme proteomes from both bacterial and fungal sources to assess the versatility of our ABP toolkit beyond cellulosomal systems. Among bacteria, *Bacillus subtilis* was selected as a model organism due to its well-documented xylanase production and widespread application in the generation of XOs for prebiotic use^51^ (Supplementary Table 6). In parallel, and in contrast (given the paucity of knowledge of this system), we also examined *Chaetomium globosum*, a saprophytic filamentous fungus with a large and diverse repertoire of lignocellulose-degrading enzymes^52^ (Supplementary Table 7). Despite the enzymatic potential, *C. globosum* remains under characterized relative to other industrial fungi such as *Aspergillus* and *Trichoderma*, positioning it as a promising source for glycoside hydrolases with unexplored substrate specificities and thus a valid testbed for an ABP approach.

To evaluate ABP performance in free-enzyme bacterial systems, *B. subtilis* was cultured on xylan from beechwood for 48 h, and its secretome was incubated with ABPs **1–6-Cy5**. Among all probes, ABP **6-Cy5** (MeGlcA-decorated) showed strong and selective labelling of a protein band between 35–55 kDa, with peak fluorescence at 48 h (Fig. 5e; Supplementary Fig. 8). This band matches the predicted size of XynC, a GH30 xylanase (47 kDa) known to be secreted by *B. subtilis*^53^ (Supplementary Table 6) and was confirmed by enrichment with **6-Bio** in pull-down assays (Fig. 5f). In contrast, the GH11 xylanase from *B. subtilis* was not pulled down by any ABP, likely due to the distinct catalytic itineraries employed by GH11 and GH10/30 families when processing xylan substrates^54–56^. This observation aligns with previous studies showing that GH11 enzymes lack the transition state features required for facile covalent capture by cyclophellitol-based ABPs^10^. These results reinforce the substrate- and mechanism-informed selectivity of the GXX probe, ABP **6**, within a native bacterial secretome.

We next investigated ABP **1-6-Cy5** labelling in fungal free-enzyme systems using *C. globosum*, cultured in Mandels medium containing arabinoxylan as the sole carbon source over a 9-day time course. In-gel fluorescence revealed distinct labelled bands between 35–70 kDa, with signal intensity peaking at day 9 (Fig. 5g; Supplementary Fig. 9). This temporal pattern is consistent with previous reports showing that glycoside hydrolase activity in *C. globosum* peaks during late-stage growth on lignocellulosic substrates such as poplar wood^57^. The high-intensity labelling of the band above 55 kDa by the non-decorated probe (**1-Cy5**) suggests that these proteins are xylanases that do not accept Ara*f* decorations (Supplementary Fig. 9). Among the Ara*f* probes, **3-Cy5**, which showed positive labelling for fungal GH10 (Fig. 3d), shows a stronger band between 35 and 55 kDa, suggesting that this ABP selectively engages Ara*f*-specific fungal enzymes during their maximal induction phase (Supplementary Fig. 9). The molecular weights of the labelled proteins align with predicted GH10 xylanases encoded in the *C. globosum* genome (Supplementary Table 8).

Having identified in-gel labelling, we next sought to identify the *C. globosum* xylanases specific toward Ara*f*-decorated xylans, through biotin pull-down with probe **3-Bio**. Among the seven GH10 xylanases annotated in the *C. globosum* genome, Q2GTY2 was the primary protein pulled down by probe **3-Bio** (>2-fold change), whereas the second xylanase hit is Q2HIC4, with a fold change of only 1.1 (Fig. 5h, Supplementary Table 8).

To understand the molecular basis for the selectivity of probe **3-Bio** toward Q2GTY2, we analysed AlphaFold-predicted structures of *C. globosum* GH10 xylanases (three candidates (Q2GY31, Q2H7X4, and Q2GXB6) were excluded, reflecting absence of a signal peptide or low structural confidence (pLDDT <50) in the catalytic region). Notably, Q2GTY2 shares 62% sequence identity with *Ta*Xyn10 - the only functionally characterized AXX from filamentous fungi^25^- hinting at a potential common recognition of Ara*f*. Indeed, structural alignment of Q2GTY2 with *Ta*Xyn10 (PDB: 2BNJ) revealed conservation of several residues involved in Ara*f* recognition at the -2 subsite (Supplementary Fig. 10a; Supplementary Table 9). One difference is the substitution of Asn25 in *Ta*Xyn10 with Gly47 in Q2GTY2, a change likely to disrupt the water-mediated hydrogen bonding network observed in the reference structure. Crucially, while Ara*f*-interacting residues are partially conserved in Q2GTY2, consistent with gel and biotin labelling, they are either absent or mispositioned in the remaining *C. globosum* GH10 enzymes, confirming their lack of Ara*f* recognition and hence non-enrichment by **3-Bio** (Supplementary Fig. 10).

These ABPP results demonstrate that probes **3,** **4** and **6** enable selective, substrate-informed detection of AXX and GXX activities across diverse microbial proteomes. By combining chemoselective labelling with proteomic identification, our approach provides a functional readout that complements genomic annotation. Beyond validating known systems such as the CAZyme-rich cellulosomes of *C. thermocellum*^58^, we reveal the enzymatic potential of underexplored organisms like *C. globosum*, uncovering glycoside hydrolases with distinct substrate preferences. Together, these findings establish a chemoproteomic framework for substrate-guided enzyme discovery and functional annotation in complex biological systems. Encouraged by the conservation of substitution-interacting residues, we next sought to analyse whether such a probe suite could usefully dovetail with sub-family specific sequence similarity networks (SSN) to enrich functional prediction, using GH10 as a testbed.

### A combined ABPP–SSN approach for functional annotation of xylanases

To explore whether substrate specificity among fungal GH10 xylanases could be inferred from sequence alone, we combined ABPP with sequence similarity network (SSN) analysis. Inspired by the structural similarity between Q2GTY2 and *Ta*Xyn10 in their interactions with Ara*f*, we hypothesized that SSN-guided clustering could help predict AXX activity. Since SSNs group enzymes by sequence-based similarity, they offer a scalable means of identifying functionally related subsets within large enzyme families^59^. Although the ABP approach is clearly going to be best applicable to very distinct subfamilies, such as glucuronoxylanm vs β-glucosidase in family GH30^60^, we were intrigued to see if it had any value in a more “subtle” SSN grouping such as CAZy GH10.

We began by aligning ten filamentous fungal GH10 xylanase sequences with known crystal structures (Supplementary Fig. 11). Considering the residues implicated in Ara*f* binding using *Ta*Xyn10 as a reference, residues at positions 25 and 26 (*Ta*Xyn10 numbering), were poorly conserved. In contrast, positions 46 and 273, which form hydrogen bonds with the O2 atom of Ara*f*, showed high and partial conservation, respectively, revealing their importance for ligand binding^25^. Position 273 draws particular attention, given that a SSN based on this sequence set separated enzymes into two groups which correlated with the identity of residue 273: sequences with aspartate (D) or tyrosine (Y) at this position distributed into two separate clusters, hinting at predictive value for the SSNs with respect to Ara*f* recognition.

**Fig. 6:**
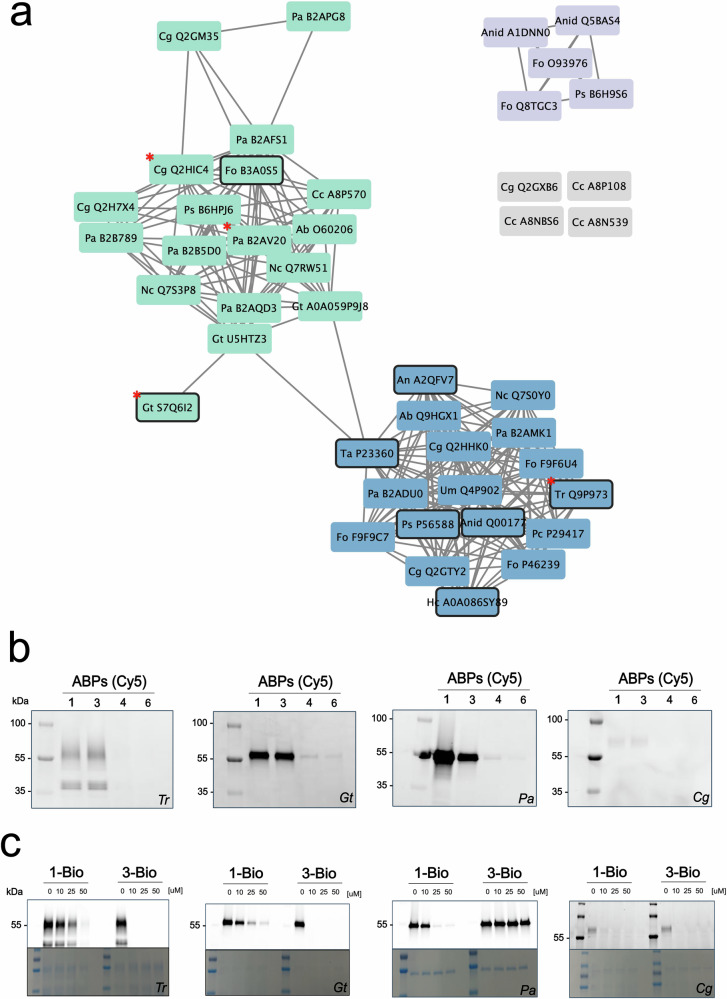
Integration of ABPP and SSN to resolve AXX-specificity in fungal GH10 xylanases. **a** Sequence similarity network (SSN) of fungal GH10 xylanases generated from biomass-degrading fungal genomes. Nodes are coloured by residue identity at position 273: blue (mostly D), green (Y, F, H, M), purple (Y), and grey (unclustered). Enzymes that accept decorations (e.g., *Ta*Xyn10, Q2GTY2, and *Tr*-Q9P973) are located in the blue cluster. Nodes marked with a black outline correspond to enzymes characterized in the literature. Nodes with a red asterisk correspond to enzymes chosen for ABPP assays. **b** In-gel fluorescence analysis of recombinant xylanases expressed in *P. pastoris*. Secretomes were incubated with ABPs **1-Cy5** (non-decorated), **3-Cy5** (Ara*f*), **4-Cy5** (Ara*f*), and **6-Cy5** (MeGlcA). **c** Competition assays comparing substrate preference. Secretomes were pre-incubated with biotinylated ABPs **1-Bio** or **3-Bio**, followed by treatment with fluorescent ABP **1-Cy5**. Stronger competition from **3-Bio** in *Tr*-Q9P973 confirms its decorated substrate preference. Mixed or non-preferential binding was observed for enzymes from the green cluster, consistent with generalist or intermediate substrate recognition profiles. For all gel-based analyses, secretomes were labelled at 30 °C for 1 h with fluorescent ABPs or 2 h with biotinylated probes and visualized by SDS-PAGE. The raw scans and gels are provided in Source Data. In-gel assays were performed in triplicate.

To assess whether these findings could be generalised, we expanded the SSN to include GH10 xylanase sequences from the genomes of saprophytic fungi (Supplementary Table 10). This “enriched” network revealed a dominant “blue” cluster comprising mostly sequences with D at position 273, and a distinct “green” cluster containing enzymes with alternative residues - Y, F, M, or H. A smaller “purple” subgroup with Y273 and unclustered sequences (grey) were also observed (Fig. 6a; Supplementary Fig. 12). Importantly, Ara*f-*recognising *Ta*Xyn10 and Q2GTY2 indeed co-localized in the blue cluster, along with xylanases from *Trichoderma reesei* (Q9P973), *Aspergillus nidulans* (Q00177), and *Penicillium simplicissimum* (P56588). All these enzymes are reported to prefer decorated over non-decorated xylan^61–63^. However, the substrate specificity of enzymes from *Gloeophyllum trabeum* (S7Q6I2) and *Fusarium oxysporum* (B3A0S5) - both found in the green cluster - remains to be addressed, as kinetic data for these enzymes have only been obtained using decorated substrates^64,65^.

To test whether SSN cluster membership indeed corresponds with ABP/substrate selectivity, we selected additional representatives for ABPP analysis. *Tr*-Q9P973 (*T. reesei*) was chosen from the blue cluster, whereas *Gt*-S7Q6I2 (*G. trabeum*), *Pa*-B2AV20 (*P. anserina*), and *Cg*-Q2HIC4 (*C. globosum*) were selected from the green cluster, representing each of Y, F, and H substitutions at position 273, respectively. All were expressed in *Pichia pastoris*, and the secretomes labelled with ABPs **1-Cy5** (non-decorated), **3-Cy5** (Ara*f*), **4-Cy5** (Ara*f*), and **6-Cy5** (MeGlcA). In-gel fluorescence revealed clear labelling by probes **1-Cy5** and **3-Cy5** in *Tr*-Q9P973, *Gt*-S7Q6I2, *Pa*-B2AV20, and *Cg*-Q2HIC4, mirroring earlier results with *Ta*Xyn10 (Fig. 6b).

To probe substrate preferences further, competition assays were performed by pre-incubating secretomes with biotinylated ABPs **1-Bio** or **3-Bio**, followed by treatment with fluorescent ABP **1-Cy5**. In *Tr*-Q9P973, probe **3-Bio** outcompeted probe **1-Cy5**, reinforcing its preference for decorated substrates and supporting the designation of the blue cluster as AXX-enriched. In contrast, xylanases from the green cluster tended to be more generalist and less predictable. For example, *Pa*-B2AV20 was more strongly inhibited by the non-decorated probe, showing it prefers undecorated xylans, whereas *Gt*-S7Q6I2 showed stronger binding to the decorated probe. *Cg*-Q2HIC4 exhibited no clear preference, consistent with a generalist profile (Fig. 6c). This generalist behaviour may explain why *Cg*-Q2HIC4 is not found enriched in pull-down assays of *C. globosum* secretome. Hence, we can conclude that position 273 is associated with, but not solely predictive of, AXX activity in fungal GH10 xylanases.

Notwithstanding the caveat that current ABPs target only the negative subsites of endo-retaining xylanases, combined ABP and SSN analyses show that while the green cluster displayed broader and less predictable substrate specificity, the blue cluster consistently grouped AXXs, indicating that the likelihood of an enzyme that sits in this cluster being an AXX is particularly high. Furthermore, the method presented here enables a reliable extrapolation of xylanase specificity even in settings where ABPP resources are limited.

In this study, we developed and validated a suite of chemically defined, substrate-decorated ABPs capable of selectively targeting the negative subsites of endo-retaining AXX and GXX. These covalent probes enabled the precise functional profiling of retaining xylanases across GH families in purified enzymes as well as complex systems such as cellulosomes and secretomes from diverse bacteria and fungi. Structural analyses revealed the molecular basis of probe selectivity, while proteomic pull-downs confirmed the detection of family- and substrate-specific enzymes in complex mixtures. The expansion of the xylanase ABP toolkit to include ABPs with defined decoration patterns offers a platform for resolving xylanase specificity at the family/subfamily level, enabling the development of precisely balanced enzyme cocktails for tailored XOs production with applications in green chemicals and gut health.

By integrating ABPP with sequence similarity networks (SSNs), we further demonstrated that substrate specificity prediction can be substantially enriched from sequence-level features, enabling the functional annotation of uncharacterized enzymes. Together, our approach provides a chemoproteomic platform for resolving enzyme function in biomass-degrading systems and discovering substrate-specialized hydrolases from both well-studied and underexplored microbial sources. This strategy holds broad potential for accelerating enzyme discovery, guiding biocatalyst engineering, and advancing lignocellulosic bioprocessing.

Future work will expand this strategy to other decorated polysaccharides and GH families, enabling a broader chemoselective toolkit for probing carbohydrate degradation across complex microbial ecosystems and especially looking forward to key industrial and biotechnological contexts. In this context, this approach can be applied in high-throughput protein engineering campaigns to predict desirable functionalities in enzyme variants, thereby enabling targeted enzyme engineering^66^ and the sustainable exploitation of complex biomass resources.

## Methods

### Synthesis of cyclophellitol-derived activity-based probes

Details on the synthesis of inhibitor compounds and ABPs are described in the Supplementary Information.

### Protein production and purification

The codon-optimised gene sequences used in this work were synthesized by Genscript for expression in *Escherichia coli* (*CtXyn30A*, *CtXyl5A*, *XynA*, *Soxyn10A*) or *P. pastoris* (*TaXyn10*) cells. Details on protein sequences and buffers used in the purification steps are described in Supplementary Tables 1 and 11.

#### Bacterial targets

The coding sequences of *CtXyn30A* (cloned in pET28a)*, CtXyl5A* (pET21a)*, XynA* (pET28a), and *SoXyn10A* (pET21a) were inserted into the *Nhe*I/*Xho*I restriction sites of the respective expression vectors. BL21(DE3) *E. coli* cells transformed with pET28a-*CtXyn30A*, pET21a-*CtXyl5A*, pET28a-*Xyna*, and pET21a-*SoXyn10A* were grown in 1 L LB medium at 37 °C/250 rpm until the optical density reached 0.6. At this point, 0.5 mM isopropyl β-D-1-thiogalactopyranoside (IPTG) was added to the cultures to induce protein production at 37 °C/180 rpm. After four hours, the cells were centrifuged at 5400 × *g* for 30 min at 4 °C. Pellets were stored at −20 °C until further use. Pellets were resuspended in 40 mL buffer A (see the buffer A used for each enzyme in Supplementary Table 11) containing cOmplete protease inhibitor cocktail (Roche) and 250 μL Lysozyme (10 mg/mL), and left on ice for 30 min. Only for XynA, the pellet was incubated at 80 °C for 30 min before the sonication step. After incubation, the pellets were sonicated and centrifuged at 18,500 × *g* for 1 h at 4 °C. The supernatant was loaded onto a His-Trap column (Cytiva) equilibrated with buffer A. Bound proteins were eluted using a gradient of buffer B. Fractions from affinity chromatography were evaluated by SDS-PAGE^67^, and those containing protein bands of the expected molecular weight were pooled and concentrated with Vivaspin (cutoff 10 kDa, Sartorius), then applied onto HiLoad Superdex G-75 16/60 (Cytiva) previously equilibrated with the respective buffer. Elution was performed at a flow rate of 1 mL/min in the same buffer.

#### Fungal targets

*TaXyn10* was cloned into the *XhoI*/*Xba*I restriction site of the pPICZα vector, in frame with the alpha-factor signal sequence. The transformation and selection of *P. pastoris* X-33 producing *Ta*Xyn10 were carried out according to the protocol recommended by Invitrogen. Scaled-up production was carried out in 500 mL BMMY medium at 30 °C/200 rpm for 48 h with daily methanol supplementation to a final concentration of 0.5% v/v. Cells were removed by centrifugation at 7800 × *g* for 10 min at 4 °C and discarded. The pH of the supernatant was adjusted to 6.8 with KOH and then filtered through a 0.45 µm cutoff membrane. Subsequently, the supernatant underwent two purification steps as described for the bacterial targets. *Ta*Xyn10 was concentrated using Vivaspin (cutoff 10 kDa, Sartorius).

The protein purification steps were carried out using Äkta Purifier System (Cytiva). Protein purity was assessed by SDS-PAGE, and the final concentration was measured in nanodrop using the molar extinction coefficients retrieved from ProtParam (https://web.expasy.org/protparam) for each enzyme (Supplementary Table 1).

### ABP labelling

A total reaction volume of 10 uL containing 0.05 M buffer, 1 µM enzyme, and 10 µM ABP was incubated for 1 h at 30 °C and 300 rpm. After incubation, the reactions were spun down and stopped by the addition of 5 µL Laemmli (4X) sample buffer (Bio-Rad), followed by boiling at 95 °C for 5 min. The labelled proteins were resolved by SDS-PAGE. Gels were imaged using the Cy5 filter/laser in Typhoon 5 laser scanner (Cytiva). The molecular weight (MW) of proteins was estimated using the Thermo Scientific PageRuler protein ladder 10-250 kDa (S26619). The buffers used for ABP labelling varied with the enzyme: *Ct*Xyl5A, 3-(N-morpholino)propanesulfonic acid (MOPS), pH 7.0; XynA, sodium phosphate, pH 6.0; *Ta*Xyn10, sodium citrate, pH 5.0; *So*Xyn10A, citrate-phosphate, pH 6.0; *Ct*Xyl30A, 2-(N-morpholino)ethanesulfonic acid (MES), pH 6.0. ABP labelling reactions were performed in triplicate.

### Intact mass spectrometry

*Ct*Xyl5A, XynA, *Ta*Xyn10, and *Ct*Xyn30A (12 uM) were incubated with compounds **4-Bio,** **1/3-Bio**, or **S7** (50 uM) in a total volume of 20 uL. Reactions were carried out in 10 mM ammonium acetate buffer (pH 6.8) at 30 °C/300 rpm for 1 h or 16 h. Following incubation, 10 μL of each sample was diluted in 30 μL of 0.1% trifluoroacetic acid (TFA) in water. A 3 μL aliquot was loaded onto a MSPac DS-10 desalting cartridge at a flow rate of 0.3 mL/min using an Acquity I-Class Synapt HPLC (Waters) coupled to a Synapt G2-Si mass spectrometer. After washing with 0.1% formic acid in acetonitrile (Buffer B) and then 0.1% formic acid in water (Buffer A), the protein was eluted using a 7 min gradient from 2–98% of Buffer B in A. Intact protein masses were determined by deconvolution of the spectra using Unidec^68^.

### Crystallization methods

#### Crystal plates set-up

XynA (8 mg/mL) was crystallised using sitting-drop vapour-diffusion with drops set up using a Mosquito robot (SPT LabTech, UK) with 150 nL protein solution plus 150 nL reservoir solution in 96-well format plates (MRC 2-well crystallization microplate, Swissci, Switzerland) equilibrated against 54 µL reservoir solution. Crystals were obtained in PEG-ion commercial screen (Hampton Research) in conditions containing 0.2 M potassium thiocyanate and 20% PEG 3350. Crystals were soaked with ABP **3-Bio** at a final concentration of 500 μM and flash-frozen in liquid nitrogen without cryoprotectant.

*Ct*Xyn30A was crystallised in an optimisation based on published conditions^26^ in 48 well format sitting drop plates (MRC MAXI 48 Well Plate, Swissci) by mixing protein (15 mg/mL) and well solution 1:1, with the final conditions 100 mM Tris-HCl pH 8.5, and 12% PEG 8000. Crystals were soaked overnight with a final concentration of 2 mM inhibitor **S7**, and flash-frozen in liquid nitrogen with 20% glycerol as cryoprotectant.

### Crystal structure solution and refinement

Diffraction data were collected at Diamond Light Source (UK), processed with DIALS^69^, as incorporated in the xia2 pipeline and data reduction carried out using Aimless^70,71^. All computations were carried out using programmes from the CCP4 software suite^72^ incorporated in the CCP4Cloud user interface^73^. The structure of *Ct*Xyn30A was solved by MOLREP^74^ using PDB entry 4CKQ (glucuronoxylan-xylanohydrolase *Ct*Xyn30A from *C. thermocellum*) as a model. The structure was refined by multiple iterations of refinement with REFMAC^75,76^ and model building in COOT^77^.

The structure of XynA was initially solved by MOLREP using the PDB entry 7D88 (GH10 XynA from *Bacillus* sp.) as a model. Structure solution was subsequently repeated with the model created by AlphaFold2^2^ through the CCP4Cloud interface to improve modelling of the N- and C-terminal, which was poorly defined in the initial maps. The AF2 model was separated into the catalytic domain and C- and N-terminal regions, with the catalytic domain used as the first MR model, then the solution was fixed, and the second round of MR was carried out to position the N- and C-termini. This procedure allowed to better trace the N- and C-termini, although the quality of the electron density was not good enough to trace the whole N- and C-termini chain.

Following initial refinement, residual electron density was observed in the active site in the region of the catalytic nucleophile (Glu280) and the bound ABP **3-Bio**. To assess the nature of this density, standard omit and weighted difference omit maps were calculated after removal of both the ligand and the Glu280 side chain, followed by 200 cycles of refinement with B-factors reset to a uniform value prior to refinement. The resulting maps indicated that the electron density at the anomeric carbon is best explained by partial occupancy of both an intact epoxide and a covalently linked glycosyl-enzyme intermediate.

Accordingly, the ligand and Glu280 were modelled in alternative conformations with refined occupancies, representing a dual-occupancy state corresponding to a mixture of non-reacted and covalently reacted probe. The structure was refined by REFMAC5 with TLS option^78^, iterated with model building in COOT. The quality of the final models was validated using Molprobity^79^. Representative omit maps are shown in Supplementary Fig. 4, and the corresponding weighted map coefficients are provided in the Supplementary Information.

For both structures, the ligand library files were prepared using JLigand^80^. The figures were generated using PyMol (The PyMOL Molecular Graphics System, Version 1.2r3pre, Schrödinger, LLC).

### Production of secretomes

#### C. thermocellum and B. subtilis

Freeze-dried cultures of *C. thermocellum* (DSM2360) and *B. subtilis* (DSM10) were reactivated according to recommendations of the DSMZ German Collection of Microorganisms and Cell Cultures. After reactivation, 0.5 mL of *C. thermocellum* culture was inoculated into 5 mL MTC medium^45^ containing 5.0 g/L glucose as a carbon source. Cells were cultivated at 58 °C/180 rpm for 48 h in anaerobiosis. This pre-culture was used to inoculate 10 mL of MTC medium containing cellobiose (0.5%) as carbon source. Cells were grown at 58 °C/180 rpm for 72 h, with aliquots taken at 24, 48, and 72 h. Cultures were centrifuged at 5700 × *g* for 10 min, after which the supernatant was concentrated to 2 mL (Vivaspin cutoff 3 kDa) and the final volume was adjusted to 5 mL with PBS buffer at pH 7.4. This sample was then used for labelling with ABPs.

Following the reactivation step, *B. subtilis* was inoculated into LB medium and grown at 37 °C/200 rpm for 16 h. Then, 1 ml of the pre-culture was used to inoculate 30 mL of inducer medium containing (g/L): NH_4_NO_3_, 5.0; KH_2_PO_4_, 1.0; NaCl, 1.0; MgSO_4_.6H_2_O, 0.6; CaCl_2_, 0.1; FeCl_3_, 0.01 with 5 g.L^−^^1^ xylan from beechwood as carbon source^81^. Cells were grown at 30 °C/140 rpm for 48 h, with 5 mL aliquots taken at 6, 12, 24, and 48 h. Cells were then centrifuged at 8000 × *g* for 15 min at 4 °C, and the supernatant was used for incubation with ABPs.

#### C. globosum

Freeze-dried spores of *C. globosum* (DSM 1962) were spread onto Petri dishes containing BDA medium with a strip of sterile filter paper and incubated at 32 °C for seven days. Three plugs of solid medium containing spores were then inoculated into Mandels medium with 10 g/L arabinoxylan as carbon source^82^. *C. globosum* was cultivated at 32 ^o^C/200 rpm for nine days. Samples were collected at 3, 6, and 9 days. The supernatant was obtained by centrifugation at 10,000 × *g* for 30 min at 4 °C. The pellet was discarded, and the resulting supernatant was used for incubation with ABPs.

Secretomes from *C. thermocellum, B. subtilis, and C. globosum* (10 µL, protein concentration 0.5–1.0 mg/mL) were incubated with compounds **1-6-Cy5** at a final concentration of 10 µM. Reaction termination and gel imaging were done following the same procedure outlined above for pure enzymes. Protein concentration in secretomes was determined by the Bradford quantification assay^83^.

### Proteome analysis

#### Pull-down of AXXs and GXXs from biological samples

After detection of labelled proteins using fluorescent ABPs, 100 μL of secretomes from *C. thermocellum*, *B. subtilis*, and *C. globosum* (0.5–1.0 mg/mL protein) grown on cellobiose, xylan, and arabinoxylan, respectively, were incubated for 2 h at 30 °C with 5 μM **1-6-Bio** dissolved in DMSO for protein identification using pull-down assays^47^. In parallel, reactions containing DMSO only were adopted as negative controls. After incubation, 10 μL of denaturing buffer (40 mM, 2% SDS) was added, and samples were heated at 80 °C for 5 min. After cooling down to RT, 5 μL of 0.5 M iodoacetamide was added and samples were incubated for 30 min at RT in the dark. Proteins were precipitated by addition of 500 μL acetone and incubation at –20 °C for 16 h. Samples were centrifuged at 4000 × *g* for 15 min, and the resulting pellets were air-dried for 1 h to remove residual acetone. Pellets were resuspended in 40 μL of 10 M urea and transferred to 0.5 mL LoBind tubes (Eppendorf), followed by dilution with 360 μL of 0.05% SDS in phosphate buffer, pH 7.4. Biotinylated proteins were captured by adding 40 μL Streptavidin Mag Sepharose magnetic beads (Cytiva) and incubating for 1 hour at RT with shaking. Beads were collected using a magnetic rack and washed twice with 500 μL 2% SDS at 40 °C, once with 500 μL urea at RT, and twice with MilliQ water at RT. Beads were finally resuspended in 20 μL of 0.05 M triethylammonium bicarbonate (TEAB) and supplemented with trypsin (0.5 μg/μL) followed by incubation for 16 h at 30 °C/800 rpm. Pull-down assays were performed with two independent biological replicates.

#### Peptides identification

Following trypsin digestion, beads were collected and transferred to a clean LoBind tube. After acidification with the addition of 2 μL 1% TFA, 5 μL of the sample was added to 15 μL 0.1% TFA before loading onto EvoTips Pure for chromatographic separation. Peptides were separated using a 100 samples-per-day (100SPD) preset gradient on an Evosep One UPLC system, and data-dependent acquisition (DDA) MS data were acquired on a Bruker timsToF HT instrument. Raw data were analysed using MSFragger within FragPipe^84,85^ and searched against *C. thermocellum*, *B. subtilis* or *C. globosum* proteomes downloaded from the Uniprot database. Enrichment in DMSO-added and ABP-added samples was assessed using FragPipe-Analyst^86^. The LFQ-MBR workflow was used with a precursor mass tolerance of ±15 ppm and a fragment mass tolerance of ±0.05 Da. Oxidation and N-terminal acetylation and fixed alkylation of cysteine residues were allowed. FragPipe-Analyst was used for downstream analysis with no normalisation, ‘min’ imputation, and Benjamini-Hochberg false discovery rate (FDR) correction for multiple comparisons. Statistical significance between ABP-treated and DMSO-treated samples was assessed using two-sided moderated t-tests implemented in the Limma framework, with p-values ≤ 0.05 considered statistically significant. Volcano plots showing log_2_ fold change enrichment versus non-adjusted p-values were generated using GraphPrism or excel.

### SSN

The filamentous fungi GH10 xylanases used in SSN analysis were manually curated from the Mycocosm database (https://mycocosm.jgi.doe.gov/mycocosm/home) The regions corresponding to the signal peptide and carbohydrate-binding modules (CBMs) were omitted in this analysis. The Uniprot-identified protein sequences were used as input in the web-based Enzyme Similarity Initiative-Enzyme Similarity Tool (https://efi.igb.illinois.edu/efi-est/) using the module “Accession IDs” to build the SSN. The 44 nodes, clustered at a threshold of 75%, represent individual GH10 xylanases. SSN was visualised using Cytoscape (https://cytoscape.org/).

### Expression of proteins to validate the SSN-ABPP approach

The proteins *Tr*-Q9P973, *Gt*-S7Q6I2, *Pa*-B2AV20, and *Cg*-Q2HIC4 were expressed in *P. pastoris* cells following the same protocol described in “*Protein production and purification - Fungal targets”*. The methanol-induced secretomes were used in the further experiments with ABPs.

### Competition assays

Secretomes of *P. pastoris* expressing *Tr*-Q9P973, *Gt*-S7Q6I2, *Pa*-B2AV20, and *Cg*-Q2HIC4 were treated with 0, 10, 25, or 50 μM of compounds **1-Bio** and **3-Bio** for 2 h at 30 °C, followed by incubation with the fluorescent ABP **1-Cy5** to assess prior binding to the inhibitors. The protein concentrations were adjusted to 0.5–1.0 mg/mL. Fluorescent bands were visualised as pointed out in “ABP labelling”.

### Reporting summary

Further information on research design is available in the Nature Portfolio Reporting Summary linked to this article.

## Supplementary information

Supplementary Information

Reporting Summary

Transparent Peer Review file

## Source data

Source Data

## Data Availability

The atomic coordinates have been deposited in the Protein Data Bank (PDB) under the accession codes 9RHV (XynA); 9RDG (*Ct*Xyn30A). The mass spectrometry proteomics data have been deposited to the ProteomeXchange Consortium via the PRIDE partner repository with the dataset identifiers PXD065060 (*C. thermocellum*), PXD065065 (*B. subtilis*), and PXD065106 (*C. globosum*). Source data are provided with this paper.
