# Supplementary material for: A chemoproteomic biotechnological toolkit for resolving xylanase specificity in decorated xylan
Source: Nat Commun. 2026 Jun 22;17:7860. doi: 10.1038/s41467-026-74484-0 (PMC13444322; doi:10.1038/s41467-026-74484-0)
Supplement: Supplementary file 1 [file 41467_2026_74484_MOESM1_ESM.pdf]

A Chemoproteomic Biotechnological Toolkit for Resolving Xylanase Specificity in Decorated Xylan

Thamy L. R. Corrêa[1&], Zirui Li[2&], Olga Moroz[1], Isabelle B. Pickles[1], Andrey A. Lebedev[3], Saeed Akkad[1,4], Lianne I. Willems[1,4], Jeroen D. C. Codée[2], Herman S. Overkleeft[2*], Gideon J. Davies[1*]

[1] York Structural Biology Laboratory, Department of Chemistry, University of York, York, North Yorkshire, YO10 5DD, UK

[2] Department of Bio-organic Synthesis, Leiden Institute of Chemistry, Leiden University, Einsteinweg 55, 2333 CC Leiden, The Netherlands

[3] CCP4, STFC Rutherford Appleton Laboratory, Harwell Oxford, Didcot OX11 0QX, UK

[4] York Biomedical Research Institute, Department of Chemistry, University of York, North Yorkshire, YO10 5DD, UK

[*]h.s.overkleeft@lic.leidenuniv.nl, gideon.davies@york.ac.uk

[&] These authors contributed equally

Supplementary information 1. Experimental and analytical details on ABPs synthesis

Chemical synthesis

Schemes for synthesis of inhibitors and probes

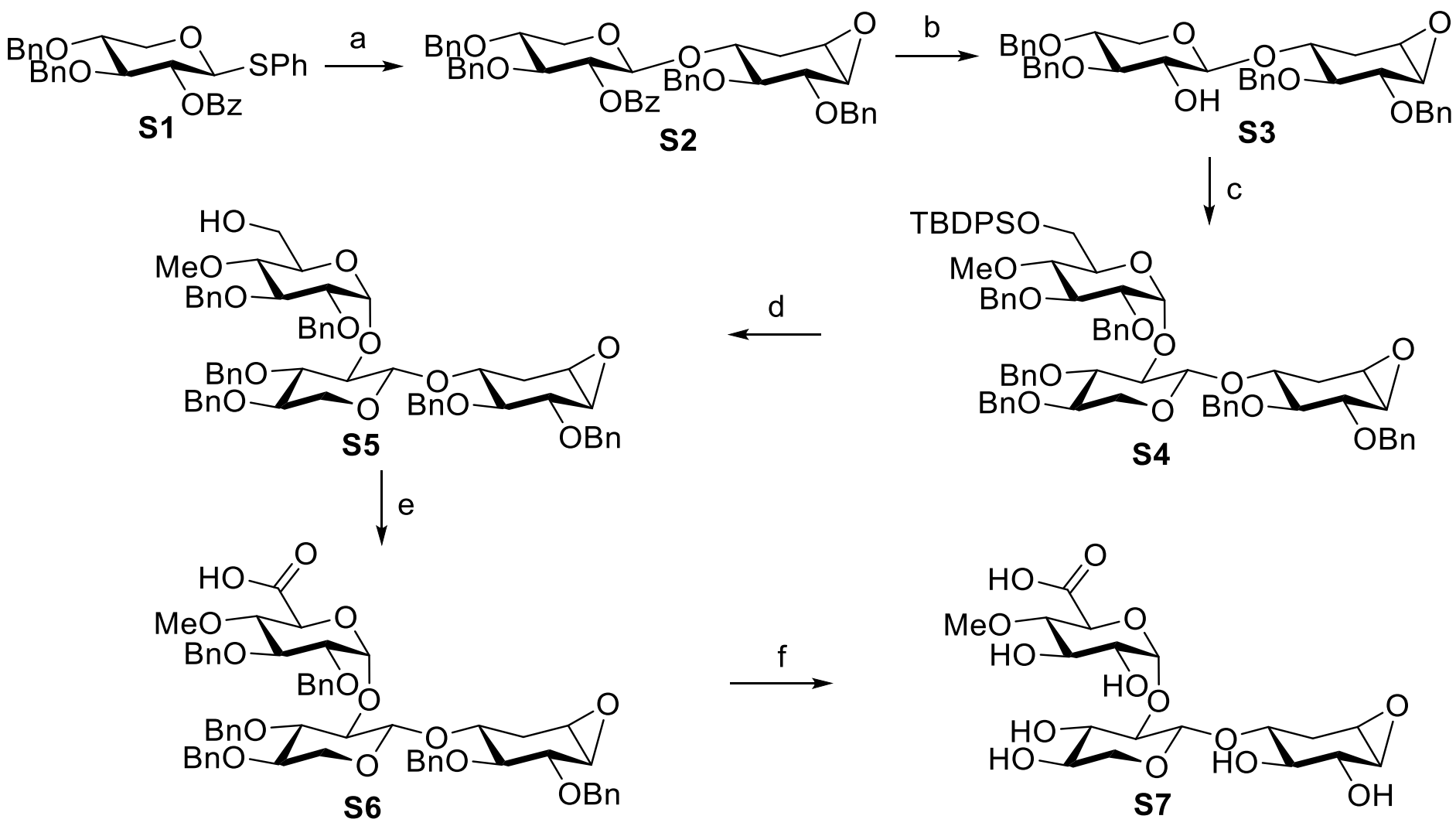

Supplementary Figure 1. **Synthesis of inhibitor S7**. Reagents and conditions: a) **13**, NIS, TMSOTf, DCM, 81%. b) NaOMe, DCM/MeOH = 1/1, 78% c) **18**, DMF, TfOH, DCM, -78 °C to 0 °C, 69%. d) TBAF, THF, reflux, 80%. e) TEMPO, BAIB, DCM/^{t}BuOH/H_2O = 4/4/1, 62%. f) $Pd(OH)_2$, H_2, dioxane/H_2O = 4/1, quant.

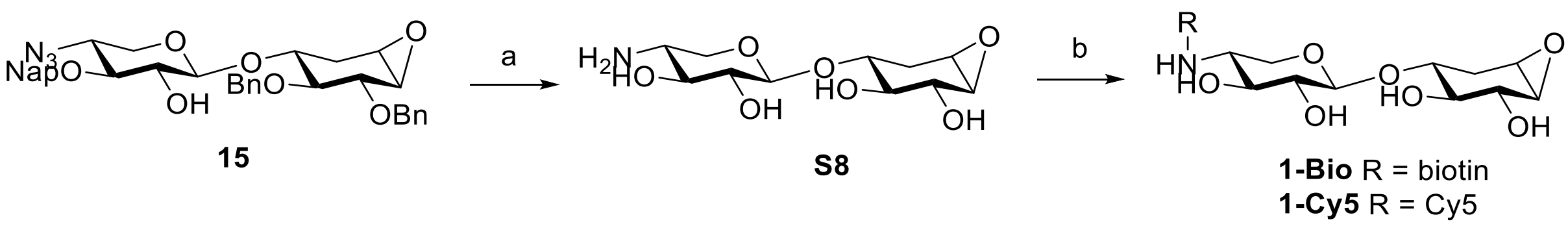

Supplementary Figure 2. **Synthesis of ABP 1.** Reagents and conditions: a) **13**, NIS, TMSOTf, DCM, 81%. b) NaOMe, DCM/MeOH = 1/1, 78% c) **18**, DMF, TfOH, DCM, -78 °C to 0 °C, 69%. d) TBAF, THF, reflux, 80%. e) TEMPO, BAIB, DCM/^{t}BuOH/H_2O = 4/4/1, 62%. f) $Pd(OH)_2$, H_2, dioxane/H_2O = 4/1, quant.

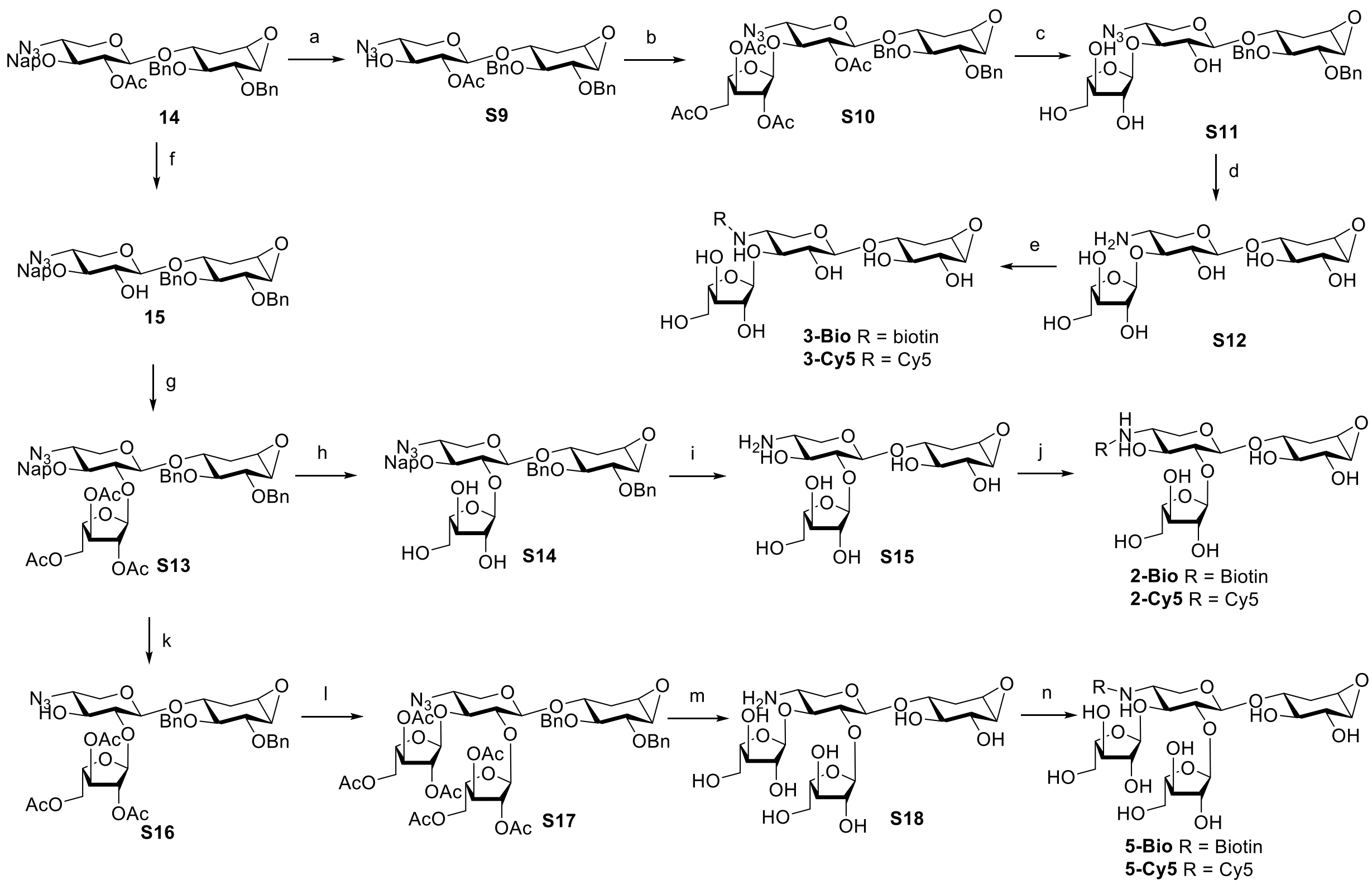

Supplementary Figure 3. **Synthesis of ABPs 2, 3, and 5**. Reagents and conditions: a) DDQ, DCM/H_2O = 19/1, 79%. b) **23**, TMSOTf, NIS, -40 ℃, DCM, 68%. c) NaOMe, DCM/MeOH = 1/2, 90%. d) $Pd(OH)_2$, H_2, TFA, dioxane/H_2O = 3/1, quant.. e) Cy5COOH or biotin, pentafluorophenyl trifluoroacetate, DIPEA, DMF, **3-Bio** 26%, **3-Cy5** 25%. f) NaOMe, DCM/MeOH = 1/1, 85%. g) **23**, TMSOTf, NIS, -40 ℃, DCM, 75%. h) NaOMe, DCM/MeOH = 1/2, 85%. i) $Pd(OH)_2$, H_2, TFA, dioxane/H_2O = 3/1, quant.. j) Cy5COOH or biotin, pentafluorophenyl trifluoroacetate, DIPEA, DMF, **2-Bio** 28%, **2-Cy5** 21%. k) DDQ, DCM/H_2O = 19/1, 76%. l) **23**, TMSOTf, NIS, -40 ℃, DCM, 61%. m) (i) NaOMe, DCM/MeOH = 1/2; (ii) $Pd(OH)_2$, H_2, TFA, dioxane/H_2O = 1.5/1, 83%. n) Cy5COOH or biotin, pentafluorophenyl trifluoroacetate, DIPEA, DMF, **5-Bio** 28%, **5-Cy5** 20%.

Experimental methods

Known compounds **13**[1], **S1**[2], **21**[2], **23**[3], **28**[4] were synthesized following procedures previously described and their spectroscopic data are in agreement with those previously reported.

General experimental details

All reagents were of experimental grade and were used without further purification unless stated otherwise. Dichloromethane and tetrahydrofuran (THF) were stored over 3 Å molecular sieves and *N,N*-dimethylformamide (DMF) was stored over 4 Å molecular sieves, which were dried *in vacuo* before use. All reactions were performed under an argon or N_2 atmosphere unless stated otherwise. Reactions were monitored by analytical thin layer chromatography (TLC) using Merck aluminum sheets pre-coated with silica gel 60 with detection by UV-absorption (254 nm) and by spraying with a solution of $(NH_4)_6Mo_7O_{24}\cdot H_2O$ (25 g L^{-1}) and $(NH_4)_4Ce(SO_4)_4\cdot H_2O$ (10 g/mL) in 10% sulfuric acid followed by charring at ~150 °C or by spraying with an aqueous solution of $KMnO_4$ (7%) and K_2CO_3 (2%) followed by charring at ~150 °C. Column chromatography was performed manually using Screening Device b.v. silica gel 60 (0.04-0.063 mm) in the indicated solvents. LC-MS analysis was performed on a LCQ Advantage Max (Thermo Finnigan) ion-trap spectrometer (ESI^+) coupled to a Surveyor HPLC system (Thermo Finnigan) equipped with a C18 column (Gemini, 4.6 mm x 50 mm, 5 µM particle size, Phenomenex). The applied buffers were H_2O, acetonitrile (MeCN) and 1% aqueous trifluoroacetic acid (TFA). ^{1}H-NMR and ^{13}C-NMR spectra were recorded on Bruker AV-400 (400/101 MHz) and Bruker AV-500 (500/126 MHz) spectrometers in the given solvent. Chemical shifts (δ) are given in ppm relative to tetramethylsilane (TMS) as internal standard (^{1}H NMR in $CDCl_3$) or the residual signal of the deuterated solvent. Coupling constants (*J*) are given in Hz. All ^{13}C APT NMR spectra are proton decoupled. The following abbreviations are used to describe peak patterns when appropriate: s (singlet), d (doublet), t (triplet), q (quartet), m (multiplet), Ar (aromatic), Cq (quarternary carbon). High-resolution mass spectrometry (HRMS) analysis was performed with a LTQ Orbitrap mass spectrometer (Thermo Finnigan), equipped with an electronspray ion source in positive mode (source voltage 3.5 kV, sheath gas flow 10 mL min^{-1}, capillary temperature 250 °C) with resolution R = 60000 at m/z 400 (mass range m/z = 150 – 2000) and dioctyl phthalate (m/z = 391.28428) as a "lock

mass”. The high-resolution mass spectrometer was calibrated prior to measurements with a calibration mixture (Thermo Finnigan).

General procedure A

To a solution of the appropriate carboxylic acid (12.5 µmol) in dry DMF (100 µL) was added DIPEA (10 µL, 50 µmol, 4 eq) and pentafluorophenyl trifluoroacetate (3.3 µL, 18.8 µmol, 1.5 eq). The mixture was allowed to stir at ambient temperature for 3 h and monitored by LC-MS. The reaction mixture was then quenched with water (1.25 µL) and transferred to an Eppendorf containing a solution of the appropriate amine in dry DMF (150 µL). The reaction was monitored by LC-MS and stirred overnight. Additional DIPEA (2 eq) was added and the reaction was stirred for additional 5h. The mixture was then concentrated *in vacuo* and purified by semi-preparative HPLC eluting with a linear gradient of solution A (MeCN) in solution B (50 mM NH_4OAc in H_2O). The fractions were then combined, concentrated, co-evaporated with MilliQ/MeCN (1/1, 3 times), re-dissolved in MilliQ/tBuOH and lyophilized to afford the product.

Compound S2

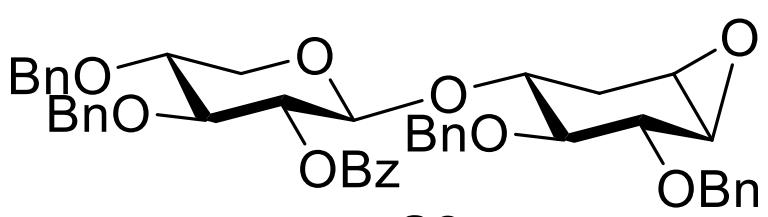

S2

Donor **S1** (174 mg, 0.33 mmol) and acceptor **13** (98 mg, 0.3 mmol) were co-evaporated with toluene for 3 times and re-dissolved in dry DCM (2.0 mL), after which NIS (80 mg, 0.36 mmol) was added under and the solution was cooled to -70°C under nitrogen. To the stirring solution was then added TMSOTf (0.03 mmol, 0.2 mL of 0.15 M solution in DCM) and the reaction mixture was warmed to -50 °C over 30 minutes. Et_3N (0.1 mL) was then added and the solution was diluted with EtOAc, washed with sat. aq. $NaHCO_3$, 10% aq. $Na_2S_2O_3$ and brine, dried over $MgSO_4$, filtered and concentrated under *in vacuo*. Flash column chromatography (Et_2O/pentane, 1/9 to 3/7, v/v) provided product **S2** (162 mg, 0.22 mmol, 73%). ^{1}H NMR (400 MHz, $CDCl_3$) 8.05 – 7.94 (m, 2H), 7.60 – 7.52 (m, 1H), 7.43 (t, J = 7.7 Hz, 2H), 7.38 – 7.22 (m, 15H), 7.14 (dtd, J = 7.2, 4.8, 2.2 Hz, 5H), 5.25 – 5.13 (m, 1H), 4.95 (d, J = 10.9 Hz, 1H), 4.82 – 4.53 (m, 9H), 3.97 (dd, J = 12.0, 3.8 Hz, 1H), 3.79 – 3.68 (m, 4H), 3.33 (dd, J = 10.1, 7.7 Hz, 1H), 3.28 – 3.17 (m, 1H), 3.09 (q, J = 2.2 Hz, 1H), 3.04 (d, J = 3.6 Hz, 1H), 2.41 (ddd, J = 14.6, 5.3, 2.4 Hz, 1H), 1.56 (ddd, J = 14.6, 10.5, 1.5 Hz, 1H). ^{13}C NMR (101 MHz, $CDCl_3$) δ 165.29, 138.93, 138.02, 137.97, 137.91, 133.23, 129.95, 129.78, 128.56, 128.52, 128.51, 128.31, 128.28, 128.18, 128.10, 128.06, 127.99, 127.94, 127.91, 127.87,

127.67, 127.40, 99.70, 82.50, 80.29, 79.35, 77.52, 75.31, 74.45, 73.84, 73.41, 73.22, 73.15, 63.33, 53.99, 53.41, 29.87. HRMS (ESI) m/z: $[M+NH_4]^+$ calculated for $C_{46}H_{50}O_9N$ 760.3480, found 760.3477.

Compound S3

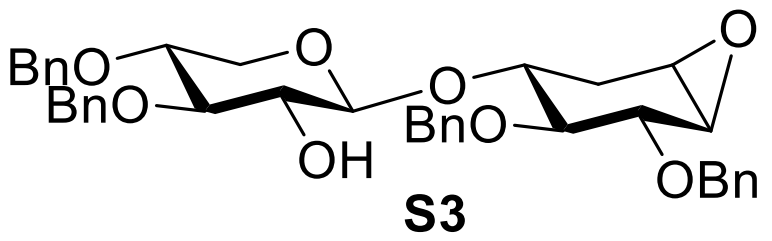
S3

To a solution of compound **S2** (160 mg, 0.22 mmol) in DCM/MeOH (2.0 mL, 2/1, v/v) was added NaOMe (5.4M, 0.02 mL) and the solution was stirred at rt overnight. The reaction was then quenched with NH_4Cl and concentrated *in vacuo*. Flash column chromatography (EtOAc/pentane, 1/4 to 2/5, v/v) afforded the product **S3** (106 mg, 78%).

^{1}H NMR (400 MHz, $CDCl_3$) δ 7.47 – 7.24 (m, 20H), 4.91 (d, J = 11.1 Hz, 1H), 4.83 (s, 2H), 4.81 – 4.62 (m, 5H), 4.50 – 4.40 (m, 1H), 4.08 – 3.97 (m, 1H), 3.92 – 3.80 (m, 2H), 3.63 – 3.53 (m, 3H), 3.46 (dd, J = 10.2, 7.7 Hz, 1H), 3.33 – 3.22 (m, 2H), 3.17 (d, J = 3.6 Hz, 1H), 3.07 – 2.76 (m, 1H), 2.67 (ddd, J = 14.5, 5.2, 2.4 Hz, 1H), 1.86 (ddd, J = 14.5, 10.4, 1.5 Hz, 1H). ^{13}C NMR (101 MHz, $CDCl_3$) δ 138.51, 137.94, 137.71, 128.54, 128.52, 128.45, 128.22, 128.16, 128.01, 127.97, 127.95, 127.87, 127.77, 127.53, 100.60, 82.28, 81.45, 79.73, 76.84, 74.95, 74.43, 73.27, 72.88, 71.86, 70.84, 62.80, 53.80, 53.57, 30.45. HRMS (ESI) m/z: $[M+NH_4]^+$ calculated for $C_{39}H_{46}O_8N$ 656.3218, found 656.3213.

Compound S4

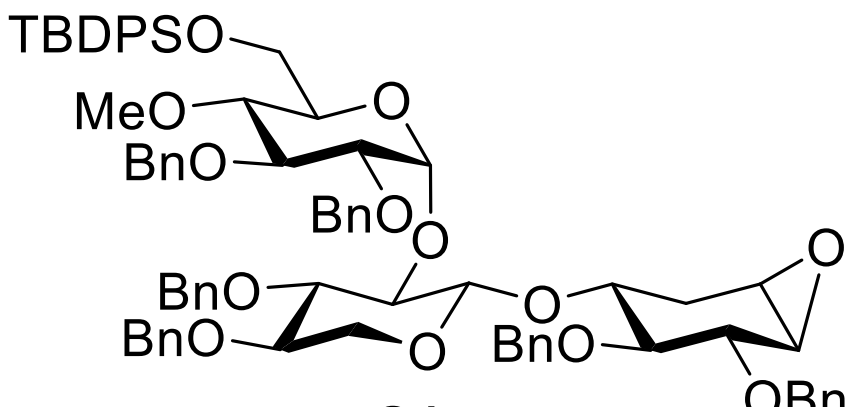
S4

Compound **S3** (70 mg, 0.11 mmol) and compound **18** (120 mg, 0.154 mmol) were co-evaporated with toluene for 3 times and dissolved in dry DCM (1 mL). The mixture was stirred over fresh flame-dried 3Å molecular sieves under argon, followed by the addition of DMF (0.19 mL, 2.46 mmol). The solution was then cooled to -78 °C, after which TfOH (23 mg, 0.154 mmol) was added. After 30 minutes, the reaction mixture was allowed to stir at 0 °C until TLC analysis showed complete conversion of compound **S3**. The reaction was then quenched with Et_3N, filtered and concentrated *in vacuo*, purified by flash column chromatography (pentane : EtOAc = 10:1 to 4:1) to afford the product **S4** (94 mg, 69%) as a colorless oil. ^{1}H NMR (400 MHz, $CDCl_3$) δ 7.65 (td, J = 7.8, 1.5 Hz, 4H, ArH), 7.45 – 7.28 (m, 28H), 7.27 – 7.15 (m, 8H, ArH), 5.51 (d, J = 3.6 Hz, 1H, H1'', α-1,2), 5.00 – 4.85 (m, 5H, CH_2Bn), 4.79 – 4.68 (m, 5H, CH_2Bn), 4.64 (d, J = 11.6 Hz, 1H, CH_2Bn), 4.55 (d, J = 11.6 Hz, 1H, CH_2Bn), 4.50 (d, J = 6.9 Hz, 1H, H1', β-1,4), 4.10 – 3.97 (m, 2H), 3.88 (ddd, J = 13.6, 11.1, 5.0 Hz, 2H), 3.80 (dd, J = 7.7, 0.8

Hz, 1H), 3.68 – 3.53 (m, 9H), 3.44 – 3.39 (m, 1H), 3.35 (dd, J = 10.2, 7.6 Hz, 1H), 3.13 (dd, J = 11.7, 9.4 Hz, 1H), 3.04 (d, J = 3.6 Hz, 1H, H1), 2.82 – 2.71 (m, 1H, H6), 2.45 (ddd, J = 14.5, 5.3, 2.5 Hz, 1H, H5b), 1.40 (ddd, J = 14.7, 10.3, 1.5 Hz, 1H, H5a), 1.02 (s, 9H, tBu). ^{13}C NMR (101 MHz, $CDCl_3$) δ 138.86, 138.65, 138.12, 138.09, 138.06, 135.84, 135.69, 133.90, 133.58, 129.53, 129.43, 128.67, 128.58, 128.56, 128.54, 128.50, 128.33, 128.28, 128.12, 128.01, 127.98, 127.90, 127.73, 127.64, 127.60, 127.58, 127.51, 127.49 (Ar), 100.29 (C1', β-1,4), 96.23 (C1'', α-1,2), 82.47 (C3''), 82.15 (C3'), 81.34 (C3), 80.44 (C2''), 79.75 (C4''), 79.60 (C2), 79.14 (C4'), 75.76 (CH_2Ar), 75.66 (CH_2Ar), 75.33 (C2'), 74.68 (CH_2Ar), 74.22 (CH_2Ar), 73.33 (CH_2Ar), 73.22 (CH_2Ar), 71.22 (C4), 71.14 (C5''), 63.73 (C5'), 62.36 (C6'), 60.54 (OMe), 53.90 (C1), 53.36 (C6), 28.88 (C5), 26.94 (CH_3), 19.42 ($C(CH_3)_3$). HRMS (ESI) m/z: $[M+NH_4]^+$ calculated for $C_{76}H_{88}O_{13}SiN$ 1250.6019, found 1250.6016.

Compound S5

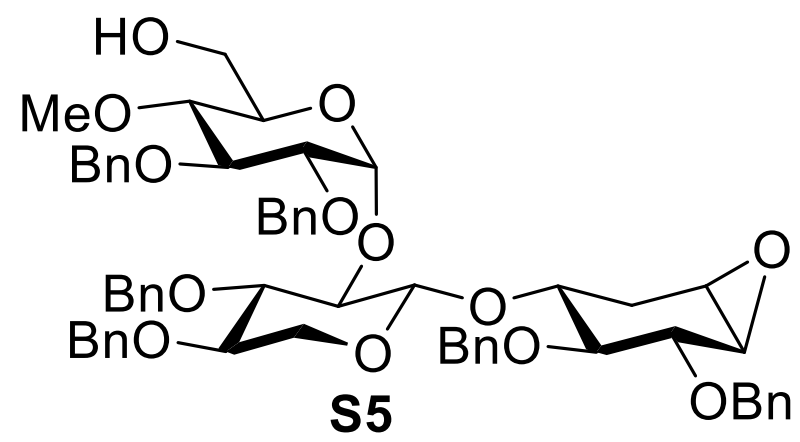

S5

To a solution of compound **S4** (88 mg, 0.072 mmol) in dry THF (1 mL) was added TBAF solution (1 M in THF, 0.108 mL, 0.108 mmol). The reaction mixture was allowed to stir at reflux for 6 hours and then quenched with sat. aq. NH_4Cl at 0 ℃. The mixture was then diluted with EtOAc, washed with water, and brine, dried over $MgSO_4$, filtered and concentrated under reduced pressure. Flash column chromatography (pentane/EtOAc = 1.5/1, v/v) afforded the product **S5** (57 mg, 80%) as an oil. ^{1}H NMR (500 MHz, $CDCl_3$) 7.38 – 7.22 (m, 30H), 5.41 (d, J = 3.6 Hz, 1H, H1'', α-1,2), 5.01 – 4.79 (m, 5H), 4.76 – 4.63 (m, 6H), 4.57 (d, J = 11.6 Hz, 1H), 4.49 (d, J = 6.5 Hz, 1H, H1', β-1,4), 4.03 (dt, J = 10.2, 3.0 Hz, 1H), 3.96 (dd, J = 9.8, 9.0 Hz, 1H), 3.92 – 3.81 (m, 2H), 3.76 (d, J = 7.6 Hz, 1H), 3.62 (dtt, J = 9.9, 7.1, 4.0 Hz, 3H), 3.53 – 3.42 (m, 5H), 3.35 – 3.20 (m, 3H), 3.17 – 3.07 (m, 1H), 3.02 (d, J = 3.6 Hz, 1H, H1), 2.84 – 2.75 (m, 1H, H6), 2.44 (ddd, J = 14.5, 5.2, 2.5 Hz, 1H, H5a), 1.43 – 1.39 (m, 1H, H5b). ^{13}C NMR (126 MHz, $CDCl_3$) δ 138.83, 138.82, 138.41, 138.05, 138.03, 138.01, 128.78, 128.62, 128.56, 128.46, 128.34, 128.28, 128.27, 128.05, 128.03, 128.01, 127.93, 127.83, 127.80, 127.67, 127.63, 127.57, 100.16 (C1', β-1,4), 96.23 (C1'', α-1,2), 82.18, 82.06, 82.02, 80.02, 79.89, 79.45, 79.21, 75.77, 75.68, 75.53, 74.91, 74.09, 73.38, 73.15, 71.05, 70.69, 63.59, 61.43, 60.68, 53.87 (C1), 53.30 (C6), 28.95 (C5). HRMS (ESI) m/z: $[M+Na]^+$ calculated for $C_{60}H_{66}O_{13}Na$ 1017.4401, found 1017.4399.

Compound S6

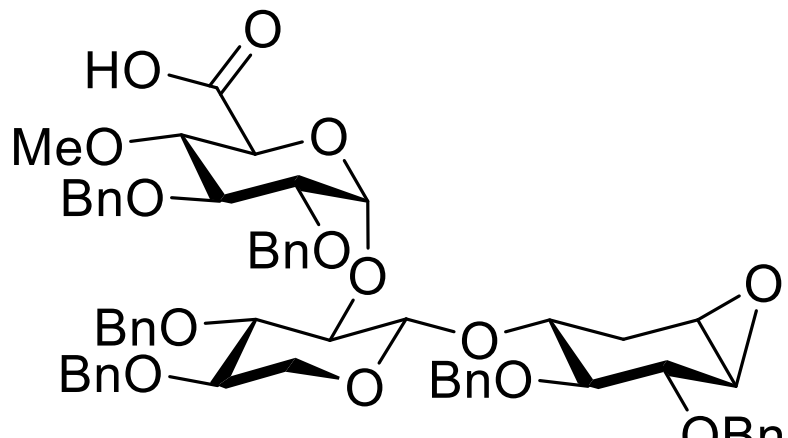

S6

Compound **S5** (54 mg, 0.054 mmol) was dissolved in a mixture of DCM/tBuOH/H2O (4/4/1, v/v/v) (1 mL) and was cooled to 0 ℃. To the solution was added TEMPO (1.7 mg, 0.011 mmol) and BAIB (14.8 mg, 0.135mmol) at the same temperature. The reaction mixture was stirred at 0 ℃ overnight and then quenched with aq. $Na_2S_2O_3$, diluted with water, and extracted with DCM (3 x 5 mL). The combined organic layers were washed with sat. aq. $NaHCO_3$ and brine, dried over $MgSO_4$, filtrated and concentrated *in vacuo*. Flash column chromatography (DCM/MeOH = 20/1, v/v) afforded the product **S6** (34 mg, 62%) as an oil. ^{1}H NMR (400 MHz, $CDCl_3$) δ 7.50 – 7.17 (m, 30H), 5.50 (d, J = 3.5 Hz, 1H, H1'', α-1,2), 5.00 (dd, J = 25.9, 10.8 Hz, 2H), 4.90 – 4.56 (m, 10H), 4.50 (d, J = 6.9 Hz, 1H, H1', β-1,4), 4.02 – 3.78 (m, 4H), 3.74 – 3.61 (m, 3H), 3.54 (s, 4H), 3.40 – 3.27 (m, 2H), 3.20 – 3.10 (m, 1H), 3.05 (d, J = 3.6 Hz, 1H, H1), 2.84 – 2.77 (m, 1H, H6), 2.44 (ddd, J = 14.2, 5.2, 2.4 Hz, 1H, H5a), 1.45 (ddd, J = 14.6, 10.5, 1.6 Hz, 1H, H5b). ^{13}C NMR (101 MHz, $CDCl_3$) δ 173.39 (C6'), 138.64, 138.15, 137.83, 137.69, 128.63, 128.57, 128.54, 128.51, 128.48, 128.40, 128.21, 128.04, 128.02, 127.95, 127.92, 127.89, 127.84, 127.53, 127.47, 100.31 (C1'', β-1,4), 95.93 (α-1,2), 82.15, 81.46, 81.36, 81.17, 79.44, 79.01, 78.95, 75.55, 75.44, 75.32, 74.97, 74.06, 73.37, 72.95, 71.59, 68.60, 63.43, 60.88, 53.75 (C1), 53.21 (C6), 29.17 (C5). HRMS (ESI) m/z: $[M+Na]^+$ calculated for $C_{60}H_{64}O_{14}Na$ 1031.4194, found 1031.4197.

Compound S7

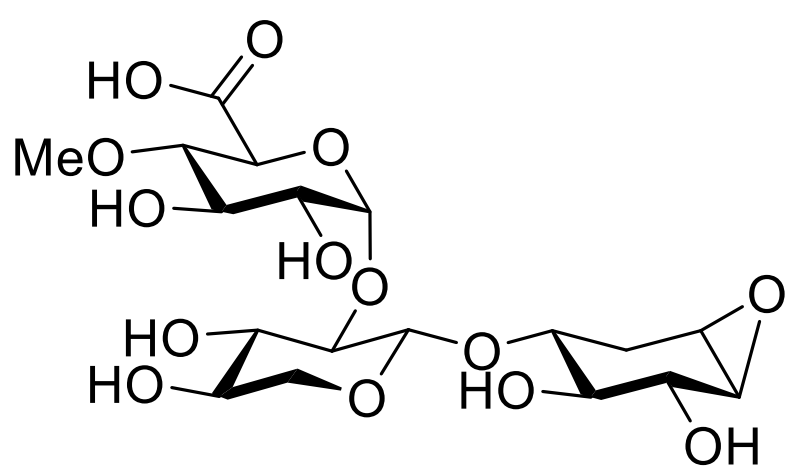

S7

Compound **S6** (34 mg, 0.034 mmol) was dissolved in a mixture of dioxane (1 mL) and H_2O (0.25mL). The solution was purged with nitrogen for 3 minutes, after which $Pd(OH)_2/C$ (20 wt%, 36 mg, 51 μmol) was added. While stirring vigorously, the flask was then purged with hydrogen for 5 minutes. The reaction mixture was stirred under hydrogen atmosphere and was monitored by LC-MS hourly. After 6 hours, LC-MS analysis indicated full conversion and the reaction mixture was then filtered over celite, concentrated *in vacuo*, purified by flash column chromatography (DCM/MeOH/ammonia water, 10/1/0.5, v/v/v) to give product **S7** (15.8 mg, quant.) as a white powder. ^{1}H NMR (500 MHz, D_2O) δ 5.28 (d, J = 3.9 Hz, 1H, H1'', α-1,2), 4.49 (d, J = 7.5 Hz, 1H, H1', β-1,4), 4.22 (d, J = 10.1 Hz, 1H), 3.84 (dd, J = 11.6, 5.5 Hz, 1H),

3.74 – 3.63 (m, 2H), 3.58 – 3.44 (m, 3H), 3.43 – 3.27 (m, 7H), 3.22 – 3.05 (m, 3H), 2.63 (ddd, J = 15.5, 5.4, 2.6 Hz, 1H, H5a), 1.85 (ddd, J = 14.8, 10.4, 1.7 Hz, 1H, H5b). ^{13}C NMR (126 MHz, D_2O) δ 100.78 (C1’, β-1,4), 97.33 (C1’’, α-1,2), 82.50, 76.18, 74.61, 74.45, 73.24, 72.27, 72.06, 71.32, 71.14, 69.46, 64.91, 59.96, 56.20 (C1), 54.84 (C6), 28.50 (C5). HRMS (ESI) m/z: $[M+Na]^+$ calculated for $C_{18}H_{28}O_{14}Na$ 491.1377, found 490.1376.

Compound 8

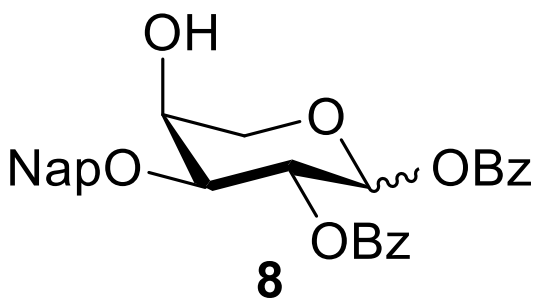

8

To a solution of compound **7** (0.72 g, 2 mmol) in toluene (20 mL) was added Bu_2SnO (0.6 g, 2.4 mmol). The mixture was allowed to stir at 90 °C for 1h, after which the reaction mixture was cooled to rt and subsequently concentrated under reduced pressure. To a solution of the crude in DMF (16 mL) was added 2-naphthylene bromide (0.53 g, 2.4 mmol) and cesium fluoride (0.49 g, 3.2 mmol). The reaction mixture was allowed to stir at rt overnight, then diluted with H_2O (160 mL) and extracted with Et_2O (3 x 60 mL). The combined organic layers were washed with brine, dried over $MgSO_4$, filtered and concentrated *in vacuo*. Flash column chromatography (pentane/EtOAc, 5:1 to 2:1) over silica afforded product **8** as a colorless oil (0.55 g, 55% over 2 steps). ^{1}H NMR (300 MHz, $CDCl_3$): δ 8.08 – 7.22 (m, 19H), 6.15 (d, J = 4.3 Hz, 1H), 5.74 (dd, J = 5.8, 4.3 Hz, 1H), 5.02 – 4.83 (m, 2H), 4.25 – 4.08 (m, 2H), 4.03 (dd, J = 5.8, 3.2 Hz, 1H), 3.83 – 3.73 (m, 1H), 2.62 (d, J = 6.9 Hz, 1H). ^{13}C NMR (101 MHz, $CDCl_3$): 13C NMR (101 MHz, CDCl3) δ 165.54, 164.67, 134.56, 133.52, 133.29, 133.13, 133.12, 129.87, 129.77, 129.68, 129.48, 129.42, 128.56, 128.47, 128.42, 127.89, 127.78, 127.73, 126.99, 126.36, 126.25, 126.13, 125.78, 91.55, 74.30, 72.18, 68.90, 66.75, 64.28, 60.43, 21.08, 14.22. HMRS (ESI) m/z: $[M+Na]^+$ calculated for $C_{30}H_{26}O_7Na$ 521.1571, found 521.1567.

Compound 9

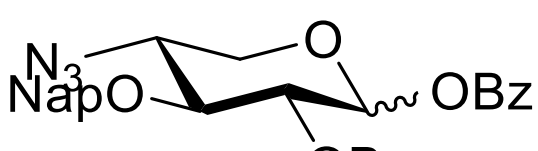

9

To a solution of compound **8** (0.31 g, 0.62 mmol) in dry DCM (6 mL) was added pyridine. Tf_2O was added to the mixture dropwise at -20 °C and the reaction was allowed to stir at the same temperature for 1h, after which the reaction mixture was diluted with DCM (15 mL), washed with H_2O (3 x 6 mL), dried over $MgSO_4$, filtered and concentrated at *in vacuo*. The crude was then re-dissolved in DMF (15 mL) and NaN_3 (0.4 g, 6.2 mmol) was added. The reaction mixture was stirred at rt overnight and then diluted with H_2O (15 mL), extracted with Et_2O (3 x 5 mL). The combined organic layers were washed with brine, then dried over $MgSO_4$, filtered and concentrated *in vacuo* and purified by flash column chromatography (pentane/EtOAc, 8:1) to give product **9** as a colorless

oil (0.28 g, 88% over 2 steps). ^{1}H NMR (400 MHz, $CDCl_3$): 8.09 – 7.98 (m, 2H), 7.94 – 7.85 (m, 2H), 7.81 – 7.32 (m, 15H), 6.61 (d, J = 3.5 Hz, 1H), 5.47 (dd, J = 9.7, 3.6 Hz, 1H), 5.08 (d, J = 11.2 Hz, 1H), 4.99 (d, J = 11.2 Hz, 1H), 4.21 (t, J = 9.3 Hz, 1H), 4.02 – 3.90 (m, 2H), 3.80 – 3.71 (m, 1H). ^{13}C NMR (101 MHz, $CDCl_3$): δ 165.30, 164.50, 134.57, 133.77, 133.47, 133.14, 133.07, 129.80, 129.69, 129.20, 129.01, 128.70, 128.47, 128.32, 127.92, 127.69, 127.20, 126.12, 126.05, 126.03, 90.74, 78.09, 75.43, 72.15, 62.32, 60.98. HRMS (ESI) m/z: $[M+Na]^+$ calculated for $C_{30}H_{25}N_3O_6Na$ 546.1636, found 546.1633.

Compound 10

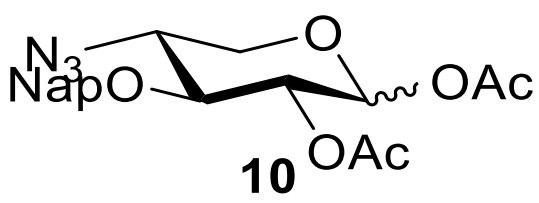

To a solution of compound **9** (0.28 g, 0.53 mmol) in a mixture of DCM/MeOH (1:1, v/v, 3 mL) was added 4.4 M NaOMe solution in MeOH (0.023 mL, 0.1 mmol). The reaction mixture was stirred overnight and was then quenched with $Et_3N{\cdot}HCl$ (250 mg, 0.16 mmol). The resulting mixture was then concentrated *in vacuo* and co-evaporated with dioxane 3 times. The crude was then re-dissolved in pyridine/Ac_2O (2:1, v/v, 3 mL) and stirred at rt for 16h. The reaction was quenched with water (0.5 mL) at 0 °C, diluted with EtOAc (10 mL), washed with 1M aq. HCl (3 x 3 mL), sat. aq. $NaHCO_3$ solution (3 x 3 mL) and brine, dried over $MgSO_4$, filtered and concentrated *in vacuo*. Subsequent flash column chromatography (pentane/EtOAc, 9:1) over silica afforded product **10** as a mixture of anomers (0.2 g, 90% over 2 steps). For major isomer: ^{1}H NMR (400 MHz, $CDCl_3$) δ 7.90 – 7.75 (m, 7H), 6.23 (d, J = 3.6 Hz, 1H), 5.03 – 4.88 (m, 3H), 4.14 – 4.05 (m, 1H), 3.90 – 3.80 (m, 2H), 3.74 – 3.66 (m, 1H), 3.57 (t, J = 11.1 Hz, 1H), 2.14 (s, 3H), 1.95 (s, 3H). ^{13}C NMR (101 MHz, $CDCl_3$) δ 170.21, 169.53, 135.51, 135.14, 128.76, 128.39, 128.18, 127.05, 126.73, 126.58, 126.11, 90.32, 78.61, 77.84, 75.82, 72.17, 62.51, 61.25, 21.37, 21.12. HRMS (ESI) m/z: $[M+Na]^+$ calculated for $C_{20}H_{21}N_3O_6Na$ 422.1323, found 422.1327.

Compound 11

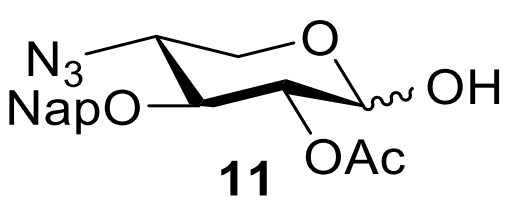

To a solution of compound **10** (0.2 g, 0.5 mmol) in THF (1 mL) was added piperidine (0.05 mL, 0.54 mmol). The mixture was stirred at rt overnight and then diluted with EtOAc (11 mL), washed with 1M aq. HCl (3 x 3 mL) and brine, dried over $MgSO_4$, filtered and concentrated *in vacuo*. The resulting mixture was purified by flash column chromatography (pentane/EtOAc, 3:1) over silica to give product **5** as a mixture of anomers (0.13 g, 74%). For major isomer **11**: ^{1}H NMR (500 MHz, $CDCl_3$): δ 7.88 – 7.81 (m, 4H), 7.53 – 7.47 (m, 3H), 5.39 (d, J = 3.5 Hz, 1H), 5.02 (t, J = 11.3

Hz, 1H), 4.96 – 4.82 (m, 3H), 3.97 (t, J = 9.2 Hz, 1H), 3.81 – 3.74 (m, 2H), 3.71 – 3.65 (m, 1H), 2.04 (s, 3H). ^{13}C NMR (126 MHz, $CDCl_3$): δ 170.38, 135.30, 133.37, 133.15, 128.34, 128.05, 127.78, 126.72, 126.28, 126.13, 125.83, 90.84, 77.79, 75.40, 73.51, 61.20, 60.07, 20.97. HRMS (ESI) m/z: $[M+Na]^+$ calculated for $C_{18}H_{19}N_3O_5Na$ 380.1217, found 380.1215.

Compound 12

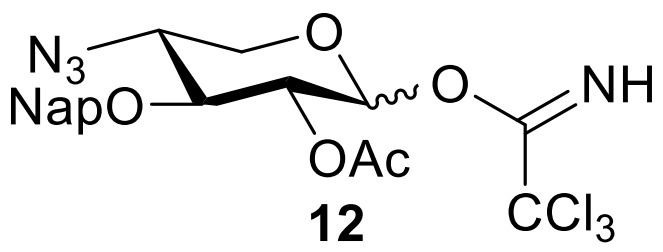

12

To a solution of compound **11** (0.13 g, 0.35 mmol) in DCM (2 mL) was added $CNCCl_3$ (70 µL, 0.7 mmol) and K_2CO_3 (97 mg, 0.7 mmol). The reaction mixture was stirred at rt overnight, after which the mixture was filtered over Celite®, concentrated *in vacuo* and purified by flash column chromatography (pentane/EtOAc, 5:1). The product **6** was obtained as a mixture of anomers (0.16 g, 88%). For main isomer **12**: ^{1}H NMR (400 MHz, $CDCl_3$): δ 8.71 (s, 1H), 7.89 – 7.85 (m, 4H), 7.55 – 7.50 (m, 3H), 5.89 (d, J = 5.8 Hz, 1H), 5.31 (dd, J = 7.4, 5.7 Hz, 1H), 4.95 (t, J = 1.2 Hz, 2H), 4.33 – 4.24 (m, 1H), 3.79 – 3.73 (m, 2H), 3.62 – 3.51 (m, 1H), 2.02 (s, 3H). ^{13}C NMR (101 MHz, $CDCl_3$): δ 161.04, 134.61, 128.33, 127.98, 127.72, 126.89, 126.28, 126.16, 125.79, 95.84, 77.89, 74.07, 69.40, 63.03, 59.01, 20.79.

Compound 14

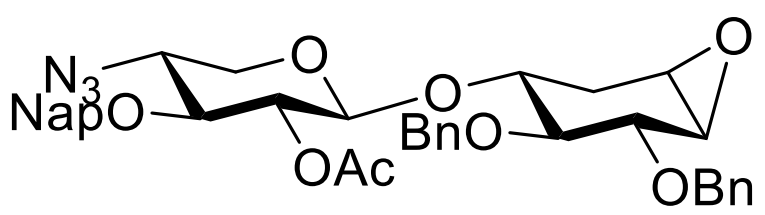

14

To a solution of compound **12** (0.16 g, 0.32 mmol) and acceptor **13** (75 mg, 0.23 mmol) (combined and co-evaporated with toluene 3 times beforehand) in dry DCM (2 mL) were added 3Å molecular sieves and the mixture was stirred for 1 hour. The mixture was then cooled to -30 °C before TMSOTf (11 µL, 0.06 mmol) was added. The reaction mixture was stirred for 1 hour at the same temperature and then quenched with Et_3N (15 µL), diluted with DCM (60 mL), washed with brine, dried over $MgSO_4$, filtered and concentrated *in vacuo*, purified by flash column chromatography (pentane/EtOAc, 6:1 to 4:1). The product **14** was obtained as a white solid (122 mg, 79%). ^{1}H NMR (400 MHz, $CDCl_3$): δ 7.90 – 7.81 (m, 4H), 7.55 – 7.47 (m, 3H), 7.44 – 7.29 (m, 12H), 5.06 – 4.95 (m, 3H), 4.87 (d, J = 11.5 Hz, 1H), 4.82 – 4.75 (m, 2H), 4.71 (d, J = 10.9 Hz, 1H), 4.48 (d, J = 6.7 Hz, 1H), 4.05 (dd, J = 12.0, 4.9 Hz, 1H), 3.86 (d, J = 7.8 Hz, 1H), 3.80 (dt, J = 10.3, 5.1 Hz, 1H), 3.69 – 3.63 (m, 1H), 3.59 (t, J = 8.3 Hz, 1H), 3.44 (dd, J = 10.1, 7.7 Hz, 1H), 3.33 – 3.26 (m, 1H), 3.22 – 3.08 (m, 2H), 2.55 (ddd, J = 14.5, 5.2, 2.4 Hz, 1H), 2.01 (s, 3H), 1.77 (ddd, J = 14.5, 10.5, 1.5 Hz, 1H). ^{13}C NMR (101 MHz, $CDCl_3$): δ 169.42, 138.81, 137.82, 134.88, 133.25, 133.11, 128.66, 128.60, 128.53, 128.35, 128.27, 128.21, 128.10, 128.04, 128.02, 127.93, 127.74, 127.46, 126.99, 126.27, 126.17,

126.16, 125.95, 99.38, 82.57, 79.97, 79.43, 75.28, 74.47, 73.68, 73.44, 72.55, 62.95, 60.37, 53.98, 53.40, 29.94, 20.97. HRMS (ESI) m/z: $[M+Na]^+$ calculated for $C_{38}H_{39}N_3O_8Na$ 688.2629, found 688.2625.

Compound 15

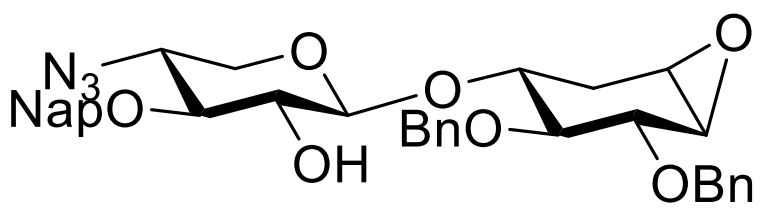

15

To a solution of compound **14** (109 mg, 0.165 mmol) in a mixture of DCM/MeOH (2 mL, 1/1) was added 4.4 M NaOMe (0.075 mL, 0.033 mmol) solution in MeOH. The mixture was stirred at rt for 5 hours and was then neutralized with $Et_3N{\cdot}HCl$ (5 mg, 35 μmol) and concentrated *in vacuo*. Flash column chromatography (pentane/EtOAc = 3/1) afforded product **15** (91 mg, 90%) as a white solid. ^{1}H NMR (400 MHz, $CDCl_3$) δ 7.82 (td, J = 5.1, 4.7, 2.2 Hz, 4H), 7.56 – 7.41 (m, 3H), 7.39 – 7.25 (m, 10H), 5.04 (d, J = 11.2 Hz, 1H, CH*H* Bn), 4.94 (d, J = 11.3 Hz, 1H, CH*H* Bn), 4.87 – 4.66 (m, 4H, CH_2 Bn, CH_2 Nap), 4.27 (d, J = 7.8 Hz, 1H, H1'), 3.98 – 3.78 (m, 3H, H5', H4, H2), 3.63 – 3.51 (m, 2H, H2', H4'), 3.50 – 3.37 (m, 2H, H3, H3'), 3.28 – 3.21 (m, 1H, H6), 3.12 (d, J = 3.6 Hz, 1H, H1), 3.05 (dd, J = 11.8, 10.8 Hz, 1H, H5'), 2.96 (d, 1H, OH), 2.59 (ddd, J = 14.5, 5.2, 2.4 Hz, 1H, H5a), 1.81 (ddd, J = 14.6, 10.5, 1.6 Hz, 1H, H5b). ^{13}C NMR (101 MHz, $CDCl_3$) δ 138.14, 137.61, 135.49, 133.35, 133.13, 128.62, 128.38, 128.33, 128.28, 128.06, 128.03, 127.83, 127.76, 127.10, 126.22, 126.13, 126.01, 101.62, 82.28, 82.01, 79.81, 74.94, 74.89, 73.31, 73.14, 70.84, 64.25, 60.74, 53.64, 53.58, 30.95. HRMS (ESI) m/z: $[M+Na]^+$ calculated for $C_{36}H_{37}N_3O_7Na$, 646.2529, found 646.2525.

Compound 17

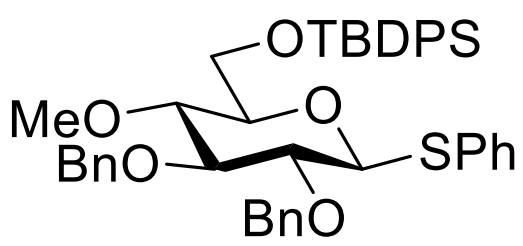

17

To a solution of compound **16**[5] (400 mg, 0.58 mmol) in dry THF (1.2 ml) was added MeI (0.22 ml, 3.5 mmol) and NaH (60% in mineral oil, 0.14 g, 3.5 mmol). The mixture was stirred for 2 hours, after which the reaction was quenched with methanol. To the mixture was then added water followed by extraction with Et_2O (3x). The combined organic layers were washed with brine, dried over Na_2SO_4 and concentrated *in vacuo*, purified by flash column chromatography (Et_2O/pentane, 0/1 to 1/9, v/v) to afford the product as a white solid. (410 mg, quant.) ^{1}H NMR (400 MHz, $CDCl_3$) δ 7.86 – 7.74 (m, 4H), 7.65 – 7.58 (m, 2H), 7.51 – 7.30 (m, 16H), 7.23 (dd, J = 5.4, 1.8 Hz, 3H), 4.93 – 4.85 (m, 3H), 4.76 (d, J = 10.3 Hz, 1H), 4.69 (d, J = 9.6 Hz, 1H), 3.97 (qd, J = 11.4, 2.7 Hz, 2H), 3.70 – 3.60 (m, 4H), 3.58 – 3.49 (m, 2H), 3.33 (ddd, J = 9.5, 3.5, 1.9 Hz, 1H), 1.12 (s, 9H). ^{13}C NMR (101 MHz, $CDCl_3$) δ 138.41, 138.24, 135.88, 135.64, 134.14, 133.61, 133.05, 131.69, 129.67, 129.61, 128.90, 128.50, 128.44, 128.18, 128.15, 127.84,

127.74, 127.66, 127.25, 87.39, 86.85, 80.53, 79.95, 79.12, 75.96, 75.41, 62.65, 60.77, 26.86, 19.38. HRMS (ESI) m/z: $[M+Na]^+$ calculated for $C_{43}H_{48}O_5SSiNa$ 727.2884, found 727.2878.

Compound 18

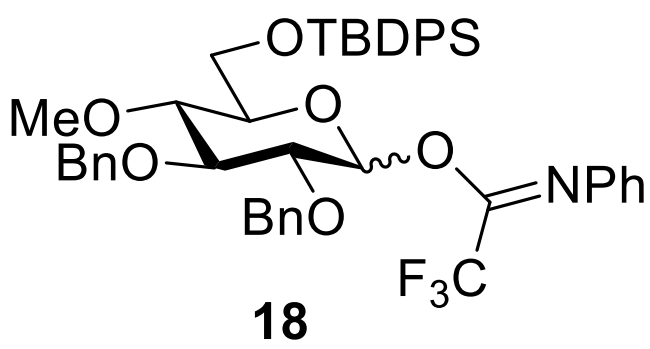

18

To a solution of compound **17** (246 mg, 0.35 mmol) in acetone:H_2O (10:1, 4 mL) was added N-iodosuccinimide (NIS) (6.2 g, 27.6 mmol). The reaction mixture was stirred at rt for 1 hour, after which the reaction mixture was diluted with DCM and quenched with saturated aqueous $Na_2S_2O_3$. The mixture was then washed with water and brine. The organic layer was dried with Na_2SO_4, filtered and concentrated *in vacuo*, purified by flash column chromatography (pentane : EtOAc = 5:1) to afford the lactol product (211 mg, quant.) as a white solid. To a solution of the lactol (210 mg, 0.34 mmol) in acetone (4 mL) was added Cs_2CO_3 (168 mg, 0.51 mmol) and 2,2,2-trifluoro-N-phenylacetimidoyl chloride (118.2 mg, 0.57 mmol) respectively. The reaction was allowed to stir at rt overnight, and subsequently quenched with Et_3N, filtered, concentrated. Flash column chromatography (pentane/EA, 40/1 to 20/1, v/v) afforded product **18** (208 mg, 78% over 2 steps) as a mixture of anomers. For major isomer: ^1H NMR (400 MHz, $CDCl_3$) δ 7.75 (dt, J = 8.0, 1.6 Hz, 4H), 7.52 – 7.26 (m, 18H), 7.17 – 7.10 (m, 1H), 6.77 (d, J = 7.7 Hz, 2H), 5.19 – 4.60 (m, 4H), 4.22 – 3.87 (m, 3H), 3.87 – 3.36 (m, 6H), 1.11 (s, 9H). ^{13}C NMR (101 MHz, $CDCl_3$) δ 143.78, 138.57, 137.99, 135.84, 135.65, 133.61, 133.19, 129.72, 129.67, 128.73, 128.52, 128.47, 128.21, 127.86, 127.80, 127.73, 127.63, 127.58, 124.15, 119.47, 81.53, 79.45, 78.44, 75.90, 74.31, 73.40, 62.35, 60.97, 26.86, 19.43. HRMS (ESI) m/z: $[M+Na]^+$ calculated for $C_{45}H_{48}F_3NO_6SSiNa$ 806.3101, found 806.3105.

Compound 19

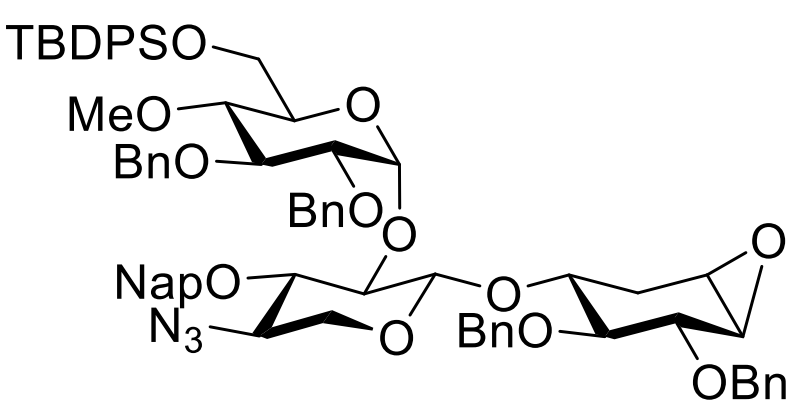

19

Compound **18** (60 mg, 0.11 mmol) and compound **12** (120 mg, 0.154 mmol) were co-evaporated with toluene for 3 times and dissolved in dry DCM (1 mL). The mixture was stirred over fresh flame-dried 3Å molecular sieves under argon, followed by the addition of DMF (0.19 mL, 2.46 mmol). The solution was then cooled to -78 °C, after which TfOH (23 mg, 0.154 mmol) was added. After 30 minutes, the reaction mixture was allowed to stir at 0 °C until TLC analysis showed complete conversion of compound **18**. The reaction was then quenched with Et_3N, filtered and concentrated *in vacuo*, purified by flash column chromatography (pentane : EtOAc

= 10:1 to 4:1) to afford the product **19** (88 mg, 67%) as a colorless oil. ^{1}H NMR (400 MHz, $CDCl_3$) δ 7.84 (s, 1H, ArH), 7.75 – 7.66 (m, 3H, ArH), 7.55 – 7.27 (m, 30H, ArH), 7.20 – 7.12 (m, 3H, ArH), 5.52 (d, J = 3.6 Hz, 1H, H1'', α-1,2), 4.96 – 4.87 (m, 6H, CH_2Bn, CH_2Nap), 4.76 – 4.66 (m, 4H, CH_2Bn), 4.48 (d, J = 7.2 Hz, 1H, H1', β-1,4), 4.09 – 3.94 (m, 3H, H5'b, H3'', H5''), 3.90 – 3.77 (m, 2H, H4, H2), 3.74 – 3.68 (m, 1H, H2'), 3.58 (m, 1H, H4'), 3.53 (m, J = 8.1, 5.0 Hz, 4H, H2'', H3', H6'a, H6'b), 3.46 (s, 3H, OMe), 3.44 – 3.40 (m, 1H, H4''), 3.34 (dd, J = 10.2, 7.5 Hz, 1H, H3), 3.16 – 3.07 (m, 1H, H5'a), 3.03 (d, J = 3.6 Hz, 1H, H1), 2.79 (d, J = 3.5 Hz, 1H, H6), 2.43 (ddd, J = 14.4, 5.1, 2.4 Hz, 1H, H5b), 1.47 – 1.38 (m, 1H, H5a), 0.93 (s, 9H, tBu). ^{13}C NMR (101 MHz, $CDCl_3$) δ 138.69, 138.54, 137.98, 135.71, 135.51, 134.57, 133.67, 133.44, 133.29, 133.12, 129.49, 129.42, 128.55, 128.52, 128.46, 128.34, 128.11, 128.02, 127.93, 127.90, 127.81, 127.78, 127.71, 127.62, 127.59, 127.45, 126.69, 125.93 (Ar), 100.38 (C1'), 96.25 (C1'', α-1,2), 82.38 (C3''), 81.74 (C3'), 81.43 (C3), 80.28 (C2''), 79.74 (C4''), 79.53 (C2), 76.17 (CH_2Ar), 75.77 (CH_2Ar), 75.02 (C2'), 74.75 (CH_2Ar), 74.28 (CH_2Ar), 73.34 (CH_2Ar), 71.66 (C4), 71.46 (C5''), 63.84 (C5'), 62.38 (C6'), 62.26 (C4'), 60.67 (OMe), 53.82 (C1), 53.31 (C6), 28.94 (C5), 26.86 (CH_3), 19.37 ($C(CH_3)_3$). HRMS (ESI) m/z: $[M+Na]^+$ calculated for $C_{73}H_{79}N_3O_{12}SiNa$ 1240.5331, found 1240.5330.

Compound S19

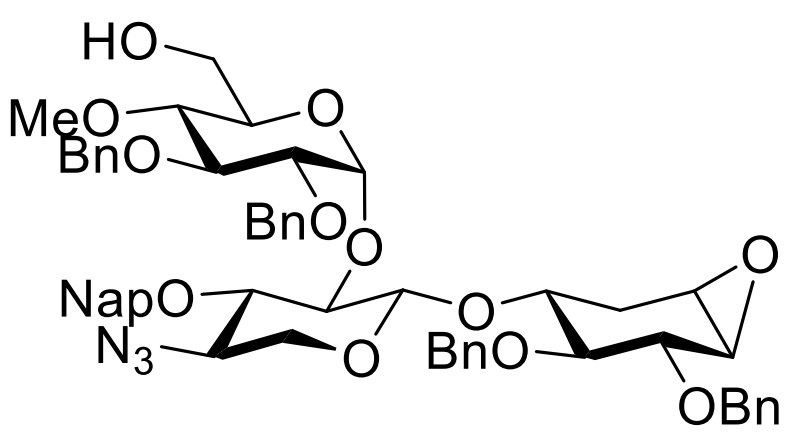

S19

To a solution of compound **19** (88 mg, 0.072 mmol) in dry THF (1 mL) was added TBAF solution (1 M in THF, 0.108 mL, 0.108 mmol). The reaction mixture was allowed to stir at reflux for 6 hours and then quenched with sat. aq. NH_4Cl at 0 °C. The mixture was then diluted with EtOAc, washed with water, and brine, dried over $MgSO_4$, filtered and concentrated under reduced pressure. Flash column chromatography (pentane/EtOAc = 1.5/1, v/v) afforded the product **S19** (64 mg, 90%) as an oil. ^{1}H NMR (500 MHz, $CDCl_3$) δ 7.89 (d, J = 1.6 Hz, 1H, ArH), 7.82 (td, J = 6.1, 3.1 Hz, 3H, ArH), 7.56 (dd, J = 8.4, 1.7 Hz, 1H, ArH), 7.50 – 7.43 (m, 2H, ArH), 7.41 – 7.25 (m, 20H, ArH), 5.44 (d, J = 3.6 Hz, 1H, H1'', α-1,2), 5.02 (d, J = 10.4 Hz, 1H, CH_2Bn), 4.97 – 4.79 (m, 5H, CH_2Bn, CH_2Nap), 4.76 – 4.64 (m, 4H, CH_2Bn), 4.50 (d, J = 7.2 Hz, 1H, H1', β-1,4), 4.06 – 3.92 (m, 3H, H5'b, H3'', H5''), 3.86 (td, J = 10.4, 5.2 Hz, 1H, H4), 3.78 (dd, J = 7.6, 0.8 Hz, 1H, H2), 3.67 (dd, J = 8.7, 7.2 Hz, 1H, H2'), 3.64 – 3.52 (m, 2H, H4', H3'), 3.49 (dd, J = 9.8, 3.6 Hz, 1H, H2''), 3.45 (s, 3H, OMe), 3.42 (d, J = 2.5 Hz, 1H, H6'a), 3.35 – 3.21 (m, 3H, H3, H6'b, H4''), 3.18 – 3.09 (m, 1H, H5'a), 3.04 (d, J = 3.6 Hz, 1H, H1), 2.84 (p, J = 1.9 Hz, 1H, H6), 2.44 (ddd, J = 14.5, 5.3, 2.5 Hz, 1H, H5a), 1.49 – 1.44 (m, 1H, H5b). ^{13}C

NMR (126 MHz, $CDCl_3$) δ 138.74, 138.72, 138.35, 137.94, 134.89, 134.55, 133.36, 133.22, 129.74, 128.58, 128.49, 128.31, 128.20, 128.16, 128.13, 128.08, 127.99, 127.97, 127.93, 127.91, 127.81, 127.78, 127.75, 127.65, 126.63, 126.11, 126.08 (Ar), 100.14, (C1'), 96.24(C1'', α-1,2), 82.22 (C3), 81.96 (C3''), 81.56 (C3'), 79.85 (C4''), 79.77 (C2''), 79.42 (C2), 76.17 (CH_2Ar), 75.55 (CH_2Ar), 75.38 (C2'), 74.93 (CH_2Ar), 74.14 (CH_2Ar), 73.41 (CH_2Ar), 71.33 (C4), 70.93 (C5''), 63.73 (C5'), 62.32 (C4'), 61.33 (C6'), 60.77 (OMe), 53.81 (C1), 53.25 (C6), 28.96 (C5). HRMS (ESI) m/z: $[M+Na]^+$ calculated for $C_{57}H_{61}N_3O_{12}Na$ 1002.4153, found 1002.4155.

Compound S20

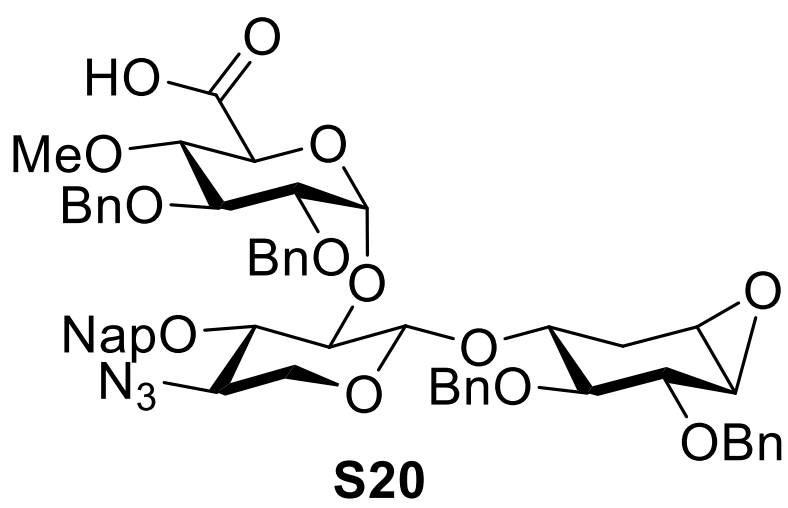

S20

Compound **S19** (53 mg, 0.054 mmol) was dissolved in a mixture of DCM/tBuOH/H_2O (4/4/1, v/v/v) (1 mL) and was cooled to 0 °C. To the solution was added TEMPO (1.7 mg, 0.011 mmol) and BAIB (14.8 mg, 0.135mmol) at the same temperature. The reaction mixture was stirred at 0 °C overnight and then quenched with aq. $Na_2S_2O_3$, diluted with water, and extracted with DCM (3 x 5 mL). The combined organic layers were washed with sat. aq. $NaHCO_3$ and brine, dried over MgSO4, filtrated and concentrated *in vacuo*. Flash column chromatography (DCM/MeOH = 20/1, v/v) afforded the product **S20** (42 mg, 79%) as an oil. ^1H NMR (500 MHz, $CDCl_3$) 7.96 – 7.88 (m, 1H, ArH), 7.85 (dd, J = 8.9, 4.6 Hz, 3H, ArH), 7.59 (dd, J = 8.4, 1.7 Hz, 1H, ArH), 7.52 – 7.44 (m, 2H, ArH), 7.38 – 7.25 (m, 20H, ArH), 5.51 (d, J = 3.4 Hz, 1H, H1'', α-1,2), 5.10 (d, J = 10.9 Hz, 1H, CH_2Bn), 5.00 (d, J = 10.9 Hz, 1H, CH_2Bn), 4.92 (d, J = 10.8 Hz, 1H, CH_2Bn), 4.89 – 4.79 (m, 3H, CH_2Bn), 4.74 (d, J = 0.9 Hz, 2H, CH_2Bn), 4.68 (dd, J = 12.7, 11.5 Hz, 2H, CH_2Bn), 4.62 (d, J = 10.2 Hz, 1H, H5''), 4.50 (d, J = 7.2 Hz, 1H, H1', β-1,4), 4.00 (dd, J = 11.9, 5.1 Hz, 1H, H5'a), 3.96 (dd, J = 9.8, 8.8 Hz, 1H, H3''), 3.84 (q, J = 5.2 Hz, 1H, H4), 3.81 (dd, J = 7.5, 0.8 Hz, 1H, H2), 3.72 (dd, J = 8.8, 7.2 Hz, 1H, H2'), 3.66 – 3.53 (m, 3H, H4', H3', H2''), 3.43 – 3.24 (m, 5H, OMe, H3, H4''), 3.15 (dd, J = 12.0, 9.5 Hz, 1H, H5'b), 3.06 (d, J = 3.6 Hz, 1H, H1), 2.90 – 2.79 (m, 1H, H6), 2.43 (ddd, J = 14.4, 5.2, 2.5 Hz, 1H, H5a), 1.50 – 1.44 (m, 1H, H5b). ^{13}C NMR (126 MHz, $CDCl_3$) δ 170.91 (C6'), 138.66, 138.19, 137.92, 137.88, 134.53, 133.38, 133.25, 128.63, 128.59, 128.57, 128.34, 128.32, 128.18, 128.08, 128.04, 127.99, 127.98, 127.96, 127.83, 127.78, 127.65, 127.60, 126.54, 126.15, 126.14 (Ar), 100.40 (C1'', β-1,4), 96.62 (α-1,2), 82.23 (C4''), 81.31 (C3), 81.27 (C3''), 81.23 (C3'), 79.50 (C2), 78.95 (C2''), 75.86 (CH_2Ar), 75.63 (CH_2Ar), 75.19 (C2'), 75.06, 74.20, 73.45, 72.02 (C4), 68.98 (C5''), 63.67 (C5'), 62.14 (C4'),

60.91 (OMe), 53.79 (C1), 53.24 (C6), 29.25 (C5). HRMS (ESI) m/z: [M+Na]$^+$ calculated for $C_{57}H_{59}N_3O_{13}Na$ 1016.3946, found 1016.3949.

Compound 20

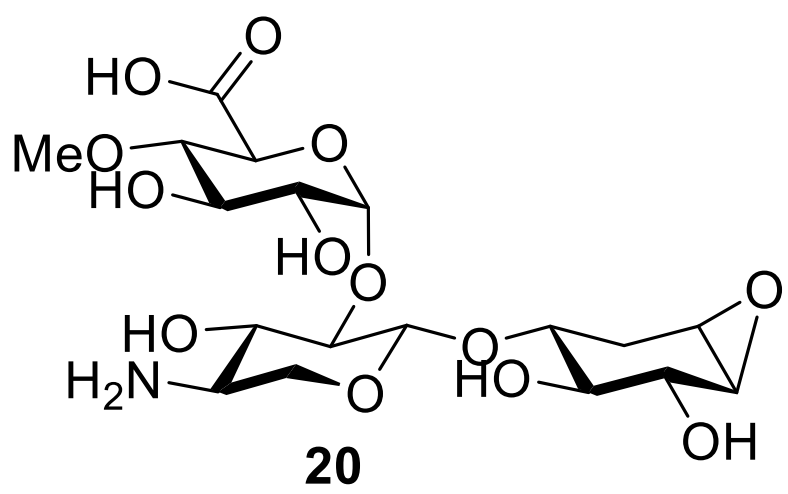

Compound **S20** (40 mg, 0.04 mmol) was dissolved in a mixture of dioxane (1 mL) and H_2O (0.25 mL). The solution was purged with nitrogen for 3 minutes and TFA (3.1 µL, 40 µmol) was added under nitrogen atmosphere. The mixture was stirred for 3 minutes and $Pd(OH)_2/C$ (20 wt%, 42 mg, 60 µmol) was added. While stirring vigorously, the flask was then purged with hydrogen for 5 minutes. The reaction mixture was stirred under hydrogen atmosphere and was monitored by LC-MS hourly. After 6 hours, LC-MS analysis indicated full conversion and the reaction mixture was the filtered over celite, concentrated *in vacuo*, purified by flash column chromatography (DCM/MeOH/ammonia water, 10/1/0.5, v/v/v) to give product **20** (14 mg, 78%) as an oil. ^{1}H NMR (500 MHz, D_2O) δ 5.27 (d, J = 3.9 Hz, 1H, H1'', α-1,2), 4.55 (d, J = 7.2 Hz, 1H, H1', β-1,4), 4.12 (d, J = 10.0 Hz, 1H, H5''), 4.05 (dd, J = 11.9, 4.9 Hz, 1H, H5'a), 3.70 (dd, J = 8.2, 0.8 Hz, 1H, H2), 3.67 – 3.59 (m, 2H, H3'', H3'), 3.58 – 3.48 (m, 2H, H4, H2''), 3.46 – 3.31 (m, 7H, H2', H5'b, H6, OMe, H3), 3.20 (td, J = 9.9, 4.9 Hz, 1H, H4'), 3.16 – 3.06 (m, 2H, H4'', H1), 2.62 (ddd, J = 14.9, 5.4, 2.4 Hz, 1H, H5a), 1.86 (ddd, J = 14.8, 10.4, 1.7 Hz, 1H, H5b). ^{13}C NMR (126 MHz, D_2O) δ 176.64 (C6'), 100.26 (C1', β-1,4), 97.51 (C1'', α-1,2), 82.49 (C4'), 75.91 (C2'), 74.51 (C3), 73.18 (C4), 72.35 (C5''), 72.29 (C3''), 71.15 (C2), 71.13 (C2''), 70.68 (C3'), 61.48 (C5'), 60.11 (OMe), 56.21 (C1), 54.82 (C6), 51.31 (C4'), 28.46 (C5). HRMS (ESI) m/z: [M+Na]$^+$ calculated for $C_{18}H_{19}NO_{13}Na$ 490.1537, found 490.1534.

Compound 6-Bio

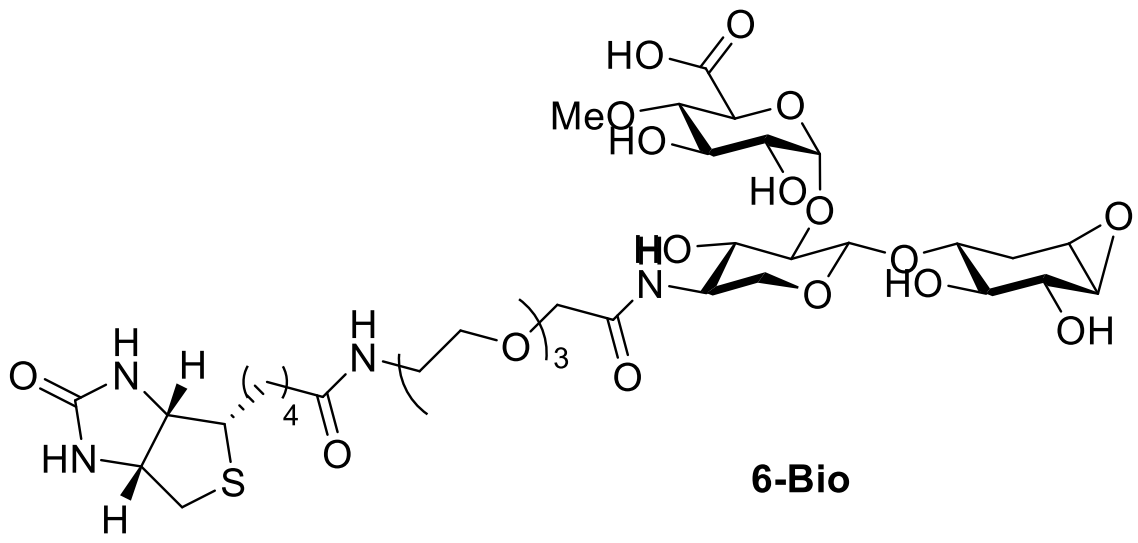

Amine **20** (5.8 mg, 0.012 mmol) was reacted with **28** according to general procedure A, affording product **6-Bio** (3.3 mg, 26%) as a white powder after lyophilization. ^{1}H NMR (500 MHz, D_2O) δ 5.30 (d, J = 3.9 Hz, 1H, H1'', α-1,2), 4.55 – 4.48 (m, 2H, NHCH&H1', β-1,4), 4.34 (dd, J = 8.0, 4.5 Hz, 1H), 4.24 (d, J = 10.1 Hz, 1H), 4.02 (s, 2H), 3.92 – 3.81 (m, 2H), 3.72 (d, J = 8.2 Hz, 1H), 3.68 – 3.57 (m, 9H), 3.55 (t, J = 5.3 Hz, 2H), 3.50 (dd, J = 9.9, 3.9 Hz, 1H),

3.40 (t, J = 4.2 Hz, 1H), 3.37 (d, J = 2.6 Hz, 3H), 3.34 – 3.29 (m, 2H), 3.28 – 3.21 (m, 2H), 3.18 – 3.09 (m, 2H), 2.91 (dd, J = 13.1, 5.0 Hz, 1H), 2.69 (d, J = 13.0 Hz, 1H), 2.65 (d, J = 13.8 Hz, 1H, H5a), 2.19 (t, J = 7.3 Hz, 2H), 1.87 (dd, J = 13.6, 10.6 Hz, 1H, H5b), 1.69 – 1.44 (m, 5H), 1.33 (q, J = 7.6 Hz, 2H), 1.26 (d, J = 6.9 Hz, 1H). ^{13}C NMR (126 MHz, D_2O) δ 179.66, 177.02 (C6'), 173.13, 168.01, 100.46 (C1', β-1,4), 97.66 (C1'', α-1,2), 82.52, 76.82, 75.03, 74.60, 73.25, 72.29, 71.68, 71.30, 71.12, 70.23, 69.71, 69.60, 69.55, 69.40, 68.95, 62.14, 60.30, 59.96, 56.15 (C1), 55.41, 54.84 (C6), 51.48, 39.74, 38.93, 35.51, 28.46 (C5), 27.91, 27.73, 25.17. HRMS (ESI) m/z: $[M+H]^+$ calculated for $C_{36}H_{59}N_4O_{19}S$ 883.3489, found 883.3486.

Compound 6-Cy5

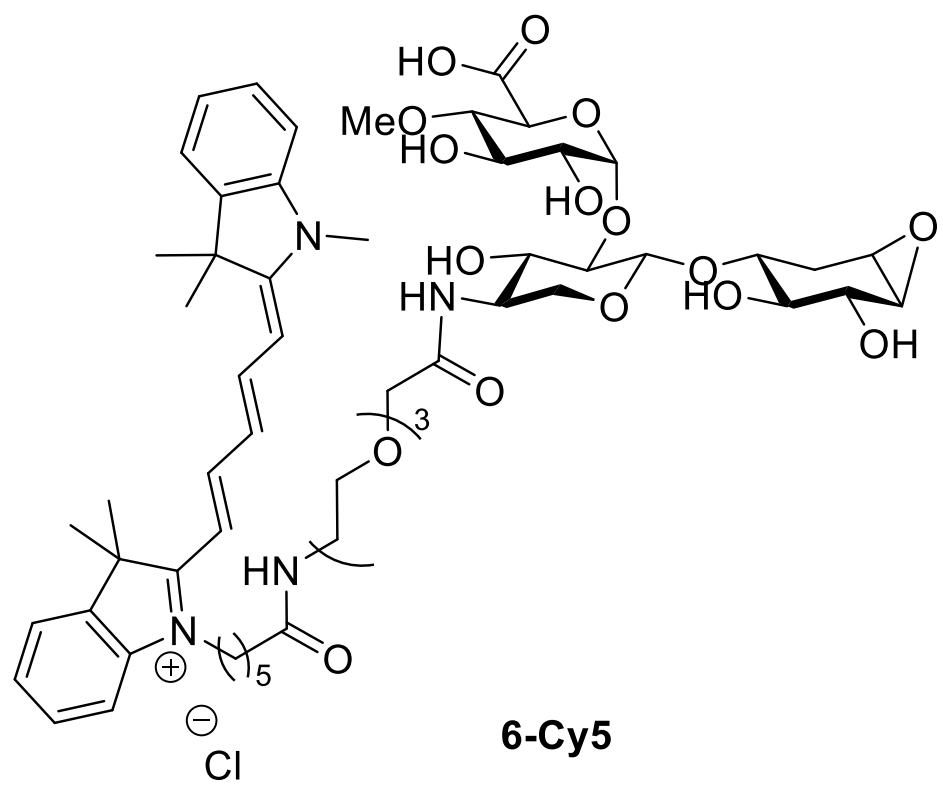

6-Cy5

Amine **20** (5.8 mg, 0.012 mmol) was reacted with **21** according to general procedure A, affording product **6-Cy5** (2.8 mg, 23%) as a blue powder after lyophilization. ^{1}H NMR (500 MHz, D_2O) δ 7.77 (s, 2H), 7.21 (m, 8H), 6.34 (s, 1H), 6.01 (d, J = 18.0 Hz, 2H), 5.26 (s, 1H, H1'', α-1,2), 4.44 (d, J = 7.5 Hz, 1H, H1', β-1,4), 4.17 (d, J = 10.1 Hz, 1H), 3.94 (d, J = 31.2 Hz, 3H), 3.79 (s, 1H), 3.66 (d, J = 7.5 Hz, 3H), 3.52 (d, J = 30.7 Hz, 10H), 3.38 (d, J = 18.7 Hz, 7H), 3.20 – 3.05 (m, 4H), 2.58 (s, 1H), 2.13 (s, 2H), 1.72 (s, 2H), 1.53 (s, 2H), 1.50 – 1.33 (m, 9H), 1.22 (t, J = 17.1 Hz, 3H). ^{13}C NMR (126 MHz, MeOD) δ 176.09, 173.07, 155.48, 155.45, 144.33, 143.58, 129.73, 126.24, 123.39, 121.41, 112.16, 111.86, 104.43, 101.90 (C1', β-1,4), 99.44 (C1'', α-1,2), 86.78, 77.52, 76.80, 74.65, 74.34, 71.81, 71.43, 71.23, 71.04, 70.51, 63.92, 54.83 (C1), 52.87 (C6), 52.81, 49.85, 47.94, 44.83, 40.31, 36.70, 34.72, 28.69 (C5), 27.97, 27.83. HRMS (ESI) m/z: $[M]^+$ calculated for $C_{58}H_{81}N_4O_{18}$ 1121.5540, found 1121.5538.

Compound S21

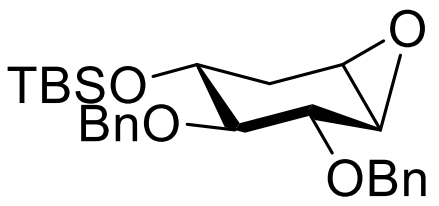

S21

Compound **13** (100 mg, 0.307 mmol) was dissolved in dry DCM (3 mL) and the solution was cooled to -78 °C. 2,6-lutidine (99 mg, 0.921 mmol) and TBSOTf (122 mg, 0.46 mmol) were added to the stirring solution. The reaction mixture was stirred at the same temperature for 30 minutes and was then diluted with DCM and washed with water. The organic layer was dried over $MgSO_4$, filtrated and concentrated *in vacuo*. Flash column chromatography (petane/EtOAc = 20/1, v/v) afforded product **S21** (128 mg, 95%) as a white solid. ^{1}H NMR (500 MHz, $CDCl_3$) δ 7.39 – 7.25 (m,

11H), 4.91 (d, J = 11.4 Hz, 1H), 4.83 – 4.70 (m, 3H), 3.82 (dd, J = 8.0, 0.8 Hz, 1H), 3.73 (td, J = 10.0, 5.3 Hz, 1H), 3.34 (dd, J = 9.8, 7.9 Hz, 1H), 3.29 – 3.24 (m, 1H), 3.17 (dd, J = 3.7, 0.7 Hz, 1H), 2.49 (dddd, J = 14.7, 5.3, 2.3, 0.8 Hz, 1H), 1.87 (ddd, J = 14.8, 10.2, 1.6 Hz, 1H), 0.93 (s, 9H), 0.11 (s, 3H), 0.06 (s, 3H). ^{13}C NMR (126 MHz, $CDCl_3$) δ 139.17, 137.98, 128.57, 128.25, 128.00, 127.92, 127.68, 127.35, 84.86, 79.97, 75.38, 73.33, 67.55, 54.19, 53.92, 34.00, 25.99, 18.14, -4.38, -4.69. HRMS (ESI) m/z: $[M+Na]^+$ calculated for $C_{26}H_{36}O_4SiNa$ 463.2281, found 463.2281.

Compound S22

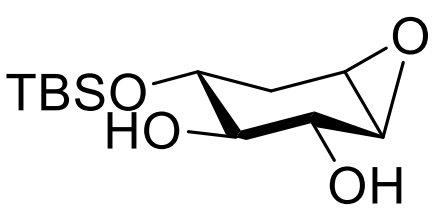

S22

Compound **S21** (106 mg, 0.24 mmol) was dissolved in a mixture of dioxane (1.5 mL), MeOH (1.5 mL) and H_2O (1.5 mL). The solution was purged with nitrogen for 3 minutes, after which $Pd(OH)_2/C$ (20 wt%, 101 mg, 145 μmol) was added. While stirring vigorously, the flask was then purged with hydrogen for 5 minutes. The reaction mixture was stirred under hydrogen atmosphere and was monitored by LC-MS. After 45 minutes, LC-MS analysis indicated full conversion and the reaction mixture was then filtered over celite, concentrated *in vacuo*, purified by flash column chromatography (DCM/MeOH, 25/1, v/v) to give product **S22** (63 mg, quant.) as a white solid. ^{1}H NMR (500 MHz, $CDCl_3$) δ 3.93 (dd, J = 7.0, 4.0 Hz, 1H, H2), 3.65 (td, J = 8.4, 5.1 Hz, 1H, H4), 3.53 (d, J = 5.1 Hz, 1H, OH), 3.40 (ddd, J = 8.4, 6.9, 3.6 Hz, 1H, H3), 3.27 (dtd, J = 3.6, 2.1, 1.7, 0.8 Hz, 1H, H6), 3.20 (d, J = 3.6 Hz, 1H, H1), 2.83 (d, J = 3.9 Hz, 1H, OH), 2.49 – 2.40 (m, 1H, H5a), 1.84 (ddd, J = 15.1, 8.0, 2.6 Hz, 1H, H5b), 0.89 (s, 9H), 0.10 (d, J = 1.4 Hz, 6H). ^{13}C NMR (126 MHz, $CDCl_3$) δ 75.96 (C3), 70.14 (C2), 67.70 (C4), 55.67 (C1), 52.87 (C6), 32.03 (C5), 25.82, 18.04, -4.40, -4.78. HRMS (ESI) m/z: $[M+Na]^+$ calculated for $C_{12}H_{24}O_4SiNa$ 283.1342, found 283.1343.

Compound 22

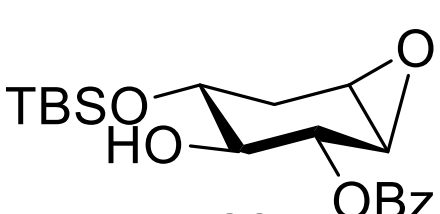

22

To a solution of compound **S22** (26 mg, 0.1 mmol) in pyridine (1 mL) was added benzoyl chloride (15 mg, 0.11 mmol) at 0 °C. The reaction mixture was stirred at the same temperature for 2 h, then stirred at rt overnight and then quenched with water and stirred for additional 15 min. The mixture was concentrated, taken up by DCM, washed with 1 M aq. HCl, sat. aq. $NaHCO_3$, brine and then concentrated *in vacuo*. Flash column chromatography (petane/EtOAc = 8/1 to 5/1, v/v) afforded product **22** (29 mg, 81%) ^{1}H NMR (500 MHz, $CDCl_3$) δ 8.17 – 8.07 (m, 2H, ArH), 7.63 – 7.57 (m, 1H, ArH), 7.50 – 7.43 (m, 2H, ArH), 5.33 – 5.22 (m, 1H, H2), 3.76 – 3.59 (m, 2H, H4, H3), 3.33 (m, 1H,

H6), 3.17 (d, J = 3.4 Hz, 1H, H1), 2.63 (d, J = 1.4 Hz, 1H, OH), 2.55 (dddd, J = 14.7, 5.0, 2.3, 0.7 Hz, 1H, H5a), 1.93 – 1.83 (m, 1H, H5b), 0.92 (s, 9H), 0.13 (d, J = 4.5 Hz, 6H). ^{13}C NMR (126 MHz, $CDCl_3$) δ 165.95, 133.39, 129.94, 129.80, 128.51, 74.92 (C3), 73.32 (C2), 67.51 (C4), 54.14 (C1), 53.70 (C6), 32.71 (C5), 25.81 (3xMe), 18.06 (CMe_3), -4.21 (SiMe), -4.70 (SiMe). HRMS (ESI) m/z: $[M+Na]^+$ calculated for $C_{19}H_{28}O_5SiNa$ 387.1604, found 387.1601.

Compound 24

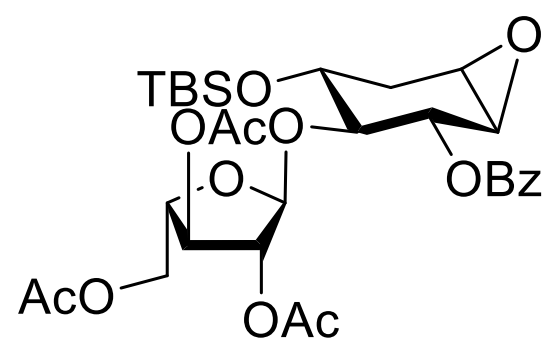

24

Acceptor **22** (75 mg, 0.2 mmol) and donor **23** (95 mg, 0.247 mmol) were co-evaporated with toluene for 3 times, re-dissolved in dry DCM (2 mL), and 3Å molecular sieves were added. The solution was stirred for 30 m9in and then cooled to -40 °C, after which NIS (56 mg, 0.247 mmol) and TMSOTf (4.6 mg, 20 μmol) were added to the stirring solution under nitrogen. The reaction mixture was stirred at the same temperature until TLC analysis indicated the full conversion of **22**. Et_3N (0.5 mL) was then added and the solution was diluted with EtOAc, washed with sat. aq. $NaHCO_3$, 10% aq. $Na_2S_2O_3$ and brine, dried over $MgSO_4$, filtered and concentrated under *in vacuo*. Flash column chromatography (EtOAc/pentane, 1/8 to 1/3, v/v) provided product **24** (87 mg, 70%). ^{1}H NMR (500 MHz, $CDCl_3$) δ 8.14 – 8.10 (m, 2H), 7.63 – 7.59 (m, 1H), 7.50 – 7.46 (m, 2H), 5.35 – 5.28 (m, 2H, H2, H1', α-1,3), 5.10 (dd, J = 1.8, 0.6 Hz, 1H, H2'), 4.88 (ddt, J = 5.2, 1.9, 1.0 Hz, 1H, H3'), 4.36 – 4.31 (m, 2H, H5'a, H4'), 4.18 – 4.14 (m, 1H, H5'b), 3.86 – 3.79 (m, 2H, H3, H4), 3.31 – 3.26 (m, 1H, H6), 3.10 (d, J = 3.4 Hz, 1H, H1), 2.58 – 2.49 (m, 1H, H5a), 2.06 (s, 3H), 1.99 – 1.93 (m, 1H, H5b), 1.85 (d, J = 5.6 Hz, 6H), 0.91 (s, 9H), 0.12 (d, J = 4.5 Hz, 6H). ^{13}C NMR (126 MHz, $CDCl_3$) δ 170.65, 170.17, 169.68, 165.34, 133.55, 129.95, 129.52, 128.68, 106.51 (C1', α-1,3), 81.49 (C2'), 80.70 (C4'), 78.70 (C3), 77.03 (C3'), 73.87 (C2), 66.26 (C4), 63.26 (C5'), 53.94 (C1), 53.40 (C6), 33.51 (C5), 25.95, 20.81, 20.50, 20.45, 18.14, -4.46, -4.83. HRMS (ESI) m/z: $[M+Na]^+$ calculated for $C_{30}H_{42}O_{12}SiNa$ 645.2343, found 645.2344.

Compound 25

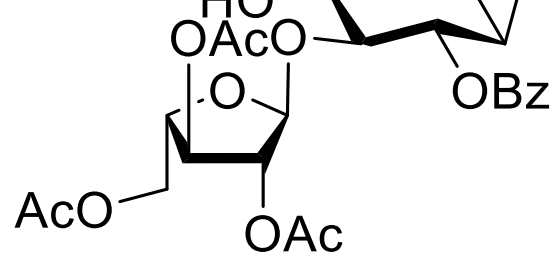

25

To a solution of compound **25** (87 mg, 0.14 mmol) in dry THF (1.5 mL) was added TBAF solution (1 M in THF, 0.21 mL, 0.21 mmol) and acetic acid (12.6 mg, 0.21 mmol). The reaction mixture was stirred at rt overnight and then quenched with sat. aq. NH_4Cl at 0 °C. The mixture was then diluted with EtOAc, washed with water, and brine, dried over $MgSO_4$, filtered and concentrated under reduced pressure. Flash column chromatography

(pentane/EtOAc = 2/1 to 1/1, v/v) afforded the product **25** (56 mg, 79%%) as an oil. ^{1}H NMR (500 MHz, $CDCl_3$) δ 8.13 – 8.08 (m, 2H), 7.66 – 7.59 (m, 1H), 7.53 – 7.48 (m, 2H), 5.30 (dd, *J* = 8.3, 0.8 Hz, 1H), 5.27 (s, 1H), 5.06 (dd, *J* = 1.9, 0.9 Hz, 1H), 5.00 (dd, *J* = 4.3, 1.9 Hz, 1H), 4.40 – 4.31 (m, 2H), 4.24 – 4.17 (m, 1H), 3.82 (dd, *J* = 9.9, 8.2 Hz, 1H), 3.75 (td, *J* = 10.1, 5.3 Hz, 1H), 3.35 (dd, *J* = 3.6, 2.1 Hz, 1H), 3.17 – 3.05 (m, 2H), 2.78 – 2.66 (m, 1H), 2.08 (d, *J* = 10.1 Hz, 6H), 1.97 – 1.89 (m, 1H), 1.85 (s, 3H). ^{13}C NMR (126 MHz, $CDCl_3$) δ 170.65, 169.88, 169.28, 165.47, 133.60, 129.94, 129.49, 128.68, 106.07, 81.46, 80.69, 80.67, 76.59, 73.48, 64.59, 63.23, 54.11, 53.42, 31.22, 20.85, 20.74, 20.43. HRMS (ESI) m/z: $[M+Na]^+$ calculated for $C_{24}H_{28}O_{12}Na$ 531.1479, found 531.1475.

Compound 26

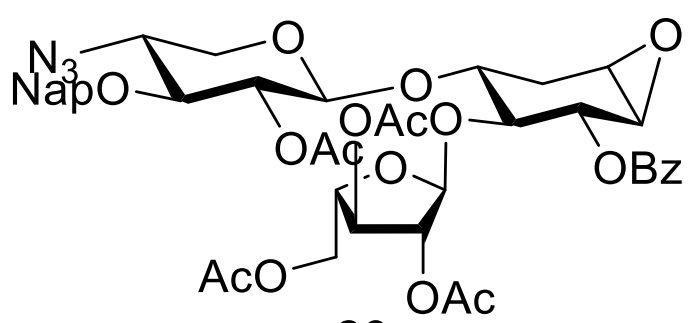

26

To a solution of compound **12** (0.029 g, 0.058 mmol) and acceptor **25** (21 mg, 0.04 mmol) (combined and co-evaporated with toluene 3 times beforehand) in dry DCM (0.5 mL) were added 3Å molecular sieves and the mixture was stirred for 1 hour. The mixture was then cooled to -30 °C before TMSOTf (1.8 mg, 0.008 mmol) was added. The reaction mixture was stirred for 1 hour at the same temperature and then quenched with Et_3N (5 µL), diluted with DCM (8 mL), washed with brine, dried over $MgSO_4$, filtered and concentrated *in vacuo*, purified by flash column chromatography (pentane/EtOAc, 2:1 to 1:1). The product **26** was obtained as a white solid (29 mg, 86%). ^{1}H NMR (400 MHz, $CDCl_3$): δ 7.90 – 7.81 (m, 4H), 7.55 – 7.47 (m, 3H), 7.44 – 7.29 (m, 12H), 5.06 – 4.95 (m, 3H), 4.87 (d, J = 11.5 Hz, 1H), 4.82 – 4.75 (m, 2H), 4.71 (d, J = 10.9 Hz, 1H), 4.48 (d, J = 6.7 Hz, 1H), 4.05 (dd, J = 12.0, 4.9 Hz, 1H), 3.86 (d, J = 7.8 Hz, 1H), 3.80 (dt, J = 10.3, 5.1 Hz, 1H), 3.69 – 3.63 (m, 1H), 3.59 (t, J = 8.3 Hz, 1H), 3.44 (dd, J = 10.1, 7.7 Hz, 1H), 3.33 – 3.26 (m, 1H), 3.22 – 3.08 (m, 2H), 2.55 (ddd, J = 14.5, 5.2, 2.4 Hz, 1H), 2.01 (s, 3H), 1.77 (ddd, J = 14.5, 10.5, 1.5 Hz, 1H). ^{13}C NMR (126 MHz, $CDCl_3$) δ 170.82, 170.19, 169.82, 169.19, 165.33, 134.93, 133.59, 133.30, 133.17, 129.95, 129.45, 128.68, 128.39, 128.05, 127.78, 126.94, 126.33, 126.21, 125.91, 81.37, 80.69, 80.32, 75.58, 74.62, 73.63, 72.90, 63.46, 63.21, 60.54, 53.57, 52.97, 29.36, 20.94, 20.89, 20.57, 20.45. HRMS (ESI) m/z: $[M+Na]^+$ calculated for $C_{42}H_{45}N_3O_{16}Na$ 870.2698, found 870.2696.

Compound 27

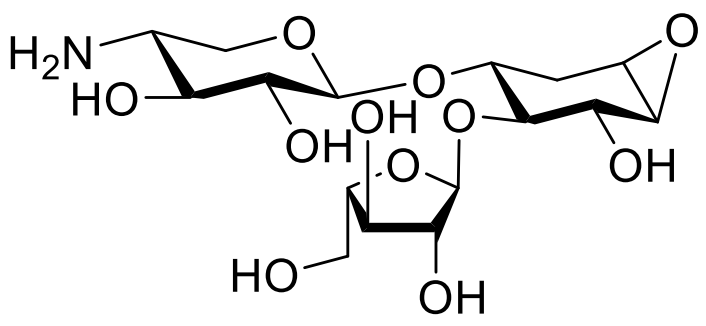

27

To a solution of compound **26** (25 mg, 0.029 mmol) in MeOH (1 mL) was added 4.4 M NaOMe (2 µL, 0.009 mmol) solution in MeOH. The mixture was stirred at rt for overnight and was then neutralized with $Et_3N·HCl$ (1.2 mg, 9 µmol) and concentrated *in vacuo*. The crude was preliminarily purified by flash column chromatography (DCM/MeOH = 12/1) and was then re-dissolved in a mixture of dioxane (1 mL) and H_2O (0.5 mL). The solution was purged with nitrogen for 3 minutes and TFA (2.2 µL, 29 µmol) was added under nitrogen atmosphere. The mixture was stirred for 3 minutes and $Pd(OH)_2/C$ (20 wt%, 30 mg, 44 µmol) was added. While stirring vigorously, the flask was then purged with hydrogen for 5 minutes. The reaction mixture was stirred under hydrogen atmosphere and was monitored by LC-MS hourly. After 4 hours, LC-MS analysis indicated full conversion and the reaction mixture was then filtrated over celite and concentrated *in vacuo* to give product **27** (9.6 mg, 81%) as an oil. ^{1}H NMR (500 MHz, D_2O) δ 5.32 (d, *J* = 0.9 Hz, 1H, H1'', α-1,3), 4.37 (d, *J* = 7.7 Hz, 1H, H1', β-1,4), 4.12 (ddd, *J* = 5.9, 4.8, 3.9 Hz, 1H, H4''), 4.06 – 3.96 (m, 2H, H2'', H5'a), 3.91 (dd, *J* = 7.6, 0.7 Hz, 1H, H2), 3.79 (ddd, *J* = 4.8, 2.2, 0.7 Hz, 1H, H3''), 3.68 (dd, *J* = 12.1, 3.9 Hz, 1H, H5''a), 3.64 – 3.57 (m, 2H, H5''b, H4), 3.56 – 3.50 (m, 2H, H3', H3), 3.45 – 3.37 (m, 2H, H5'b, H6), 3.23 (dd, *J* = 9.0, 7.7 Hz, 1H, H2'), 3.17 (ddd, *J* = 10.8, 10.0, 4.9 Hz, 1H, H4'), 3.11 (d, *J* = 3.7 Hz, 1H, H1), 2.64 – 2.50 (m, 1H, H5a), 1.89 – 1.83 (m, 1H, H5b). ^{13}C NMR (126 MHz, D_2O) δ 107.14 (C1'', α-1,3), 100.69 (C1', β-1,4), 85.01 (C4''), 80.41 (C2''), 78.23 (C3), 77.22 (C3''), 72.99 (C2'), 71.87 (C3'), 71.70 (C2), 71.46 (C4), 61.63 (C5'), 61.43 (C5''), 56.44 (C1), 54.57 (C6), 51.12 (C4'), 28.95 (C5). HRMS (ESI) m/z: $[M+Na]^+$ calculated for $C_{16}H_{27}O_{11}NNa$ 432.1482, found 432.1480.

Compound 4-Bio

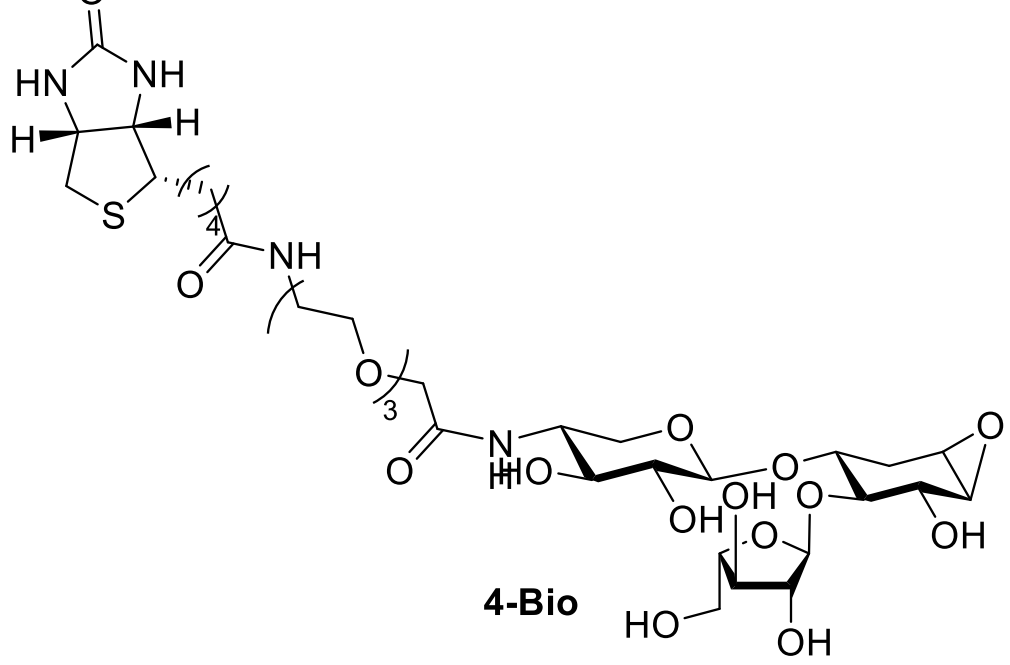

4-Bio

Amine **27** (5 mg, 0.012 mmol) was reacted with **28** according to general procedure A, affording product **4-Bio** (3 mg, 30%) as a white powder after lyophilization. ^{1}H NMR (400 MHz, MeOD) δ 5.48 (s, 1H), 4.58 (s, 1H), 4.55 – 4.49 (m, 1H), 4.43 – 4.21 (m, 4H), 3.99 – 3.90 (m, 6H), 3.83 (d, J = 12.0 Hz, 3H), 3.79 – 3.65 (m, 15H), 3.58 (q, J = 5.1, 4.3 Hz, 4H), 3.47 – 3.38 (m, 4H), 3.28 – 3.20 (m, 1H), 2.96 (dd, J = 12.7, 4.9 Hz, 1H), 2.73 (d, J = 12.9 Hz, 1H), 2.34 – 2.21 (m, 3H), 2.07 (d, J = 13.9 Hz, 1H), 1.98 – 1.86 (m, 2H), 1.69 (dddt,

J = 33.1, 20.8, 12.8, 6.7 Hz, 6H), 1.48 (q, J = 7.4 Hz, 3H), 1.31 (s, 8H), 1.00 – 0.83 (m, 3H). ^{13}C NMR (126 MHz, D_2O) δ 174.83, 171.74, 107.90, 100.75, 86.14, 80.06, 78.02, 75.75, 73.74, 73.50, 73.19, 72.93, 72.26, 70.43, 70.08, 70.02, 69.83, 69.74, 69.22, 68.36, 63.02, 62.05, 61.99, 60.24, 57.97, 57.00, 55.59, 53.98, 50.77, 39.63, 38.95, 35.35, 30.94, 29.33, 28.36, 28.11, 25.42. HRMS (ESI) m/z: $[M+Na]^+$ calculated for $[M+H]^+$ calculated for $C_{34}H_{57}N_4O_{17}S$ 825.3434, found 825.3430.

Compound 4-Cy5

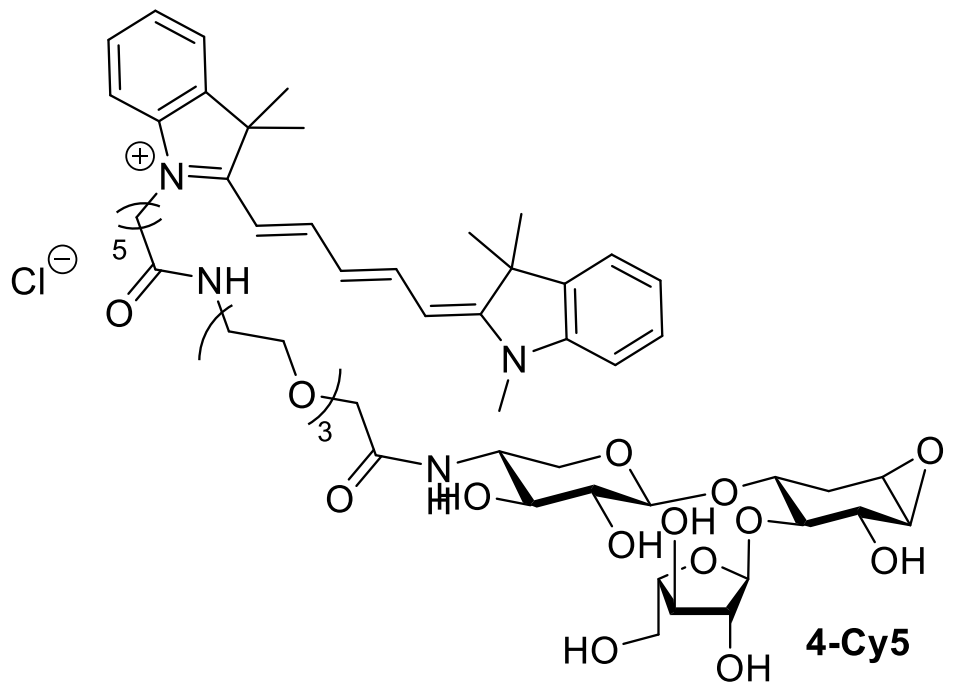

4-Cy5

Amine **27** (5 mg, 0.012 mmol) was reacted with **21** according to general procedure A, affording product **4-Cy5** (2.5 mg, 19%) as a blue powder after lyophilization. ^{1}H NMR (500 MHz, MeOD) δ 8.21 (t, J = 13.2 Hz, 2H), 7.45 (d, J = 7.5 Hz, 2H), 7.37 (d, J = 7.8 Hz, 2H), 7.24 (dt, J = 14.9, 7.7 Hz, 5H), 6.59 (t, J = 12.4 Hz, 1H), 6.25 (dd, J = 13.7, 4.1 Hz, 2H), 5.21 (s, 1H, H1'', α-1,3), 4.52 (d, J = 7.9 Hz, 1H, H1', β-1,4), 2.96 (d, J = 3.6 Hz, 1H), 2.70 – 2.44 (m, 1H), 2.19 (t, J = 7.4 Hz, 3H), 2.01 (dd, J = 32.2, 8.8 Hz, 2H), 1.90 (s, 3H), 1.79 (p, J = 7.4, 6.6 Hz, 4H), 1.43 (p, J = 7.5, 7.0 Hz, 3H), 1.35 – 1.14 (m, 5H), 0.92 – 0.77 (m, 1H). ^{13}C NMR (126 MHz, MeOD) δ 173.26, 171.68, 161.61, 154.12, 142.86, 142.17, 141.13, 128.33, 128.04, 127.06, 124.86, 122.02, 110.68, 110.44, 109.25, 108.90 (C1'', α-1,3), 102.90, 101.73 (C1', β-1,4), 84.72, 83.83, 81.73, 77.79, 77.25, 75.40, 73.93, 73.64, 70.44, 70.11, 69.81, 69.20, 66.73, 64.14, 63.21, 61.53, 55.58 (C1), 54.02 (C6), 53.39, 49.28, 48.99, 45.64, 43.78, 43.38, 38.92, 35.25, 29.44 (C5), 27.36, 26.80, 26.56, 26.41, 25.12, 24.50. HRMS (ESI) m/z: $[M]^+$ calculated for $C_{56}H_{79}N_4O_{16}$ 1063.5486, found 1063.5489.

Compound S8

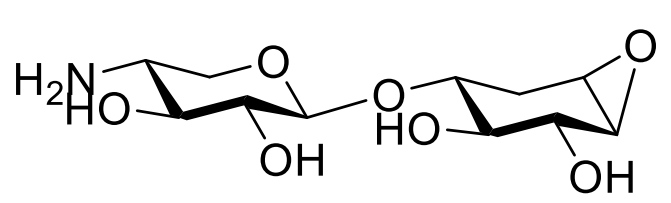

S8

Compound **15** (25 mg, 0.04 mmol) was dissolved in a mixture of dioxane (1 mL) and H_2O (0.25 mL). The solution was purged with nitrogen for 3 minutes and TFA (3.1 µL, 40 µmol) was added under nitrogen atmosphere. The mixture was stirred for 3 minutes and $Pd(OH)_2$/C (20 wt%, 42 mg, 60 µmol) was added. While stirring vigorously, the flask was then purged with hydrogen for 5 minutes. The reaction mixture was stirred under hydrogen atmosphere and was monitored by LC-MS hourly. After 4 hours, LC-MS analysis indicated full conversion and the reaction mixture was then filtrated over celite and concentrated *in vacuo* to give product **S8** (11 mg,

quant.) as an oil. ^{1}H NMR (500 MHz, D_2O) δ 4.33 (d, *J* = 7.7 Hz, 1H, H1', β-1,4), 3.84 (dd, *J* = 11.8, 5.2 Hz, 1H, H5'a), 3.73 (dd, *J* = 8.2, 0.8 Hz, 1H, H2), 3.55 (td, *J* = 10.3, 5.2 Hz, 1H, H4), 3.40 (dt, *J* = 3.8, 2.0 Hz, 1H, H6), 3.33 (dd, *J* = 10.2, 8.2 Hz, 1H, H3), 3.24 (t, *J* = 9.4 Hz, 1H, H3'), 3.20 – 3.13 (m, 2H, H2', H5'b), 3.12 (d, *J* = 3.8, 1H, H1), 2.77 (ddd, *J* = 10.8, 9.5, 5.2 Hz, 1H, H4'), 2.65 – 2.51 (m, 1H, H5a), 1.80 (ddd, *J* = 14.9, 10.4, 1.7 Hz, 1H, H5b). ^{13}C NMR (126 MHz, D_2O) δ 101.17 (C1', β-1,4), 75.86 (C3'), 74.54 (C3), 73.73 (C4), 73.20 (C2'), 71.24 (C2), 65.67 (C5'), 56.24 (C1), 54.81 (C6), 51.85 (C4'), 28.87 (C5). HRMS (ESI) m/z: $[M+Na]^+$ calculated for $C_{11}H_{19}NO_7Na$ 300.1059, found 300.1062.

Compound 1-Bio

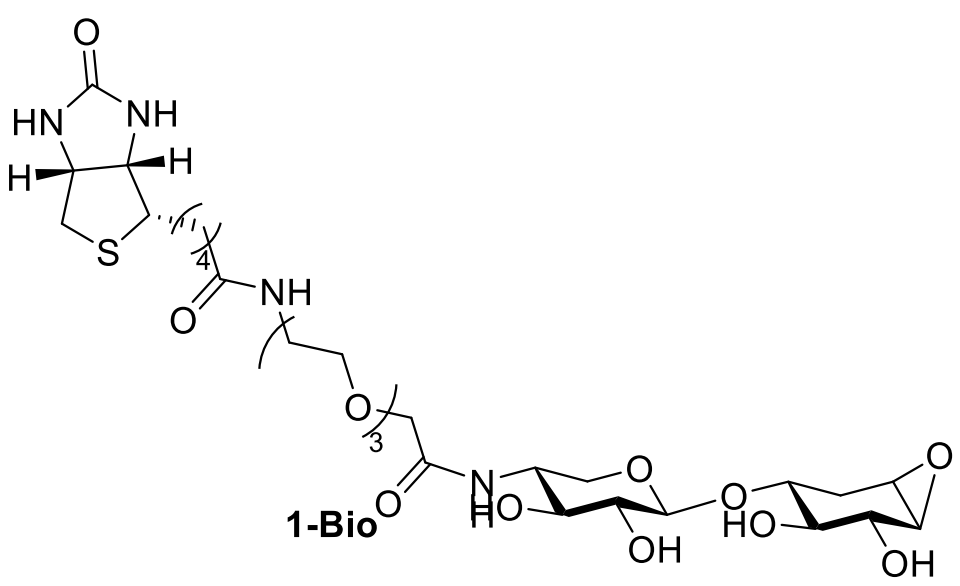

1-Bio

Amine **S8** (5 mg, 0.012 mmol) was reacted with **28** according to general procedure A, affording product **1-Bio** (2.5 mg, 30%) as a white powder after lyophilization. ^{1}H NMR (500 MHz, D_2O) δ 4.60 (dd, J = 8.3, 5.0 Hz, 1H, H1', β-1,4), 4.46 – 4.37 (m, 2H), 3.93 (dd, J = 11.7, 5.4 Hz, 1H), 3.68 (d, J = 8.4 Hz, 1H), 3.63 – 3.50 (m, 2H), 3.42 (t, J = 9.4 Hz, 1H), 3.34 – 3.11 (m, 6H), 2.99 (dd, J = 12.9, 5.1 Hz, 1H), 2.78 (d, J = 13.1 Hz, 1H), 2.52 (dd, J = 13.9, 5.4 Hz, 1H), 2.36 – 2.17 (m, 4H), 2.05 (s, 3H), 1.90 (s, 3H), 1.78 – 1.18 (m, 19H). ^{13}C NMR (126 MHz, MeOD) δ 174.80, 171.82, 101.62, 101.53 (C1', β-1,4), 77.25, 75.39, 73.80, 73.29, 72.98, 72.70, 72.56, 71.74, 71.53, 70.55, 70.45, 70.13, 70.09, 70.04, 69.83, 69.80, 69.75, 69.71, 69.22, 68.61, 63.00, 61.99, 61.97, 60.23, 57.97, 56.98 (C1), 55.59 (C6), 53.99, 53.39, 50.79 (C4'), 39.63, 38.92, 35.35, 35.33, 31.26, 29.37 (C5), 28.36, 28.09, 26.74, 25.43, 25.23, 25.04. HRMS (ESI) m/z: $[M+Na]^+$ calculated for $C_{29}H_{49}N_4O_{13}S$ 693.3011, found 693.3008.

Compound 1-Cy5

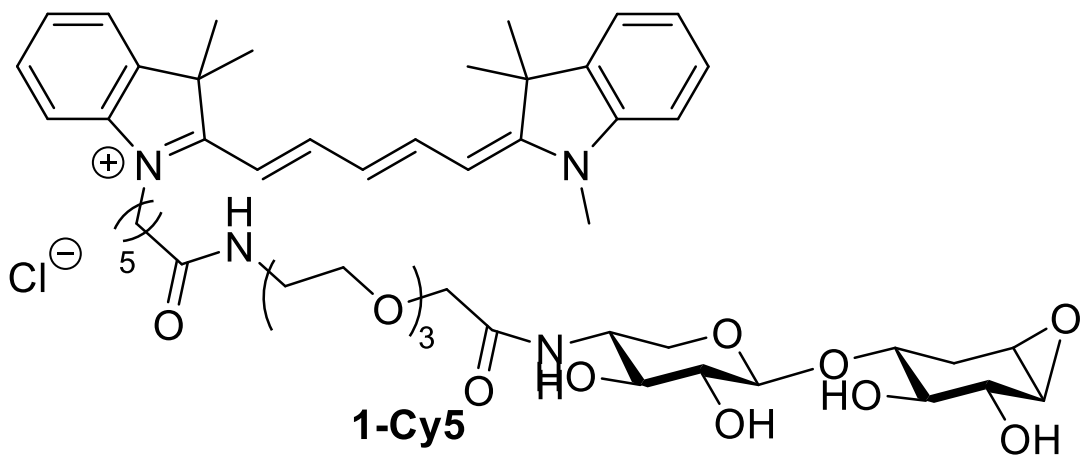

1-Cy5

Amine **S8** (5 mg, 0.012 mmol) was reacted with **21** according to general procedure A, affording product **1-Cy5** (3.8 mg, 33%) as a blue powder after lyophilization. ^{1}H NMR (500 MHz, D_2O) δ 7.77 (t, J = 12.7 Hz, 2H), 7.35 (dd, J = 7.2, 2.0 Hz, 2H), 7.25 (q, J = 7.5 Hz, 2H), 7.19 – 7.03 (m, 4H), 6.33 (t, J = 12.5 Hz, 1H), 6.02 (t, J = 13.0 Hz, 2H), 4.25 (d, J = 7.8 Hz, 1H, H1', β-1,4), 3.96 (s, 2H), 3.80 (dd, J = 11.4, 5.4 Hz, 1H), 3.57 – 3.43 (m, 5H), 3.39 (d, J = 5.2 Hz, 1H), 3.30 (t, J = 9.2 Hz, 1H), 3.20 – 3.07 (m, 3H), 2.91 (t,

J = 7.0 Hz, 2H), 2.31 (dd, J = 13.8, 5.4 Hz, 1H), 2.09 (t, J = 6.7 Hz, 2H), 2.03 – 1.87 (m, 2H), 1.81 (s, 6H), 1.70 (q, J = 7.4 Hz, 3H), 1.61 – 1.48 (m, 3H), 1.43 (d, J = 6.6 Hz, 13H), 1.22 (d, J = 6.7 Hz, 6H), 1.03 (d, J = 16.9 Hz, 8H). ^{13}C NMR (126 MHz, D_2O) δ 176.05, 173.35, 162.69, 153.14, 146.49, 142.78, 142.07, 141.14, 128.52, 125.11, 122.39, 112.21, 111.04, 100.94 (C1', β-1,4), 75.73, 75.15, 75.10, 72.77, 72.33, 69.23, 65.20, 59.63, 56.90 (C1), 54.95 (C6), 49.06, 43.63, 43.05, 39.68, 39.38, 35.53, 30.79, 28.98 (C5), 28.86, 28.60, 28.45, 28.36, 27.00, 26.88, 26.60, 26.23, 25.43, 25.06, 23.30. HRMS (ESI) m/z: $[M]^+$ calculated for $C_{51}H_{71}N_4O_{12}$ 931.5063, found 931.5059.

Compound S9

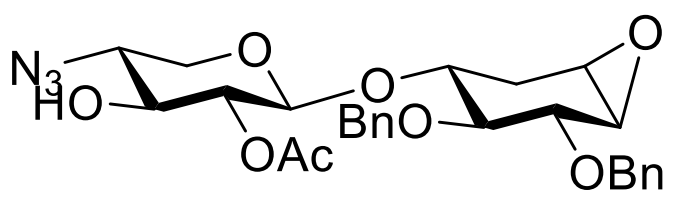

S9

Compound **14** (166 mg, 0.25 mmol) was dissolved in a mixture of DCM/H_2O (19/1, v/v, 5 mL). DDQ (62.4 mg, 0.275 mmol) was added to the mixture. The reaction was stirred until TLC-analysis indicated full consumption of **14**. The mixture was then diluted with DCM, washed with sat. aq. $NaHCO_3$ and brine, dried over Na_2SO_4, filtrated and concentrated under reduced pressure. Flash column chromatography of the crude afforded product **S9** (103 mg, 79%) as a white solid. ^{1}H NMR (400 MHz, $CDCl_3$): δ 7.40 – 7.29 (m, 10H), 4.86 – 4.71 (m, 5H), 4.60 (d, J = 5.6 Hz, 1H, H1', β-1,4), 4.06 (dd, J = 12.4, 4.4 Hz, 1H), 3.88 (dd, J = 7.5, 0.8 Hz, 1H), 3.81 (td, J = 10.3, 5.2 Hz, 1H), 3.71 (q, J = 7.1 Hz, 1H), 3.50 – 3.41 (m, 2H), 3.32 (dt, J = 3.5, 2.4 Hz, 1H, H6), 3.25 – 3.10 (m, 3H), 2.58 (ddd, J = 14.1, 5.3, 2.4 Hz, 1H, H5a), 2.18 (s, 3H, OAc), 1.80 (ddd, J = 14.5, 10.3, 1.6 Hz, 1H, H5b). ^{13}C NMR (101 MHz, $CDCl_3$): δ 170.67 (C=O), 138.45, 137.61, 128.62, 128.32, 128.20, 128.06, 128.03, 127.69, 97.88 (C1', β-1,4), 82.03, 79.64, 75.05, 73.25, 72.59, 72.50, 71.98, 61.52, 60.13, 53.72 (C1), 53.30 (C6), 29.54, 20.99. HRMS (ESI) m/z: $[M+Na]^+$ calculated for $C_{38}H_{39}N_3O_8Na$ 548.2009, found 548.2010.

Compound S10

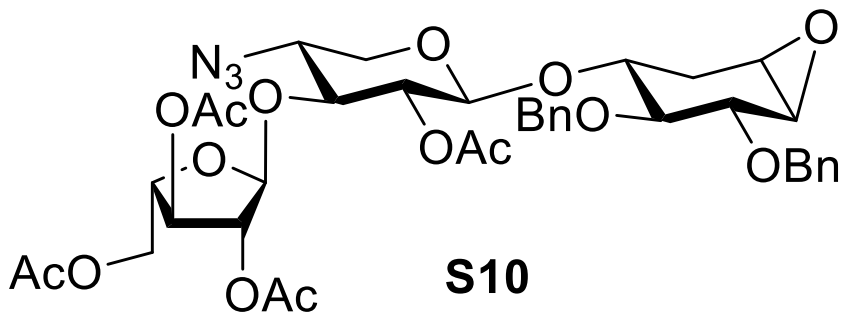

S10

Acceptor **S9** (26.3 mg, 0.05 mmol) and donor **23** (23 mg, 0.06 mmol) were co-evaporated with toluene for 3 times, re-dissolved in dry DCM (0.5 mL), and 3Å molecular sieves were added. The solution was stirred for 30 min and then cooled to -40 °C, after which NIS (14 mg, 0.06 mmol) and TMSOTf (1.1 mg, 0.005 mmol) were added to the stirring solution under nitrogen. The reaction mixture was stirred at the same temperature until TLC analysis indicated the full conversion of **S9**. Et_3N (0.1 mL) was then added and the solution was diluted with EtOAc, washed with sat. aq. $NaHCO_3$, 10% aq.

$Na_2S_2O_3$ and brine, dried over $MgSO_4$, filtered and concentrated under *in vacuo*. Flash column chromatography (EtOAc/pentane, 1/4 to 1/2, v/v) provided product **S10** (26.6 mg, 68%). ^{1}H NMR (500 MHz, $CDCl_3$) δ 7.38 – 7.26 (m, 10H), 5.22 (s, 1H, H1'', α-1,3), 5.03 – 4.88 (m, 4H), 4.79 – 4.65 (m, 3H), 4.48 – 4.40 (m, 2H), 4.36 (d, J = 7.4 Hz, 1H, H1', β-1,4), 4.27 – 4.21 (m, 1H), 3.99 (dd, J = 12.0, 5.4 Hz, 1H, H5'a), 3.80 (dd, J = 7.7, 0.8 Hz, 1H), 3.77 – 3.65 (m, 2H), 3.48 (ddd, J = 10.3, 9.1, 5.4 Hz, 1H), 3.39 (dd, J = 10.1, 7.7 Hz, 1H), 3.28 – 3.20 (m, 1H, H6), 3.15 – 3.04 (m, 2H, H1, H5'b), 2.49 (ddd, J = 14.4, 5.1, 2.5 Hz, 1H, H5a), 2.11 – 2.07 (m, 12H, 4xOAc), 1.72 (ddd, J = 14.5, 10.5, 1.5 Hz, 1H, H5b). ^{13}C NMR (126 MHz, $CDCl_3$) δ 170.71, 170.42, 169.58, 169.27, 138.88, 137.86, 128.56, 128.26, 128.19, 127.96, 127.52, 106.41 (C1'', α-1,3), 99.94 (C1', β-1,4), 82.54, 81.47, 81.37, 79.45, 77.61, 76.78, 75.29, 74.26, 73.48, 73.12, 63.67, 63.44, 60.51, 54.03 (C1), 53.42 (C6), 30.08 (C5), 20.99, 20.87, 20.79, 20.74. HRMS (ESI) m/z: $[M+Na]^+$ calculated for $C_{38}H_{45}O_{15}N_3Na$ 806.2748, found 806.2750.

Compound S11

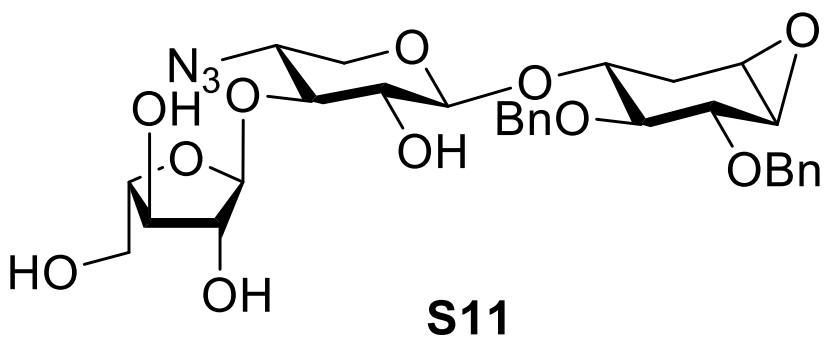

S11

To a solution of compound **S10** (39 mg, 0.05 mmol) in MeOH (1 mL) was added 4.4 M NaOMe (3.4 µL, 0.015 mmol) solution in MeOH. The mixture was stirred at rt for 6 hours and was then neutralized with $Et_3N \cdot HCl$ (2 mg, 15 µmol) and concentrated *in vacuo*. Flash column chromatography (DCM/MeOH = 20/1) afforded product **S11** (28 mg, 90%) as a white solid. ^{1}H NMR (500 MHz, $CDCl_3$) δ 7.40 – 7.26 (m, 10H), 5.46 (s, 1H), 4.91 – 4.52 (m, 5H), 4.33 – 4.19 (m, 2H), 4.11 (d, J = 34.8 Hz, 3H), 3.93 (dd, J = 11.8, 5.4 Hz, 1H), 3.89 – 3.67 (m, 5H), 3.63 (t, J = 9.4 Hz, 2H), 3.50 – 3.31 (m, 3H), 3.25 (d, J = 3.8 Hz, 1H), 3.19 – 2.99 (m, 2H), 2.64 – 2.52 (m, 1H), 2.12 (s, 1H), 1.82 (dd, J = 13.5, 10.4 Hz, 1H). ^{13}C NMR (126 MHz, $CDCl_3$) δ 138.30, 137.61, 128.64, 128.41, 128.27, 128.10, 128.06, 127.84, 108.63 (C1'', α-1,3), 101.66 (C1', β-1,4), 85.89, 82.07, 80.50, 79.53, 78.29, 77.18, 74.67, 73.64, 73.24, 72.06, 64.02, 61.41, 59.99, 53.74, 53.62, 30.52. HRMS (ESI) m/z: $[M+Na]^+$ calculated for $C_{30}H_{37}O_{11}N_3Na$ 638.2326, found 638.2323.

Compound S12

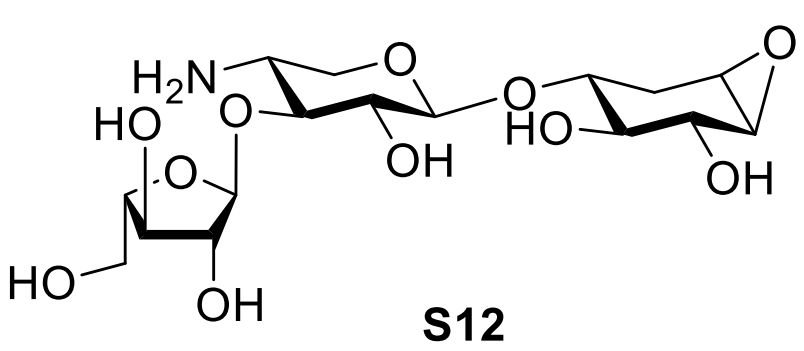

S12

Compound **S11** (25 mg, 0.04 mmol) was dissolved in a mixture of dioxane (1 mL) and H_2O (0.5 mL). The solution was purged with nitrogen for 3 minutes and TFA (3.1 µL, 40 µmol) was added under nitrogen atmosphere. The mixture was stirred for 3 minutes and $Pd(OH)_2/C$ (20 wt%, 42 mg, 60 µmol) was added. While stirring

vigorously, the flask was then purged with hydrogen for 5 minutes. The reaction mixture was stirred under hydrogen atmosphere and was monitored by LC-MS hourly. After 3 hours, LC-MS analysis indicated full conversion and the reaction mixture was then filtrated over celite and concentrated *in vacuo* to give product **S12** (16 mg, quant.) as an oil. ^{1}H NMR (500 MHz, D_2O) δ 5.26 (s, 1H, H1'', α-1,3), 4.44 (d, J = 7.5 Hz, 1H, H1', β-1,4), 4.09 (dtd, J = 11.0, 6.3, 5.7, 3.1 Hz, 3H), 3.92 (dd, J = 6.0, 4.3 Hz, 1H), 3.78 – 3.71 (m, 3H), 3.63 – 3.58 (m, 1H), 3.55 (dt, J = 10.3, 5.2 Hz, 1H), 3.50 – 3.42 (m, 2H), 3.41 (dq, J = 3.4, 1.7 Hz, 1H), 3.36 – 3.28 (m, 2H), 3.13 (d, J = 3.7 Hz, 1H, H1), 2.60 (ddd, J = 14.8, 5.4, 2.4 Hz, 1H, H5b), 1.86 – 1.75 (m, 1H, H5b). ^{13}C NMR (126 MHz, D_2O) δ 108.32 (C1'', α-1,3), 100.65 (C1', β-1,4), 84.24, 80.81, 77.86, 75.64, 74.50, 73.75, 72.61, 71.26, 61.52, 60.66, 56.25 (C1), 54.80 (C6), 49.79, 48.94, 30.90 (C5). HRMS (ESI) m/z: $[M+Na]^+$ calculated for $C_{16}H_{27}NO_{11}Na$ 409.1584, found 409.1588.

Compound 3-Bio

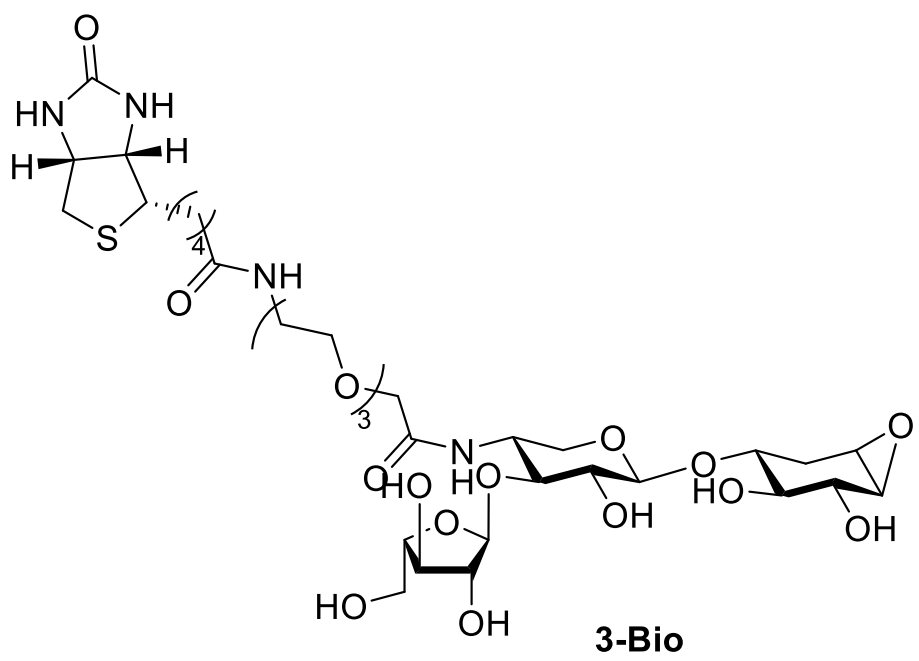

3-Bio

Amine **S12** (5 mg, 0.012 mmol) was reacted with **28** according to general procedure A, affording product **3-Bio** (2.6 mg, 26%) as a white powder after lyophilization. ^{1}H NMR (500 MHz, D_2O) δ 5.16 (d, J = 1.7 Hz, 1H, H1'', α-1,3), 4.52 (ddd, J = 7.9, 5.0, 0.8 Hz, 1H), 4.37 (d, J = 7.9 Hz, 1H, H1', β-1,4), 4.33 (dd, J = 8.0, 4.5 Hz, 1H), 4.08 – 3.90 (m, 4H), 3.89 – 3.79 (m, 3H), 3.77 – 3.47 (m, 15H), 3.45 – 3.18 (m, 7H), 3.12 (d, J = 3.7 Hz, 1H, H1), 2.90 (dd, J = 13.1, 5.0 Hz, 1H), 2.69 (d, J = 13.0 Hz, 1H), 2.59 (ddd, J = 14.8, 5.3, 2.2 Hz, 1H), 2.18 (t, J = 7.3 Hz, 2H), 1.68 – 1.45 (m, 4H), 1.32 (p, J = 7.2 Hz, 2H). ^{13}C NMR (126 MHz, D_2O) δ 172.71, 108.57 (C1'', α-1,3), 100.76 (C1', β-1,4), 84.05, 81.23, 79.10, 76.24, 74.43, 73.71, 73.48, 71.14, 70.15, 69.64, 69.55, 69.48, 69.33, 68.87, 63.14, 62.05, 60.90, 60.21, 56.99, 56.13 (C1), 55.31, 54.70 (C6), 48.97, 39.65, 38.84, 35.41, 28.77 (C5), 27.82, 27.64, 25.08. HRMS (ESI) m/z: $[M+H]^+$ calculated for $C_{34}H_{57}N_4O_{17}S$ 825.3434, found 825.3430.

Compound 3-Cy5

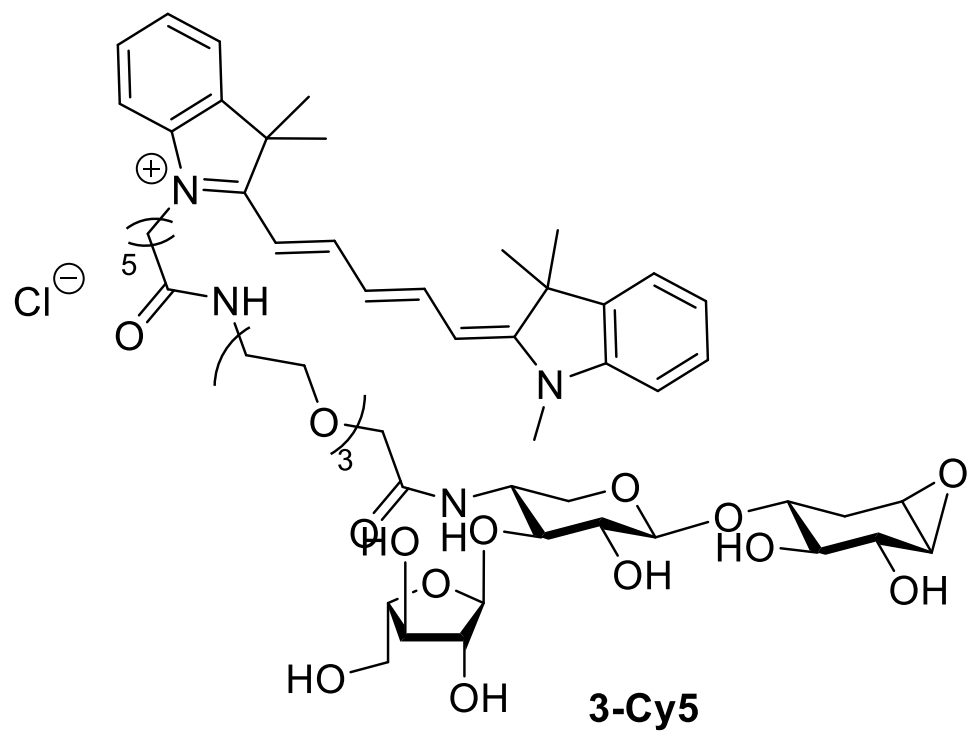

3-Cy5

Amine **S12** (5 mg, 0.012 mmol) was reacted with **21** according to general procedure A, affording product **3-Cy5** (3.3 mg, 25%) as a blue powder after lyophilization. ^{1}H NMR (500 MHz, D_2O) δ 8.27 (t, J = 12.7 Hz, 1H), 8.09 (d, J = 8.3 Hz, 2H), 7.95 – 7.89 (m, 1H), 7.72 – 7.63 (m, 1H), 7.53 – 7.41 (m, 6H), 7.34 – 7.26 (m, 4H), 6.65 (s, 1H), 6.31 (dd, J = 13.8, 3.3 Hz, 2H), 5.32 – 5.29 (d, 1H, H1'', α-1,3)), 4.69 – 4.58 (m, 8H), 4.35 (m, J = 11.4, 7.5 Hz, 2H), 4.17 – 4.09 (m, 4H), 4.08 (d, J = 1.5 Hz, 1H), 4.06 – 3.93 (m, 11H), 3.88 (d, J = 1.5 Hz, 4H), 3.80 – 3.62 (m, 25H), 3.61 – 3.52 (m, 11H), 3.51 – 3.43 (m, 4H), 3.21 (q, J = 5.8, 5.1 Hz, 2H), 3.01 (d, J = 3.6 Hz, 1H), 2.60 (d, J = 12.8 Hz, 1H), 2.45 – 2.33 (m, 2H), 2.29 – 2.18 (m, 4H), 2.08 (d, J = 5.8 Hz, 1H), 1.94 (s, 3H), 1.86 (dd, J = 13.3, 7.5 Hz, 3H), 1.75 (s, 21H), 1.58 – 1.43 (m, 12H), 1.39 – 1.28 (m, 3H), 1.23 – 1.16 (m, 3H). ^{13}C NMR (126 MHz, MeOD) δ 175.16, 174.58, 173.85, 172.09, 154.72, 143.46, 142.78, 141.85, 141.73, 129.00, 128.94, 125.83, 125.47, 125.44, 122.63, 122.48, 111.28, 111.05, 109.07 (C1'', α-1,3), 103.60, 103.50, 99.76 (C1', β-1,4), 85.78, 82.12, 78.26, 77.17, 75.91, 73.69, 72.24, 70.96, 70.69, 70.67, 70.47, 70.32, 69.76, 62.46, 62.37, 56.17 (C1), 53.94 (C6), 50.69, 49.75, 49.71, 43.98, 39.50, 35.86, 30.73, 29.42 (C5), 27.41, 27.16, 27.01, 26.58, 25.73, 23.00. HRMS (ESI) m/z: $[M]^+$ calculated for $C_{56}H_{79}N_4O_{16}$ 1063.5486, found 1063.5489.

Compound S13

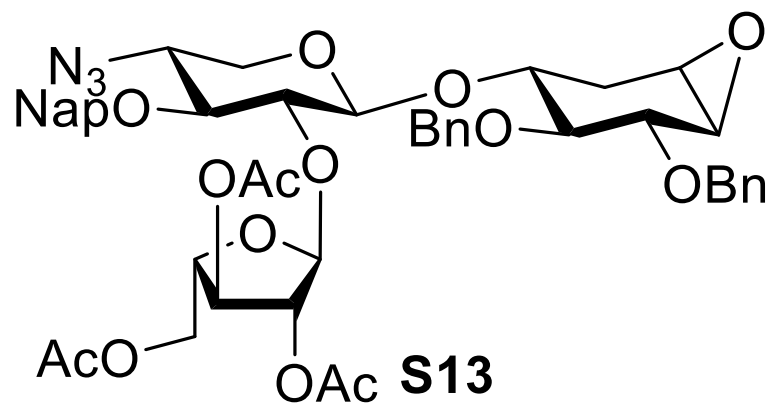

S13

Acceptor **15** (46 mg, 0.075 mmol) and donor **23** (35 mg, 0.09 mmol) were co-evaporated with toluene for 3 times, re-dissolved in dry DCM (0.5 mL), and 3Å molecular sieves were added. The solution was stirred for 30 min and then cooled to -40 °C, after which NIS (20 mg, 0.09 mmol) and TMSOTf (1.7 mg, 7.5 µmol) were added to the stirring solution under nitrogen. The reaction mixture was stirred at the same temperature until TLC analysis indicated the full conversion of **15**. Et_3N (0.1 mL) was then added and the solution was diluted with EtOAc, washed with sat. aq. $NaHCO_3$, 10% aq. $Na_2S_2O_3$ and brine, dried over $MgSO_4$, filtered and concentrated under *in vacuo*. Flash column chromatography (EtOAc/pentane, 1/4 to 2/3, v/v) provided product **S13** (46 mg, 75%). ^{1}H NMR (400 MHz, $CDCl_3$) δ 7.88 – 7.75 (m, 4H), 7.50 – 7.43 (m, 3H), 7.38 – 7.26 (m, 10H), 5.40 (s, 1H, H1'', α-1,2), 5.16 (d, J = 1.6 Hz, 1H), 5.07 – 4.89 (m, 4H), 4.79 – 4.64 (m, 3H), 4.48 (td, J = 5.6, 3.4

Hz, 1H), 4.39 (d, J = 7.1 Hz, 1H, H1', β-1,4), 4.34 (dd, J = 12.0, 3.5 Hz, 1H), 4.19 (dd, J = 12.0, 5.9 Hz, 1H), 4.04 – 3.83 (m, 3H), 3.71 (t, J = 7.9 Hz, 1H), 3.65 – 3.51 (m, 2H), 3.42 (dd, J = 10.1, 7.6 Hz, 1H), 3.33 – 3.24 (m, 1H, H1), 3.21 – 3.05 (m, 2H, H6, H5'b), 2.65 (ddd, J = 14.5, 5.1, 2.5 Hz, 1H, H5a), 2.16 – 2.07 (m, 1H, H5b), 2.04 (s, 3H), 1.97 (s, 3H), 1.84 (s, 3H). ^{13}C NMR (100 MHz, $CDCl_3$) δ 170.65, 169.94, 169.61, 138.78, 137.79, 135.14, 133.32, 133.03, 128.59, 128.55, 128.33, 128.16, 128.12, 128.04, 128.02, 127.75, 127.64, 126.45, 126.21, 126.06, 125.70, 106.27 (C1'', α-1,2), 99.50 (C1', β-1,4), 83.26, 82.90, 81.36, 80.30, 79.31, 77.67, 76.88, 75.33, 75.16, 73.60, 72.52, 63.64, 63.45, 61.54, 53.93 (C1), 53.43 (C6), 29.51 (C5), 20.80, 20.77, 20.56. HRMS (ESI) m/z: $[M+Na]^+$ calculated for $C_{47}H_{51}O_{14}N_3Na$ 904.3269, found 904.3270.

Compound S14

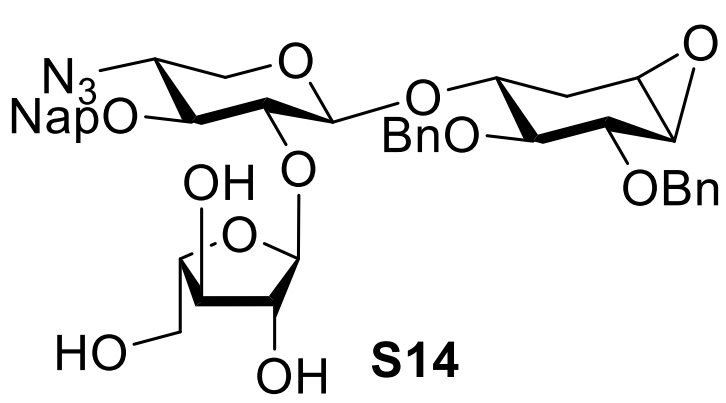

S14

To a solution of compound **S13** (44 mg, 0.05 mmol) in MeOH (1 mL) was added 4.4 M NaOMe (3.4 µL, 0.015 mmol) solution in MeOH. The mixture was stirred at rt for 6 hours and was then neutralized with Et_3N·HCl (2 mg, 15 µmol) and concentrated *in vacuo*. Flash column chromatography (DCM/MeOH = 20/1) afforded product **S14** (28 mg, 90%) as a white solid. ^{1}H NMR (400 MHz, $CDCl_3$) δ 7.96 – 7.81 (m, 4H), 7.60 – 7.47 (m, 3H), 7.42 – 7.26 (m, 10H), 5.46 (d, J = 0.8 Hz, 1H), 5.07 (d, J = 10.9 Hz, 1H), 4.95 (d, J = 10.9 Hz, 1H), 4.88 (d, J = 11.1 Hz, 1H), 4.82 – 4.72 (m, 2H), 4.69 (d, J = 11.0 Hz, 1H), 4.34 (d, J = 7.4 Hz, 1H), 4.24 (q, J = 2.1 Hz, 1H), 4.07 – 3.95 (m, 4H), 3.88 (dd, J = 7.7, 0.8 Hz, 1H), 3.79 (dd, J = 8.9, 7.3 Hz, 1H), 3.72 – 3.59 (m, 3H), 3.55 (t, J = 9.0 Hz, 1H), 3.46 (dd, J = 10.3, 7.6 Hz, 1H), 3.30 (dq, J = 3.1, 1.5 Hz, 1H), 3.17 (d, J = 3.6 Hz, 1H), 3.12 (dd, J = 12.0, 10.3 Hz, 1H), 2.64 (s, 1H), 2.58 (ddd, J = 14.2, 5.2, 2.5 Hz, 1H). ^{13}C NMR (101 MHz, $CDCl_3$) δ 138.63, 137.67, 134.49, 133.28, 133.15, 128.55, 128.46, 128.29, 128.09, 128.01, 127.99, 127.73, 127.66, 127.59, 126.38, 126.30, 126.26, 108.31, 98.24, 87.71, 83.06, 82.15, 79.33, 78.94, 78.37, 75.38, 74.74, 74.72, 73.40, 71.17, 63.76, 61.90, 61.62, 53.69, 53.45, 28.95. HRMS (ESI) m/z: $[M+Na]^+$ calculated for $C_{41}H_{45}O_{11}N_3Na$ 778.2952, found 778.2955.

Compound S15

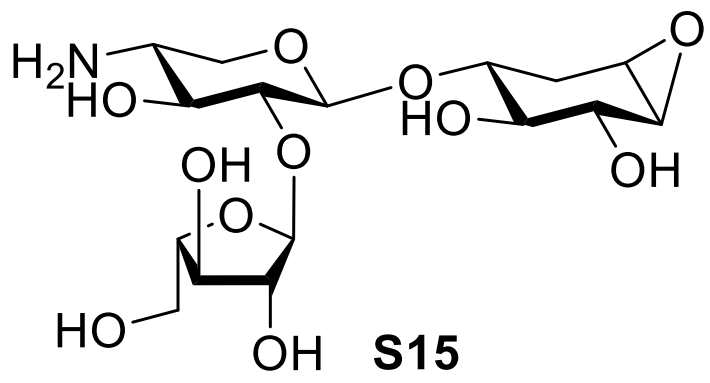

S15

Compound **S14** (30 mg, 0.04 mmol) was dissolved in a mixture of dioxane (1 mL) and H_2O (0.5 mL). The solution was purged with nitrogen for 3 minutes and TFA (3.1 µL, 40 µmol) was added under nitrogen atmosphere. The mixture was stirred for 3 minutes and $Pd(OH)_2/C$ (20 wt%, 42 mg, 60 µmol) was added. While stirring vigorously, the flask was then purged with hydrogen for 5 minutes. The reaction mixture was stirred under hydrogen atmosphere and was monitored by LC-MS hourly. After 4 hours, LC-MS analysis indicated full conversion and the reaction mixture was then filtrated over celite and concentrated *in vacuo* to give product **S15** (16 mg, quant.) as an oil. ^1H NMR (500 MHz, D_2O) δ 5.19 (s, 1H, H1'', α-1,2), 4.56 (dd, J = 6.6, 3.3 Hz, 1H, H1', β-1,4), 4.08 (tdt, J = 7.3, 2.8, 1.7 Hz, 3H, H5'a, H2'', H4''), 3.87 (ddd, J = 5.6, 2.7, 0.7 Hz, 1H, H3''), 3.76 – 3.68 (m, 3H, H2, H3', H5''a), 3.63 (dd, J = 12.3, 5.9 Hz, 1H, H5''b), 3.55 (td, J = 10.3, 5.2 Hz, 1H, H4), 3.48 – 3.41 (m, 2H, H2', H5'b), 3.40 (dt, J = 4.0, 2.1 Hz, 1H, H6), 3.35 (dd, J = 10.3, 8.2 Hz, 1H, H3), 3.22 – 3.16 (m, 1H, H, H4'), 3.12 (d, J = 3.7 Hz, 1H, H1), 2.62 (ddd, J = 14.8, 5.4, 2.4 Hz, 1H, H5a), 1.86 – 1.82 (m, 1H, H5b). ^{13}C NMR (126 MHz, D_2O) δ 108.51 (C1'', α-1,2), 98.69 (C1', β-1,4), 84.78 (C4''), 81.07 (C2''), 77.48 (C2'), 76.78 (C3''), 74.55 (C3), 72.97 (C4), 71.52 (C3'), 71.18 (C2), 61.27 (C5'), 61.27 (C5''), 56.23 (C1), 54.80 (C6), 50.87 (C4'), 28.61(C5). HRMS (ESI) m/z: $[M+Na]^+$ calculated for $C_{16}H_{27}NO_{11}Na$ 432.1482, found 432.1486.

Compound 2-Bio

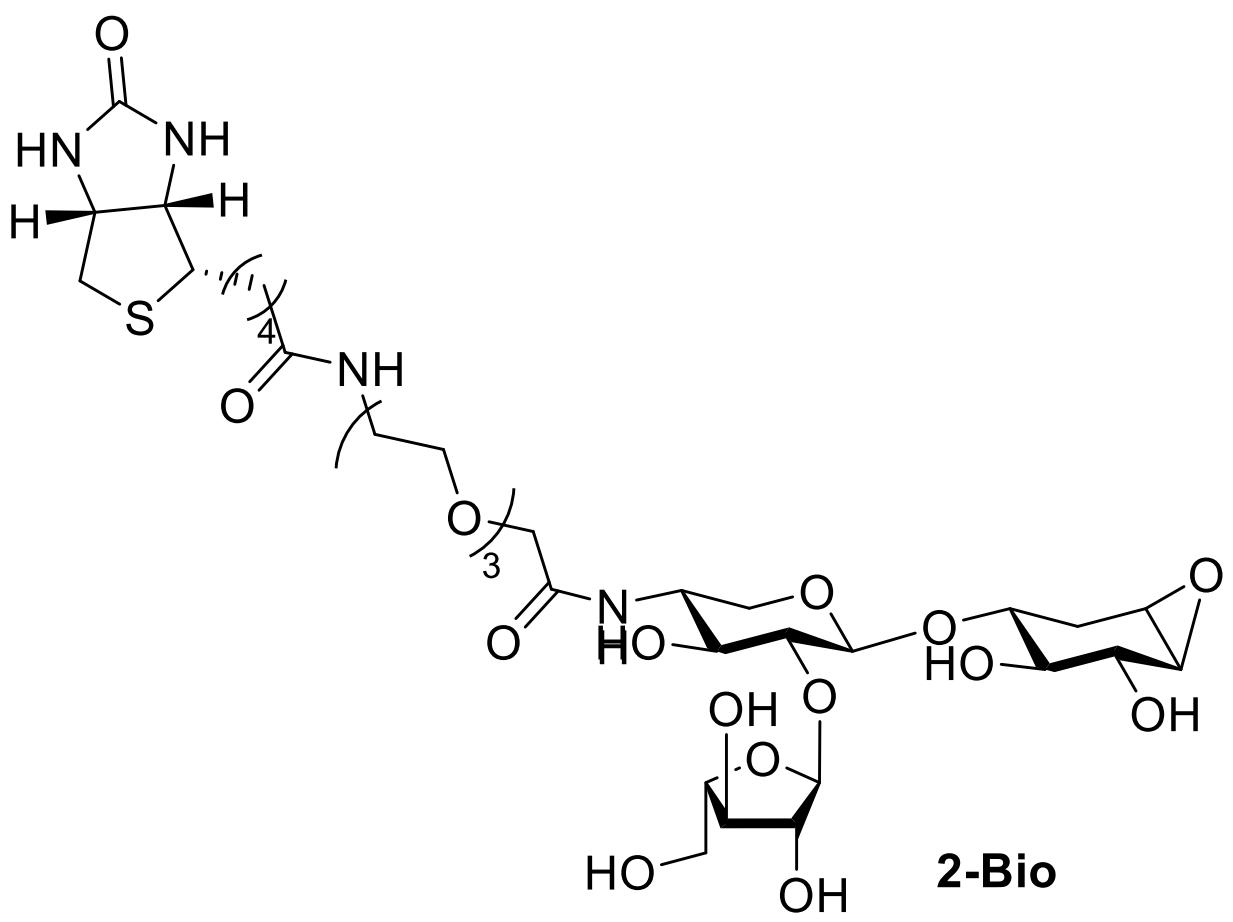

2-Bio

Amine **S15** (5 mg, 0.012 mmol) was reacted with **28** according to general procedure A, affording product **2-Bio** (2.8 mg, 28%) as a white powder after lyophilization. ^1H NMR (500 MHz, D_2O) δ 5.22 (d, J = 1.1 Hz, 1H), 4.51 (dd, J = 7.9, 4.9 Hz, 1H), 4.46 (d, J = 7.4 Hz, 1H), 4.10 – 4.05 (m, 2H), 4.02 (d, J = 1.1 Hz, 2H), 3.92 – 3.84 (m, 4H), 3.75 – 3.70 (m, 2H), 3.66 – 3.60 (m, 11H), 3.54 (p, J = 5.0 Hz, 3H), 3.43 – 3.38 (m, 2H), 3.34 (dd, J = 10.2, 8.2 Hz, 1H), 3.31 (t, J = 5.4 Hz, 2H), 3.28 – 3.19 (m, 3H), 3.11 (d, J = 3.7 Hz, 1H), 2.90 (dd, J = 13.0, 5.0 Hz, 1H), 2.69 (d, J = 13.0 Hz, 1H), 2.61 (ddd, J = 14.8, 5.2, 2.4 Hz, 1H), 2.18 (t, J = 7.3 Hz, 2H), 1.56 (dddd, J = 50.1, 31.5, 13.7, 7.4 Hz, 5H), 1.32 (p, J = 7.1 Hz, 2H). ^{13}C NMR (126 MHz, D_2O) δ 177.00, 173.01,

166.98, 110.04, 100.26, 84.61, 81.13, 78.18, 77.14, 75.40, 73.23, 72.78, 71.17, 70.25, 69.72, 69.59, 69.55, 69.40, 68.96, 62.86, 62.14, 61.22, 60.31, 56.62, 55.41, 54.79, 50.46, 39.74, 38.93, 35.52, 28.71, 27.92, 27.74, 25.18. $[M+H]^+$ calculated for $C_{34}H_{57}N_4O_{17}S$ 825.3434, found 825.3431.

Compound 2-Cy5

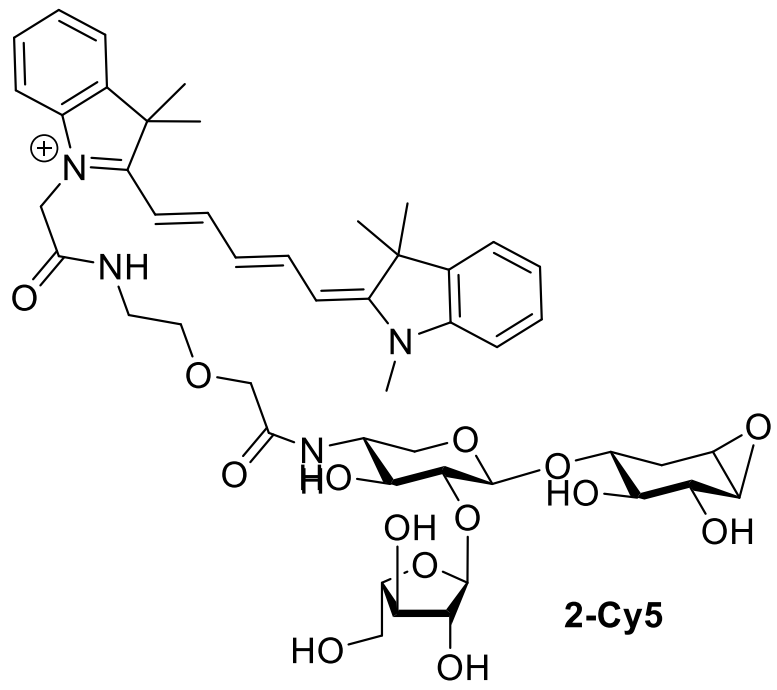
2-Cy5

Amine **S15** (5 mg, 0.012 mmol) was reacted with **21** according to general procedure A, affording product **2-Cy5** (2.8 mg, 21%) as a blue powder after lyophilization. ^{1}H NMR (500 MHz, D_2O) δ 8.34 – 8.18 (m, 2H), 7.51 (d, J = 7.6 Hz, 2H), 7.43 (dd, J = 7.7, 1.2 Hz, 2H), 7.36 – 7.26 (m, 5H), 6.65 (t, J = 12.4 Hz, 1H), 6.31 (dd, J = 13.7, 3.9 Hz, 2H), 5.27 (s, 1H, H1'', α-1,2), 4.58 (d, J = 7.9 Hz, 1H, H1', β-1,4), 3.02 (d, J = 3.5 Hz, 1H), 2.65 (ddd, J = 14.9, 5.3, 2.4 Hz, 1H), 2.31 – 2.17 (m, 3H), 2.16 – 2.01 (m, 2H), 1.96 (s, 2H), 1.86 (ddd, J = 14.6, 10.3, 1.7 Hz, 4H), 1.55 – 1.44 (m, 3H), 1.42 – 1.20 (m, 5H), 0.99 – 0.84 (m, 1H). ^{13}C NMR (126 MHz, MeOD) δ 174.56, 173.98, 173.25, 171.49, 154.12, 142.86, 142.18, 141.25, 141.13, 128.40, 128.34, 125.23, 124.85, 122.03, 121.88, 110.68, 110.45, 108.46 (C1'', α-1,2), 103.00, 102.90, 99.16 (C1', β-1,4), 85.18, 81.52, 77.66, 76.57, 75.31, 73.10, 71.64, 70.36, 70.07, 69.87, 69.72, 69.16, 61.86, 61.77, 61.64, 56.20, 55.57 (C1), 53.34 (C6), 50.09, 49.15, 43.38, 38.90, 35.26, 30.13, 28.82 (C5), 26.81, 26.56, 26.41, 25.98, 25.12, 22.40. HRMS (ESI) m/z: $[M]^+$ calculated for $C_{56}H_{79}N_4O_{16}$ 1063.5486, found 1063.5488.

Compound S16

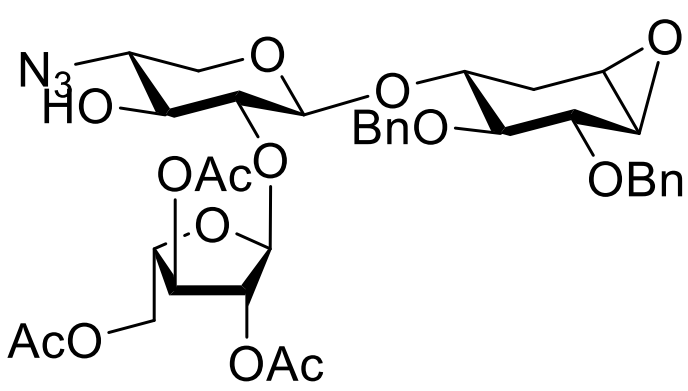
S16

Compound **S13** (48 mg, 0.054 mmol) was dissolved in a mixture of DCM/H_2O (19/1, v/v, 2 mL). DDQ (15 mg, 0.065 mmol) was added to the mixture. The reaction was stirred until TLC-analysis indicated full consumption of **S13**. The mixture was then diluted with DCM, washed with sat. aq. $NaHCO_3$ and brine, dried over Na_2SO_4, filtrated and concentrated under reduced pressure. Flash column chromatography of the crude afforded product **S16** (35 mg, 85%) as a white solid. ^{1}H NMR (500 MHz, $CDCl_3$) δ 7.40 – 7.26 (m, 10H), 5.37 (s, 1H, H1'', α-1,2), 5.14 – 5.05 (m, 2H), 4.90 (d, J = 11.0 Hz, 1H), 4.83 – 4.65 (m, 3H), 4.48 – 4.32 (m, 3H), 4.29 – 4.17 (m, 1H), 3.94 (dd, J = 12.0, 5.2 Hz, 1H), 3.91 – 3.79 (m, 2H), 3.69 (td, J = 8.7, 3.7 Hz, 1H), 3.57 – 3.39 (m, 4H), 3.29 (dq, J = 3.3, 1.9

Hz, 1H, H6), 3.16 (d, J = 3.6 Hz, 1H, H1), 3.07 (dd, J = 12.1, 9.9 Hz, 1H), 2.66 (ddd, J = 14.6, 5.3, 2.4 Hz, 1H, H5a), 2.13 (s, 6H), 2.07 (s, 3H), 1.84 (ddd, J = 14.6, 10.4, 1.5 Hz, 1H, H5b). ^{13}C NMR (126 MHz, $CDCl_3$) δ 170.76, 170.69, 169.93 (3xC=O), 138.78, 137.79, 128.60, 128.31, 128.18, 128.01, 127.64, 106.82 (C1'', α-1,2), 99.49 (C1', β-1,4), 82.73, 82.13, 80.65, 79.46, 79.11, 76.79, 75.29, 75.20, 73.46, 72.76, 63.33, 63.25, 60.54, 53.86 (C1), 53.45 (C6), 29.64 (C5), 20.89, 20.87, 20.82 (3x Me). HRMS (ESI) m/z: $[M+Na]^+$ calculated for $C_{36}H_{43}N_3O_{14}Na$ 764.2643, found 764.2642.

Compound S17

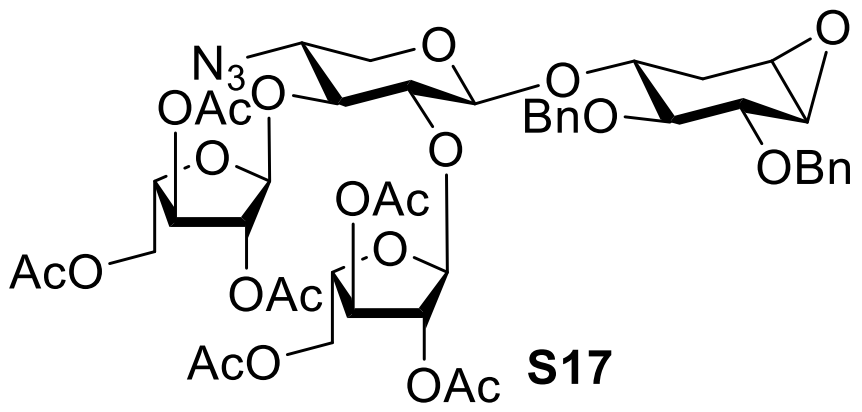

S17

Acceptor **S16** (35 mg, 0.047 mmol) and donor **23** (23.4 mg, 0.061 mmol) were co-evaporated with toluene for 3 times, re-dissolved in dry DCM (0.5 mL), and 3Å molecular sieves were added. The solution was stirred for 30 min and then cooled to -40 °C, after which NIS (13.6 mg, 0.061 mmol) and TMSOTf (1 mg, 4.7 µmol) were added to the stirring solution under nitrogen. The reaction mixture was stirred at the same temperature until TLC analysis indicated the full conversion of **S16**. Et_3N (0.1 mL) was then added and the solution was diluted with EtOAc, washed with sat. aq. $NaHCO_3$, 10% aq. $Na_2S_2O_3$ and brine, dried over $MgSO_4$, filtered and concentrated under *in vacuo*. Flash column chromatography (EtOAc/pentane, 1/2 to 2/1, v/v) provided product **S17** (29 mg, 61%). ^{1}H NMR (500 MHz, $CDCl_3$) δ 7.41 – 7.24 (m, 10H), 5.47 (s, 1H, H1''', α-1,3), 5.31 (s, 1H, H1'', α-1,2), 5.12 (d, J = 1.3 Hz, 1H), 5.01 (d, J = 1.0 Hz, 1H), 4.99 – 4.84 (m, 3H), 4.82 – 4.63 (m, 3H), 4.48 (ddt, J = 11.6, 7.7, 3.8 Hz, 3H), 4.42 – 4.31 (m, 2H), 4.28 – 4.14 (m, 2H), 4.02 (dd, J = 11.9, 5.5 Hz, 1H), 3.95 – 3.83 (m, 2H), 3.74 (t, J = 9.3 Hz, 1H), 3.64 (dd, J = 9.1, 6.9 Hz, 1H), 3.44 (dd, J = 10.2, 7.6 Hz, 1H), 3.35 (td, J = 9.7, 5.5 Hz, 1H), 3.31 – 3.24 (m, 1H), 3.22 – 3.07 (m, 2H), 2.62 (ddd, J = 14.4, 5.1, 2.3 Hz, 1H), 2.14 – 2.08 (m, 15H), 2.02 (s, 3H), 1.92 – 1.83 (m, 1H). ^{13}C NMR (126 MHz, $CDCl_3$) δ 177.57, 170.79, 170.72, 170.44, 170.02, 169.75, 169.66, 138.87, 137.79, 128.59, 128.34, 128.03, 127.63, 106.39 (C1'', α-1,2), 106.17 (C1''', α-1,3), 99.15 (C1', β-1,4), 82.76, 81.67, 81.12, 80.81, 80.50, 79.37, 78.99, 77.59, 77.57, 75.22, 73.52, 71.94, 63.71, 63.62, 63.59, 60.85, 53.87 (C1), 53.50 (C6), 29.02 (C5), 20.90, 20.88, 20.83, 20.82, 20.80, 20.77. HRMS (ESI) m/z: $[M+Na]^+$ calculated for $C_{47}H_{57}O_{21}N_3Na$ 1022.3382, found 1022.3385.

Compound S18

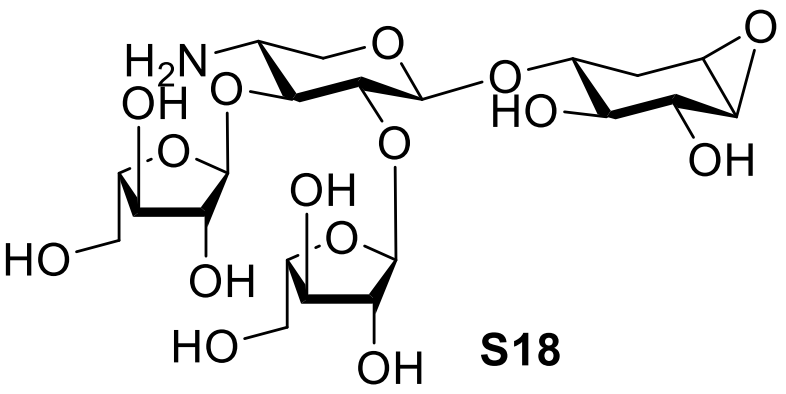

To a solution of compound **S17** (29 mg, 0.029 mmol) in MeOH (1 mL) was added 4.4 M NaOMe (2 µL, 0.009 mmol) solution in MeOH. The mixture was stirred at rt for overnight and was then neutralized with $Et_3N{\cdot}HCl$ (1.2 mg, 9 µmol) and concentrated *in vacuo*. The crude was preliminarily purified by flash column chromatography (DCM/MeOH = 10/1) and was then re-dissolved in a mixture of dioxane (1 mL) and H_2O (0.5 mL). The solution was purged with nitrogen for 3 minutes and TFA (2.2 µL, 29 µmol) was added under nitrogen atmosphere. The mixture was stirred for 3 minutes and $Pd(OH)_2/C$ (20 wt%, 30 mg, 44 µmol) was added. While stirring vigorously, the flask was then purged with hydrogen for 5 minutes. The reaction mixture was stirred under hydrogen atmosphere and was monitored by LC-MS hourly. After 3 hours, LC-MS analysis indicated full conversion and the reaction mixture was then filtrated over celite and concentrated *in vacuo* to give product **S18** (13 mg, quant.) as an oil. ^{1}H NMR (500 MHz, D_2O) δ 5.21 – 5.11 (m, 2H, H1'', H1''', α-1,3, α-1,2), 4.65 – 4.64 (m, 1H, H1', β-1,4), 4.18 (dd, J = 12.2, 4.3 Hz, 1H, H5'a), 4.11 – 4.06 (m, 4H, H2'', H2''', H4'', H4'''), 3.92 – 3.86 (m, 3H, H3'', H3''', H3'), 3.75 – 3.70 (m, 3H, H5''a, H5'''a, H2), 3.67 – 3.46 (m, 5H, H2', H4, H5''b, H5'''b, H5'b), 3.42 – 3.34 (m, 3H, H4', H6, H3), 3.12 (d, J = 3.7 Hz, 1H, H1), 2.68 – 2.55 (m, 1H, H5a), 1.82 (ddd, J = 14.7, 10.5, 1.7 Hz, 1H, H5b). ^{13}C NMR (126 MHz, D_2O) δ 108.70 (C1'', α-1,2), 108.54 (C1''', α-1,3), 98.10 (C1', β-1,4), 84.82 (C2''), 84.17 (C2'''), 81.11 (C4''), 80.68 (C4'''), 76.81, 76.53, 75.56, 74.59, 72.47, 71.18, 61.25 (C5'', C5'''), 60.05 (C5'), 56.23 (C1), 54.77 (C6), 49.49 (C4'), 28.45 (C5). HRMS (ESI) m/z: $[M+Na]^+$ calculated for $C_{21}H_{35}O_{15}NNa$ 564.1904, found 564.1900.

Compound 5-Bio

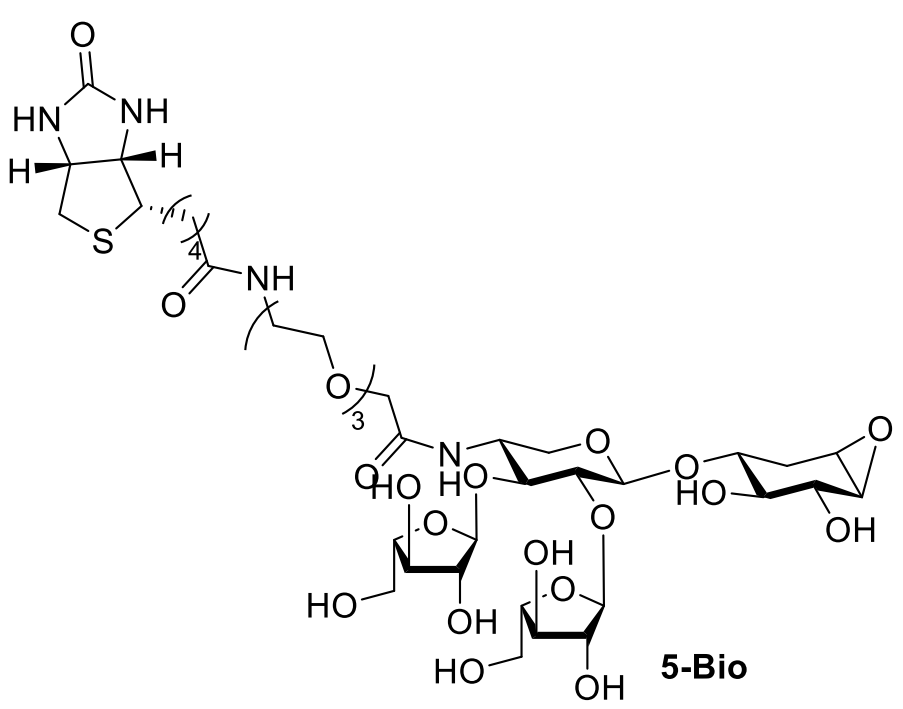

Amine **S18** (6.5 mg, 0.012 mmol) was reacted with **28** according to general procedure A, affording product **5-Bio** (3.2 mg, 28%) as a white powder after lyophilization. ^{1}H NMR (500 MHz, D_2O) δ 5.18 (d, J = 1.2 Hz, 1H), 5.08 (d, J = 1.7 Hz, 1H), 4.52 (dd, J = 8.0, 4.9 Hz, 1H), 4.49 (d, J = 7.4 Hz, 1H), 4.33 (dd, J = 7.9, 4.5 Hz, 1H), 4.10 – 3.94 (m, 6H), 3.92 – 3.82 (m, 4H), 3.78 – 3.59 (m, 15H), 3.58 – 3.50 (m, 4H), 3.40 (d, J = 3.3 Hz, 1H), 3.37 – 3.27 (m, 5H), 3.12 (d, J = 3.7 Hz, 1H), 2.91 (dd, J = 13.1, 5.0 Hz, 1H), 2.69 (d, J = 13.0 Hz, 1H), 2.66 – 2.59 (m, 1H), 2.19 (t, J = 7.3 Hz, 2H), 1.83 (dd, J = 14.3, 10.7 Hz, 1H), 1.57 (dddq, J = 52.7, 22.7, 15.3, 8.2, 7.8 Hz, 4H),

1.33 (p, J = 7.0, 6.4 Hz, 2H). ^{13}C NMR (126 MHz, D_2O) δ 176.99, 172.71, 109.10, 108.81, 98.90, 84.39, 84.25, 81.36, 81.22, 79.66, 78.68, 76.80, 76.34, 74.63, 72.82, 71.15, 70.28, 69.76, 69.65, 69.60, 69.45, 68.99, 62.89, 62.15, 61.15, 60.94, 60.32, 56.20, 55.42, 54.79, 49.24, 48.91, 39.75, 38.96, 35.53, 28.59, 27.94, 27.75, 25.19. HRMS (ESI) m/z: $[M+H]^+$ calculated for $C_{39}H_{65}N_4O_{21}S$ 957.3857, found 957.3855.

Compound 5-Cy5

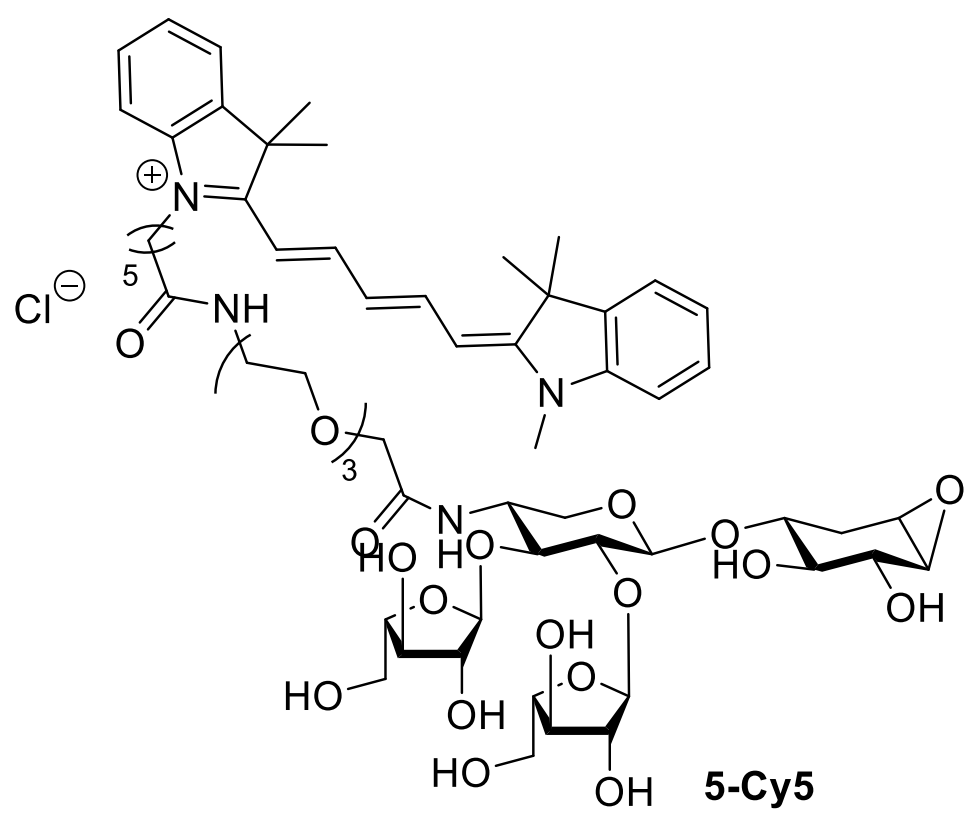

5-Cy5

Amine **S18** (6.5 mg, 0.012 mmol) was reacted with **21** according to general procedure A, affording product **5-Cy5** (3.0 mg, 20%) as a blue powder after lyophilization. ^{1}H NMR (500 MHz, D_2O) δ 7.77 (s, 2H), 7.34 (d, J = 7.5 Hz, 2H), 7.23 (d, J = 7.2 Hz, 2H), 7.15 (d, J = 8.0 Hz, 2H), 7.09 (t, J = 7.2 Hz, 2H), 6.33 (t, J = 12.3 Hz, 1H), 6.02 (dd, J = 24.7, 13.3 Hz, 2H), 5.12 (s, 1H), 5.04 (d, J = 1.7 Hz, 1H), 4.45 – 4.38 (m, 1H), 4.07 – 3.95 (m, 8H), 3.90 (s, 1H), 3.89 – 3.78 (m, 7H), 3.72 – 3.65 (m, 5H), 3.64 – 3.59 (m, 3H), 3.58 – 3.43 (m, 18H), 3.38 (d, J = 5.2 Hz, 3H), 3.35 (s, 1H), 3.32 (dd, J = 10.2, 8.0 Hz, 1H), 3.18 (t, J = 5.5 Hz, 3H), 3.07 (d, J = 3.9 Hz, 1H), 2.54 (d, J = 13.0 Hz, 1H), 2.15 – 2.11 (m, 2H), 1.82 – 1.76 (m, 1H), 1.72 (s, 2H), 1.53 (t, J = 7.4 Hz, 2H), 1.42 (d, J = 10.4 Hz, 12H), 1.24 (d, J = 7.4 Hz, 2H). ^{13}C NMR (126 MHz, D_2O) δ 179.57, 176.44, 174.31, 172.42, 142.76, 142.18, 141.23, 129.79, 124.90, 124.04, 121.59, 110.96, 110.79, 109.07, 108.79, 98.69, 84.37, 84.18, 81.37, 81.24, 79.46, 78.56, 76.81, 76.31, 74.64, 72.64, 71.17, 70.22, 69.73, 69.65, 69.58, 69.45, 68.86, 61.14, 60.92, 56.14, 54.71, 49.16, 48.98, 48.91, 38.86, 35.43, 30.84, 28.44, 27.00, 26.81, 26.69, 25.48, 25.06. HRMS (ESI) m/z: $[M]^+$ calculated for $C_{61}H_{87}N_4O_{20}$ 1095.5908, found 1095.5910.

NMR spectra:

^{1}H and ^{13}C of compound **S2** in $CDCl_3$

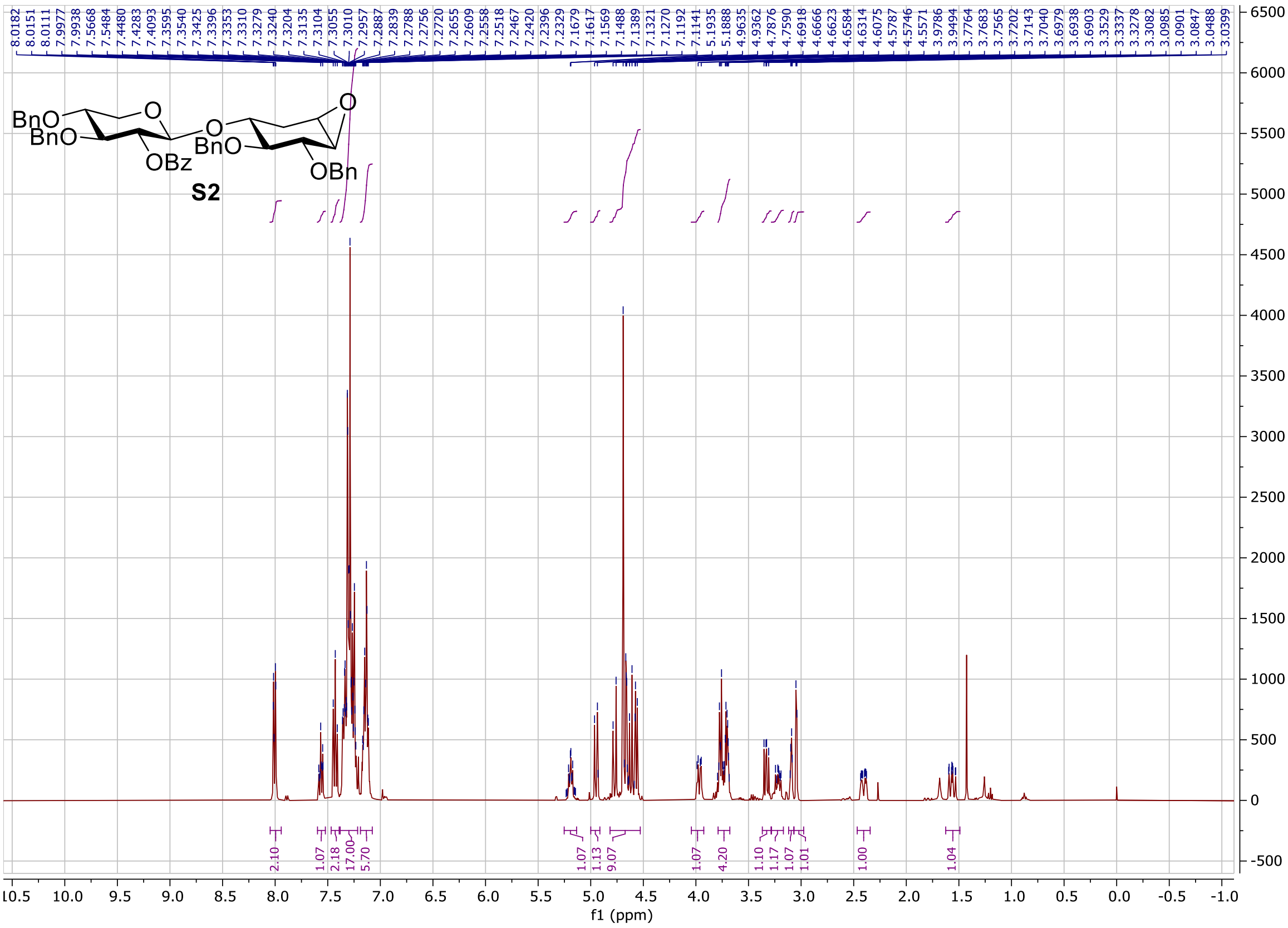

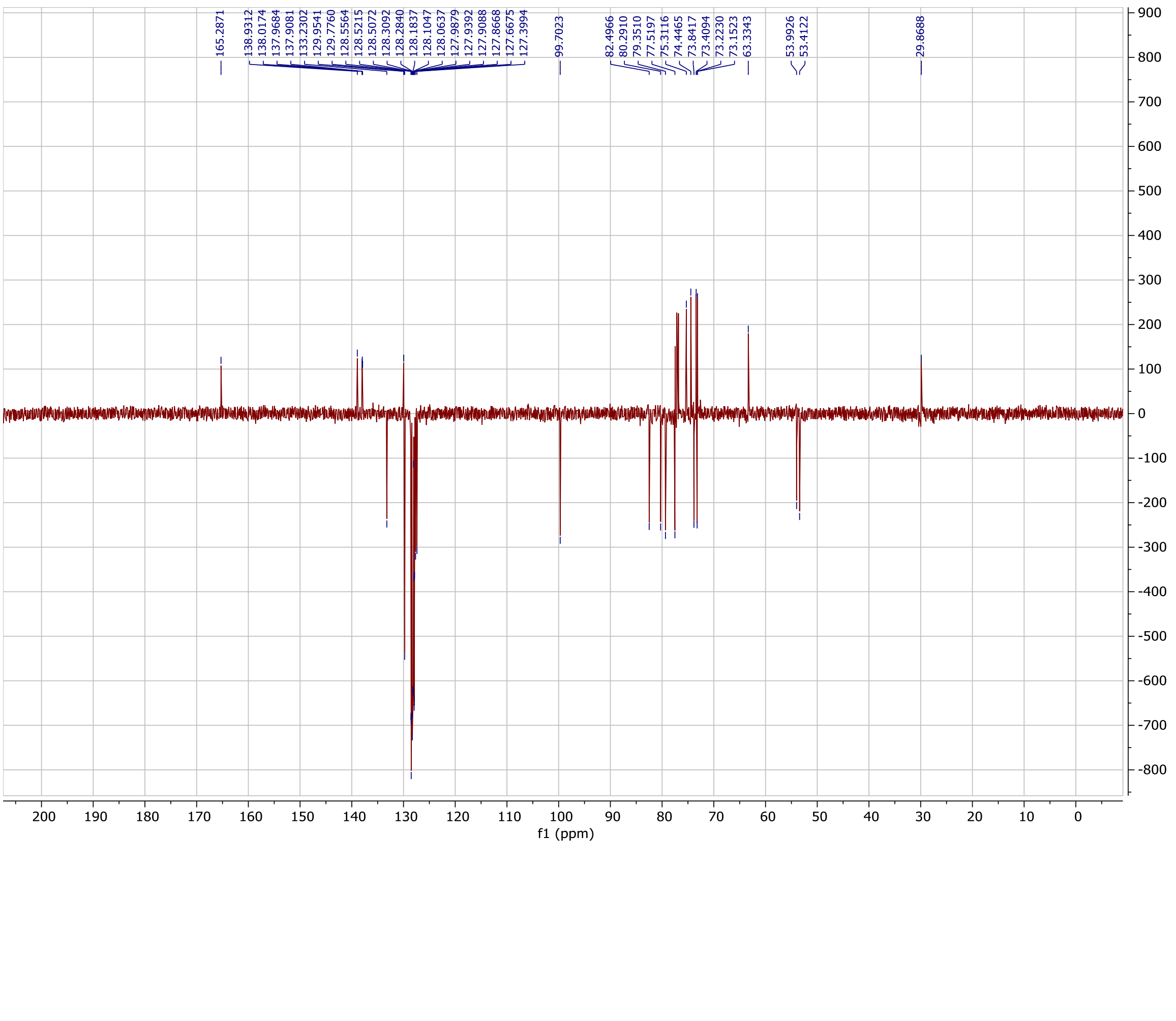

^{1}H and ^{13}C of compound **S3** in $CDCl_3$

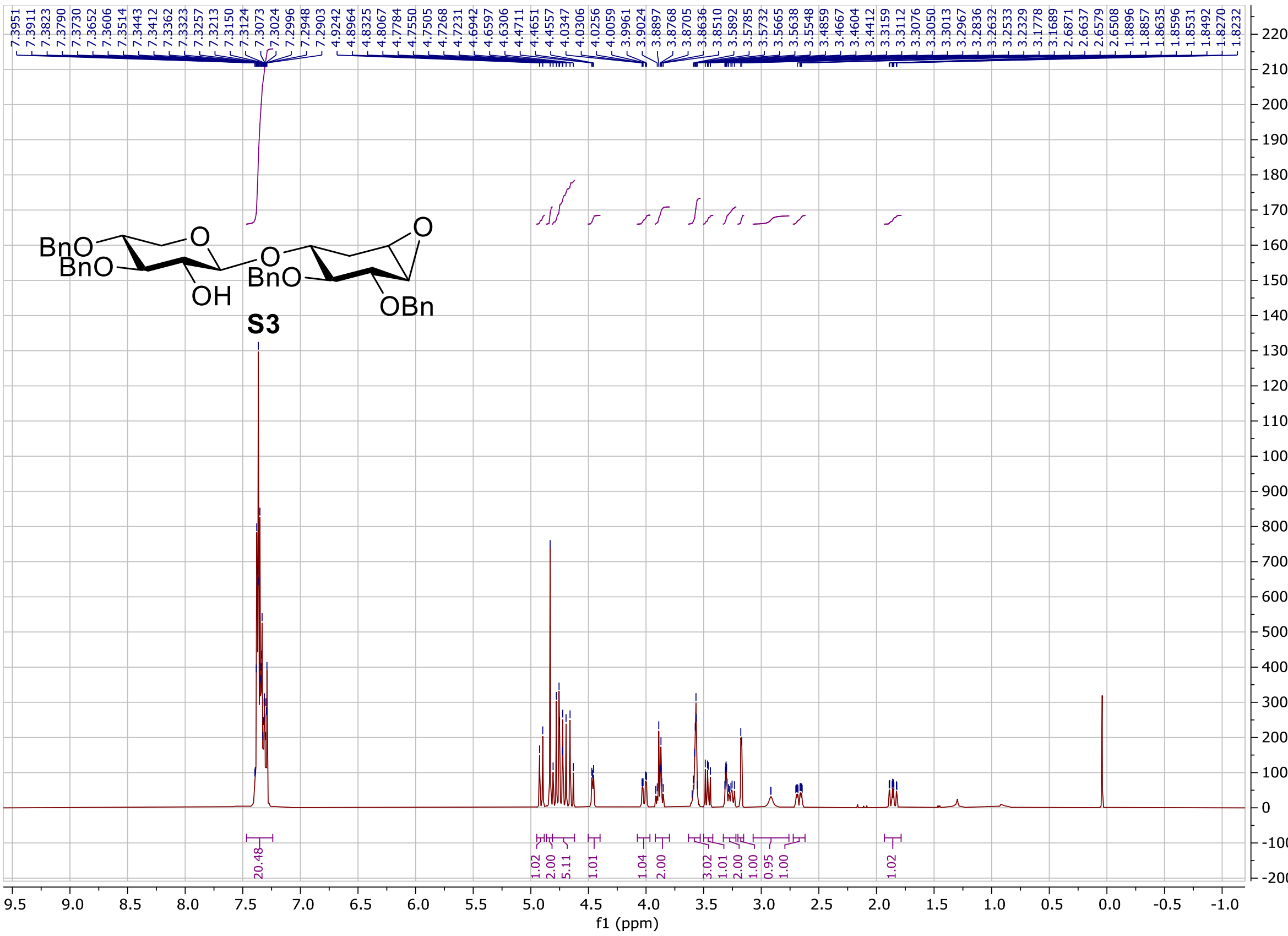

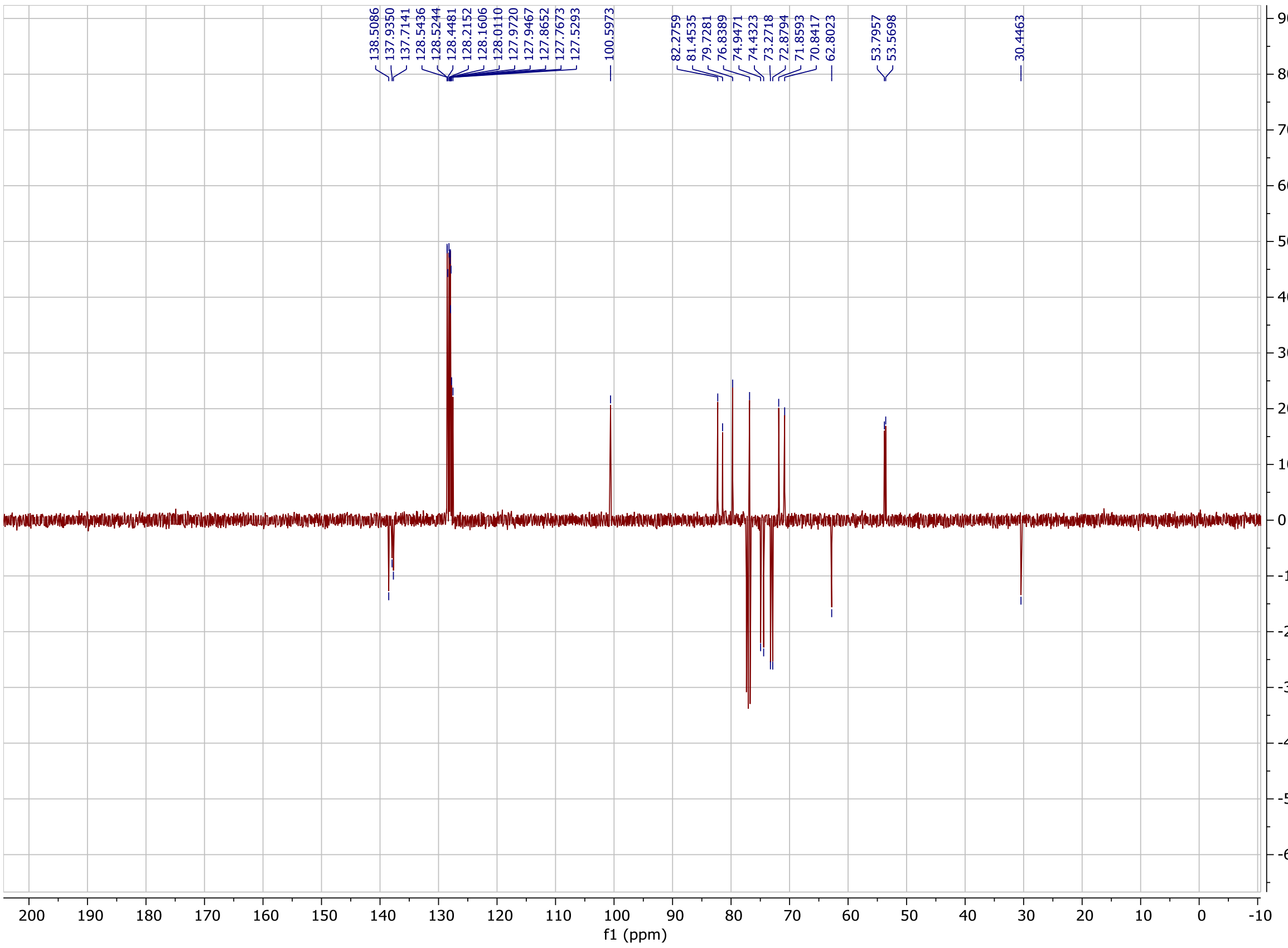

^{1}H and ^{13}C of compound **S4** in $CDCl_3$

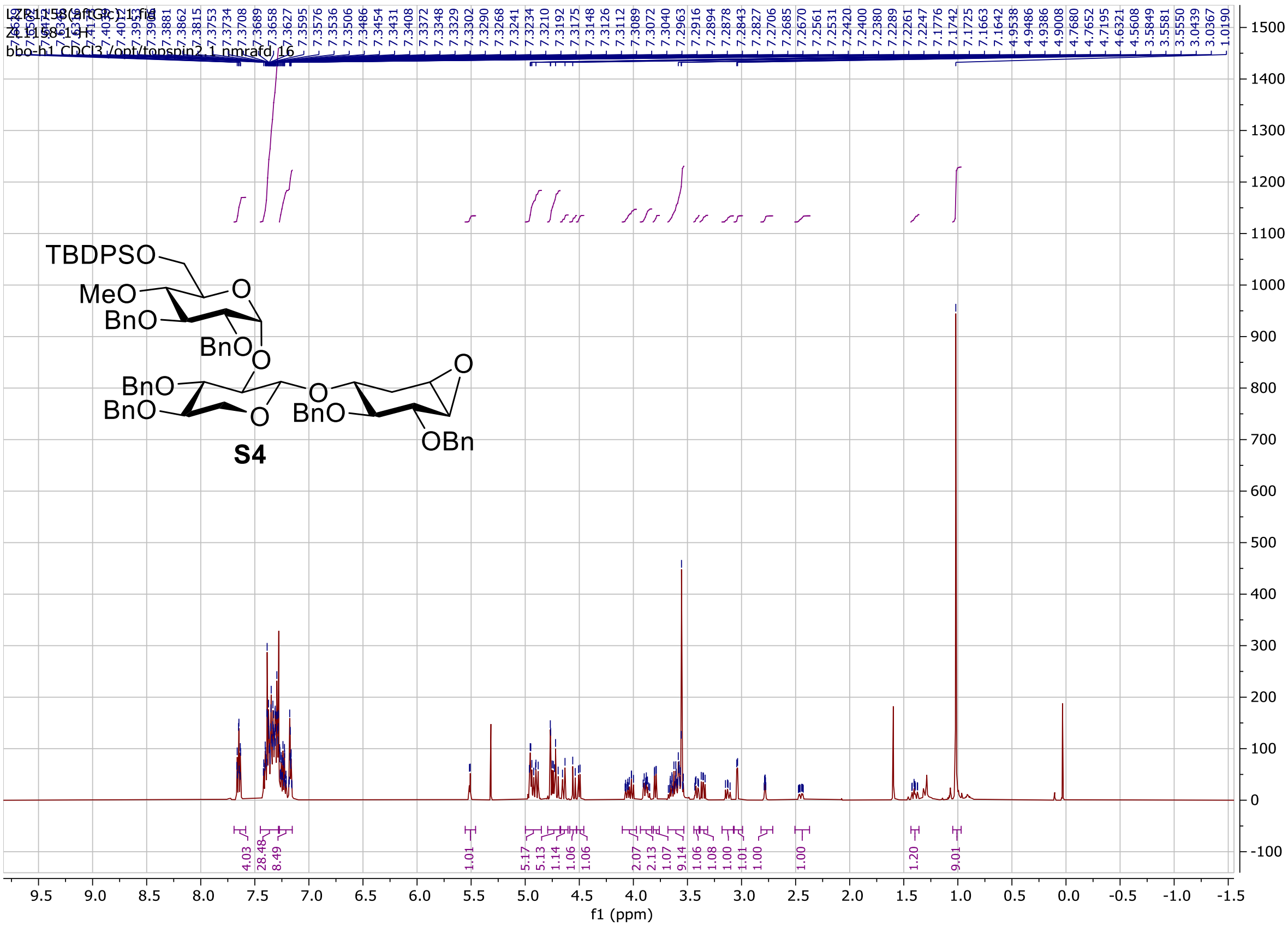

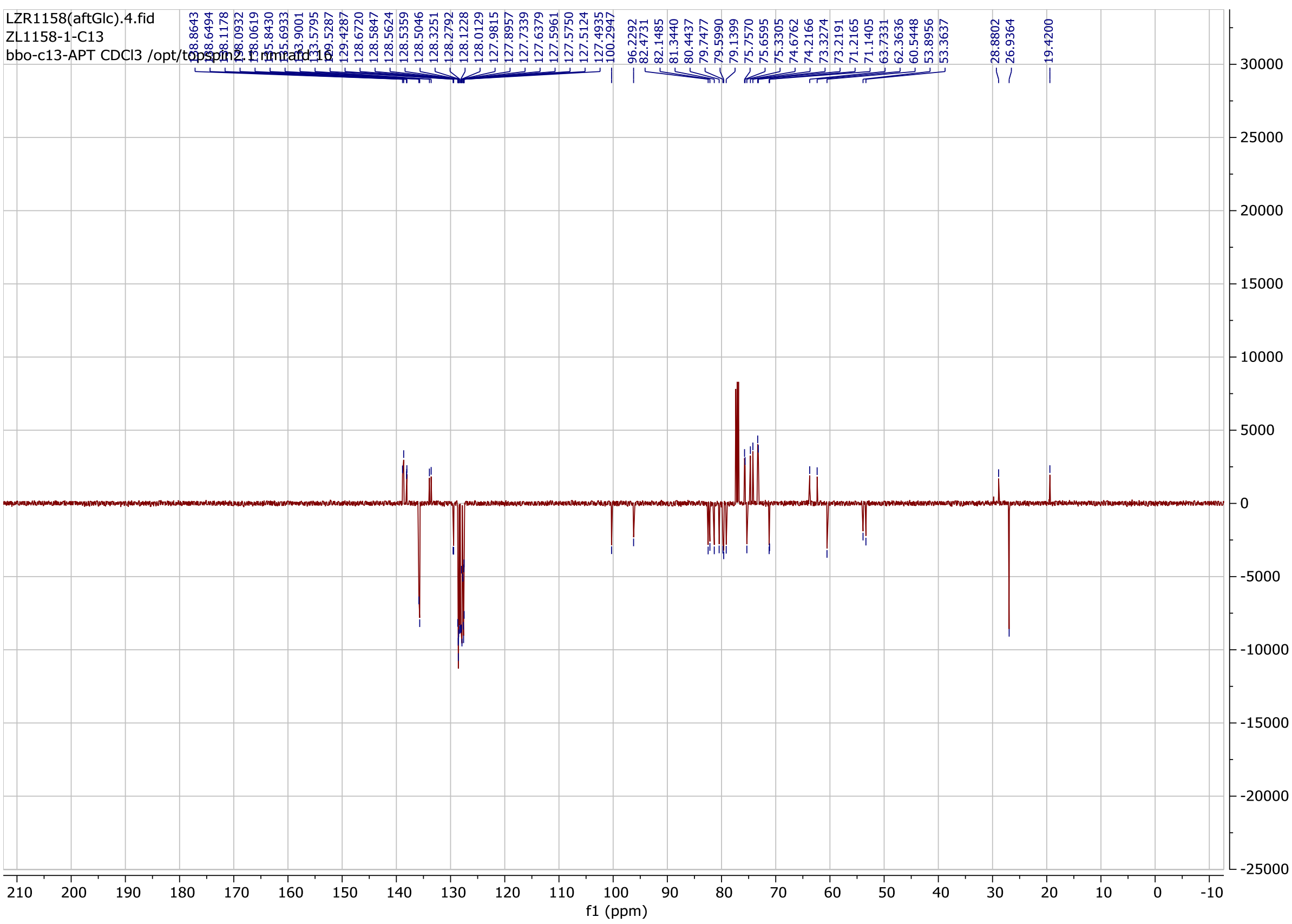

^{1}H and ^{13}C of compound **S5** in $CDCl_3$

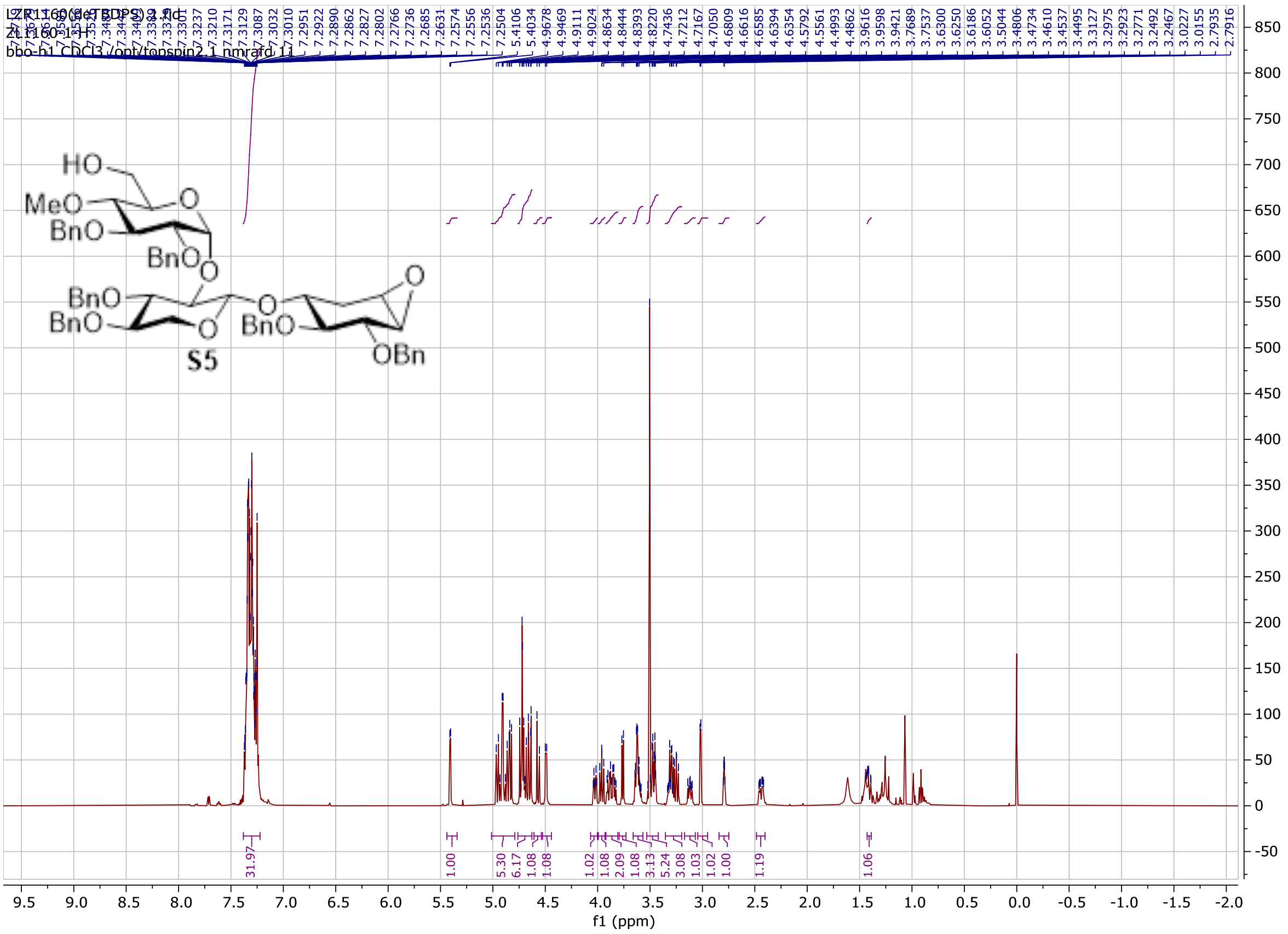

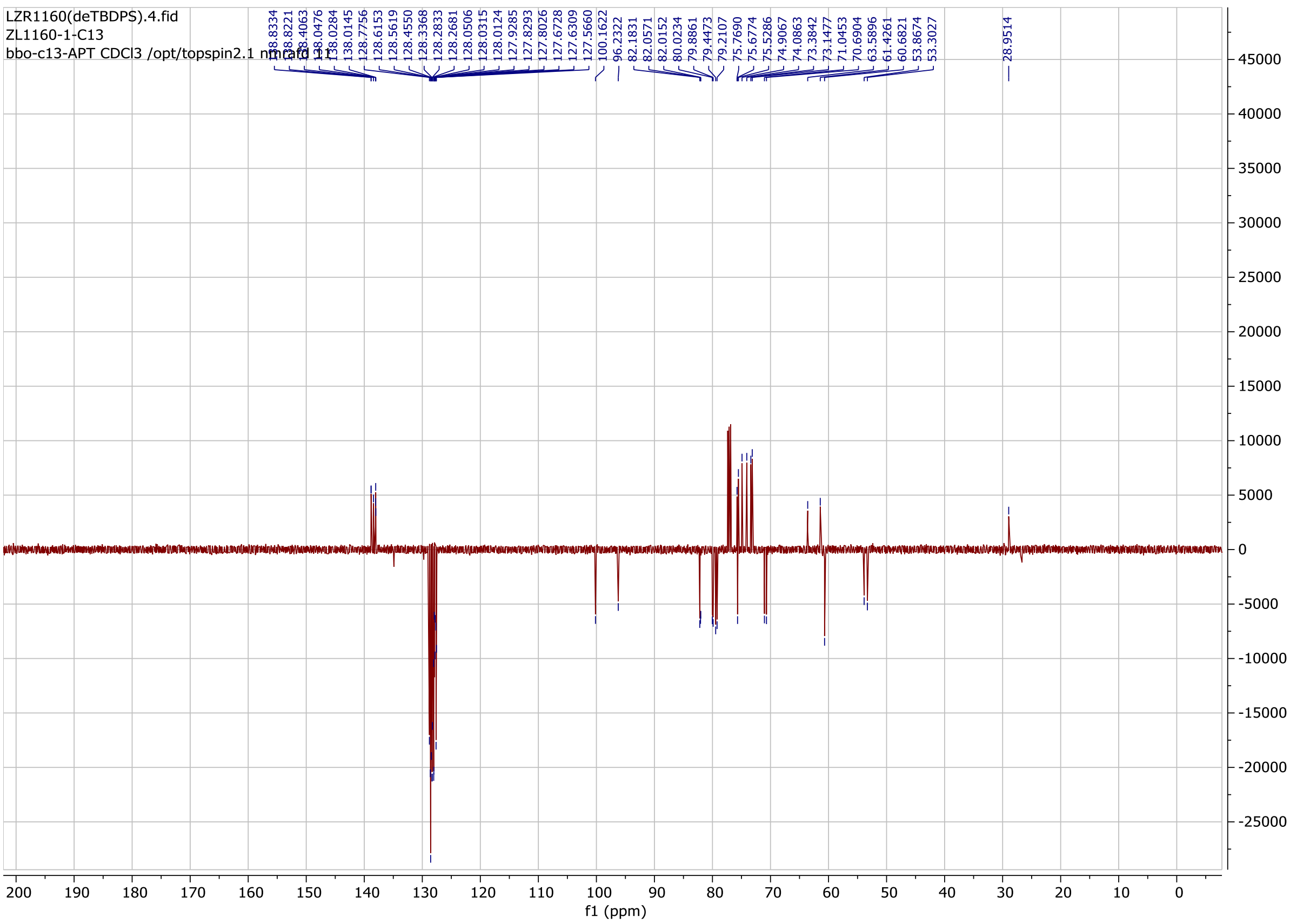

[1]H and ^{13}C of compound **S6** in $CDCl_3$

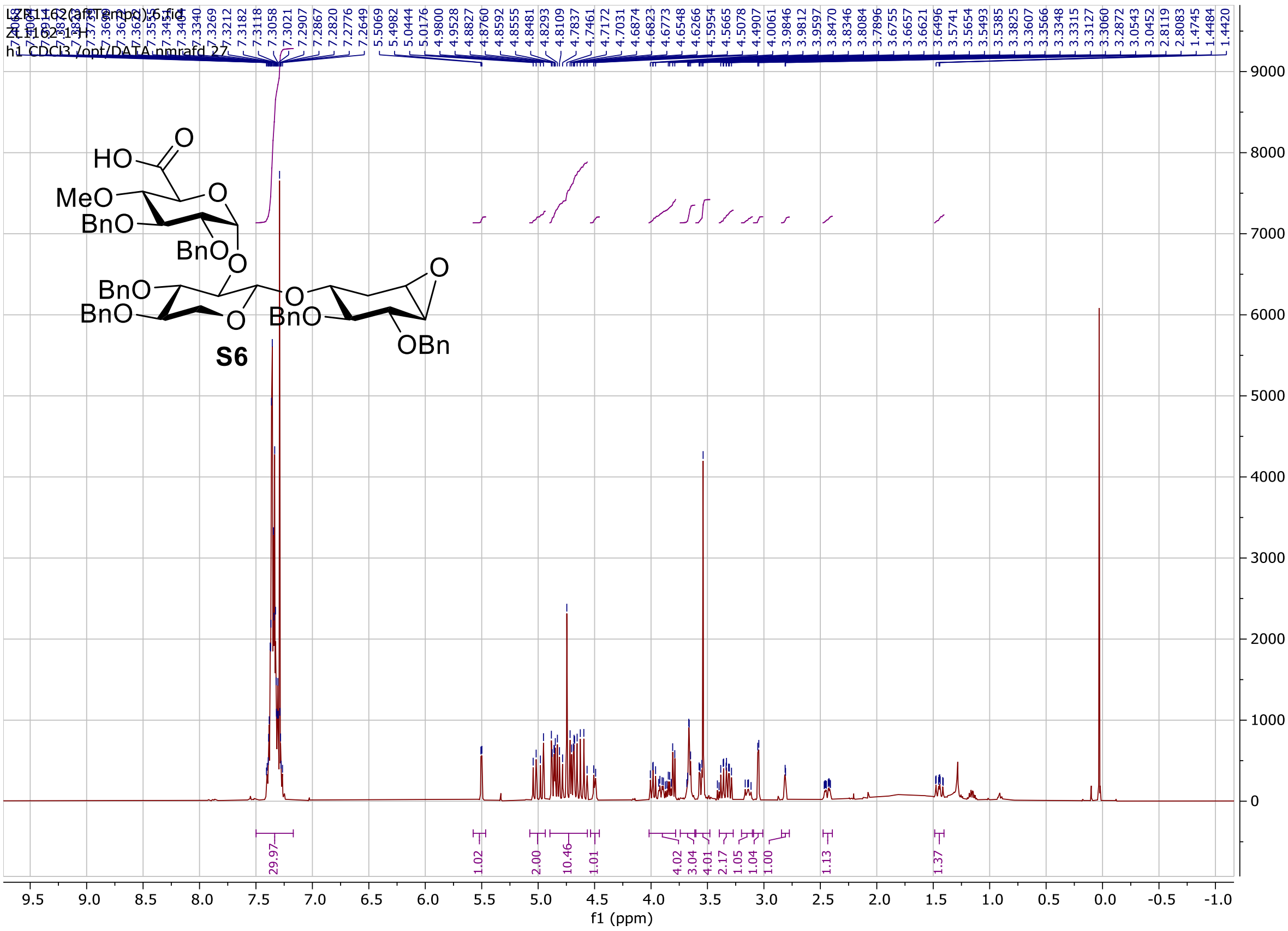

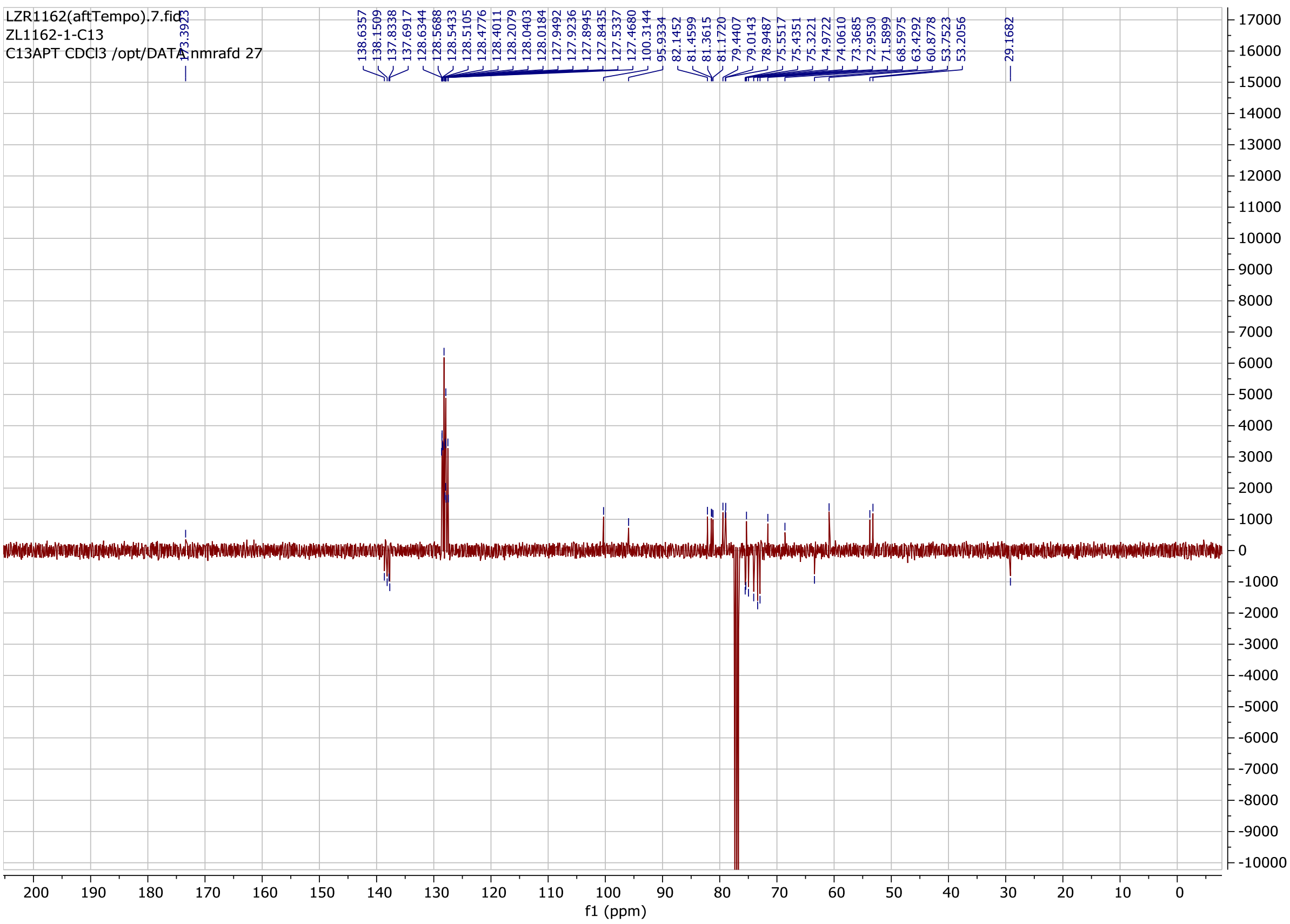

^{1}H and ^{13}C of compound **S7** in D_2O

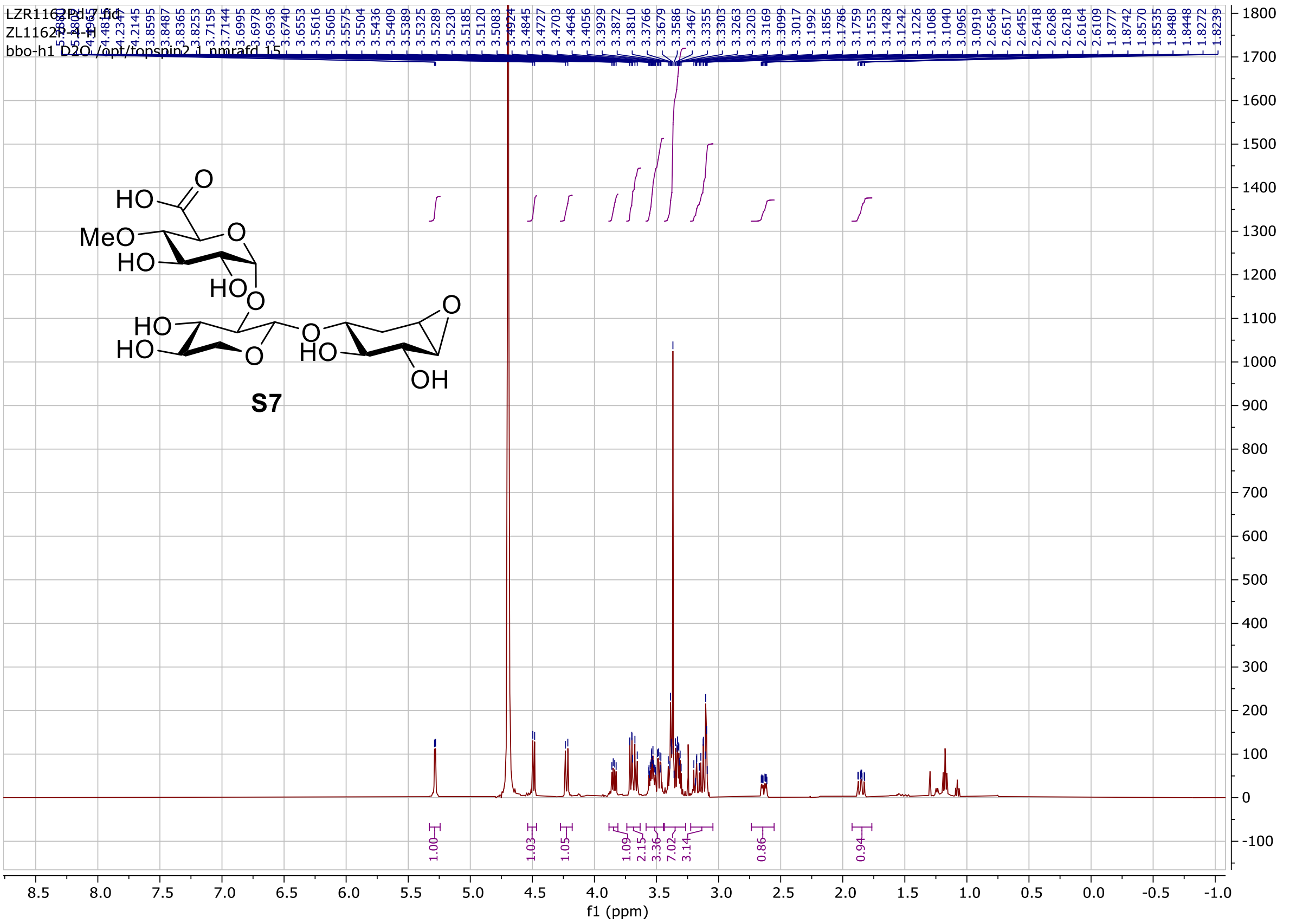

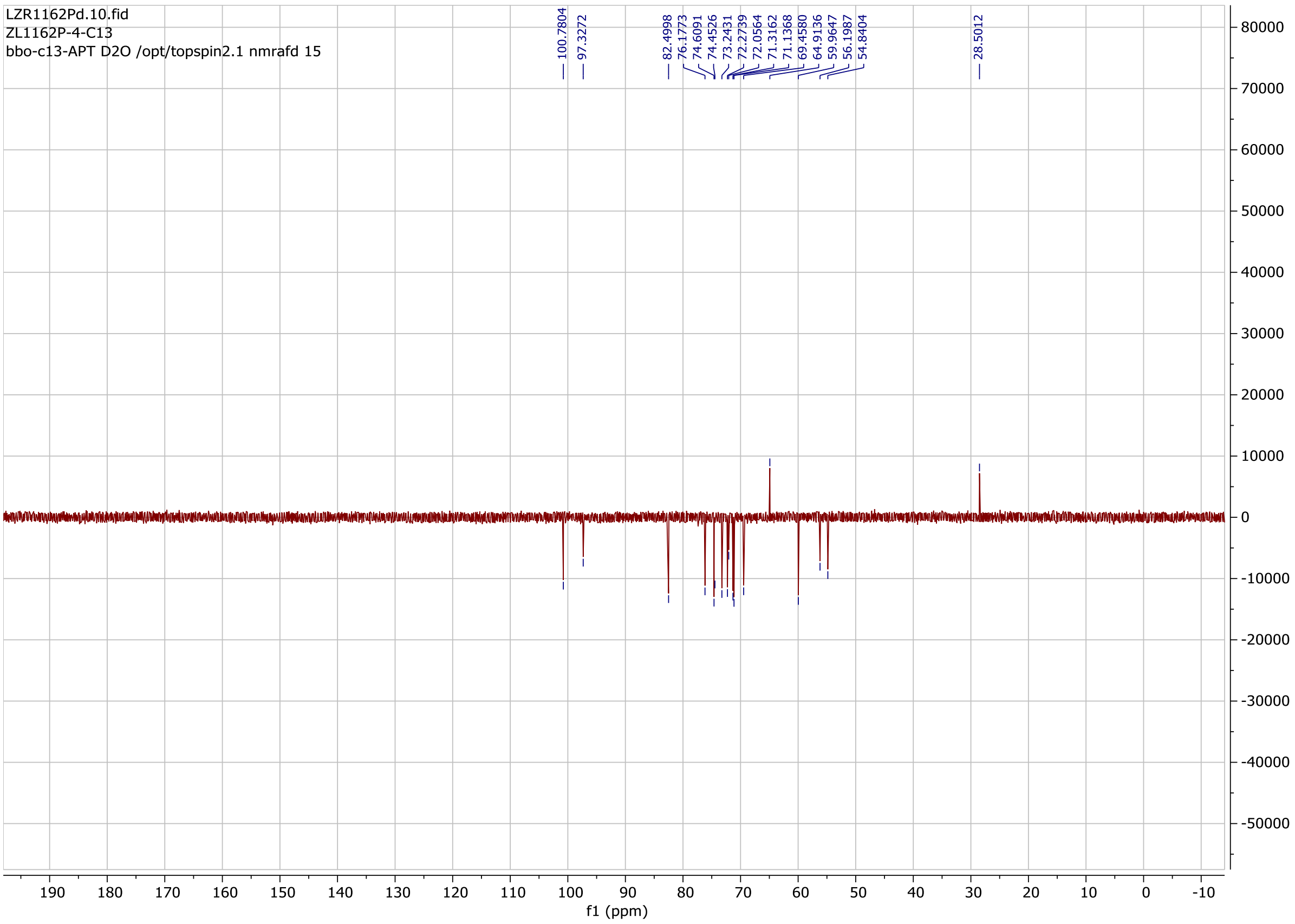

^{1}H and ^{13}C of compound **8** in $CDCl_3$

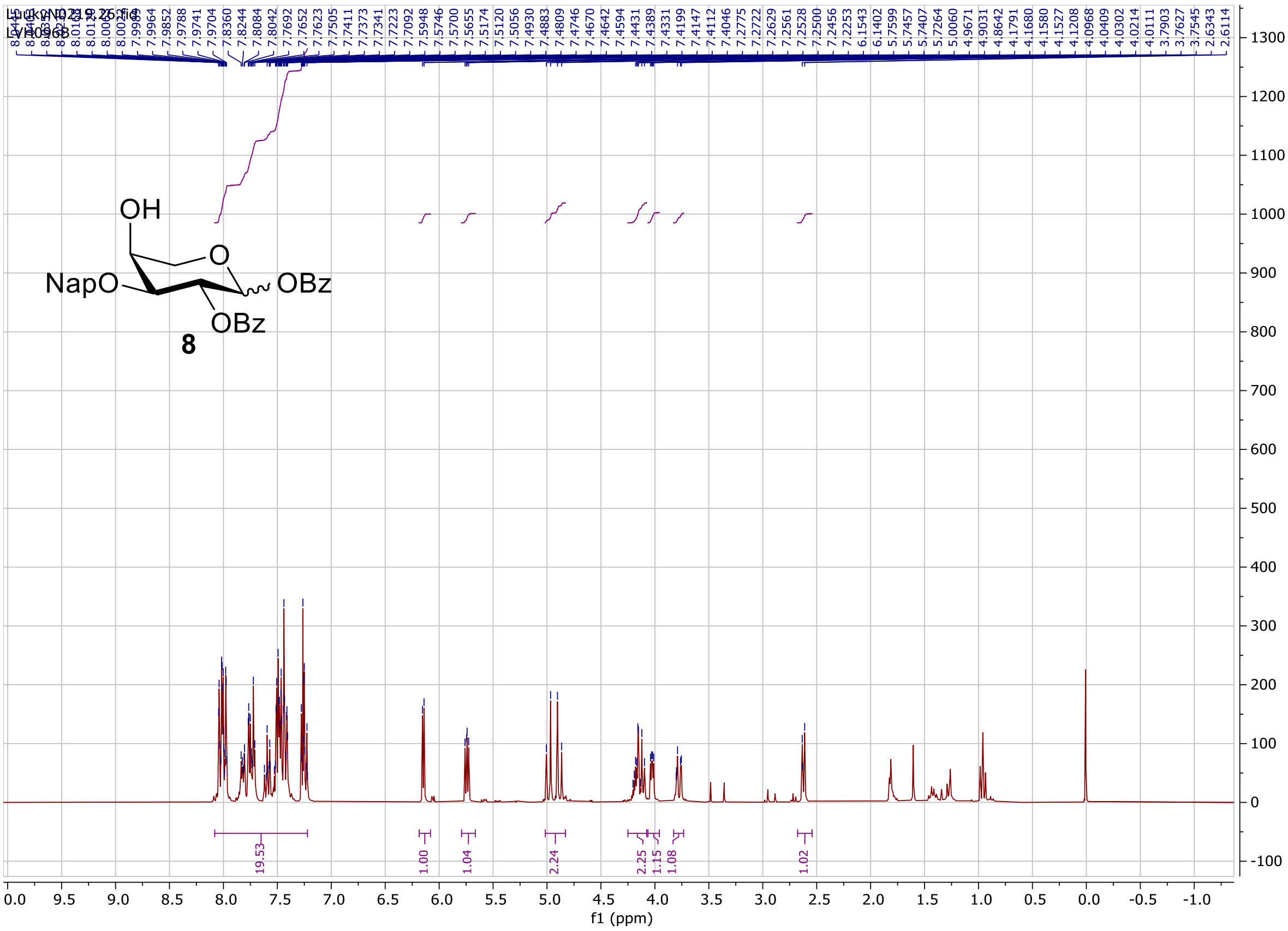

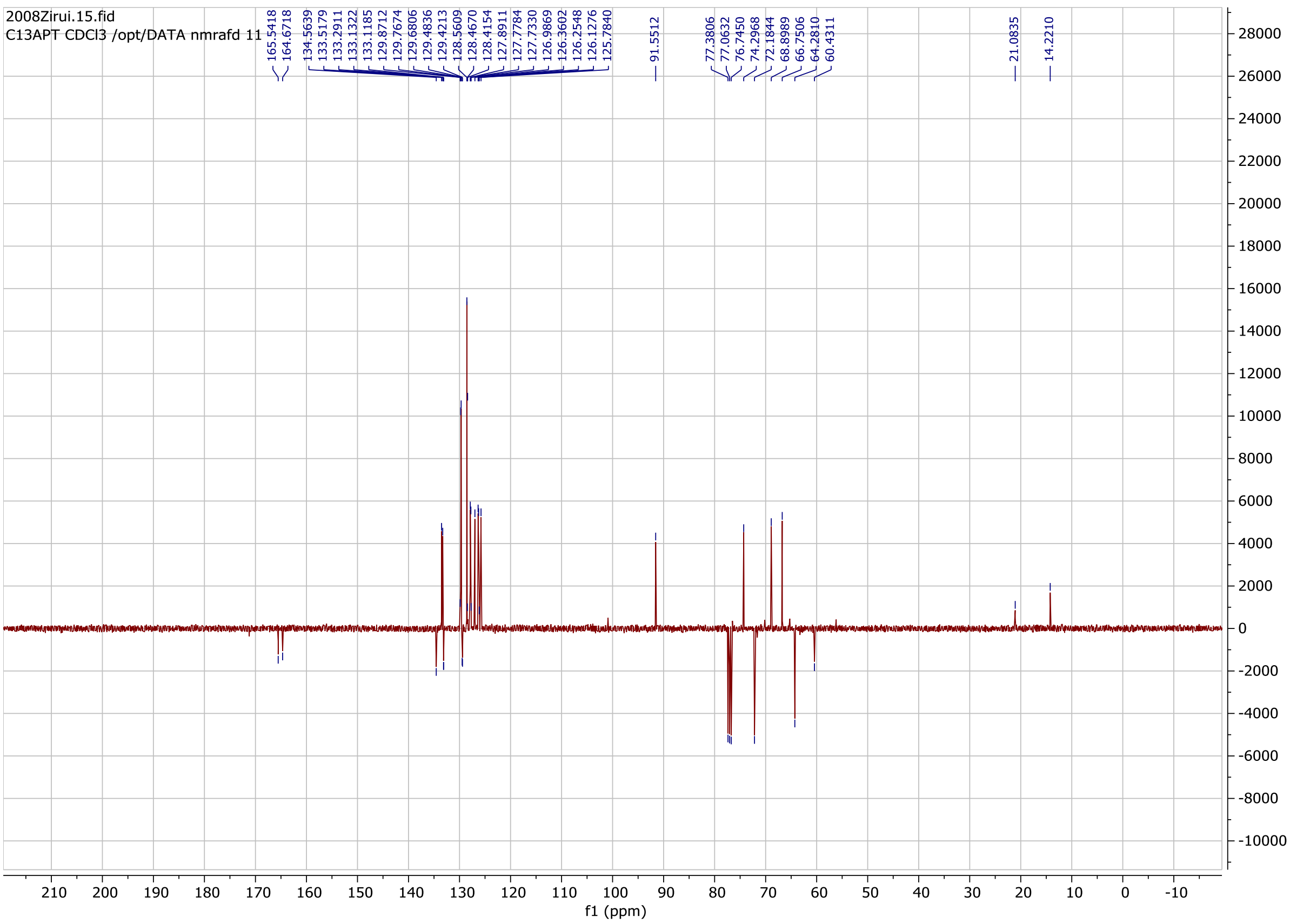

[1]H and ^{13}C of compound **9** in $CDCl_3$

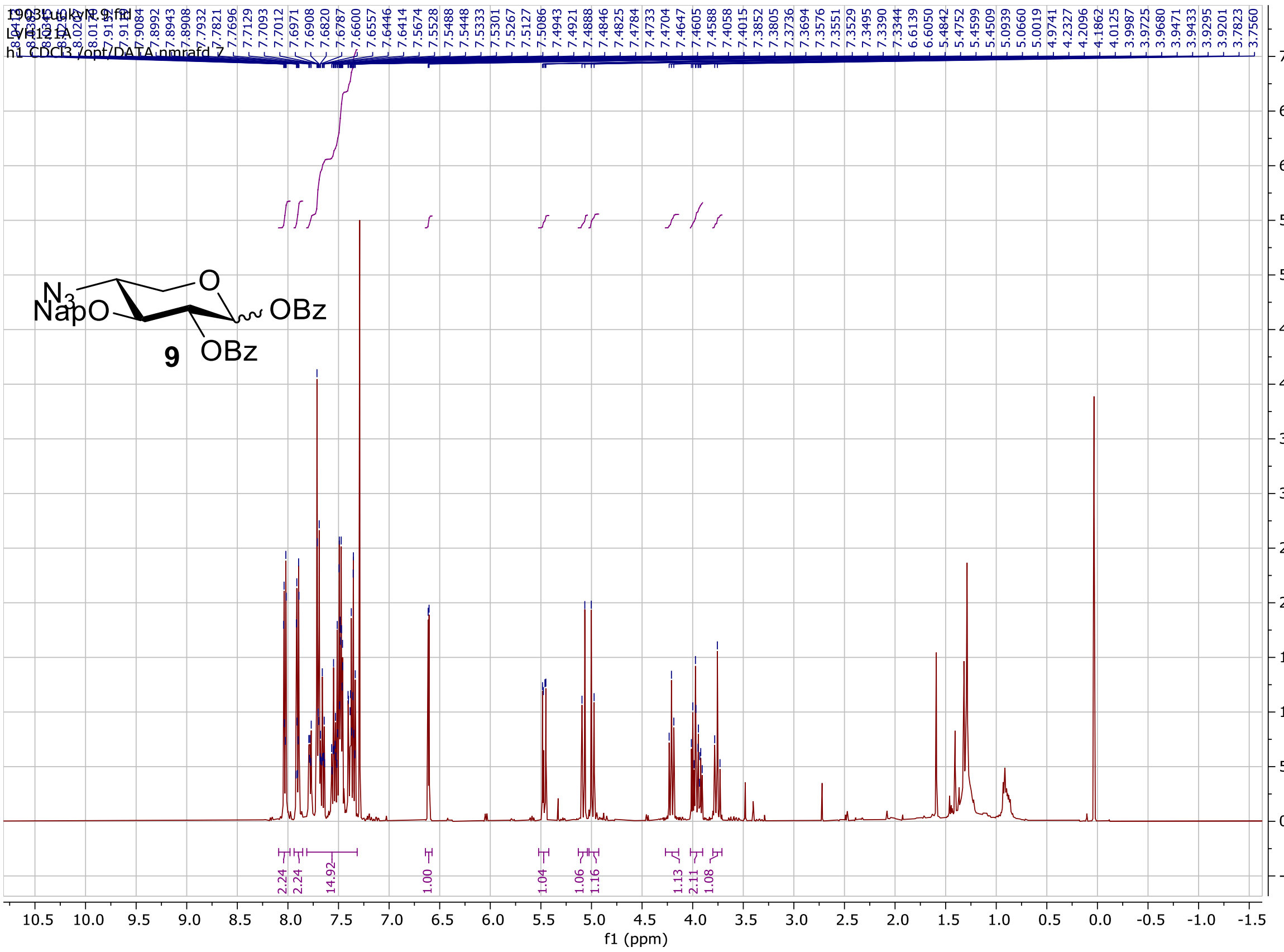

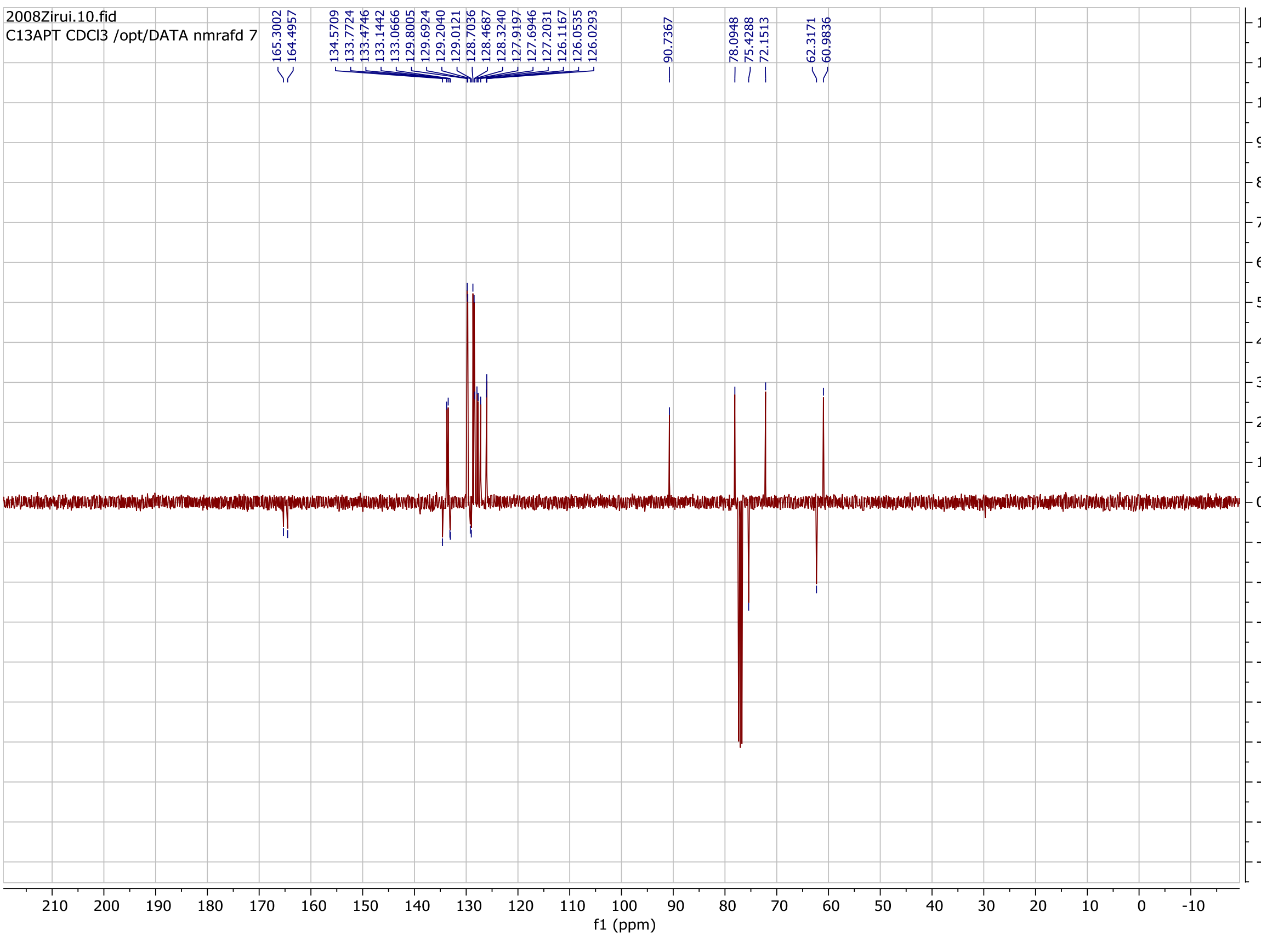

^{1}H and ^{13}C of compound **10** in $CDCl_3$

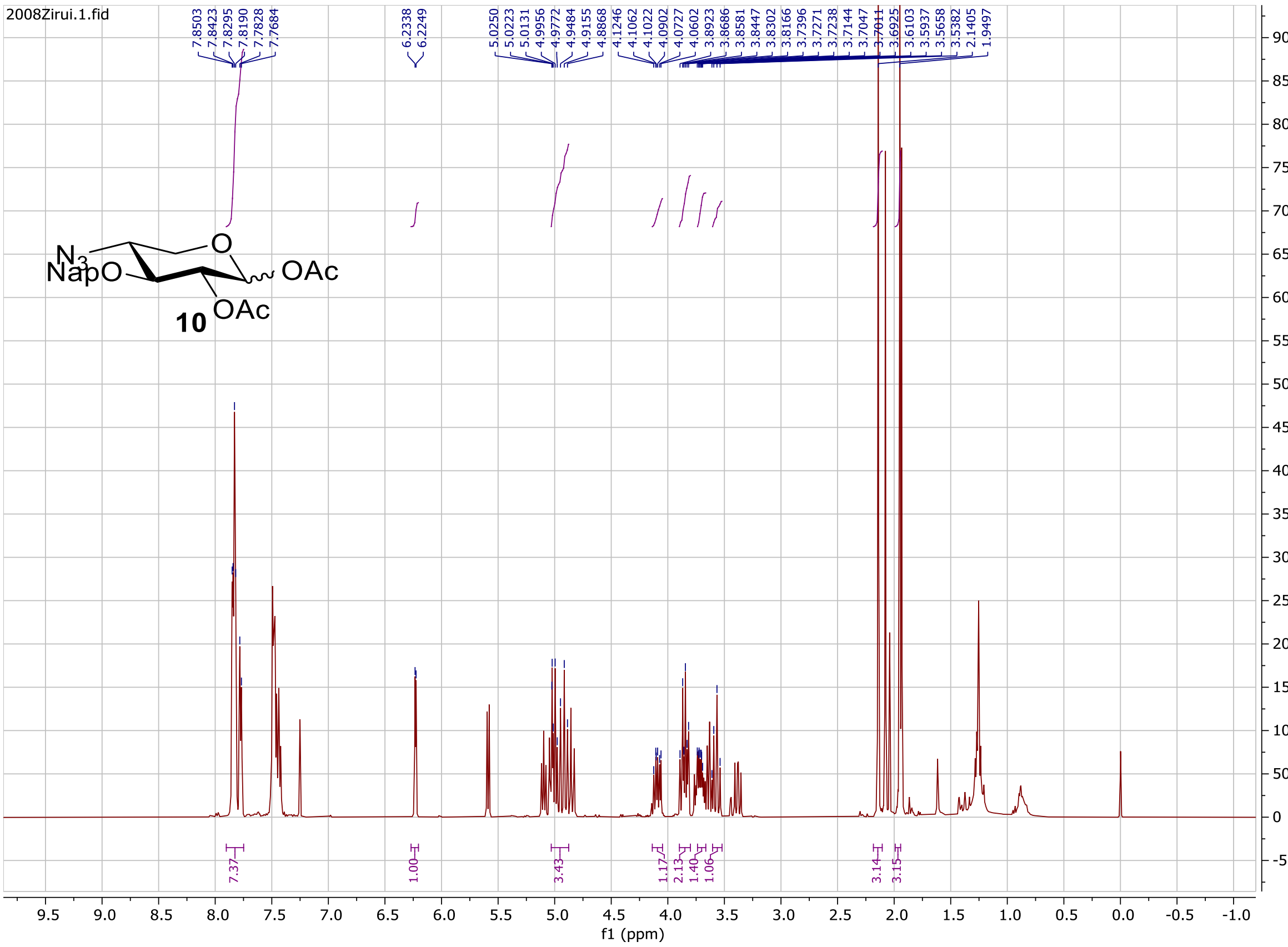

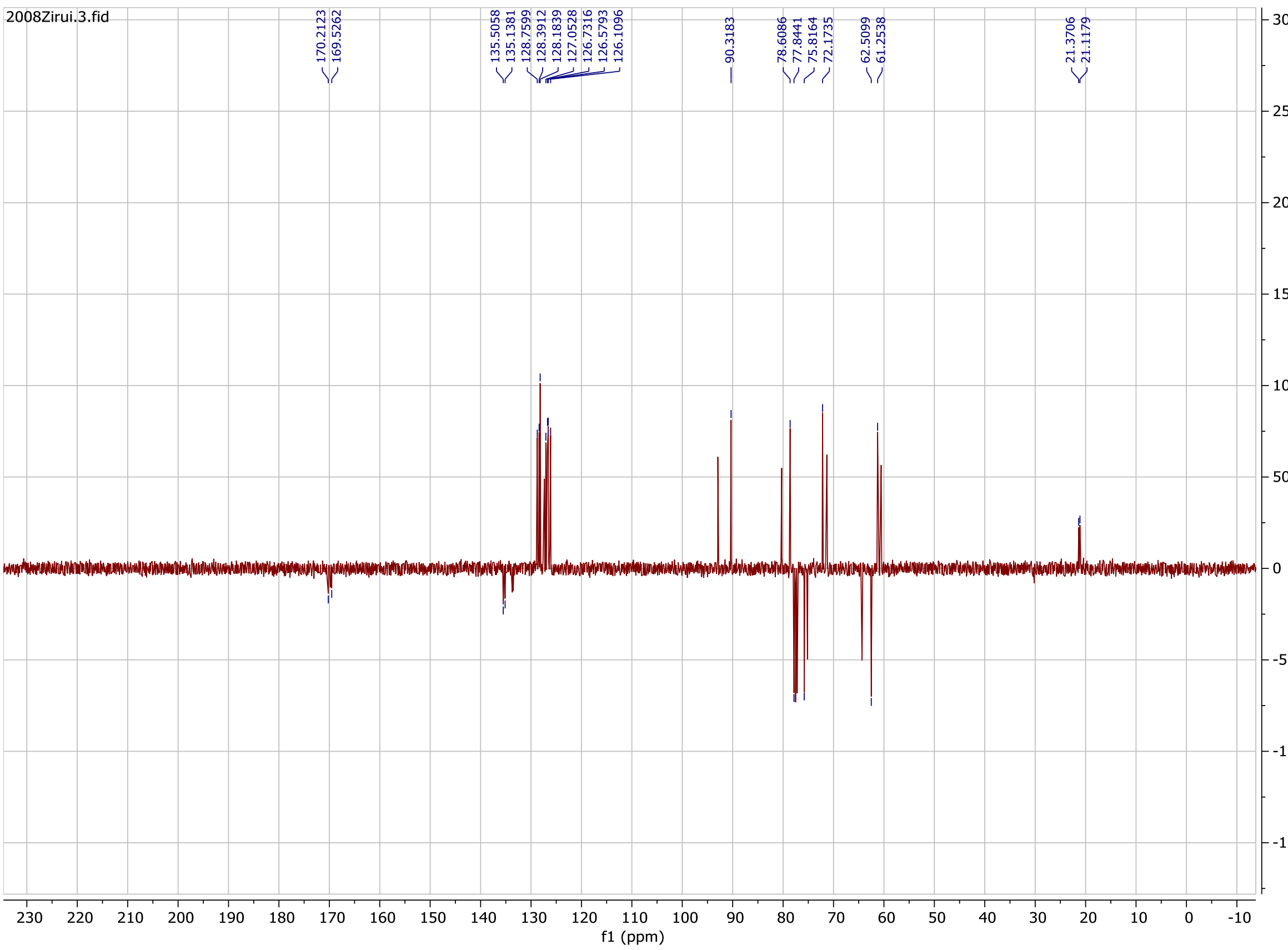

[1]H and ^{13}C of compound **11** in $CDCl_3$

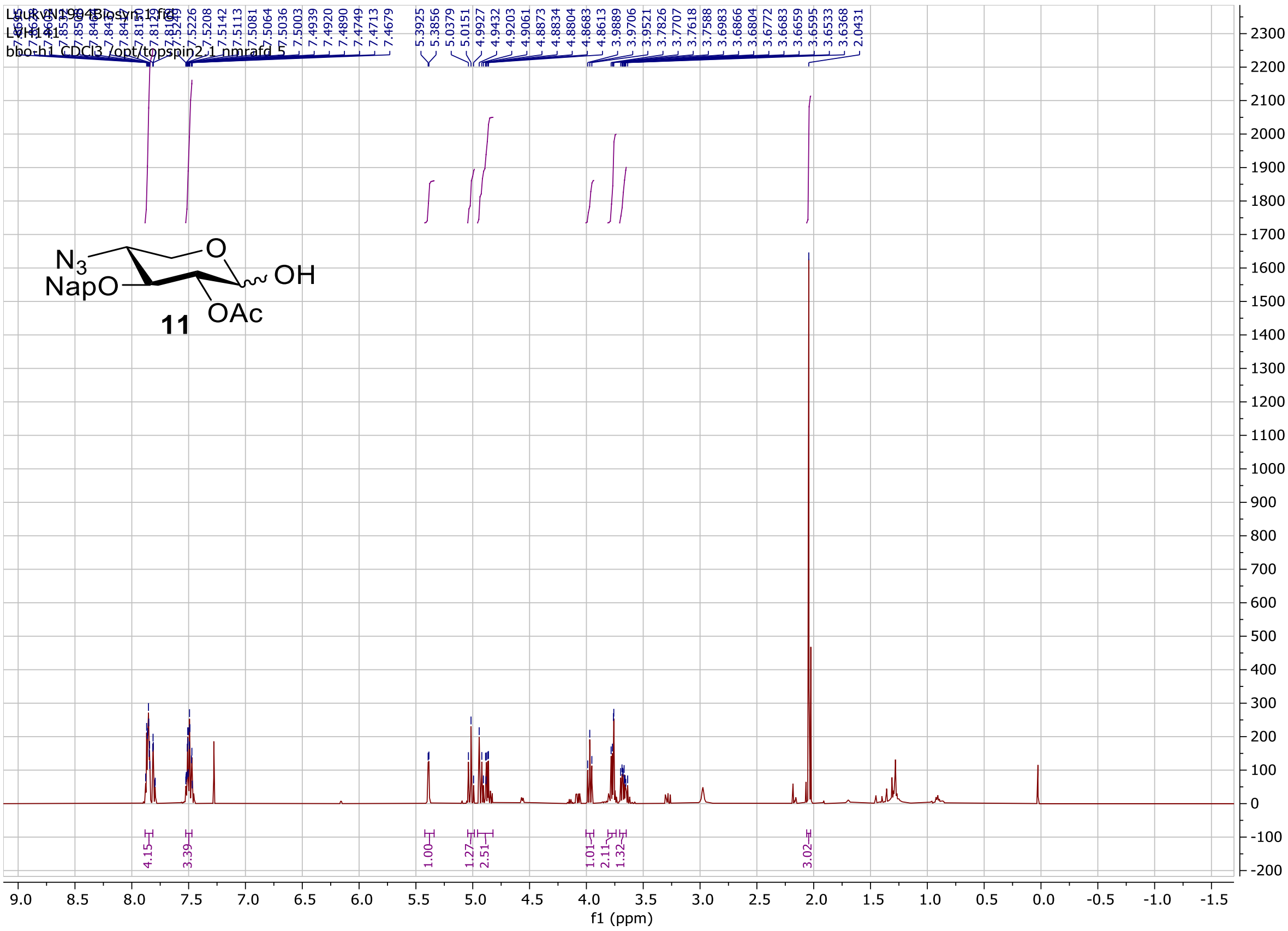

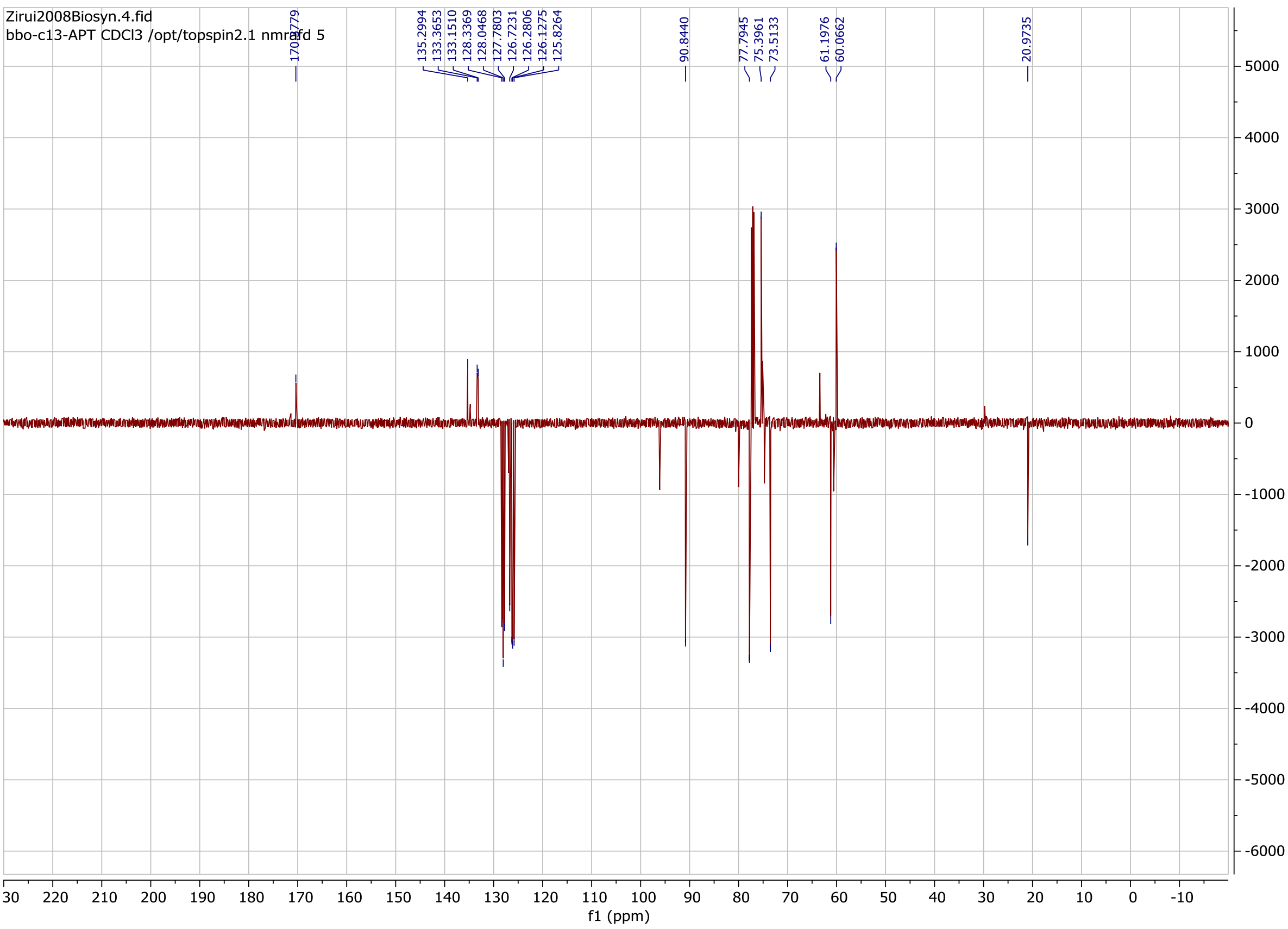

^1H and ^{13}C of compound **12** in $CDCl_3$

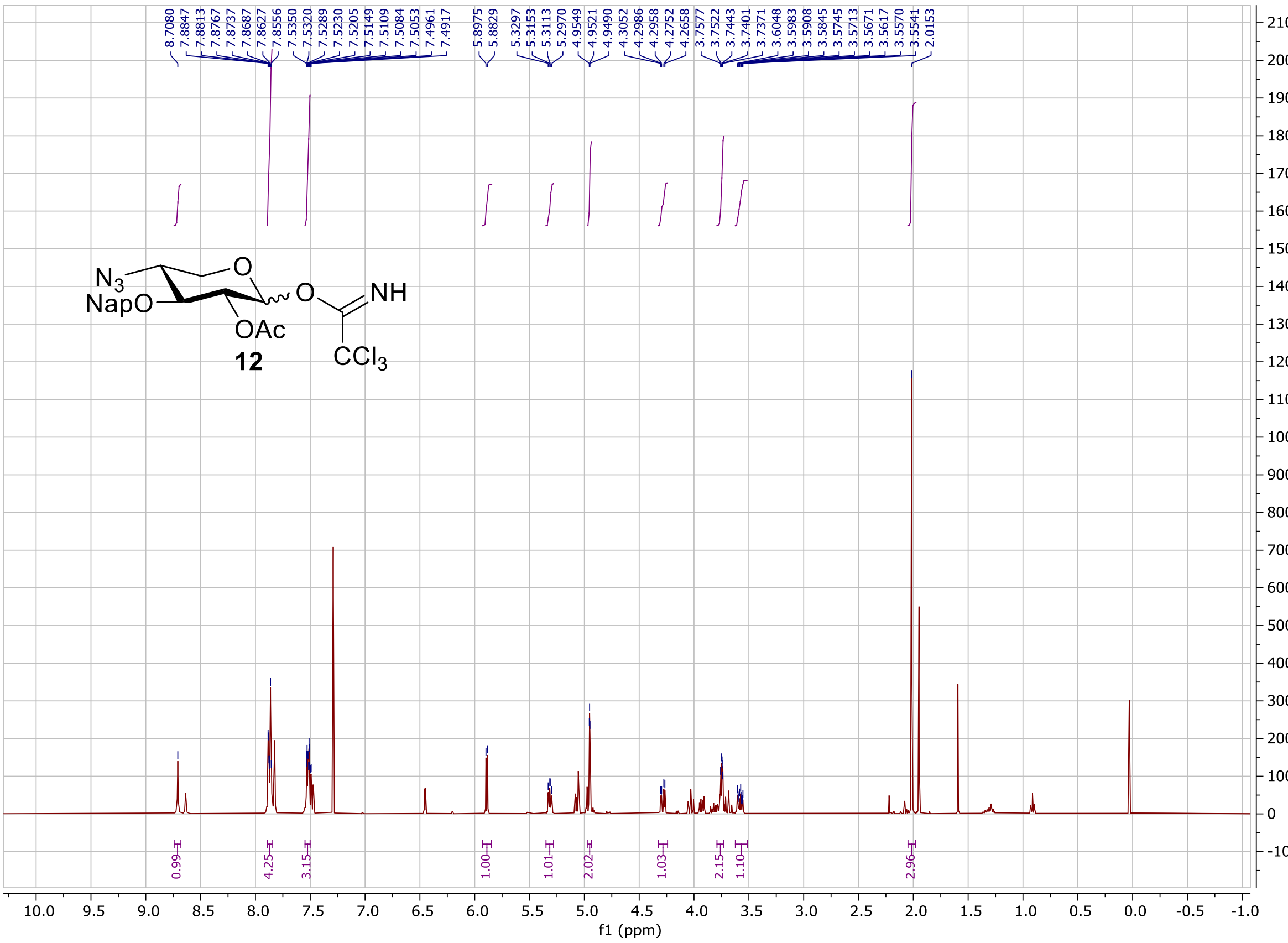

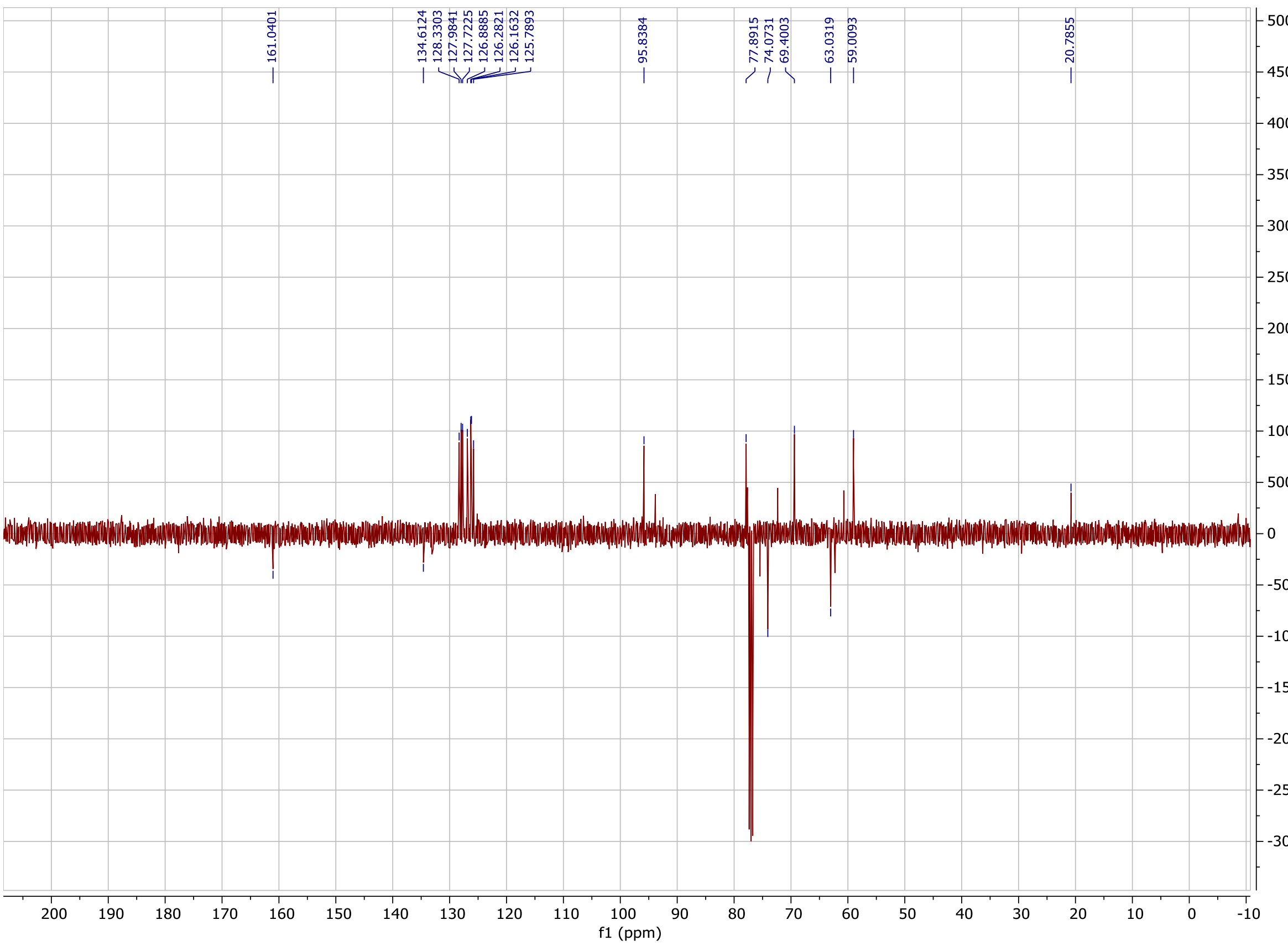

[1]H and ^{13}C of compound **14** in $CDCl_3$

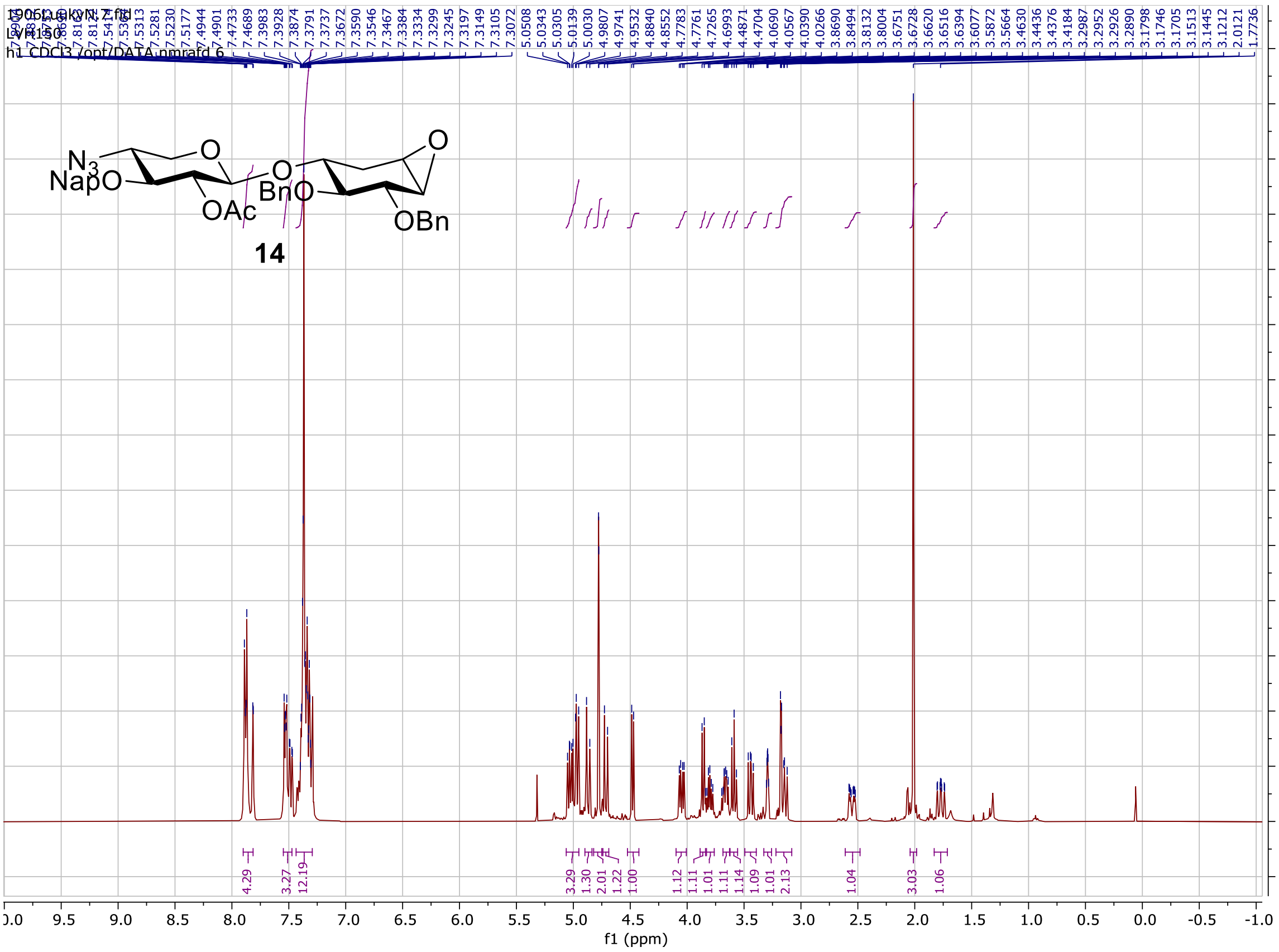

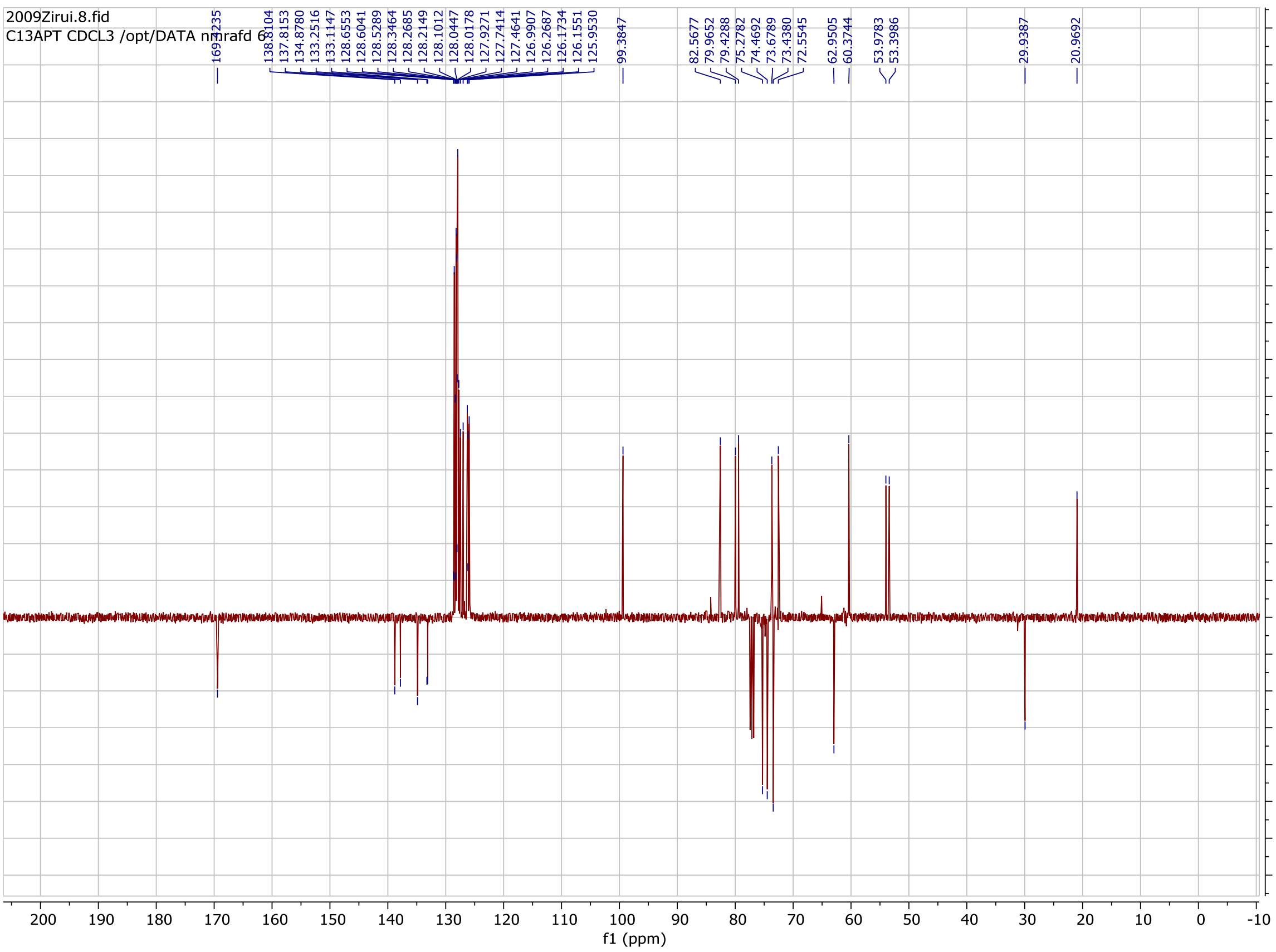

[1]H and ^{13}C of compound **15** in $CDCl_3$

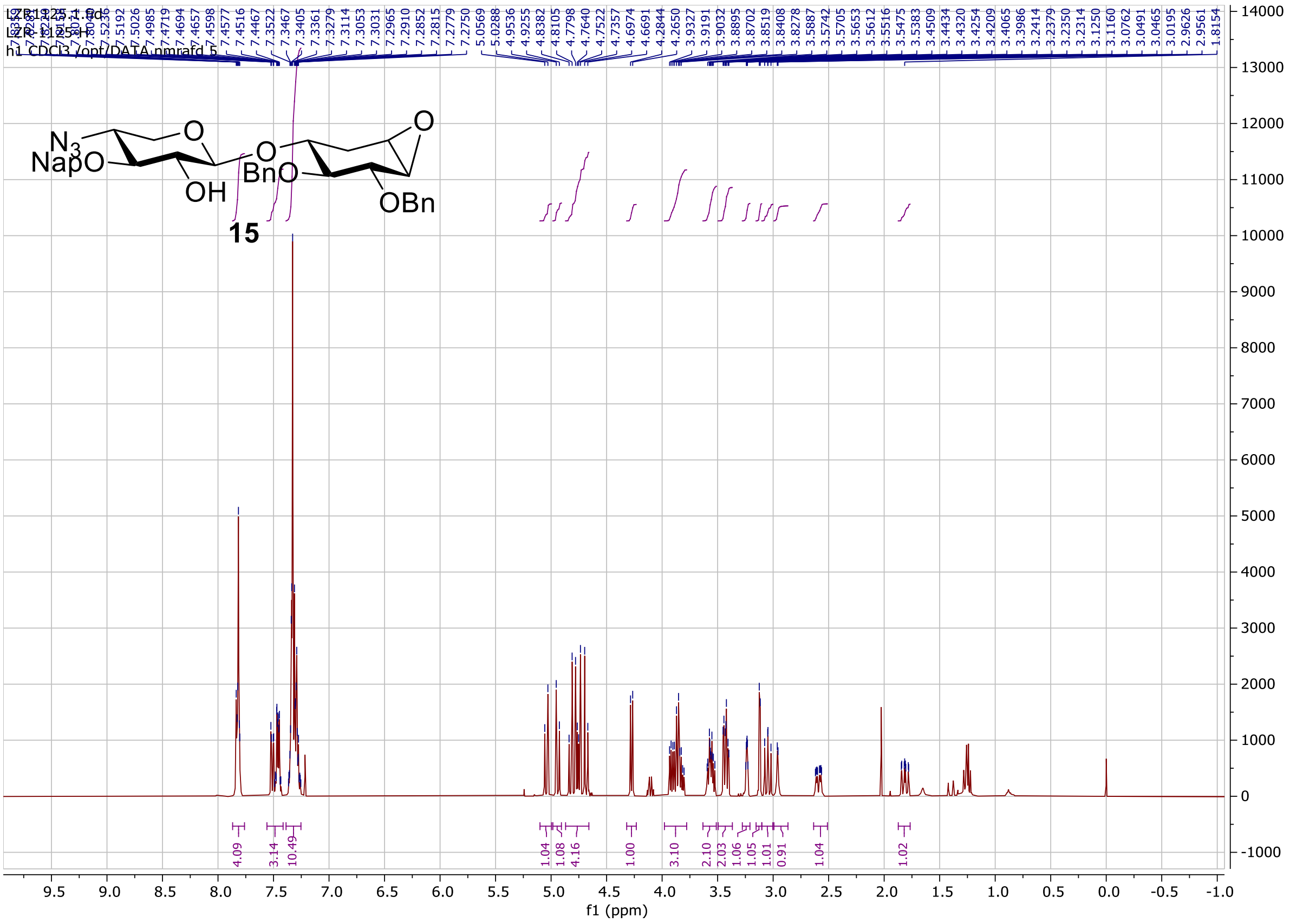

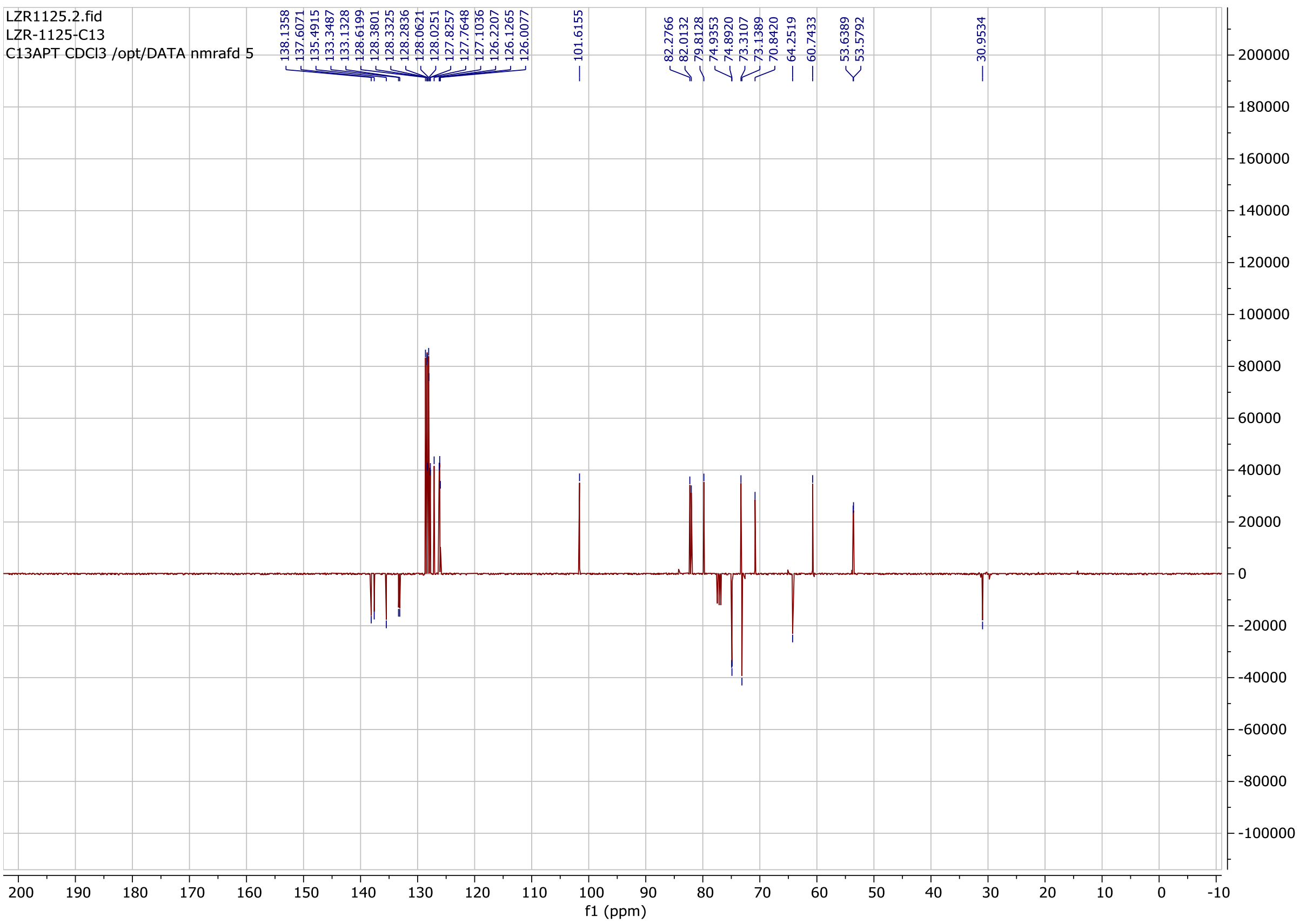

[1]H and ^{13}C of compound **17** in $CDCl_3$

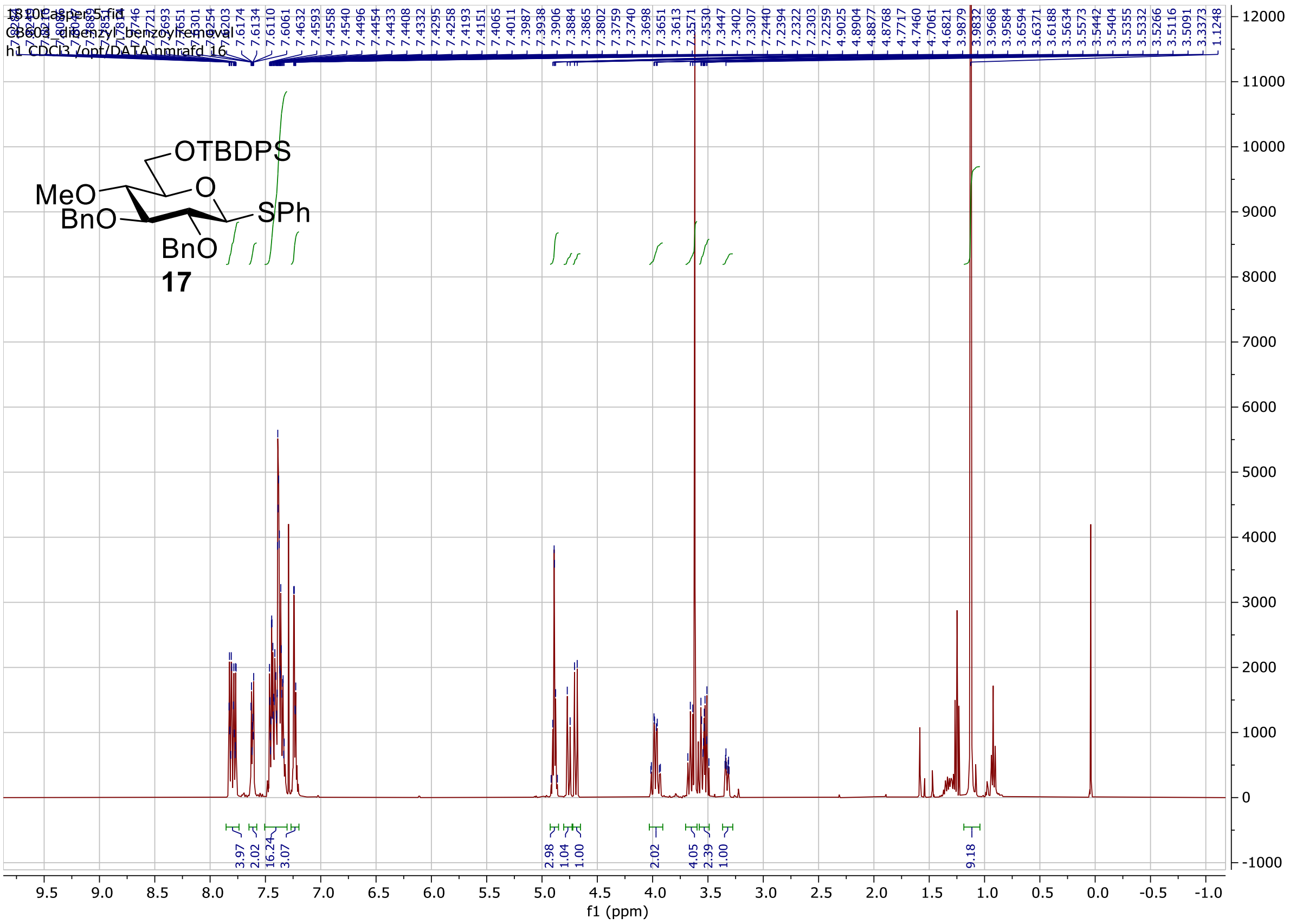

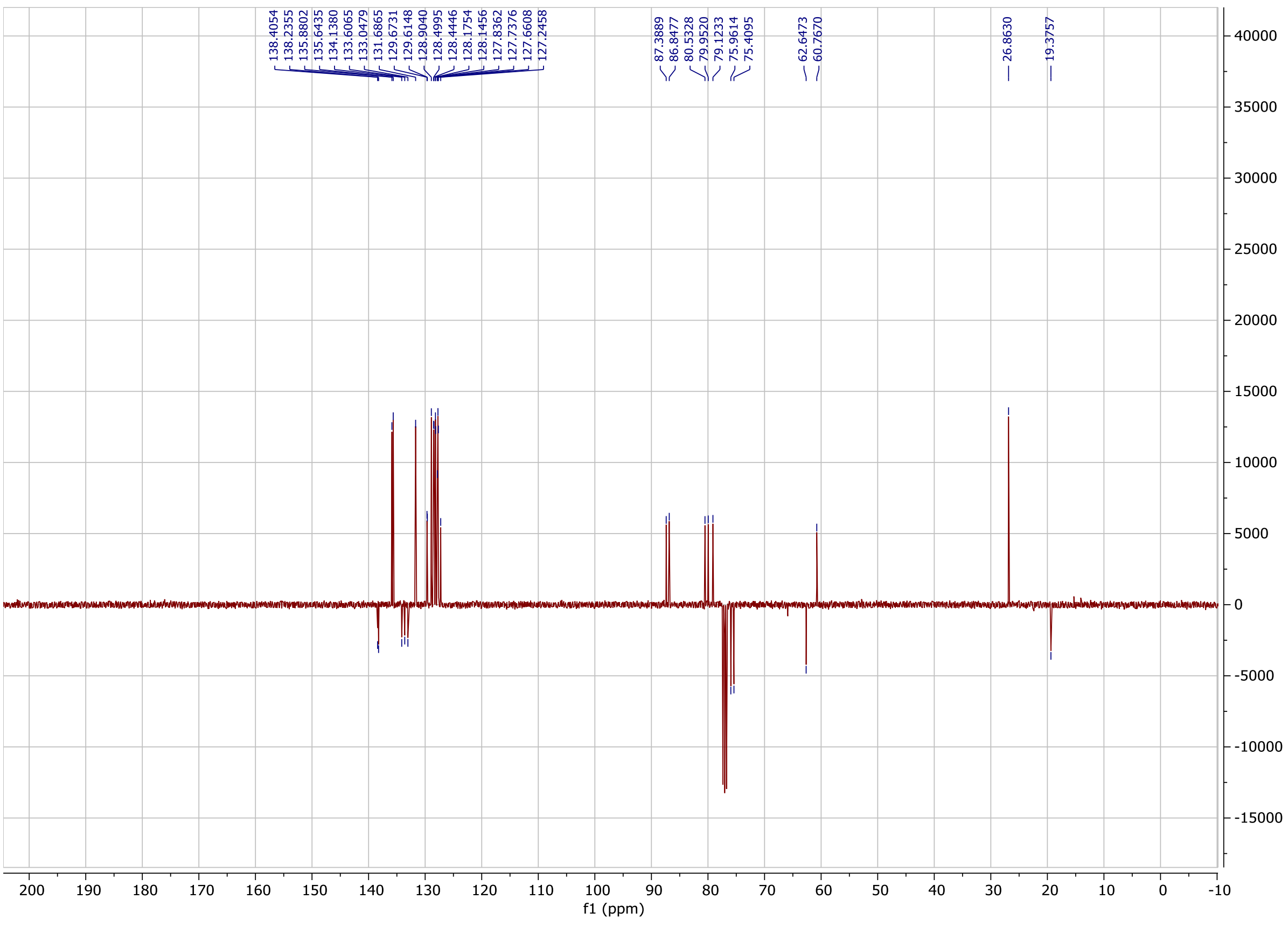

[1]H and ^{13}C of compound **18** in $CDCl_3$

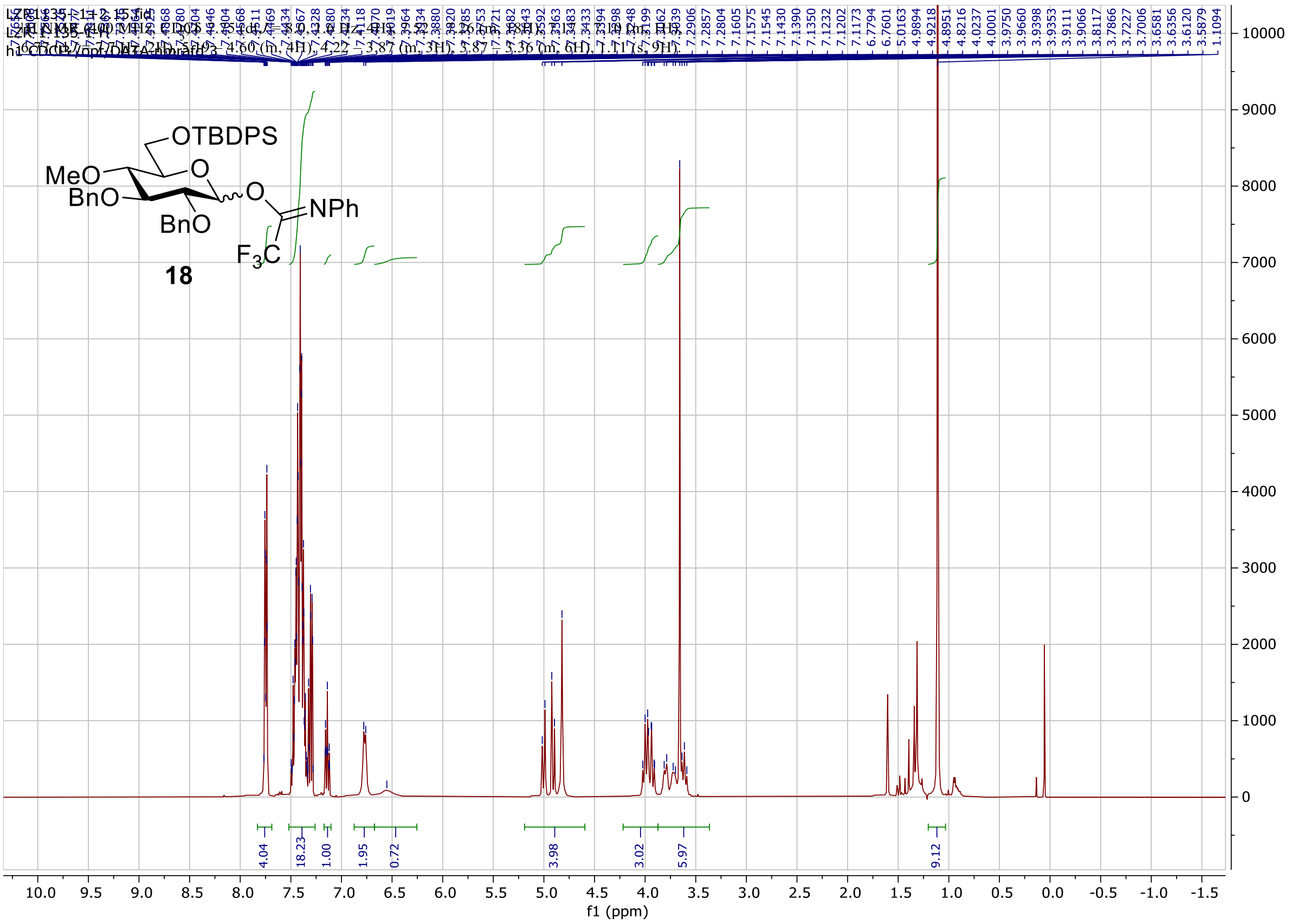

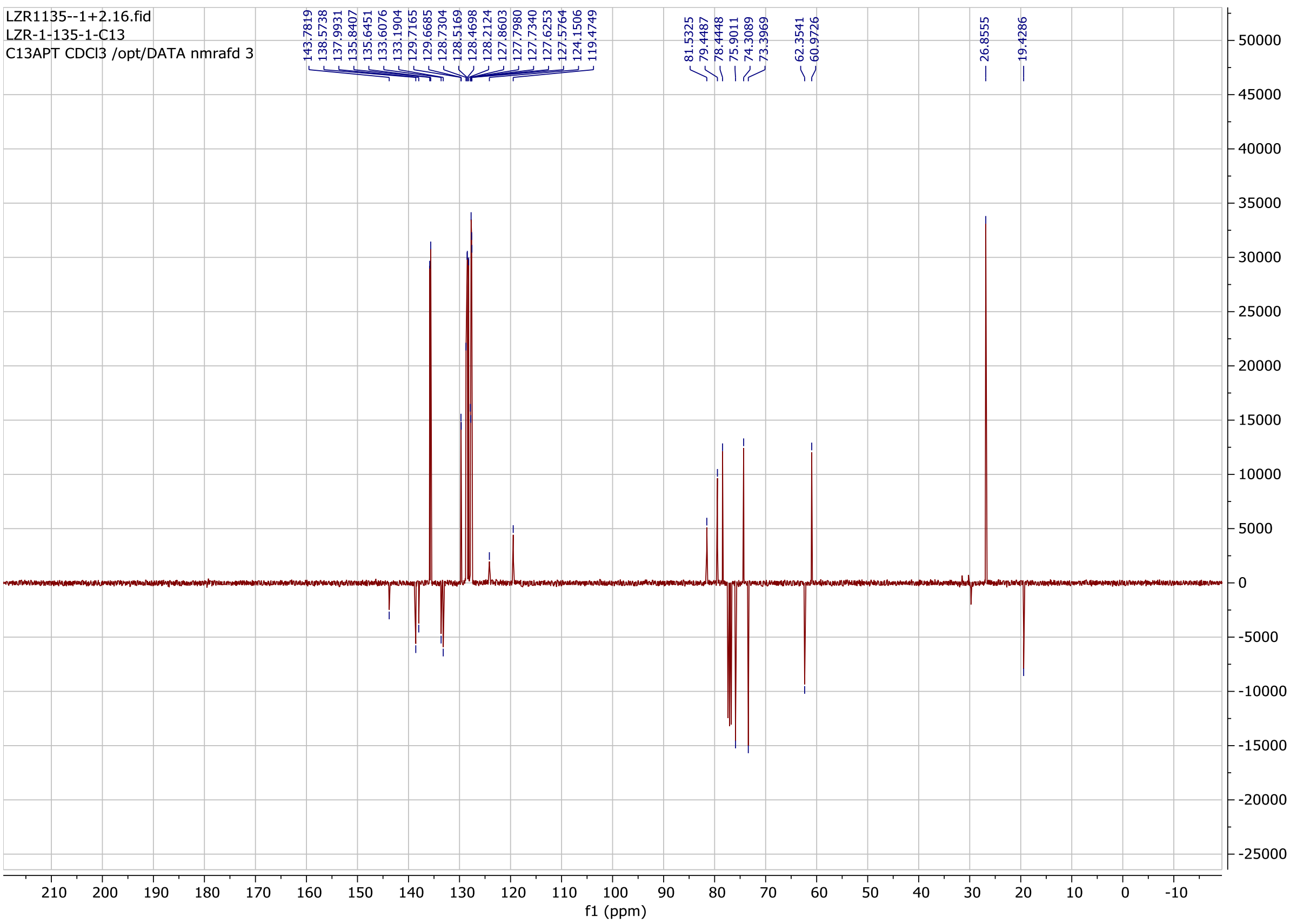

^{1}H and ^{13}C of compound **19** in $CDCl_3$

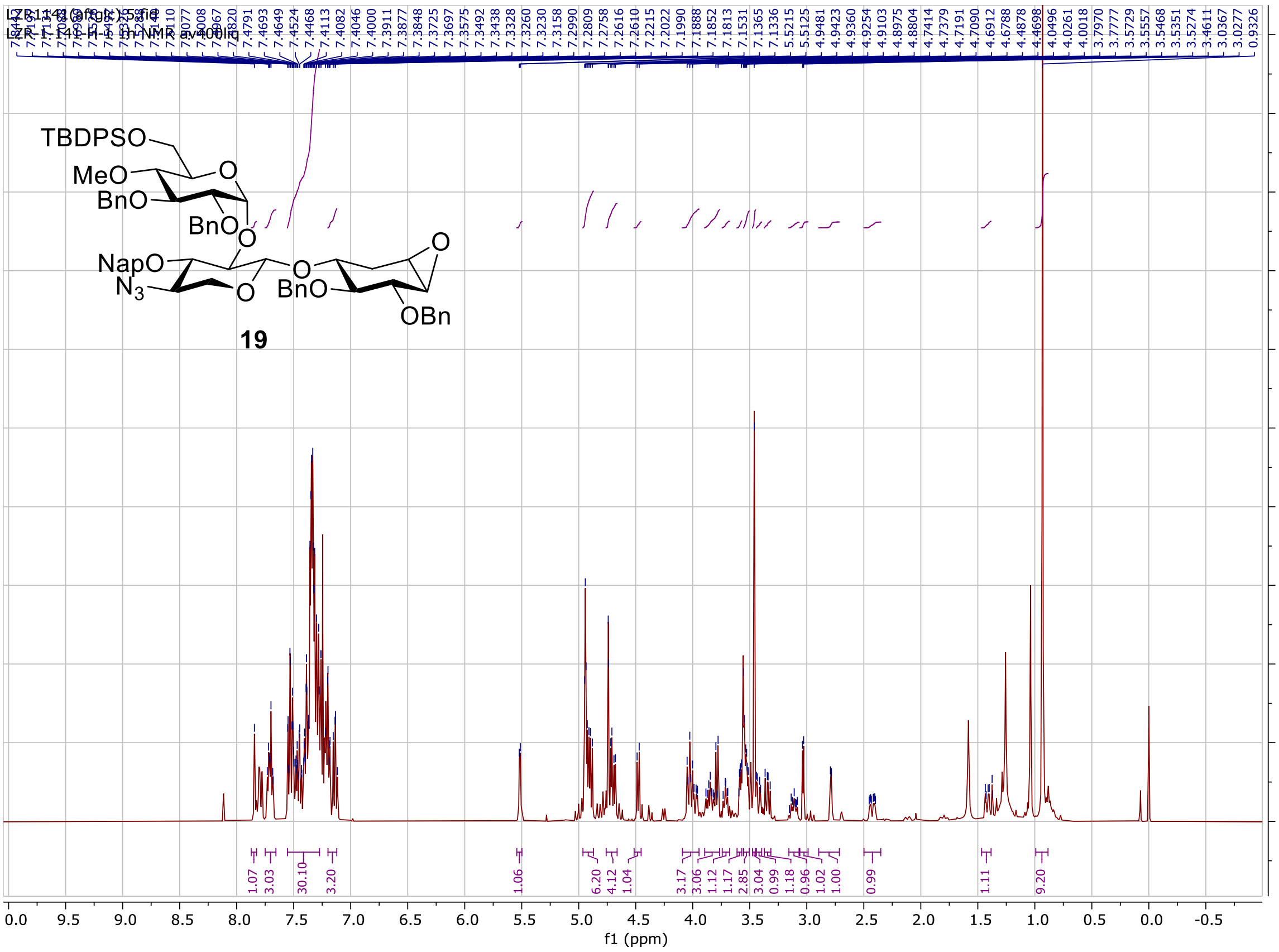

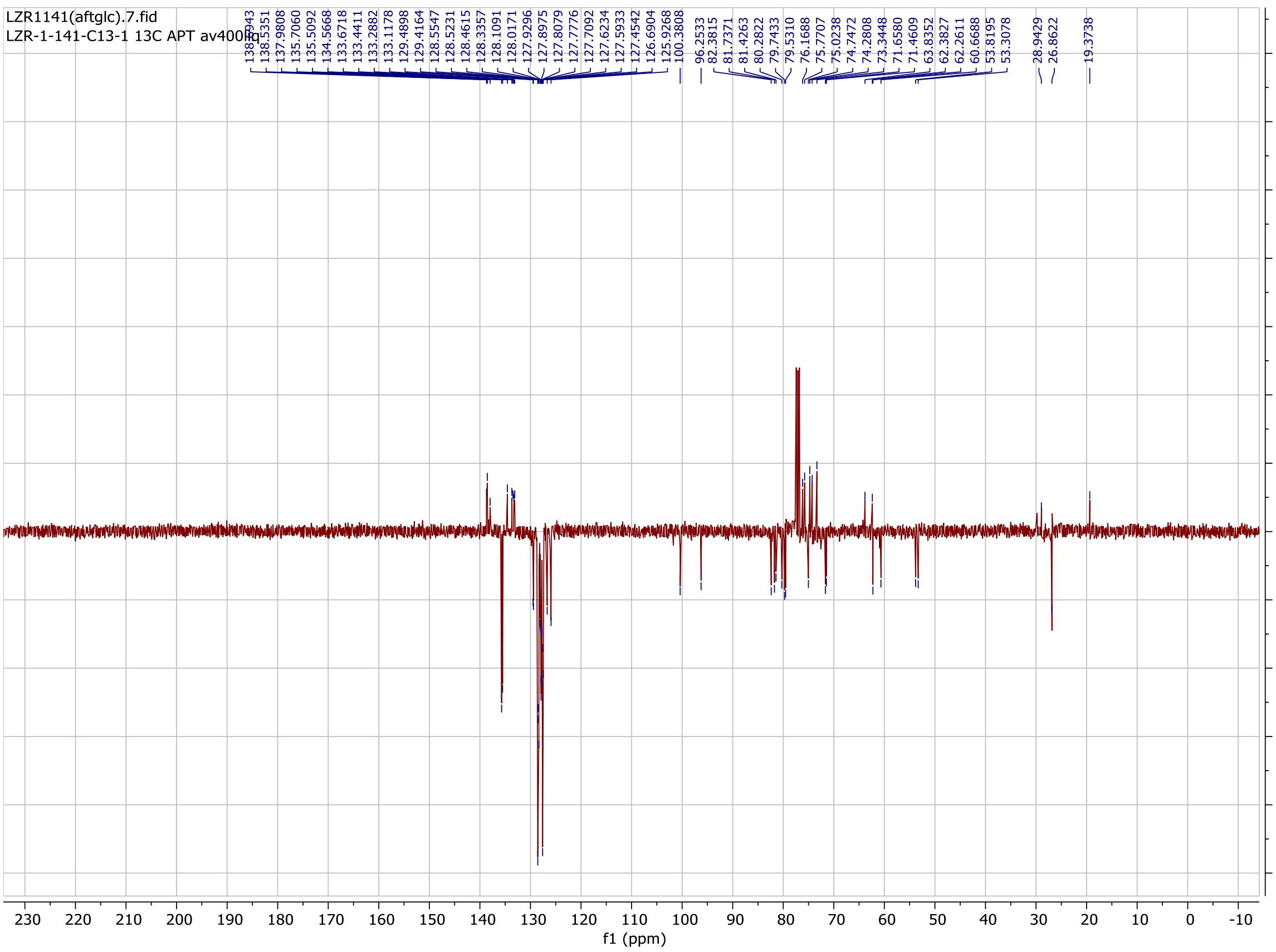

^{1}H and ^{13}C of compound **S19** in $CDCl_3$

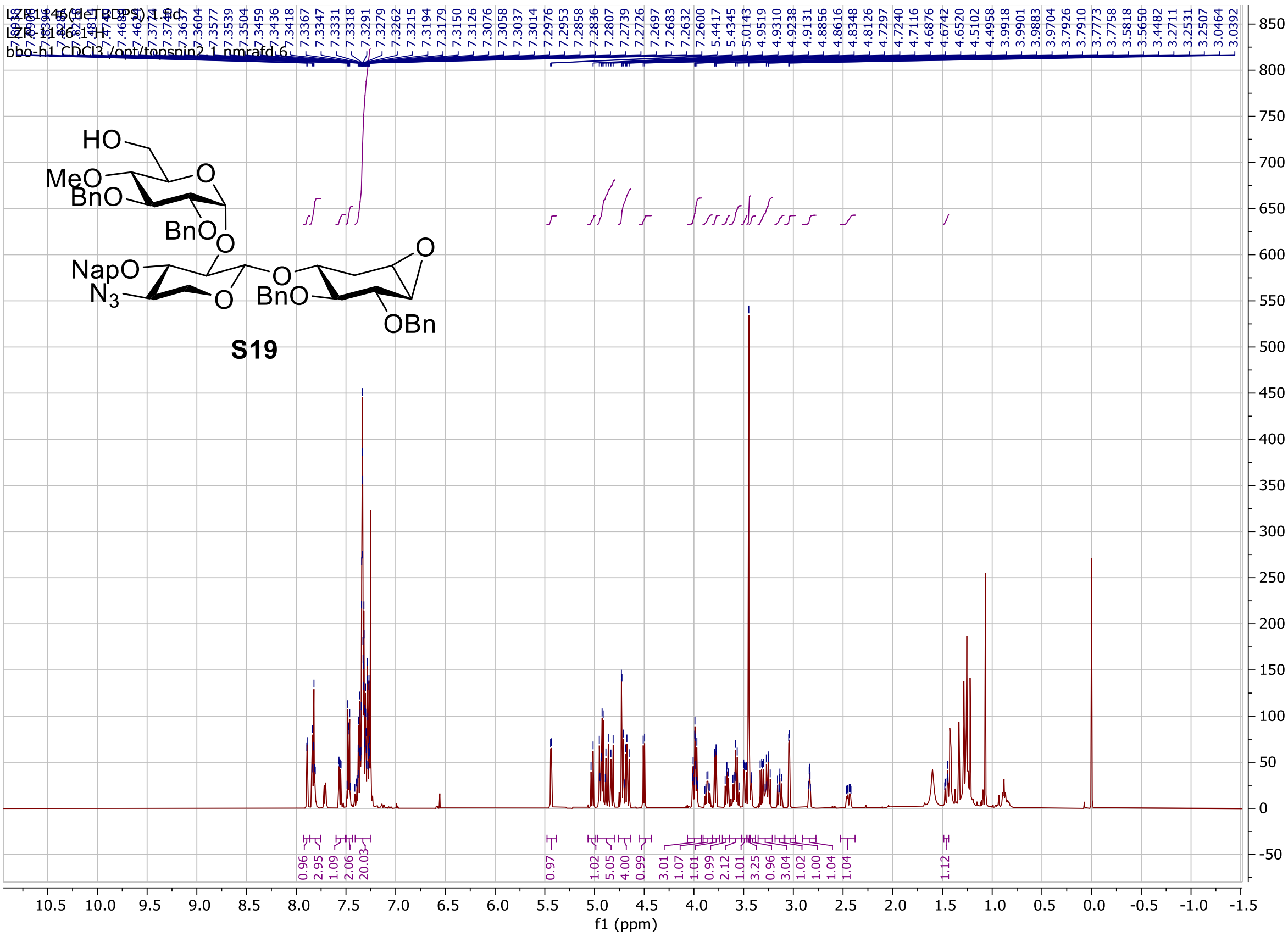

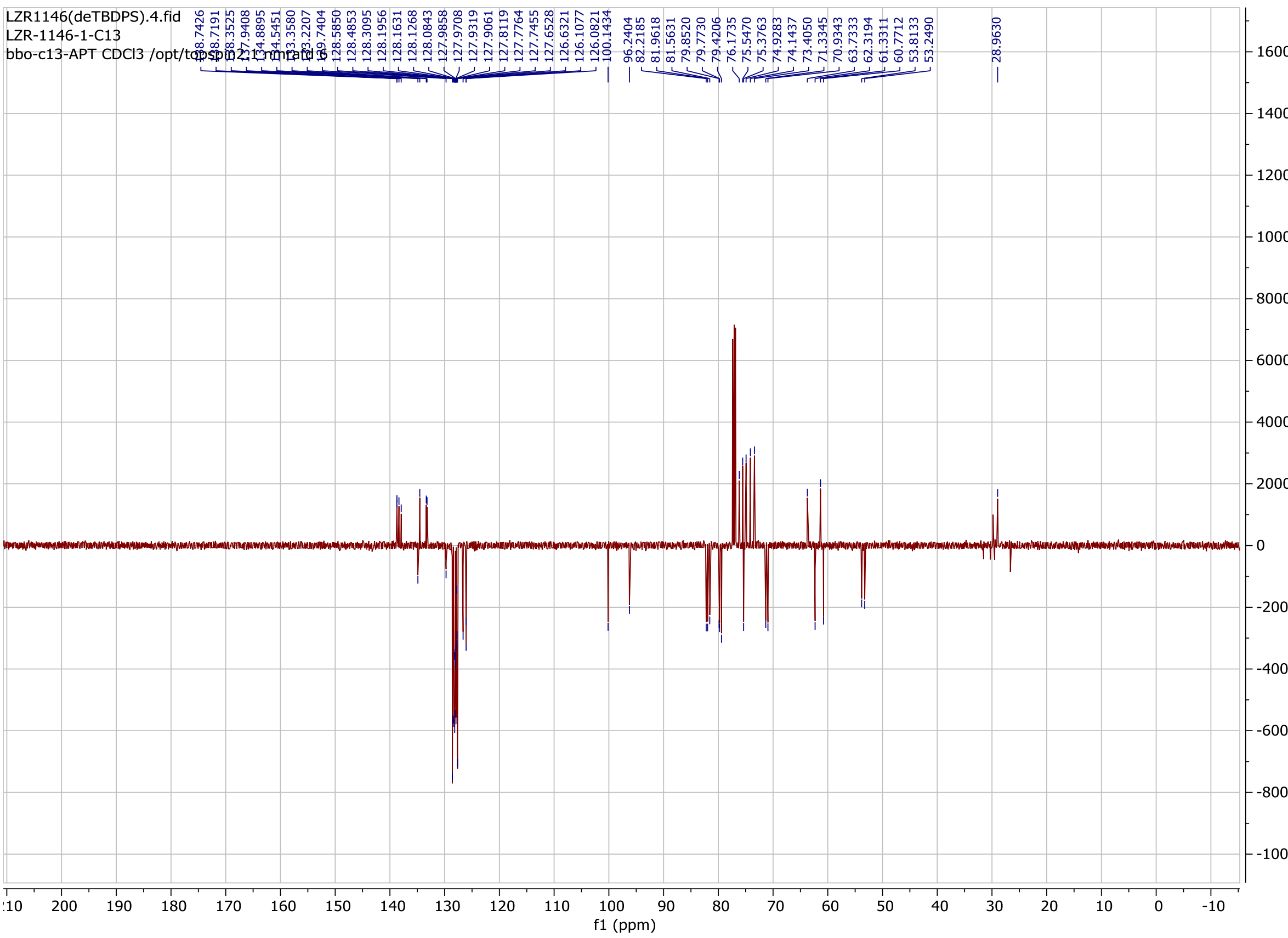

^{1}H and ^{13}C of compound **S20** in $CDCl_3$

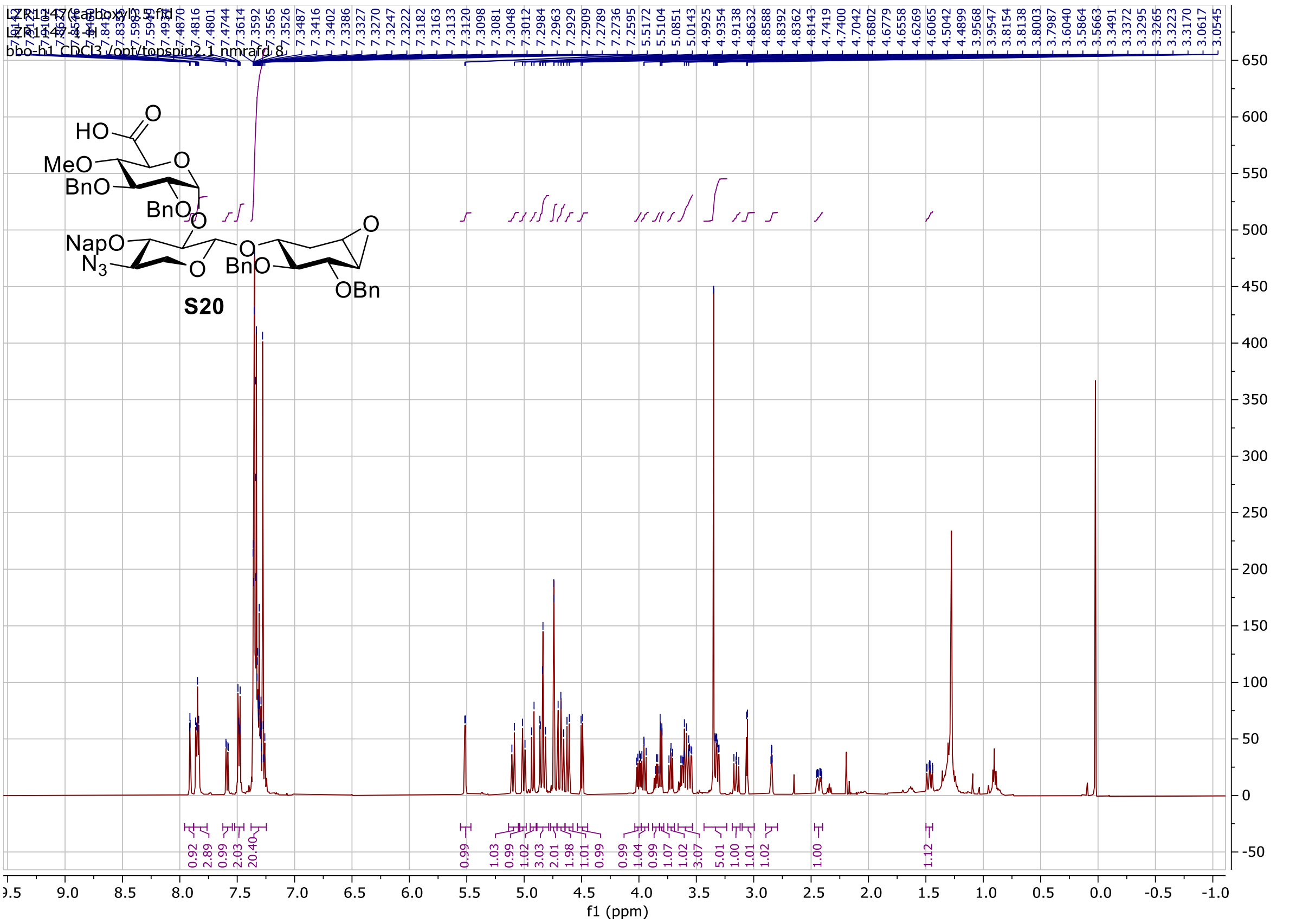

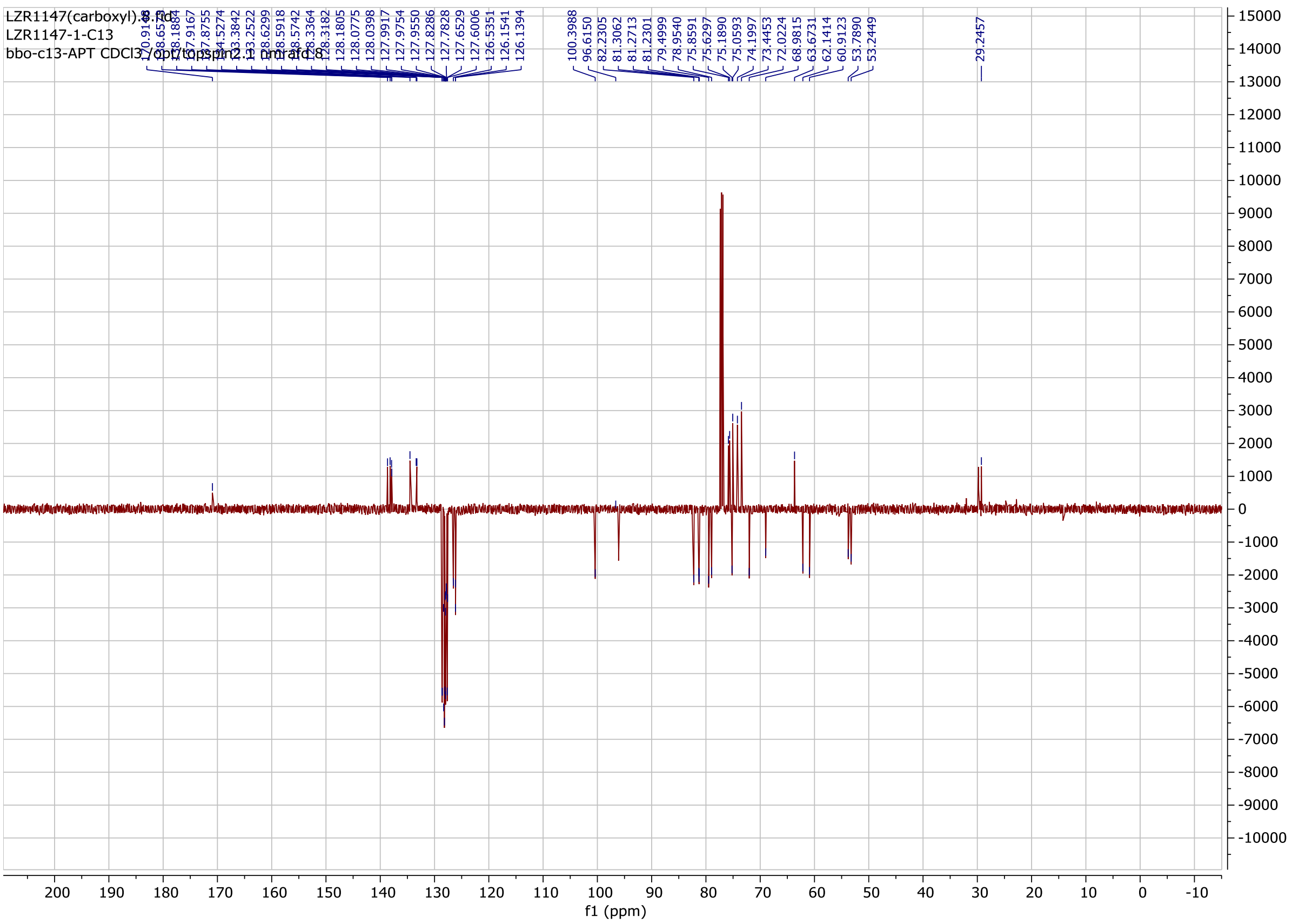

^{1}H and ^{13}C of compound **20** in D_2O

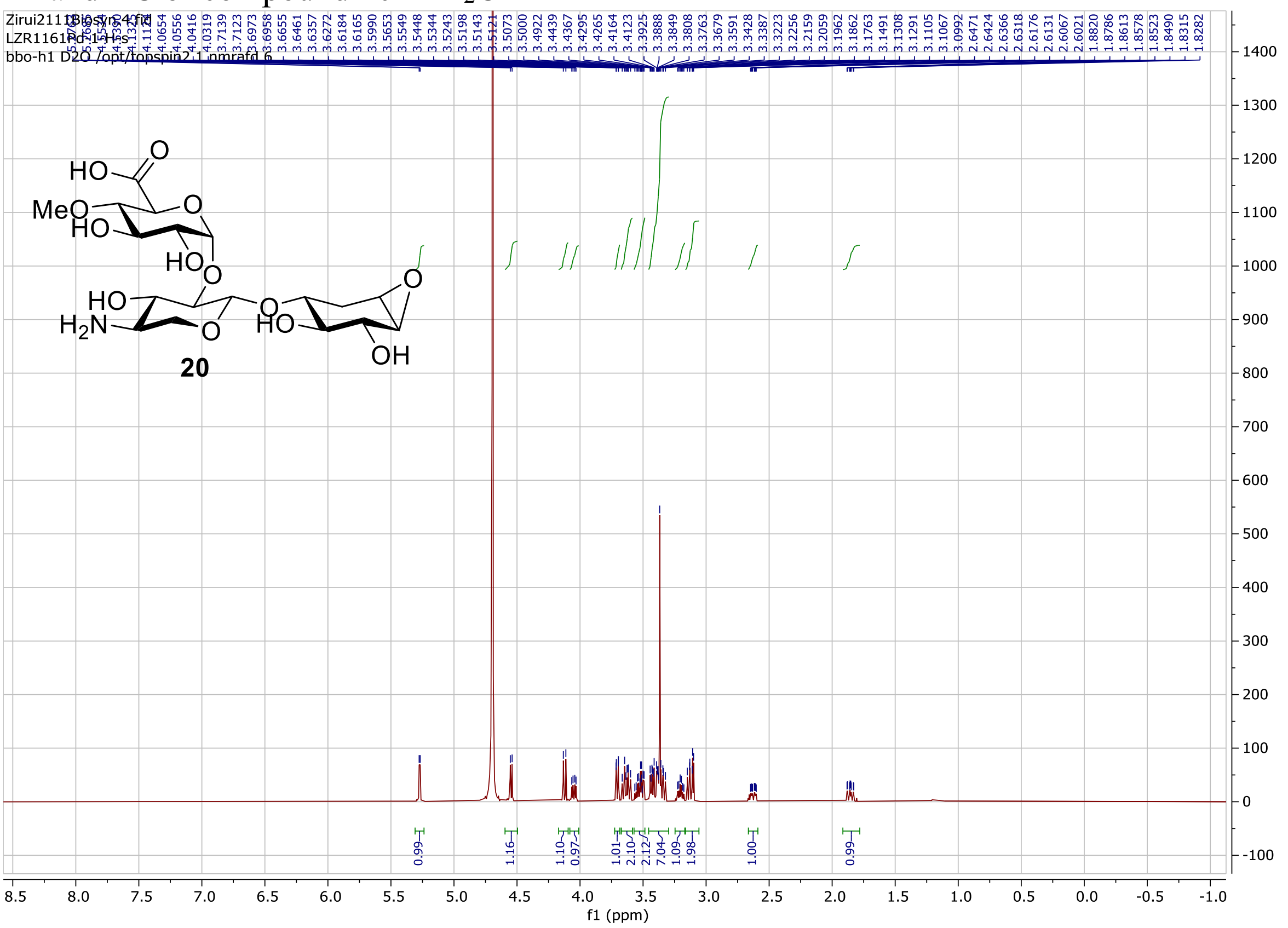

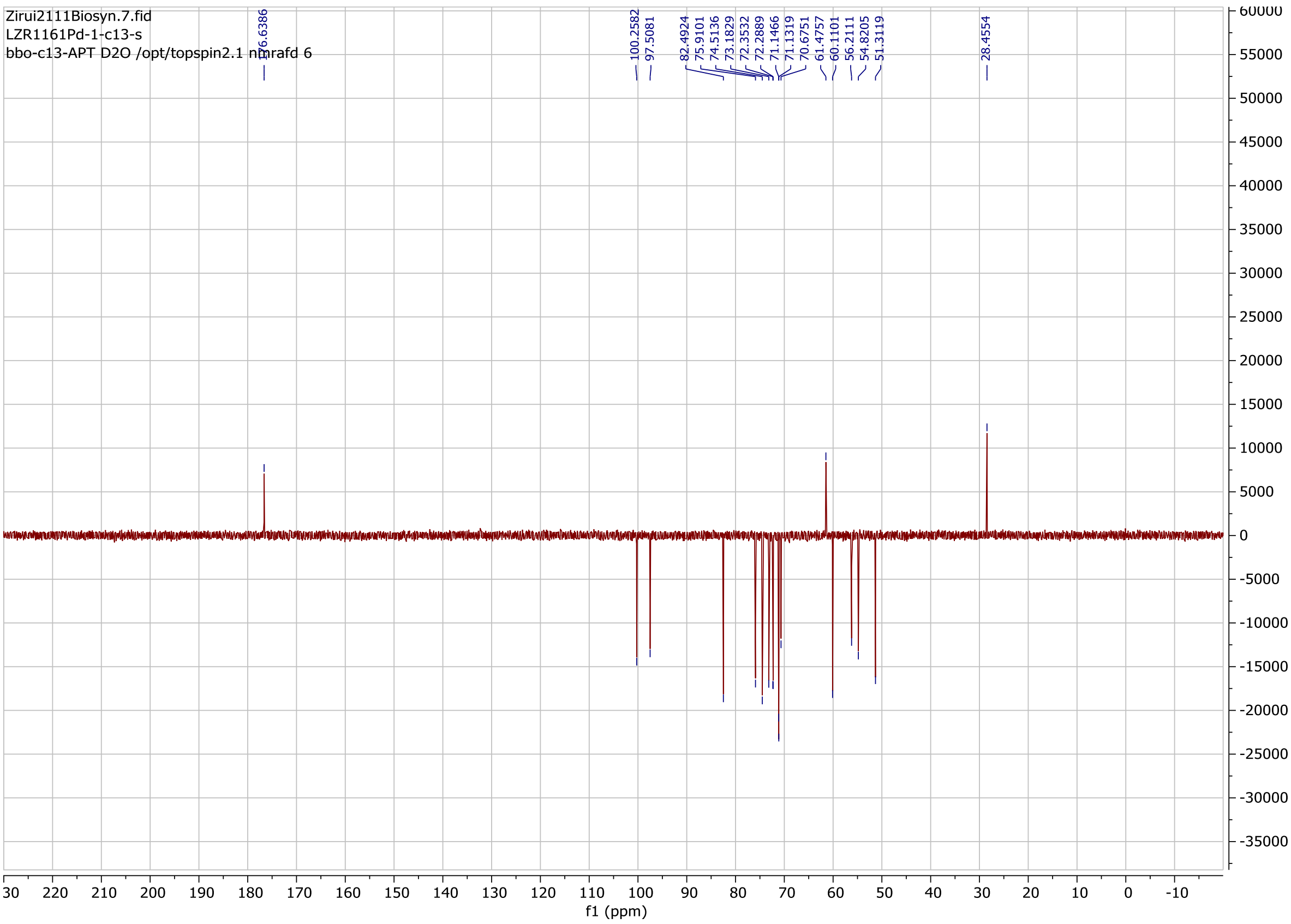

^{1}H and ^{13}C of compound **6-Bio** in D_2O

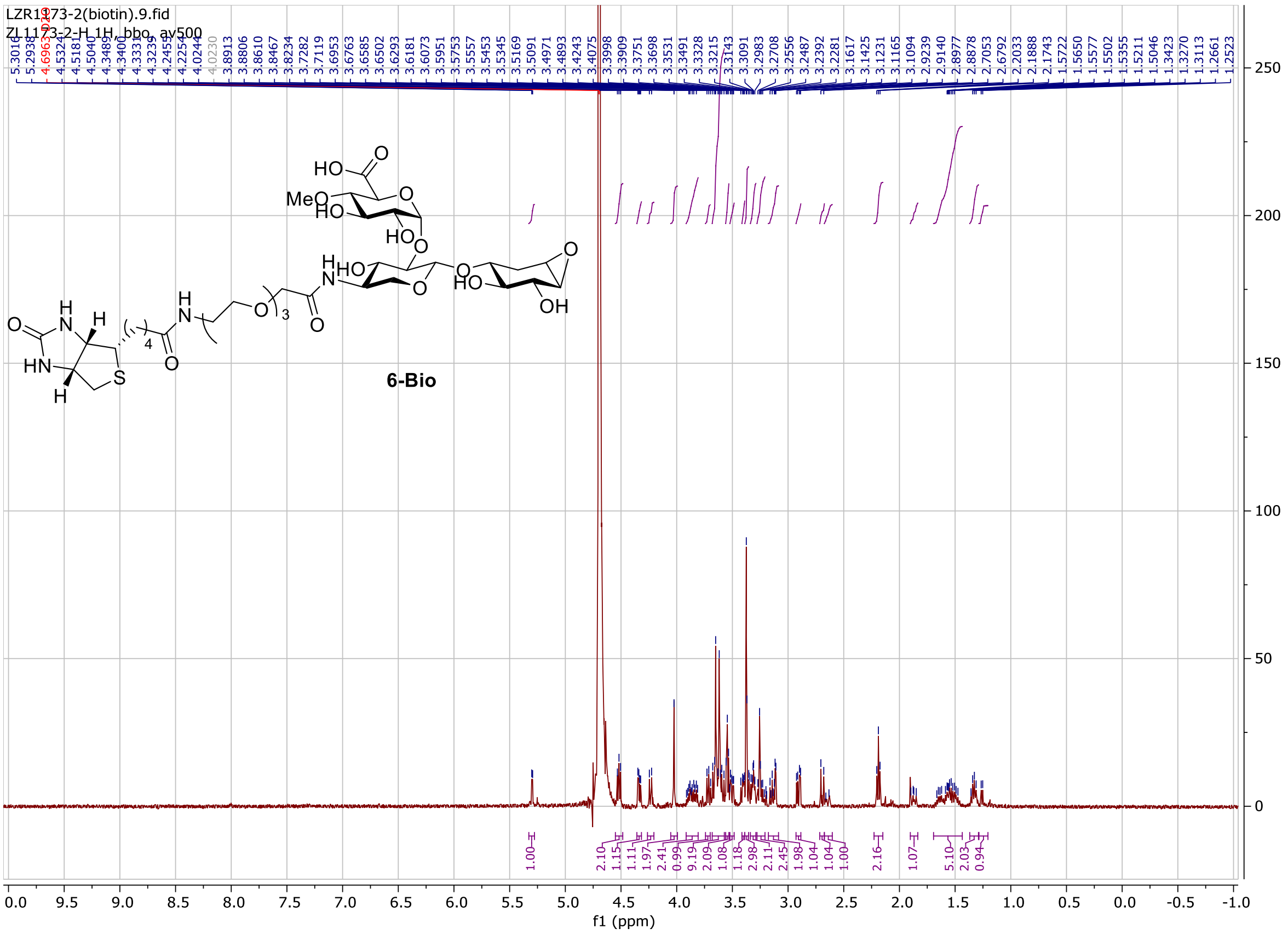

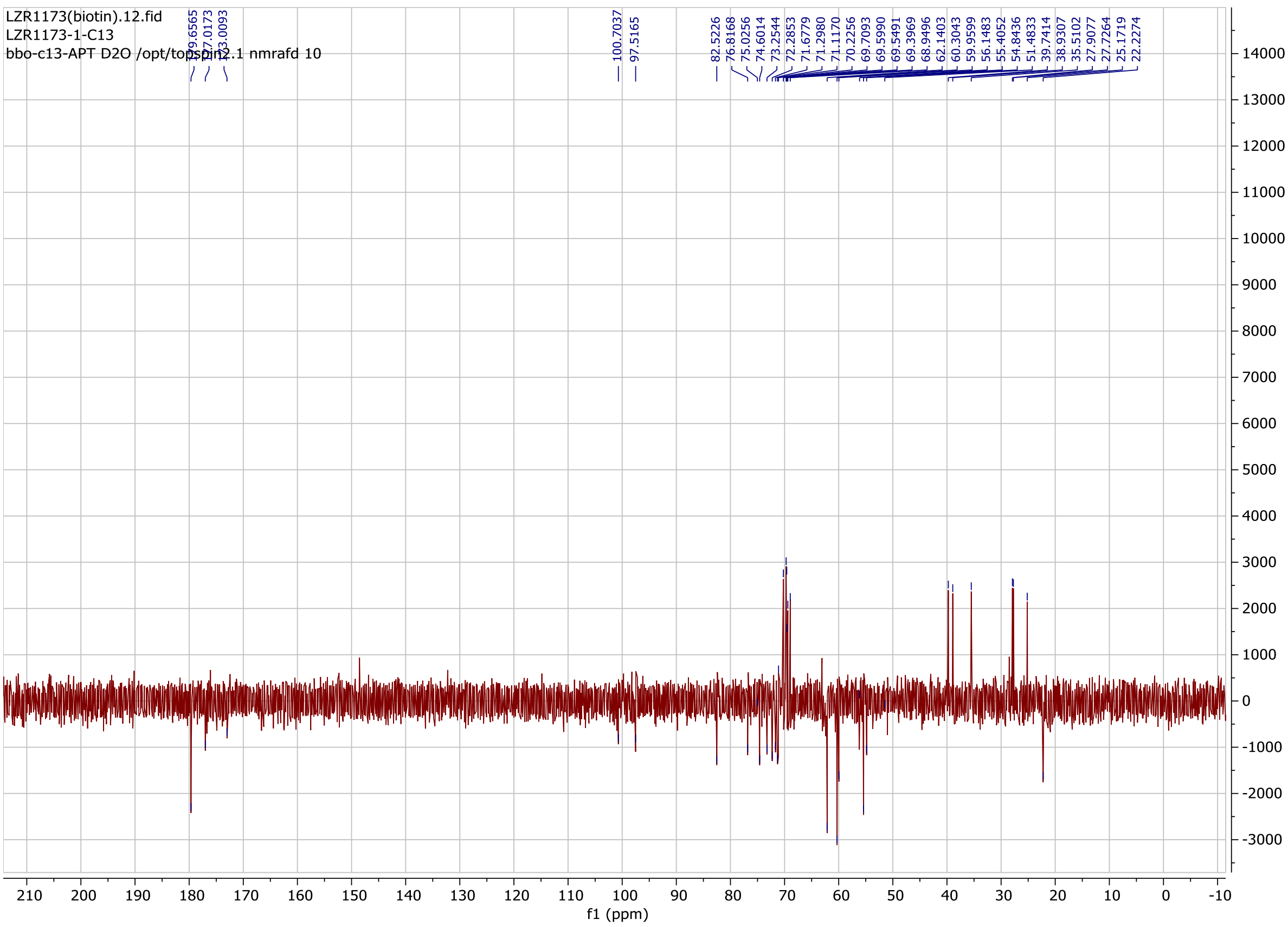

^{1}H of compound **6-Cy5** in D_2O and ^{13}C in MeOD

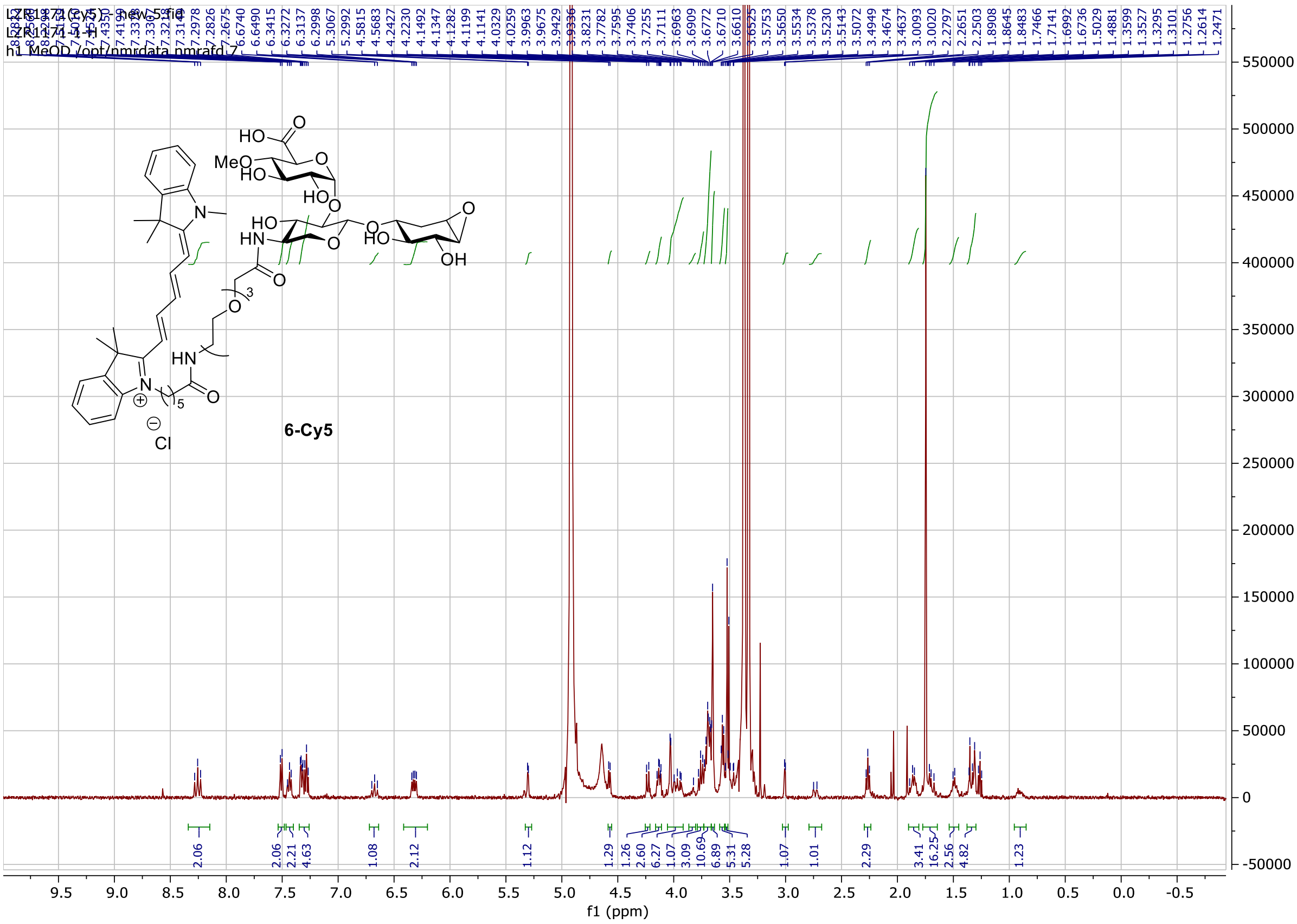

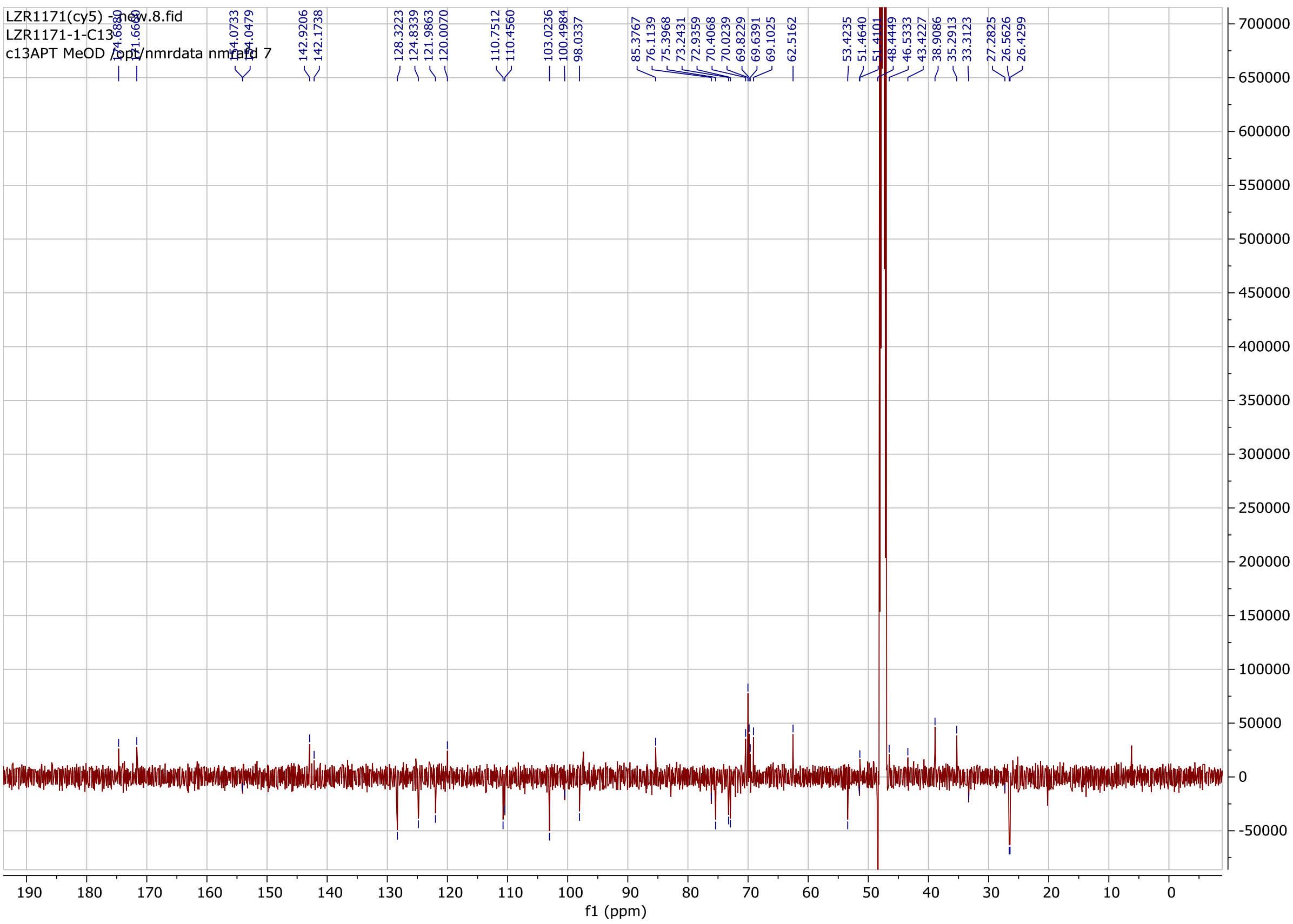

[1]H and ^{13}C of compound **S21** in $CDCl_3$

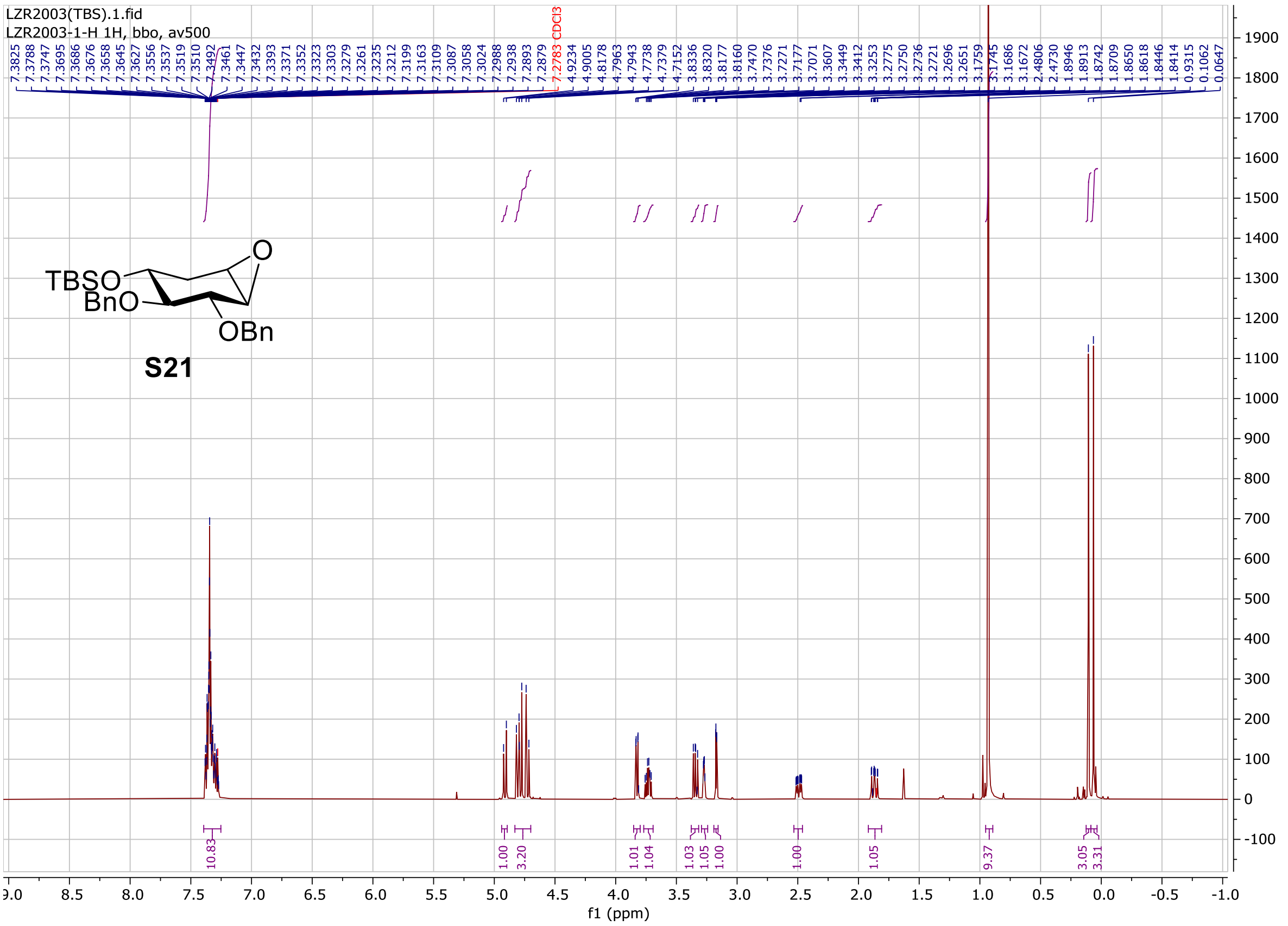

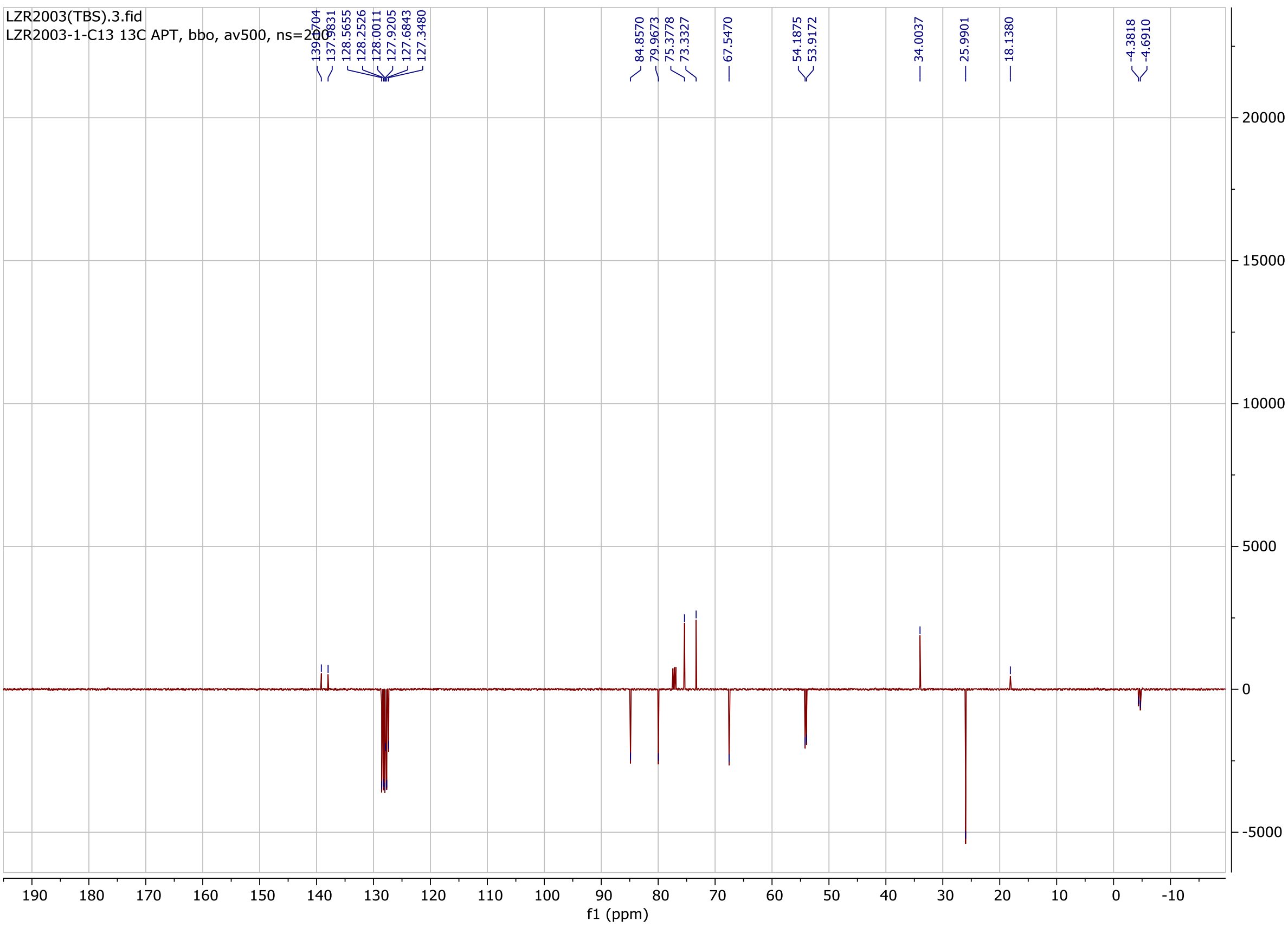

[1]H and ^{13}C of compound **S22** in $CDCl_3$

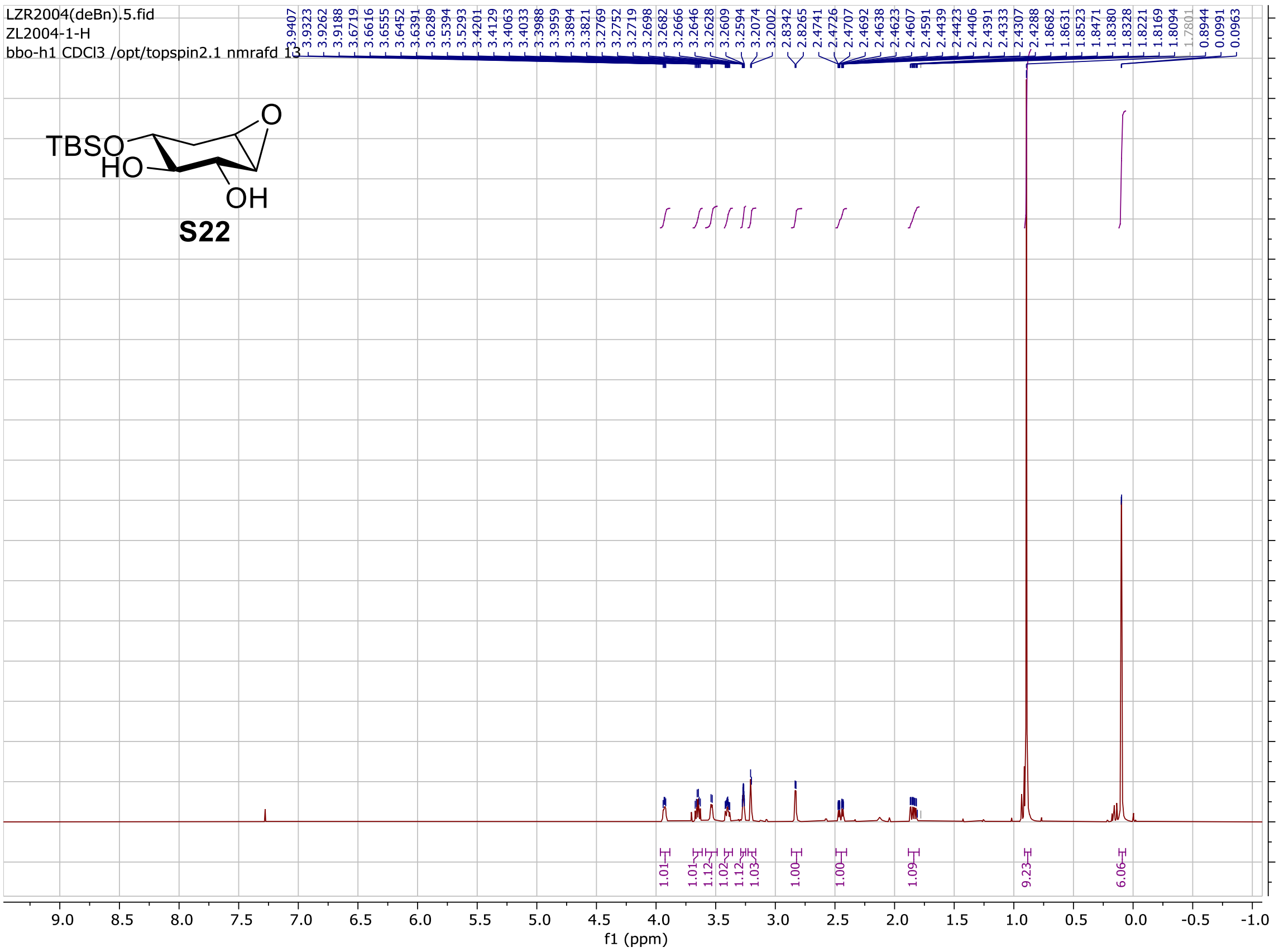

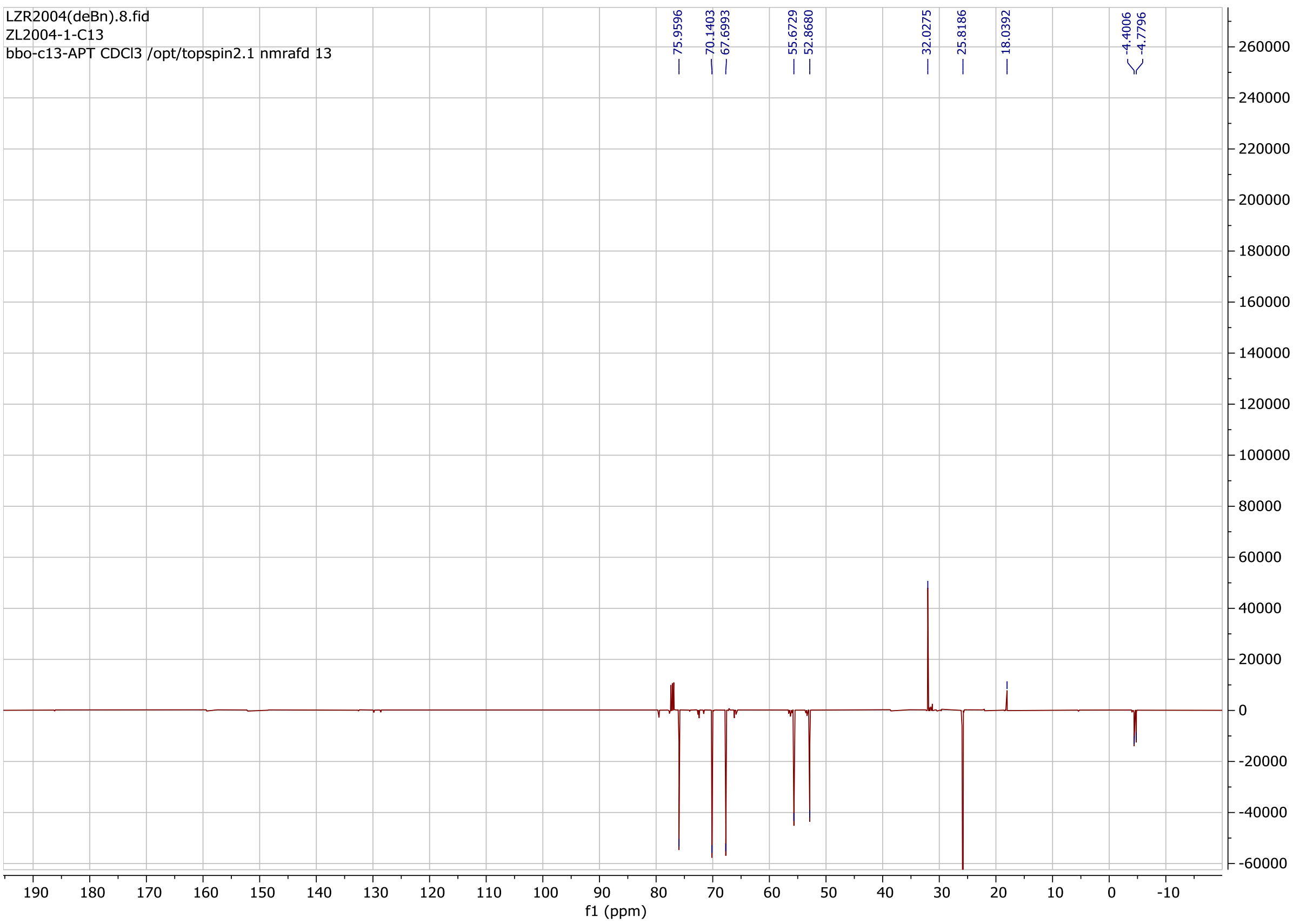

^{1}H and ^{13}C of compound **22** in $CDCl_3$

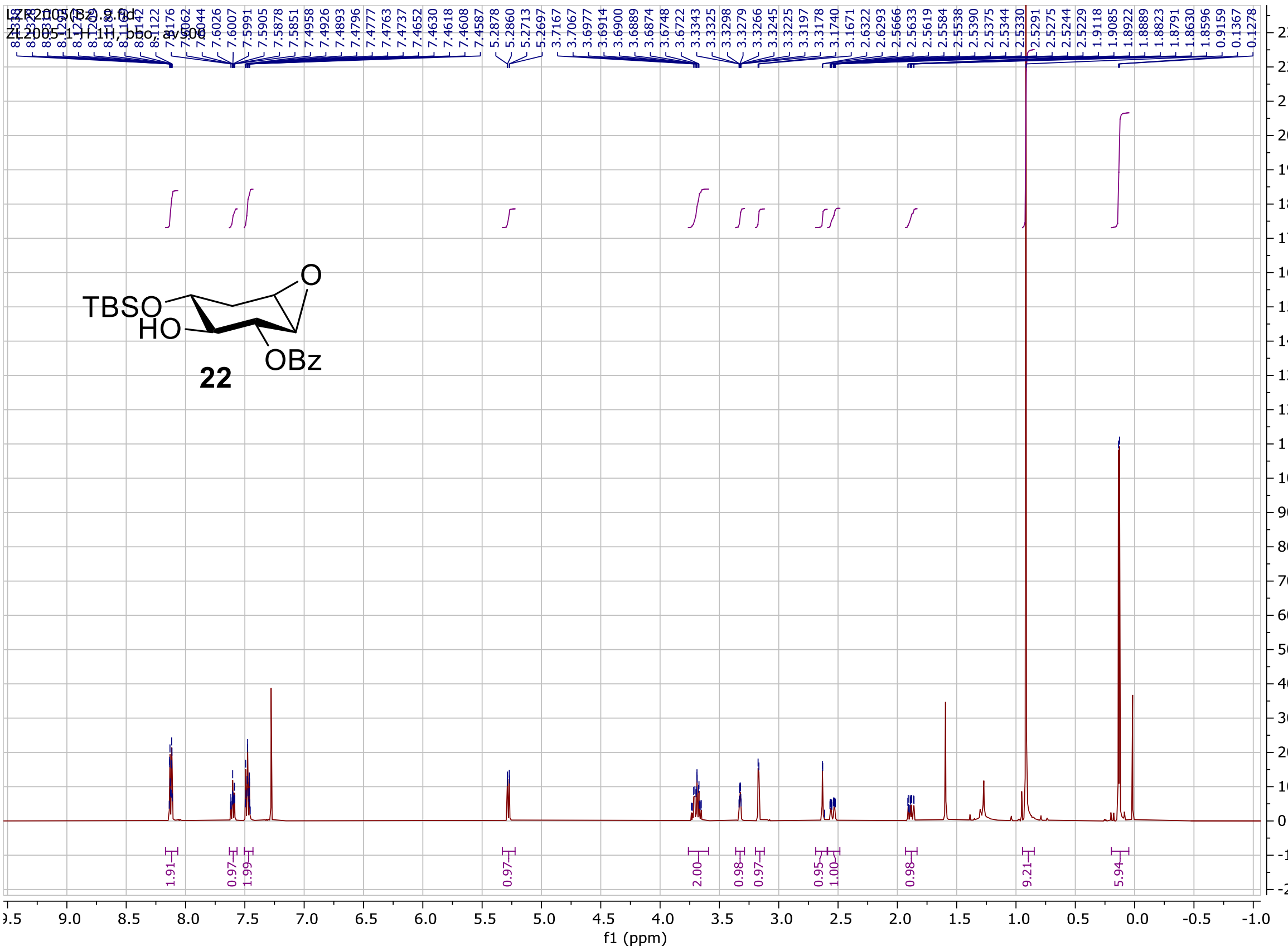

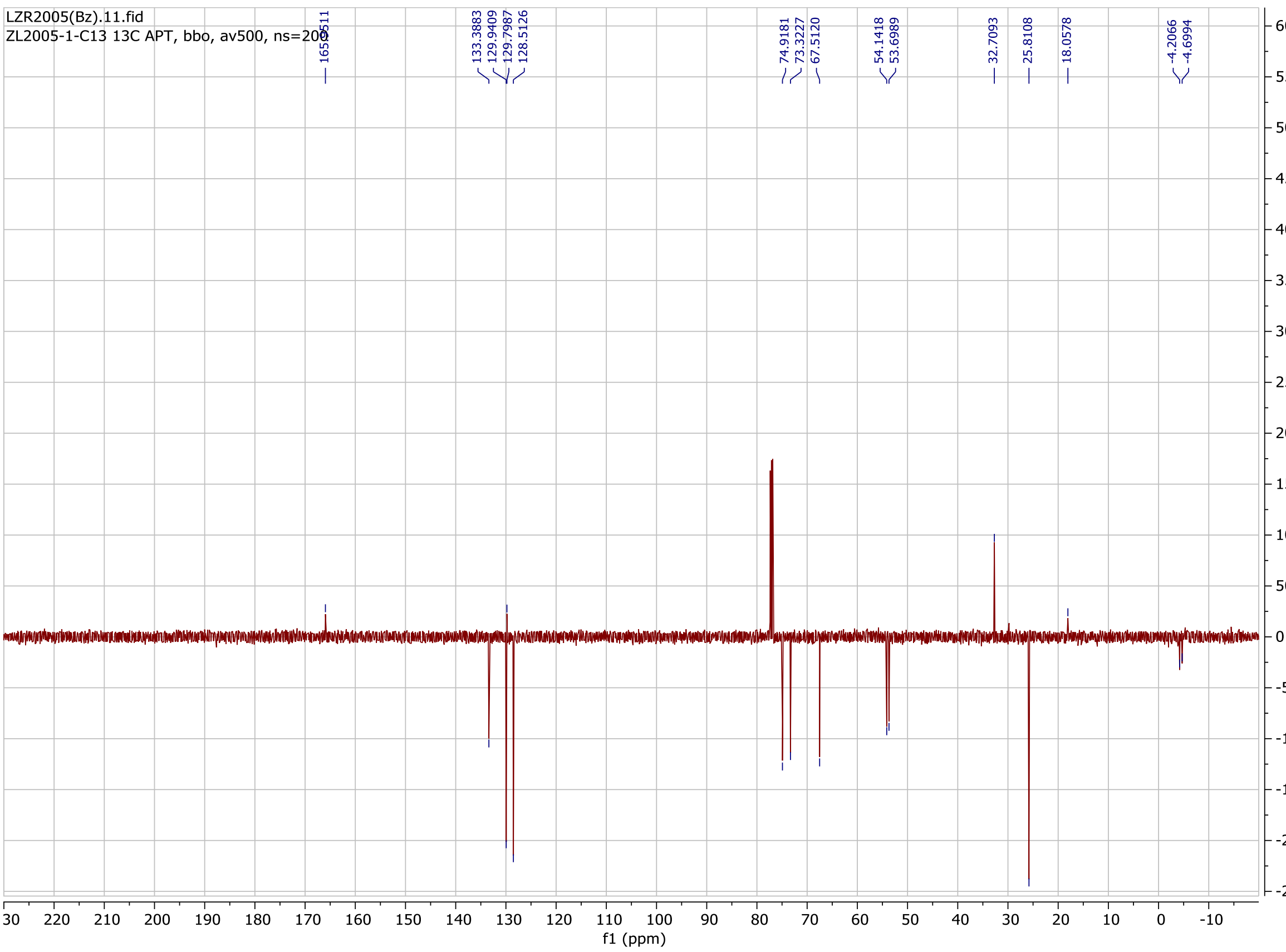

^{1}H and ^{13}C of compound **24** in $CDCl_3$

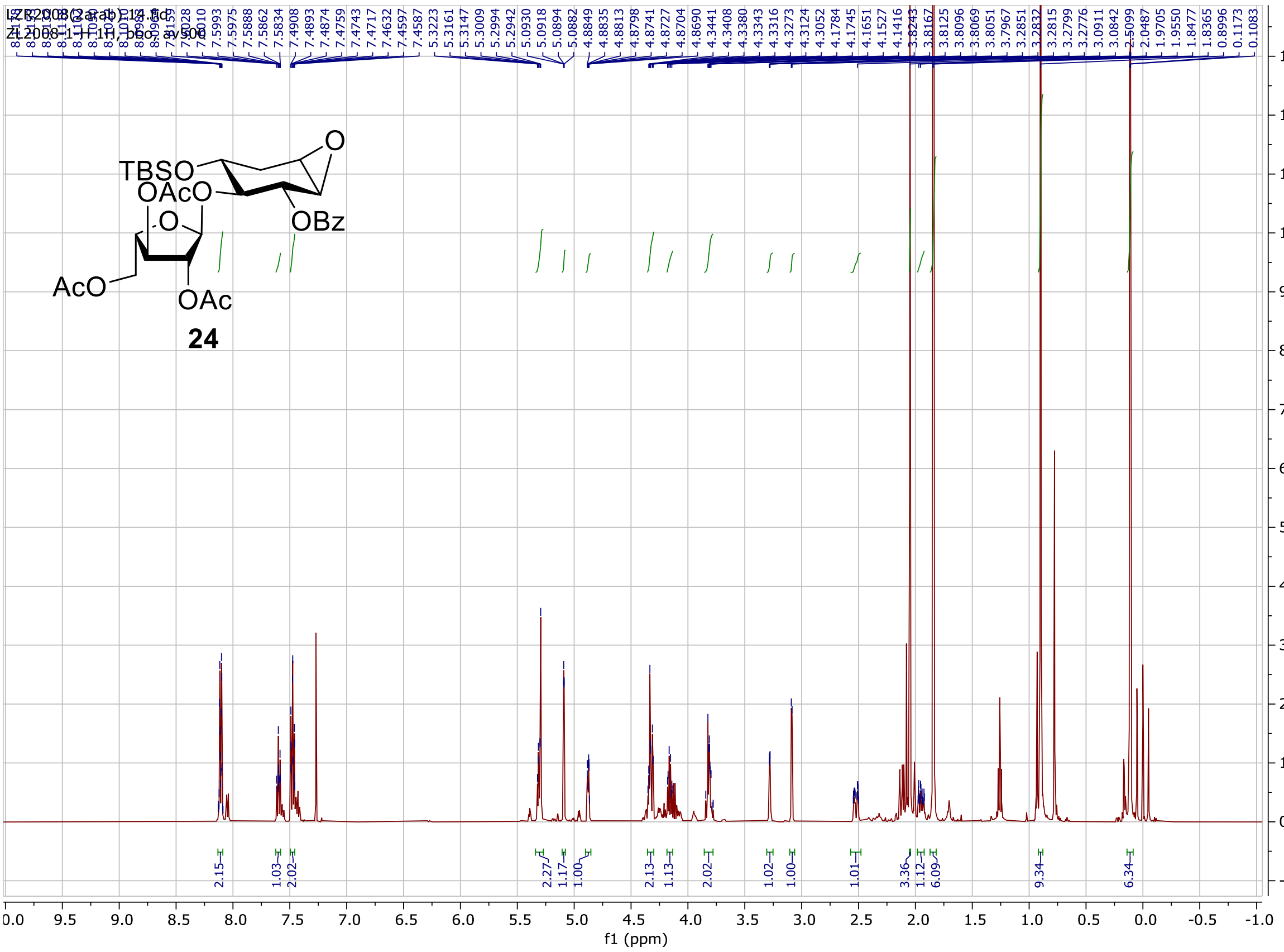

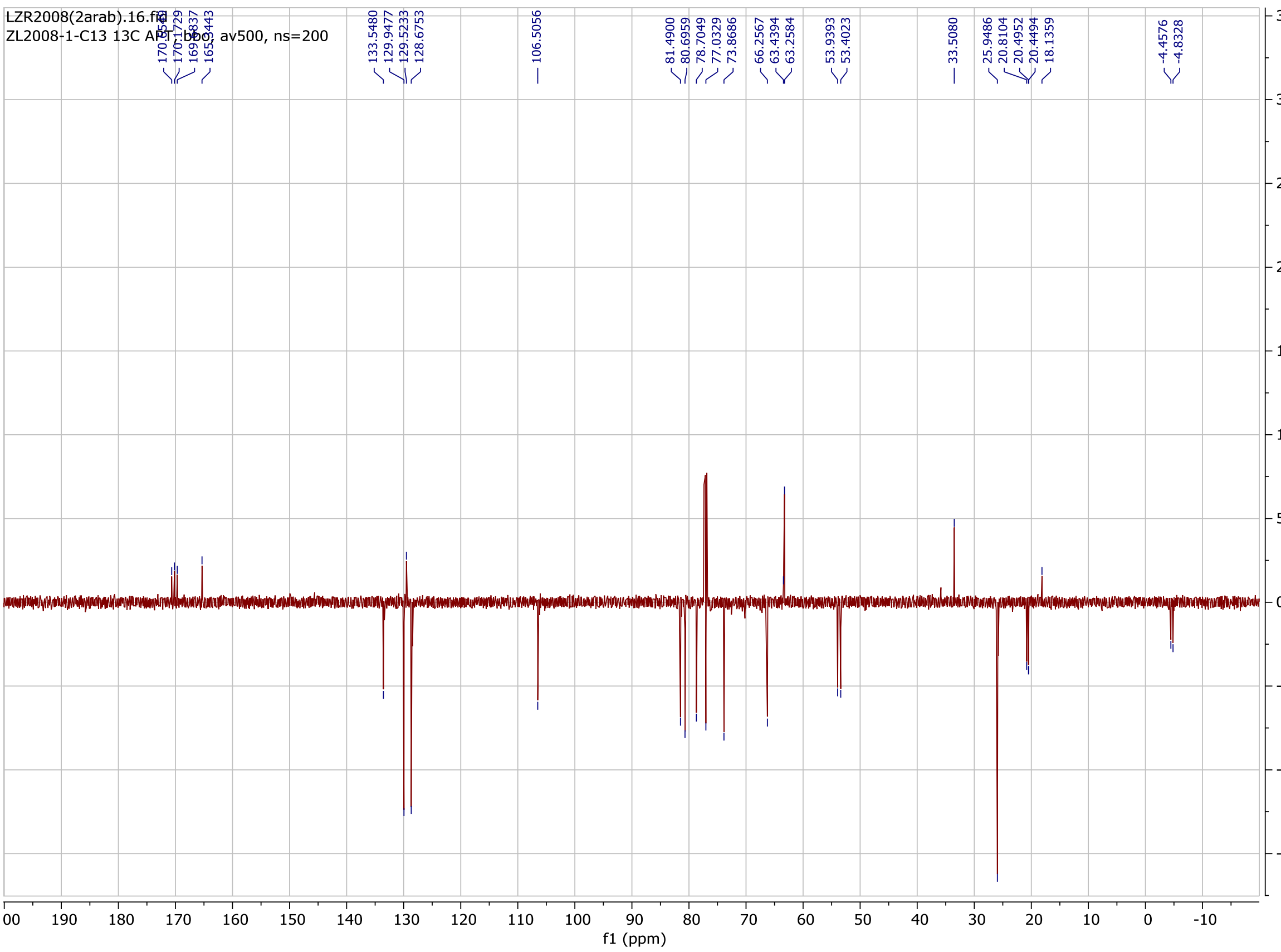

[1]H and ^{13}C of compound **25** in $CDCl_3$

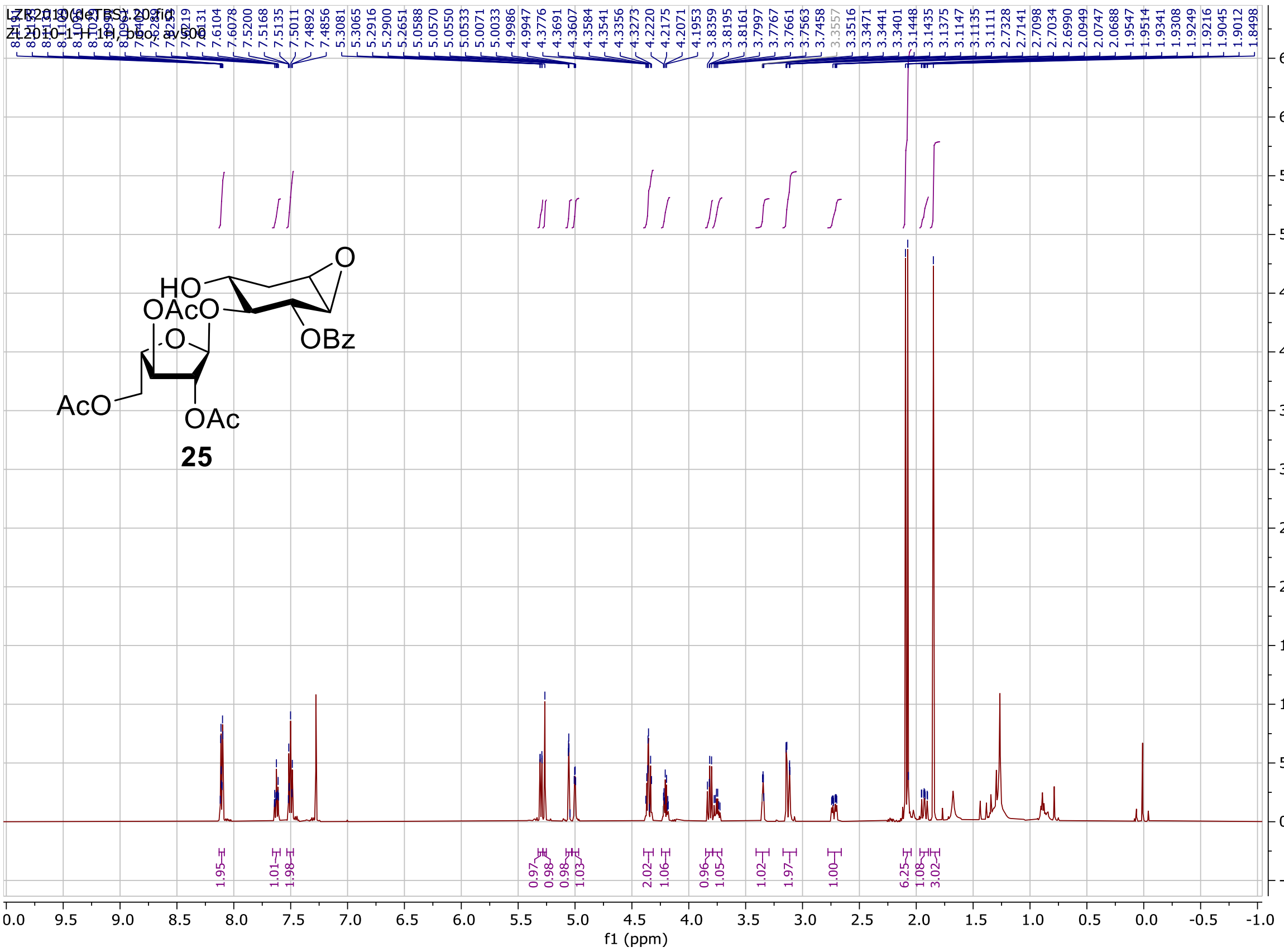

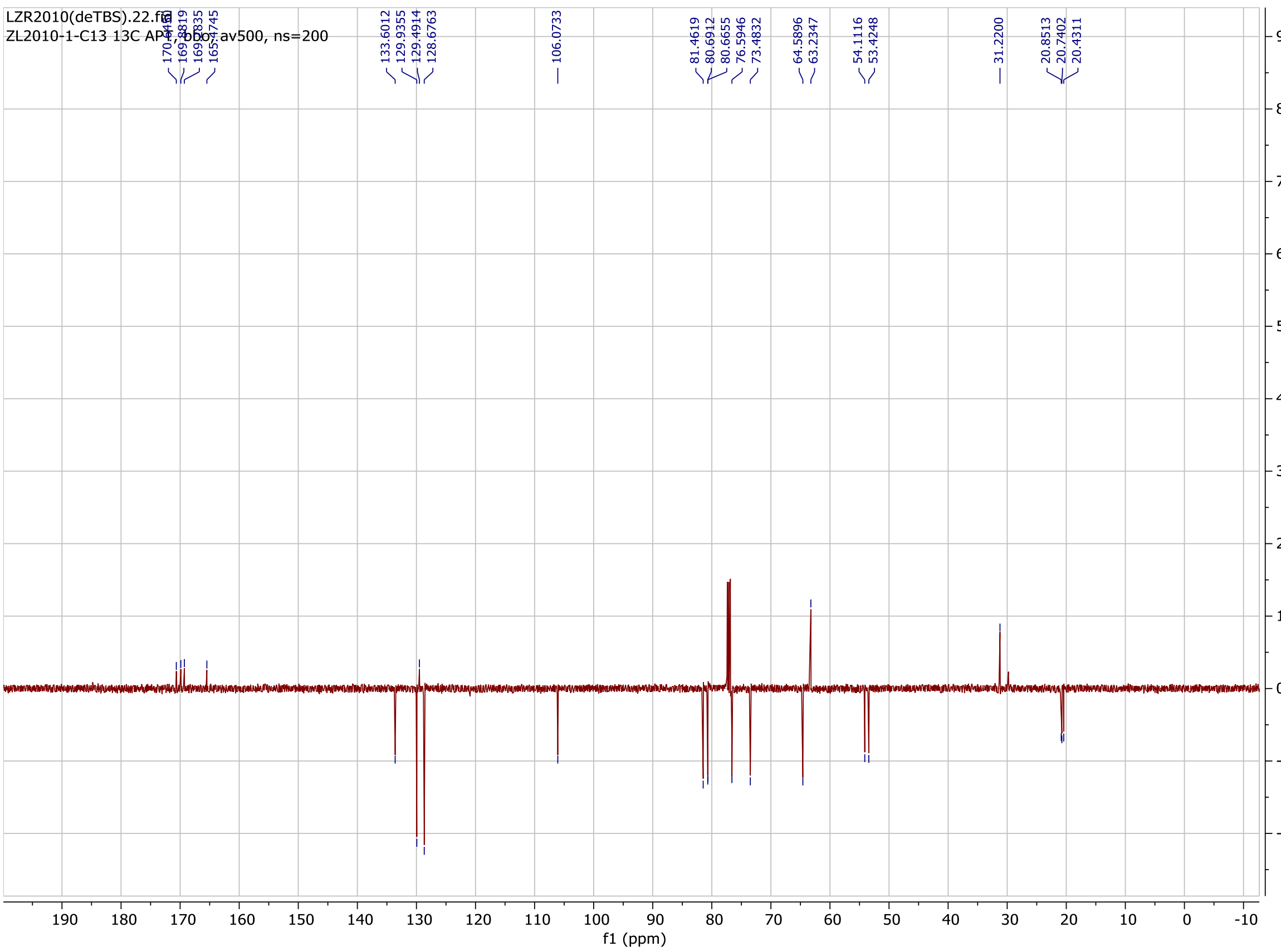

^{1}H and ^{13}C of compound **26** in $CDCl_3$

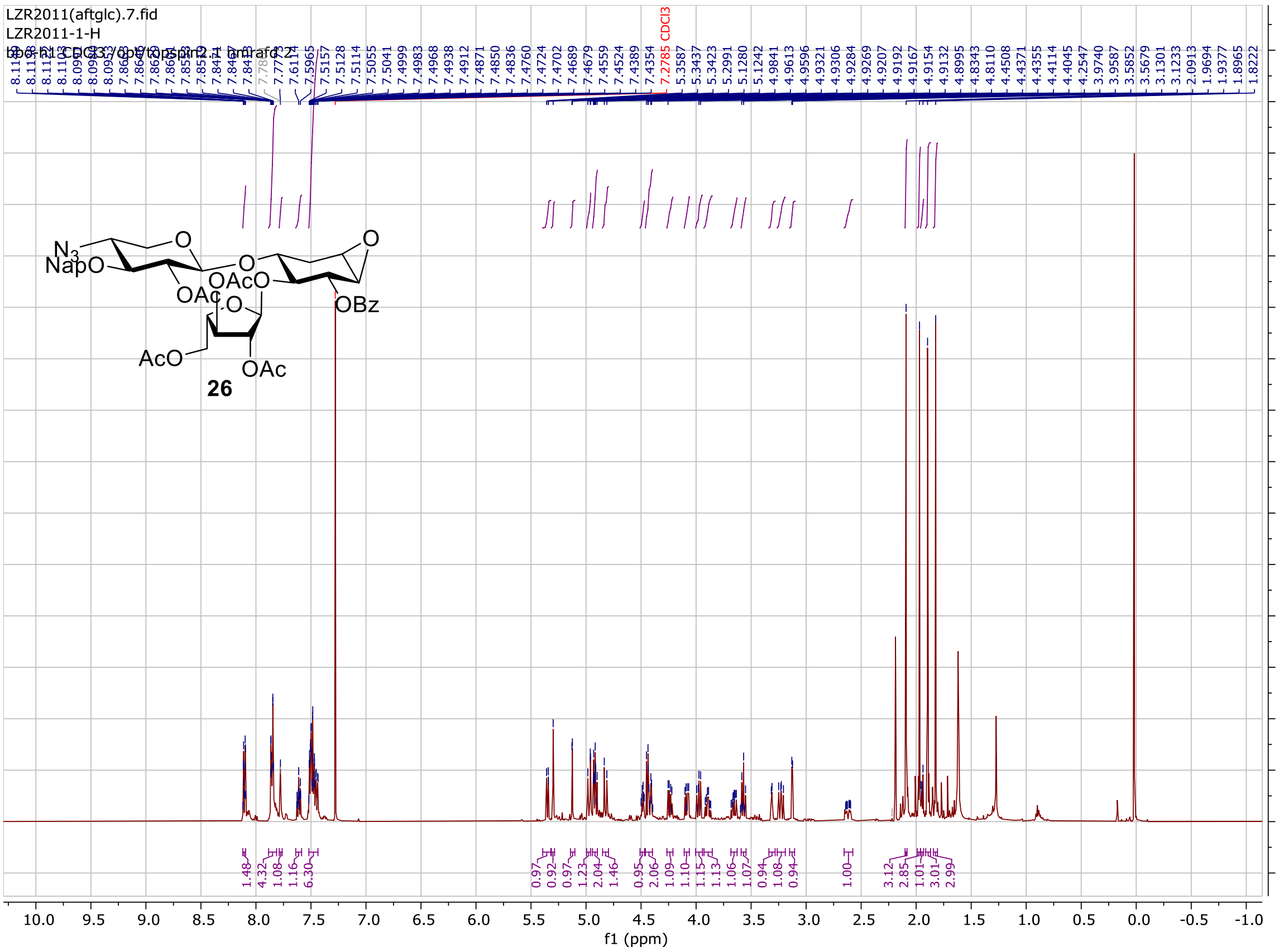

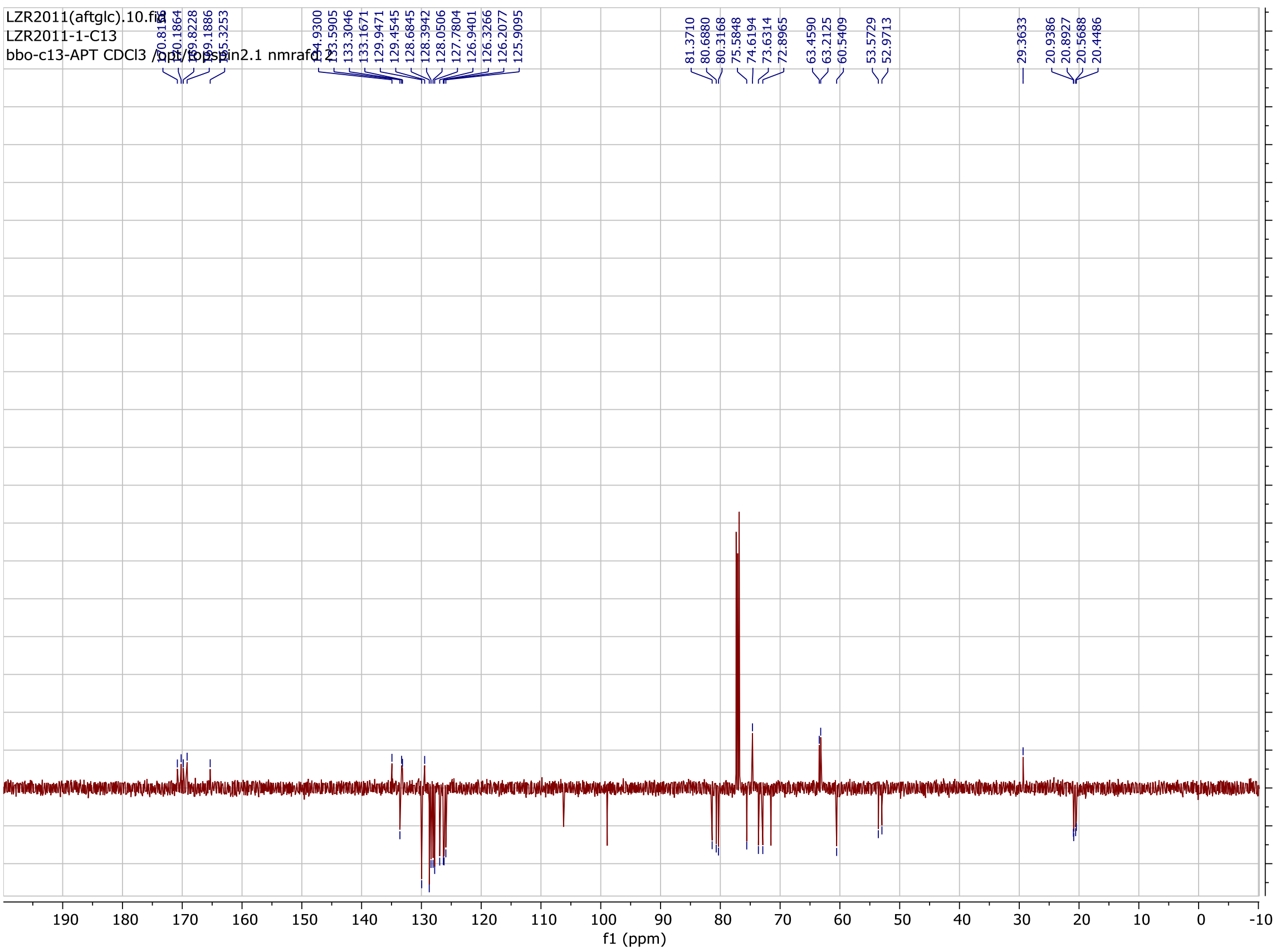

[1]H and ^{13}C of compound **27** in D_2O

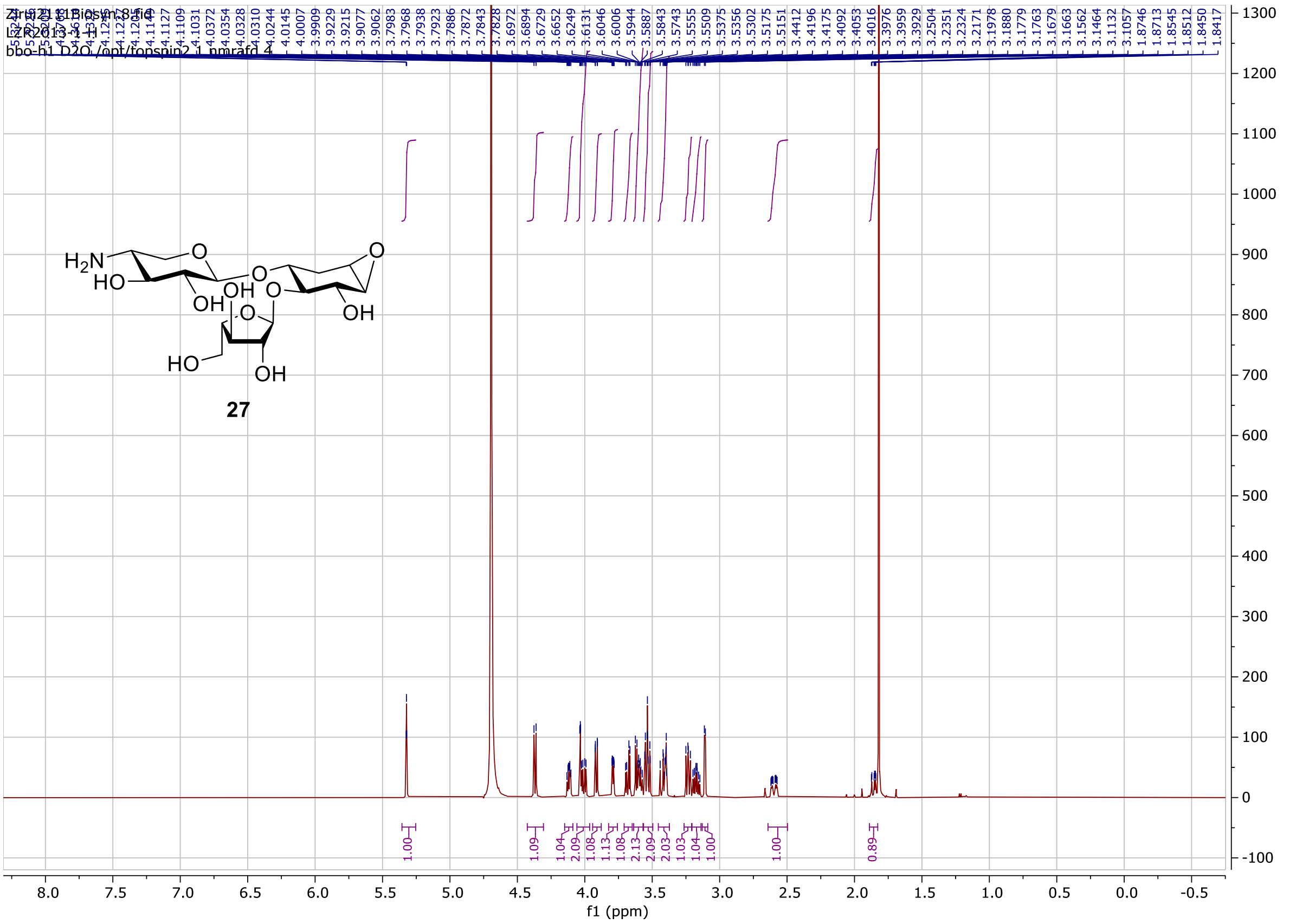

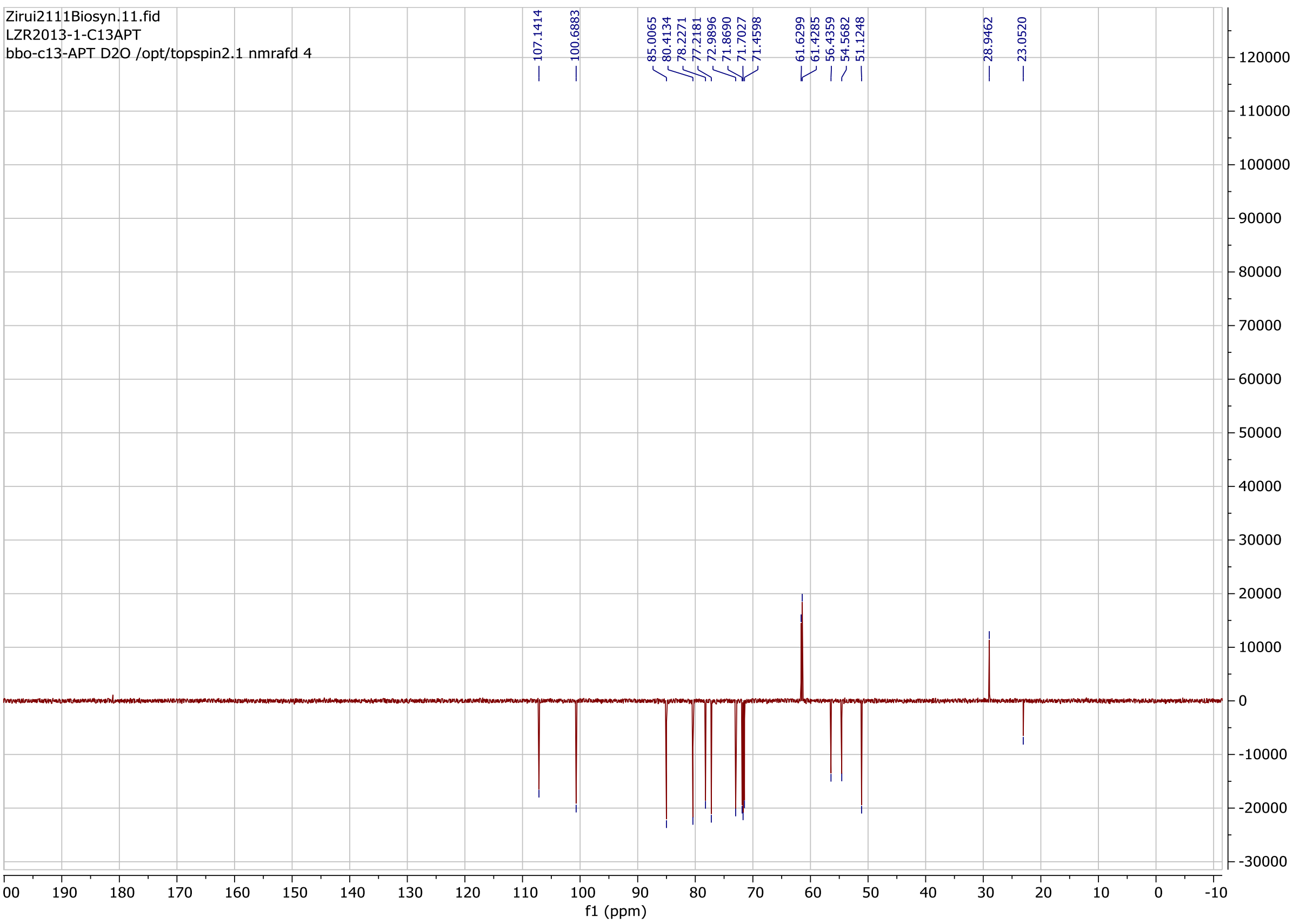

[1]H and ^{13}C of compound **4-Bio** in MeOD

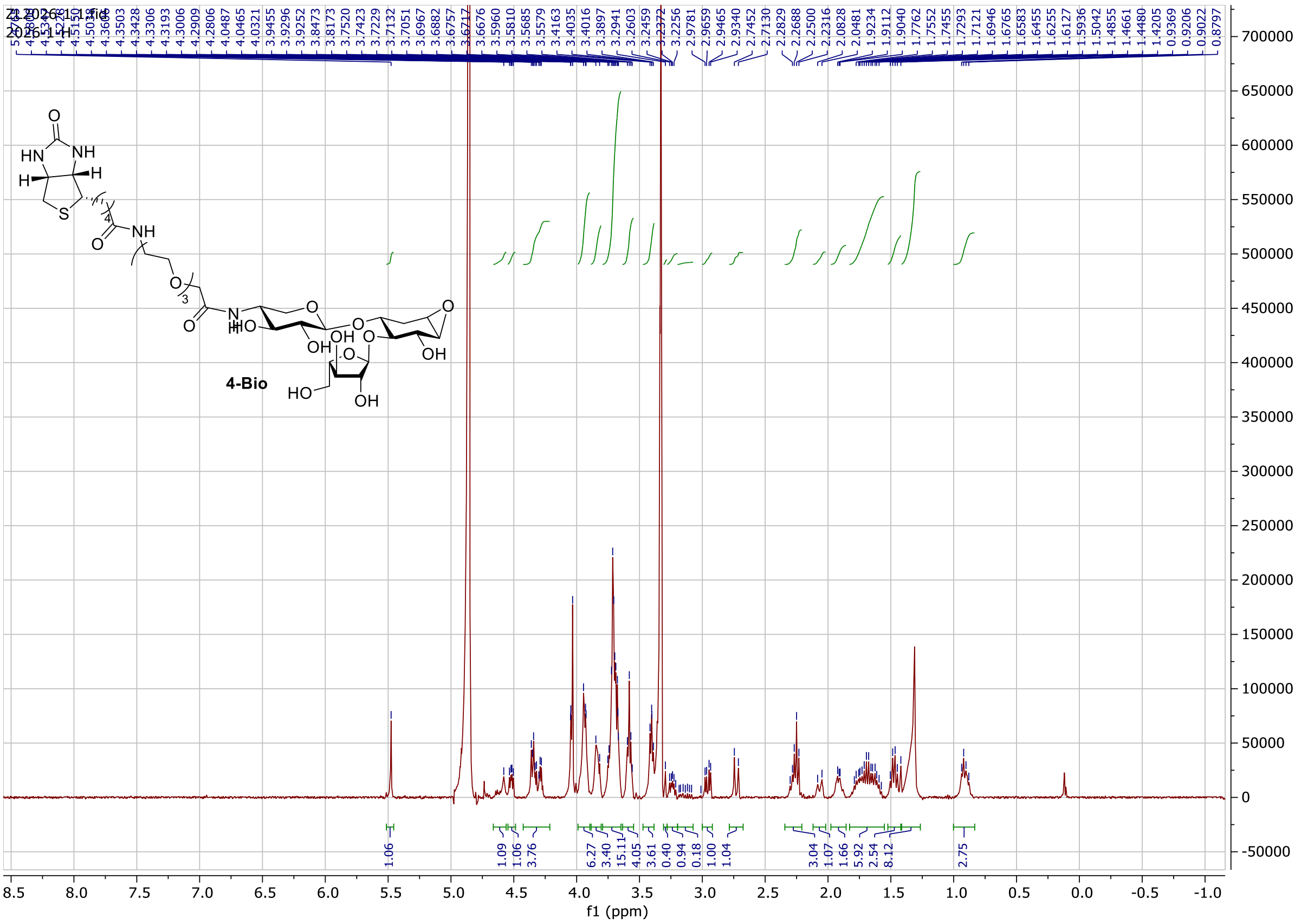

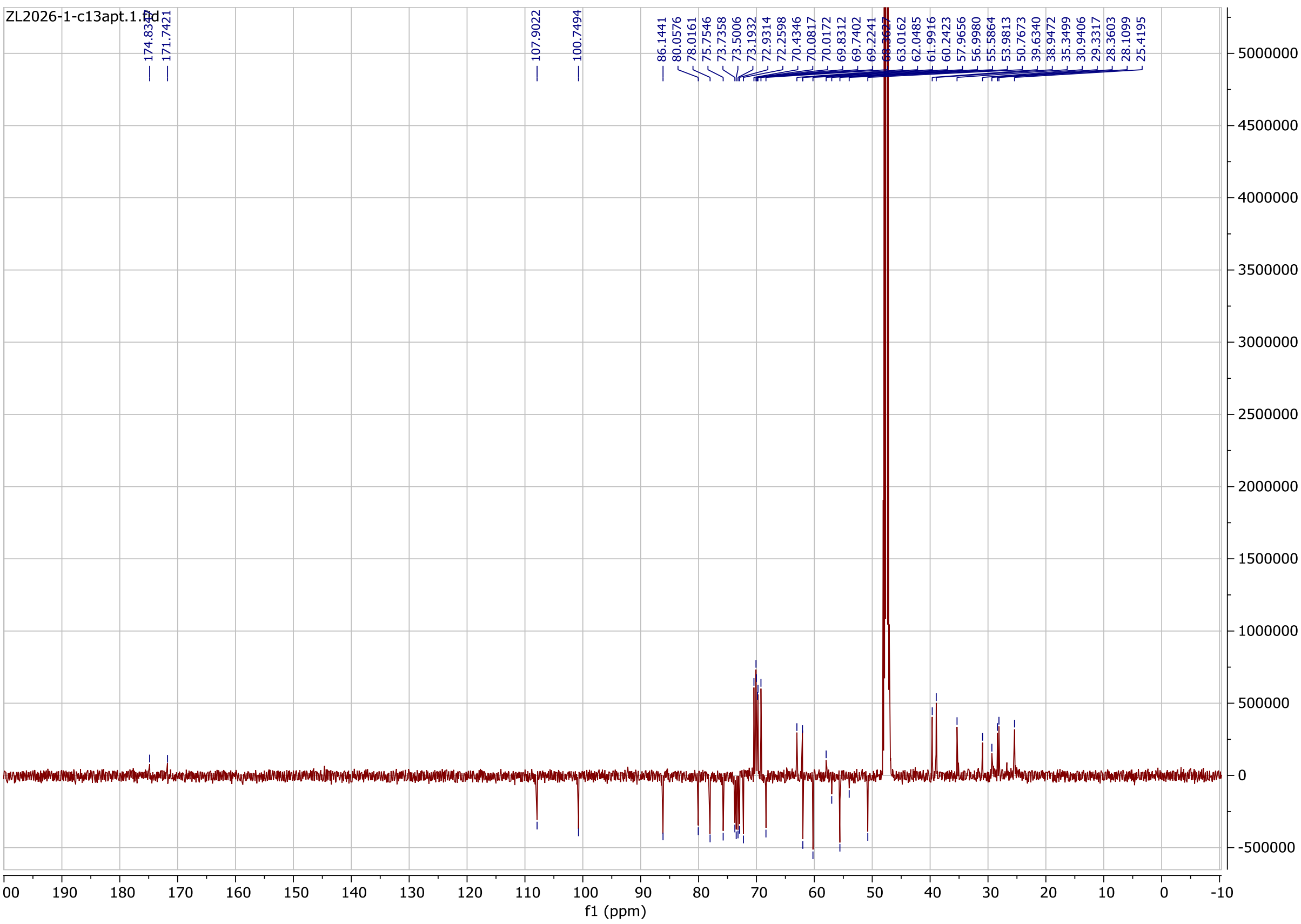

[1]H and ^{13}C of compound **4-Cy5** in MeOD

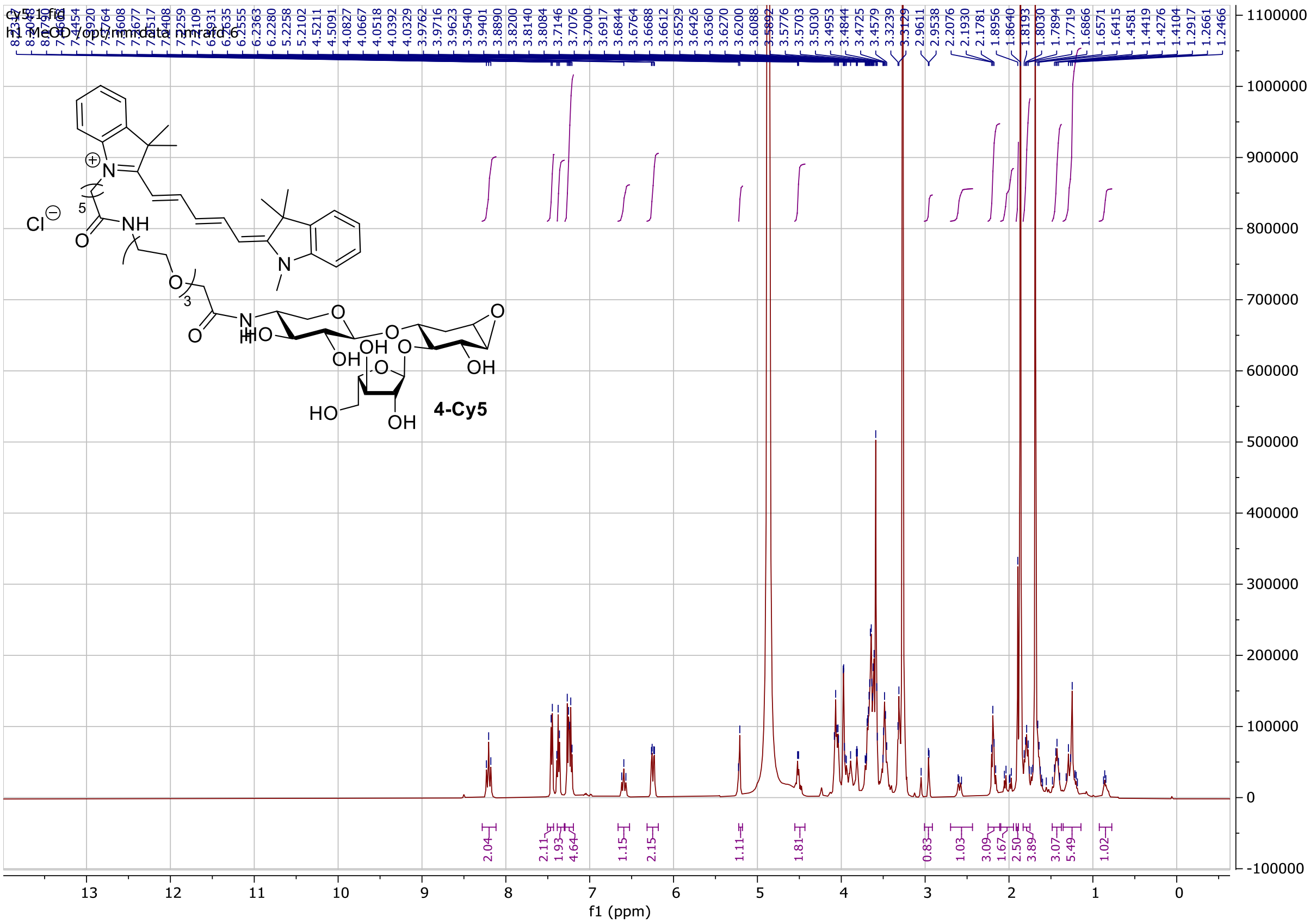

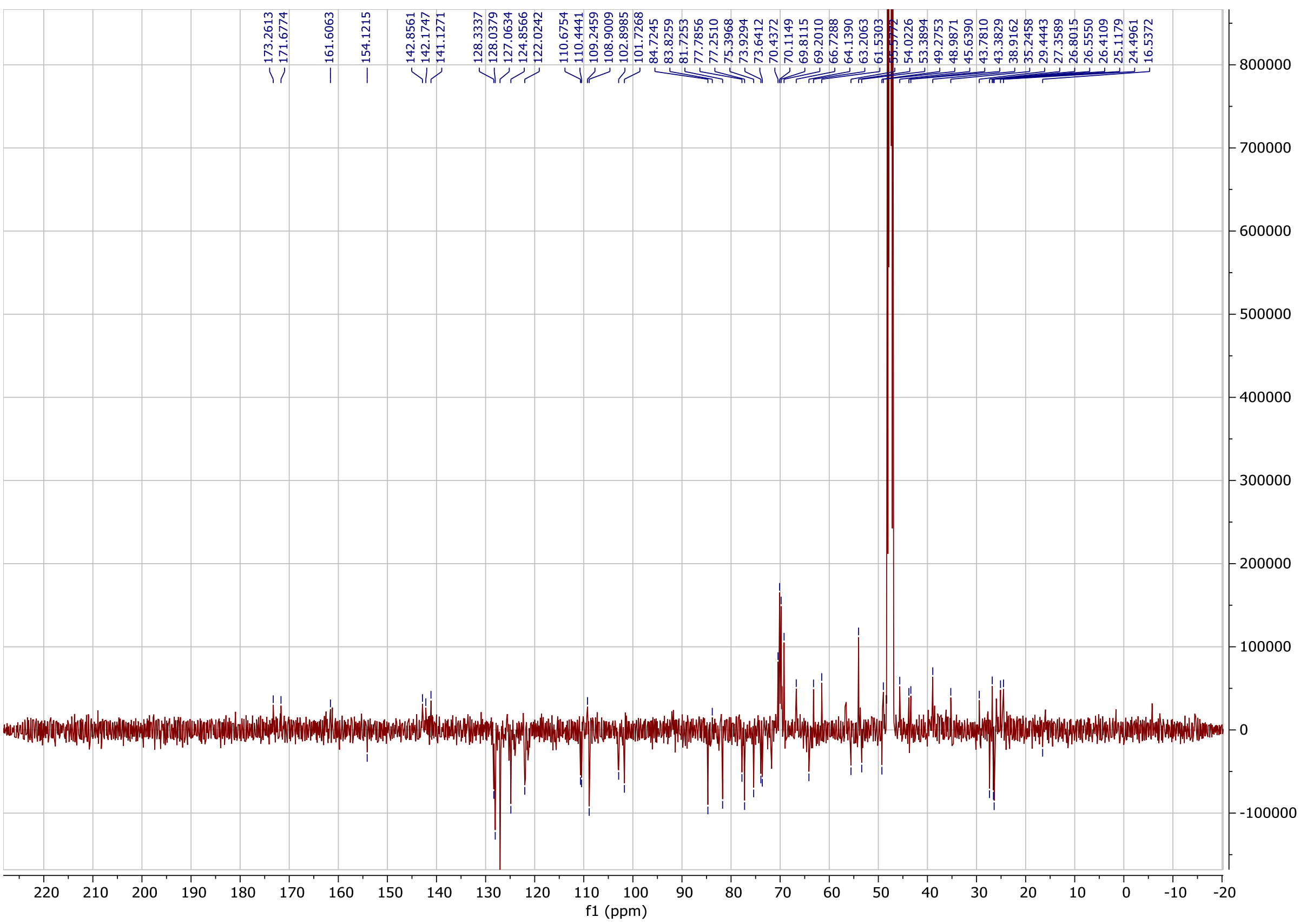

^1H and ^{13}C of compound **S8** in D_2O

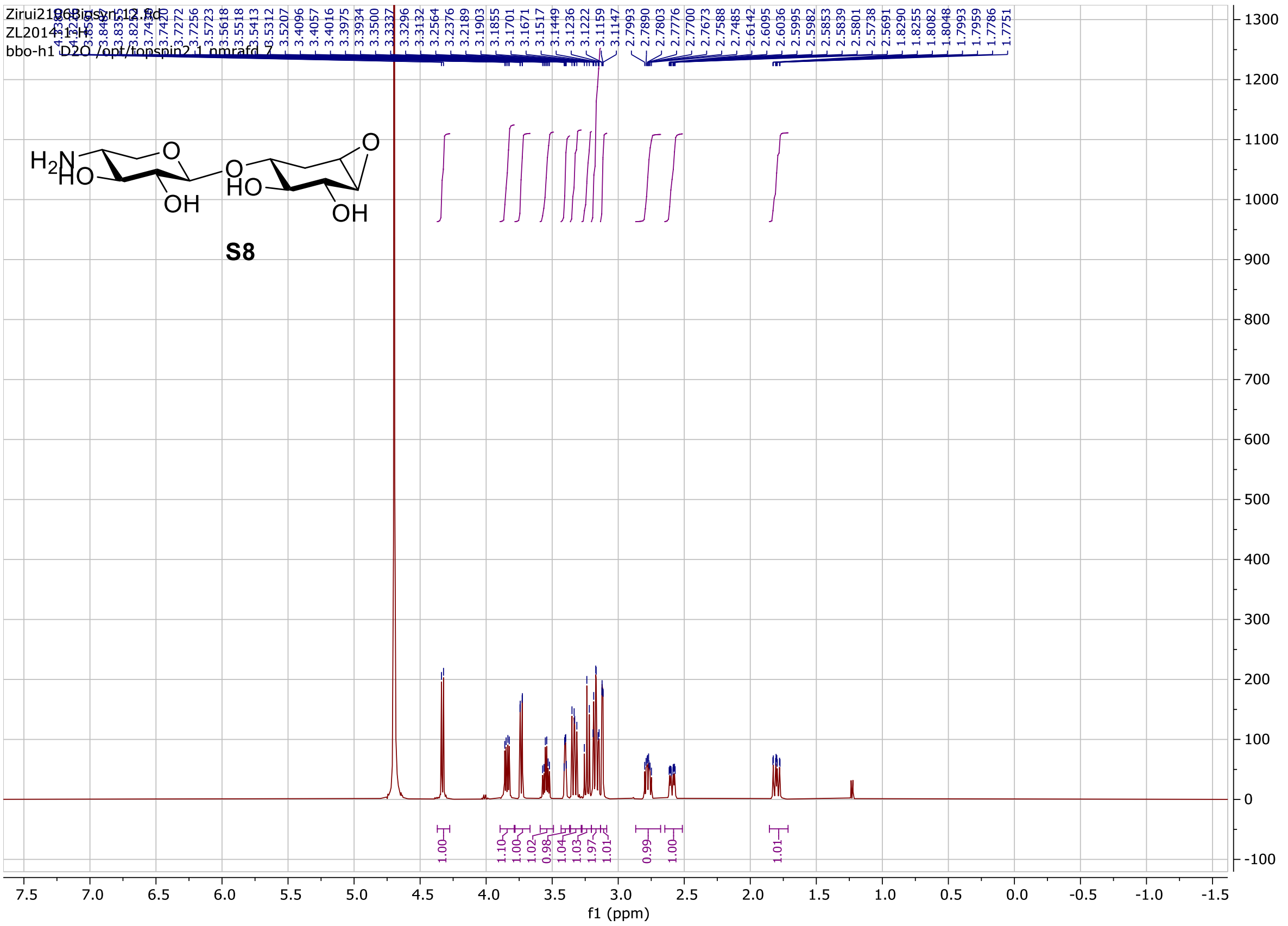

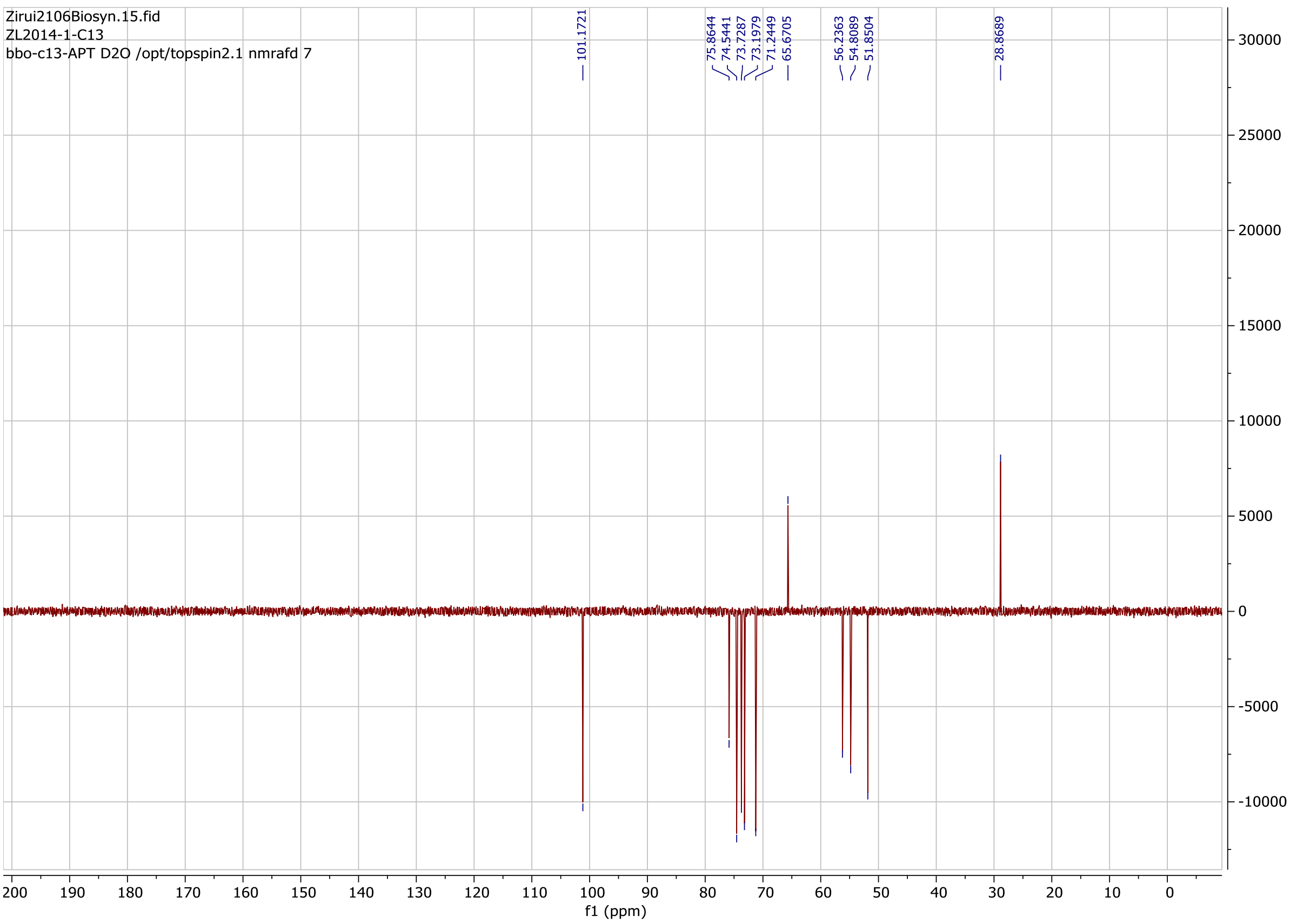

^{1}H of compound **1-Bio** in D_2O and ^{13}C in MeOD

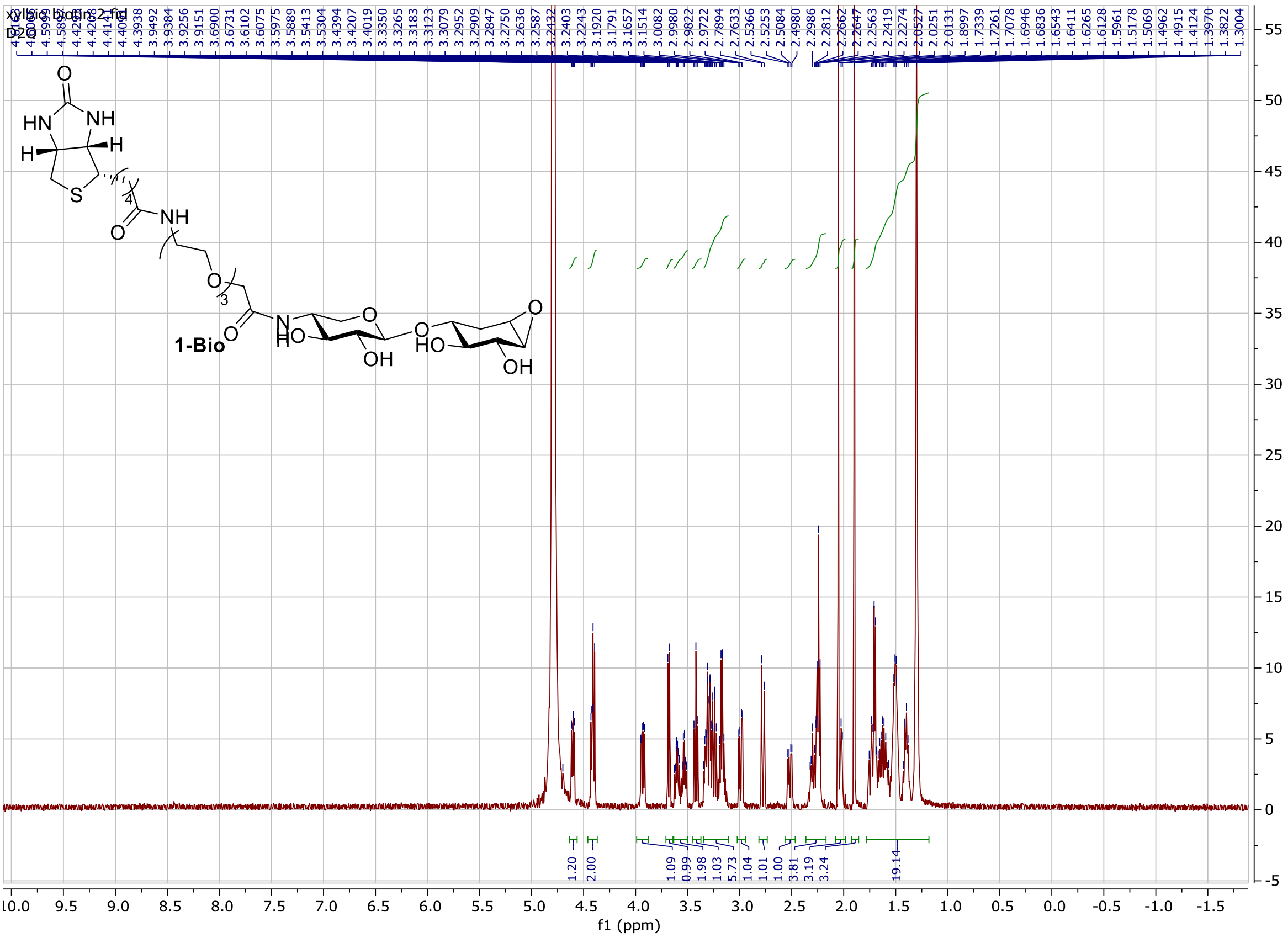

1-Bio

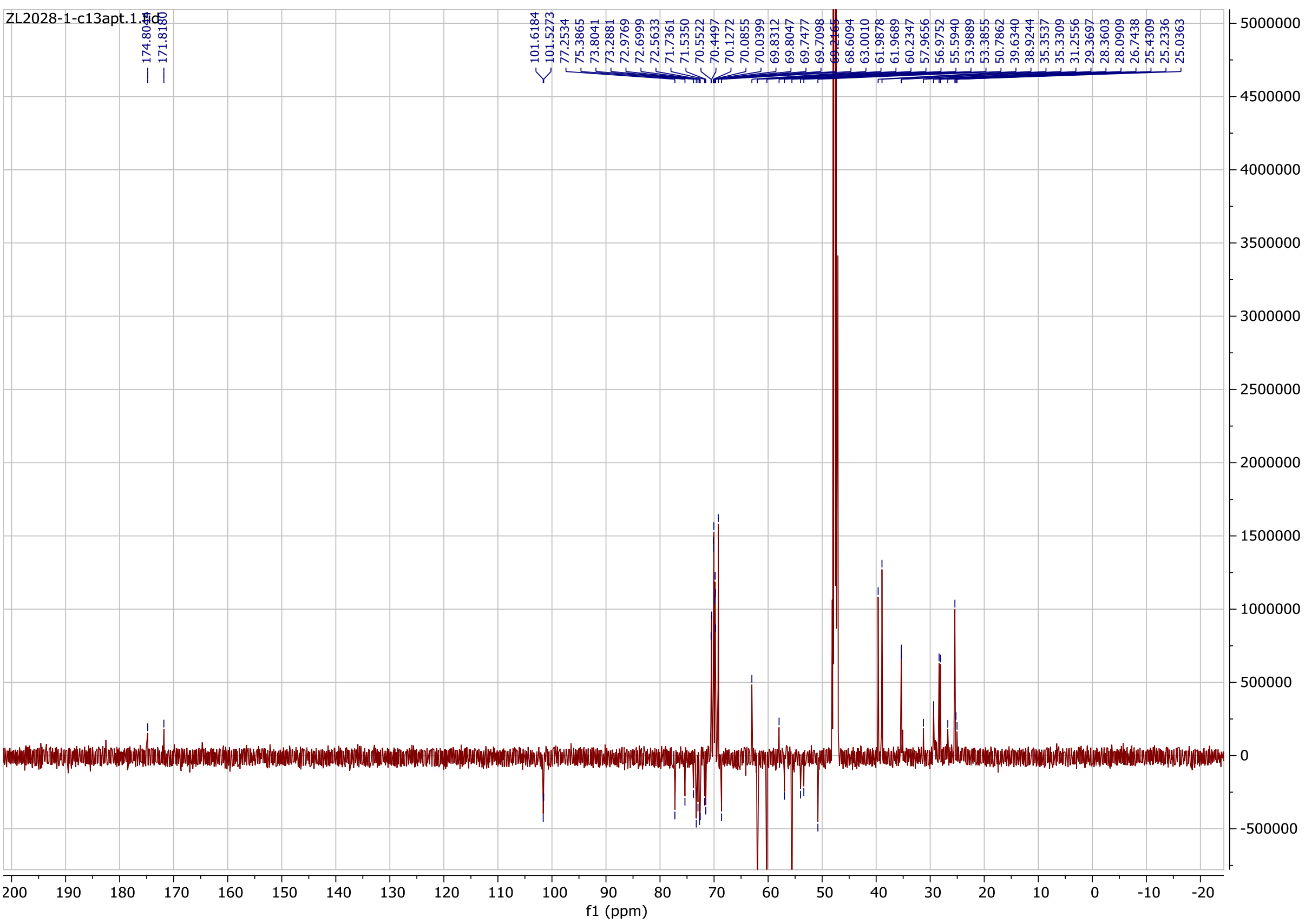

[1]H and ^{13}C of compound **1-Cy5** in D_2O

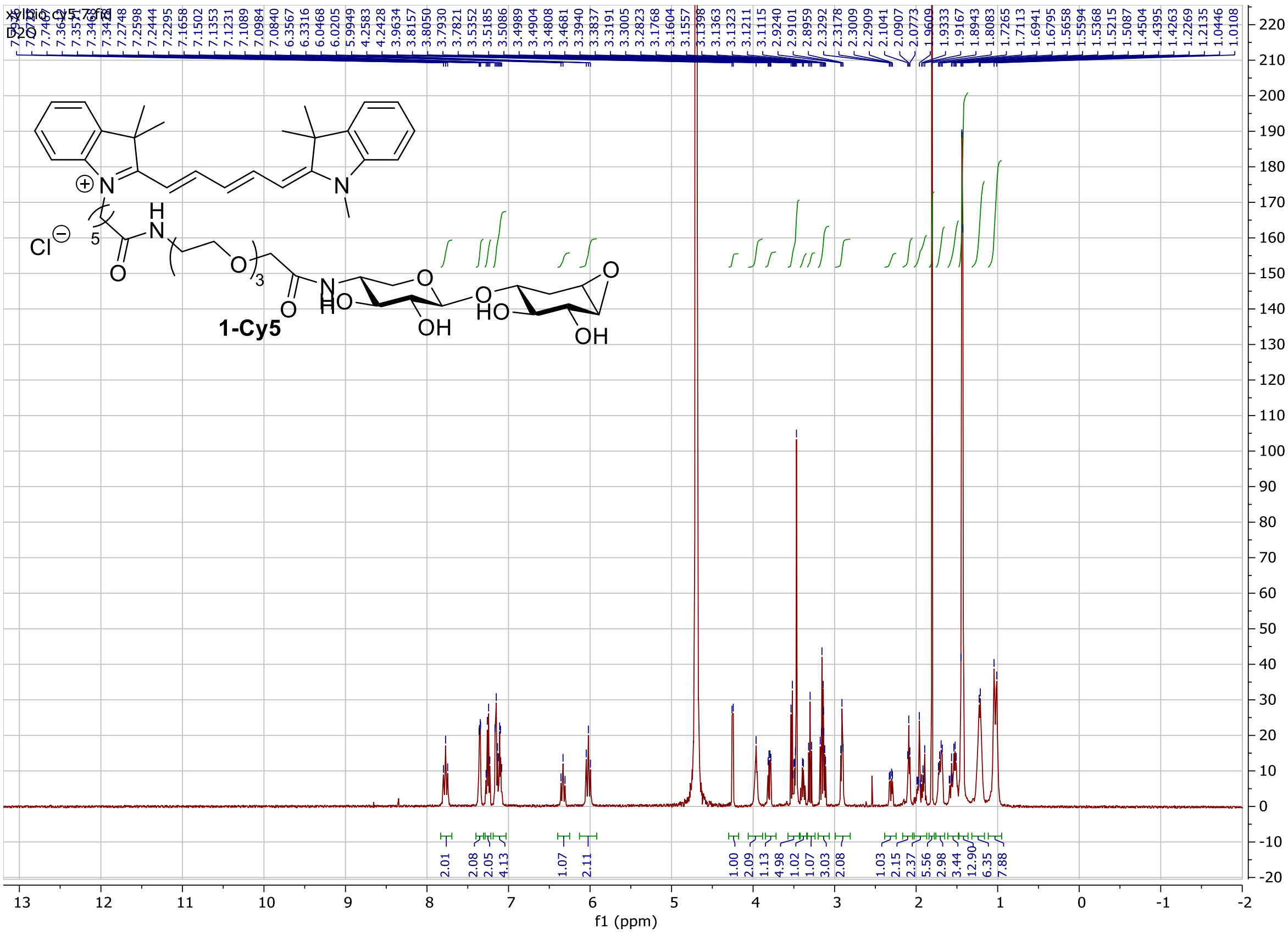

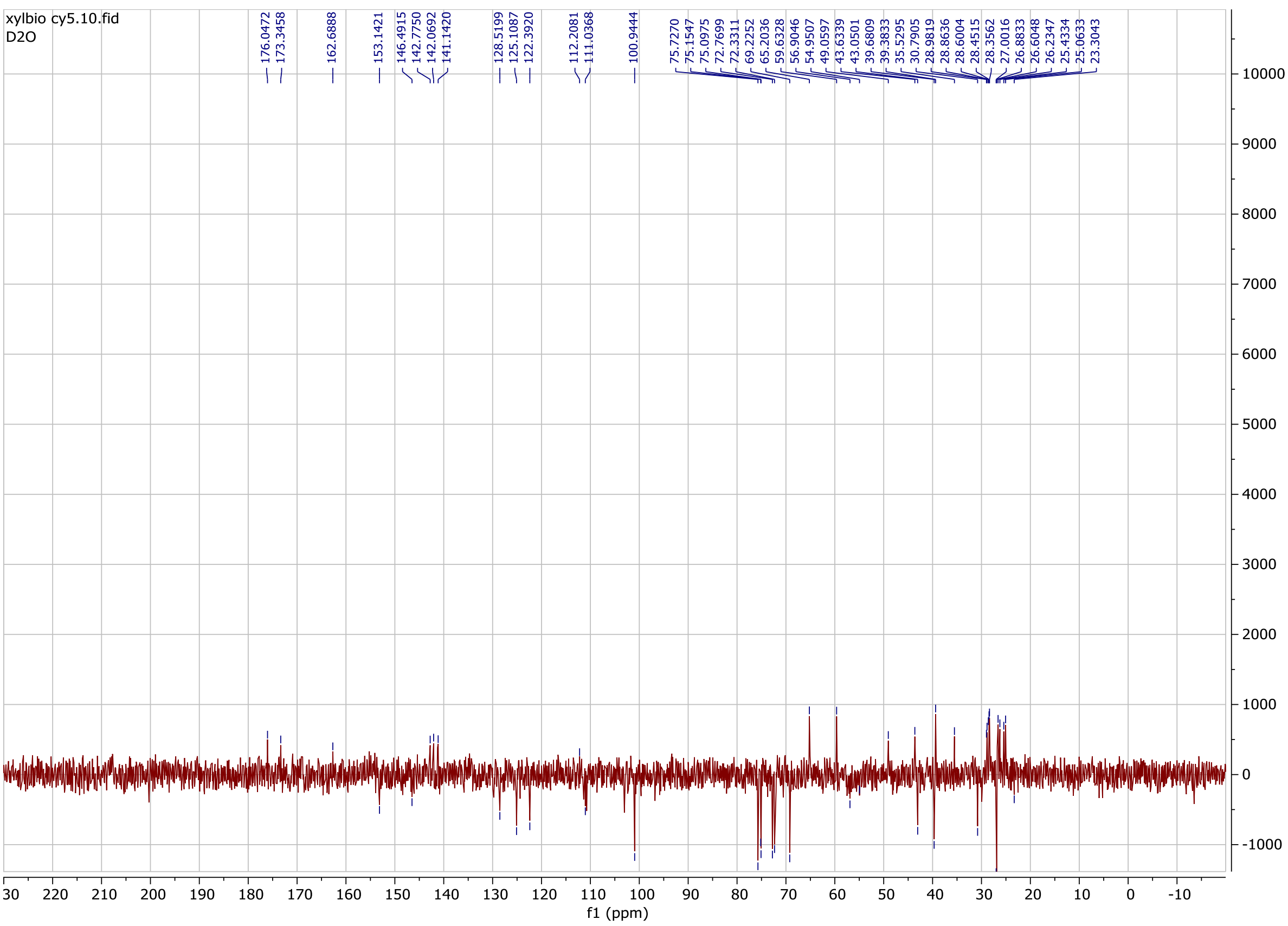

^{1}H and ^{13}C of compound **S9** in $CDCl_3$

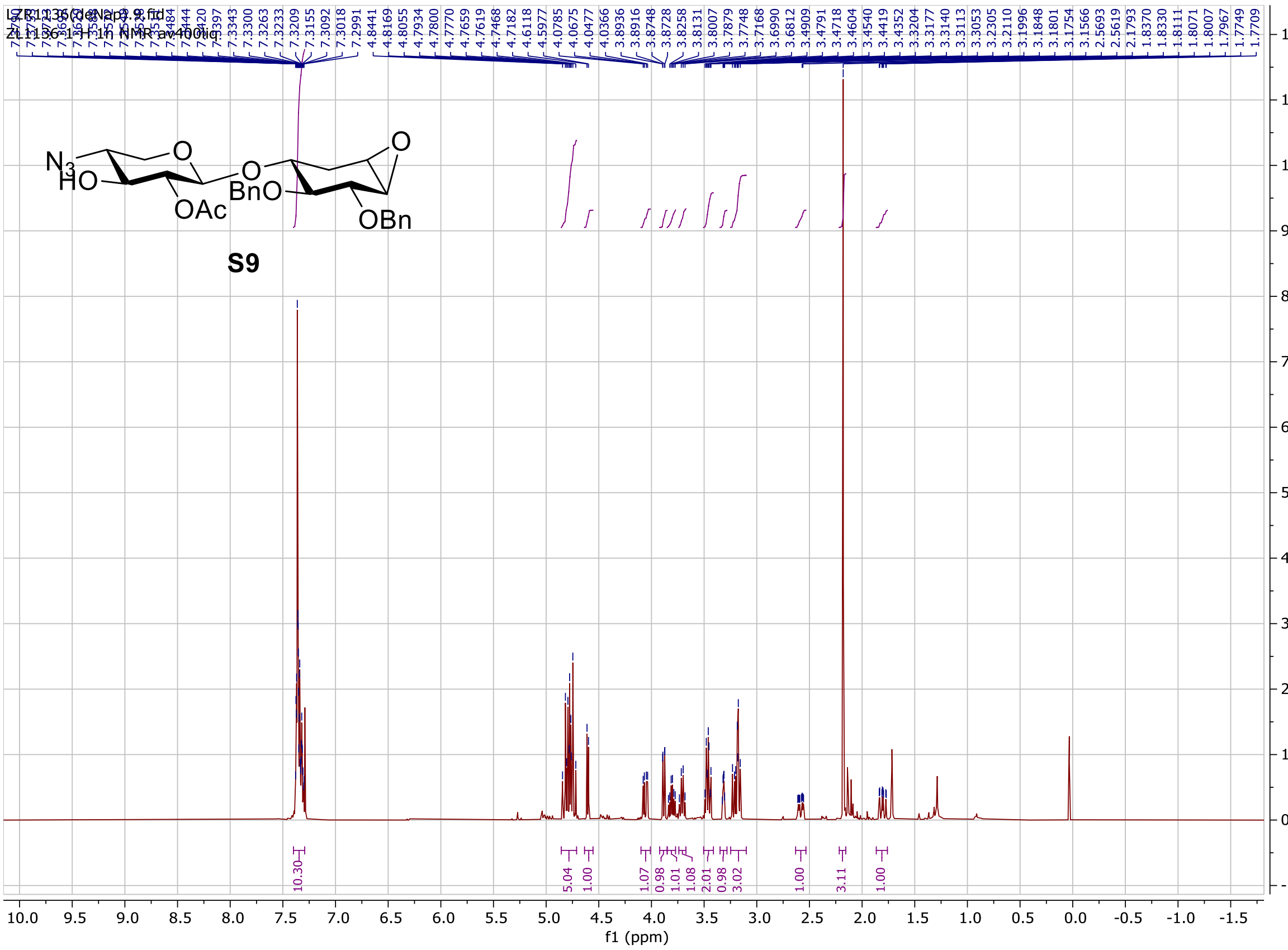

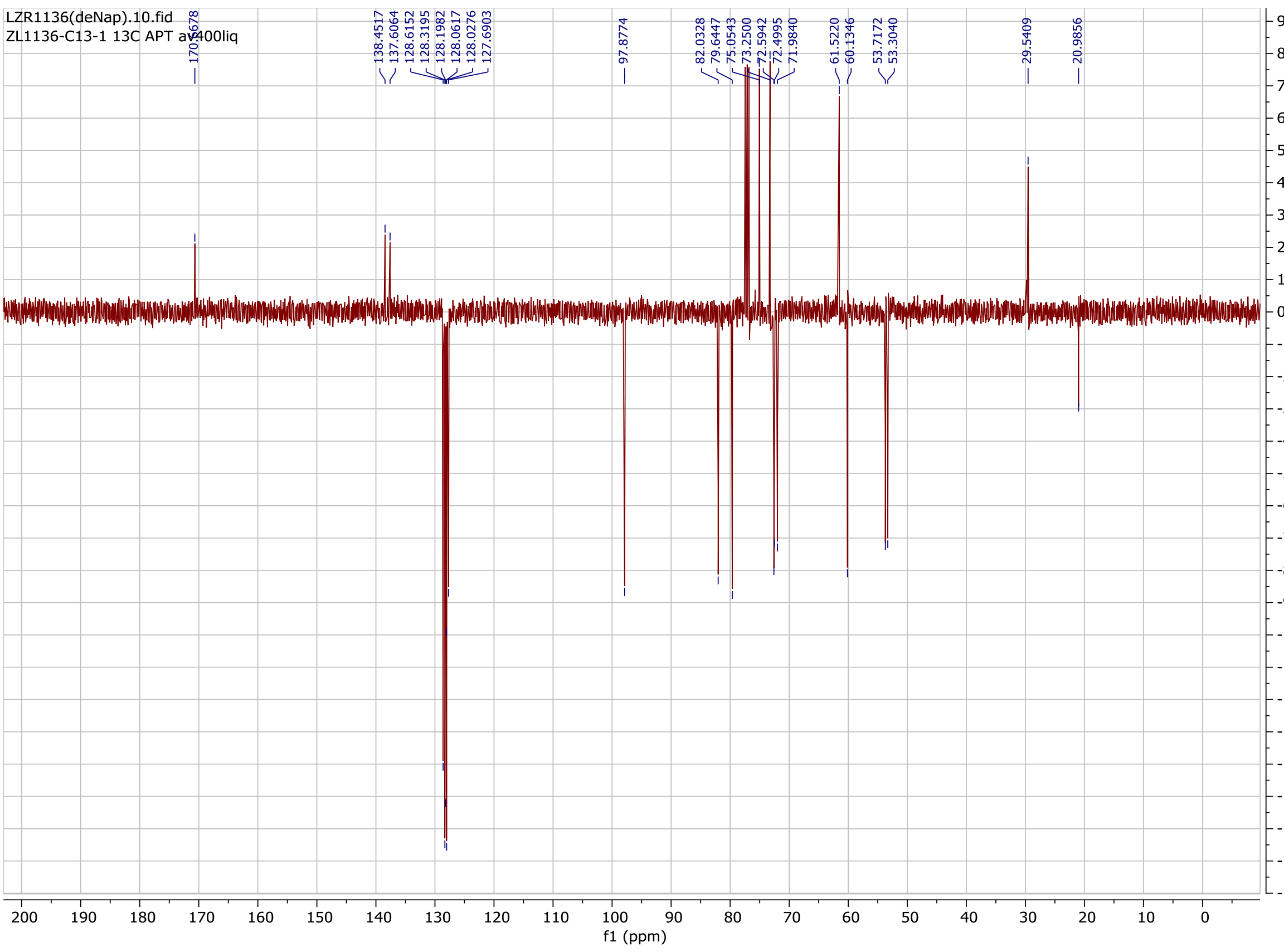

[1]H and ^{13}C of compound **S10** in $CDCl_3$

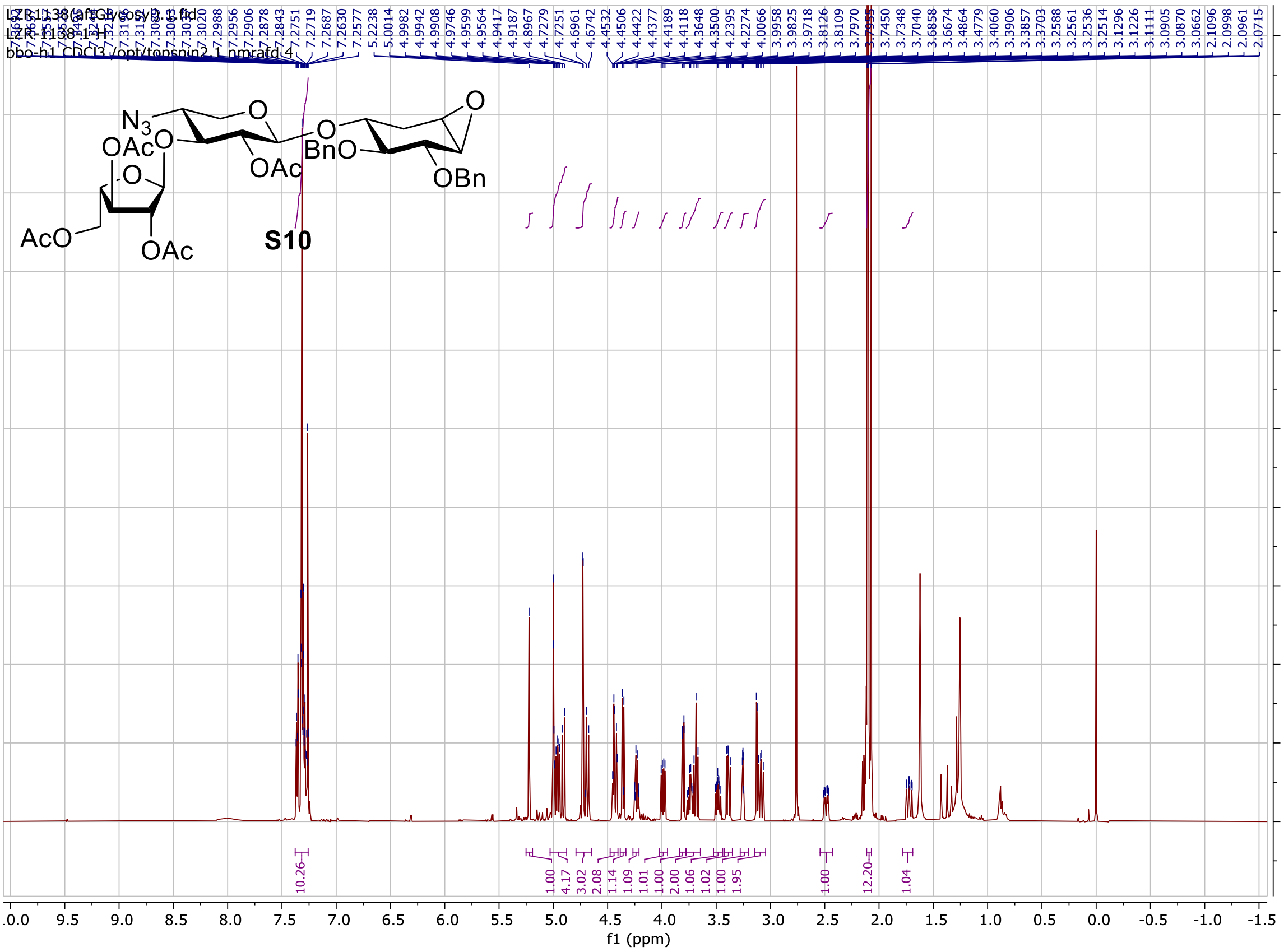

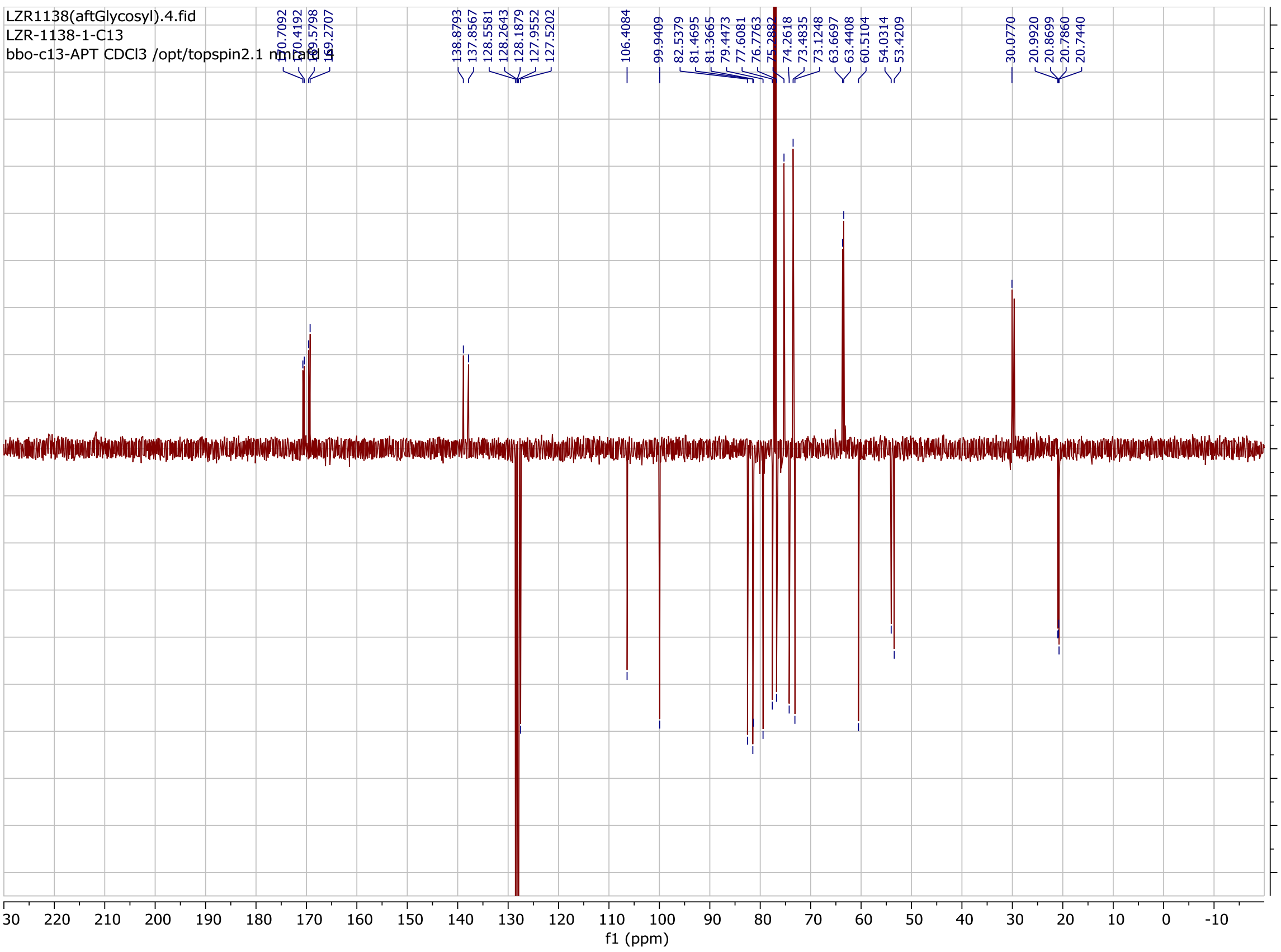

[1]H and ^{13}C of compound **S11** in $CDCl_3$

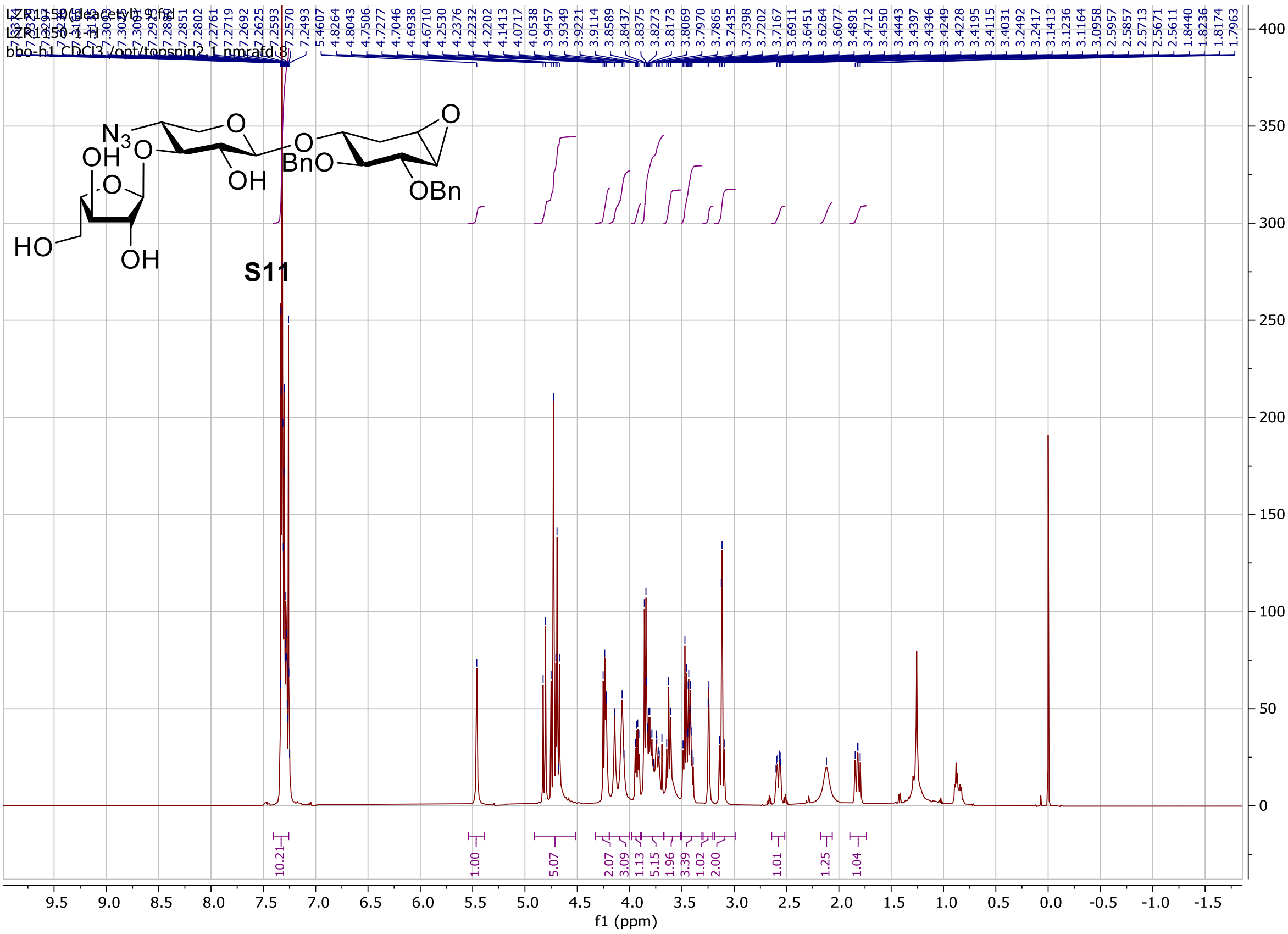

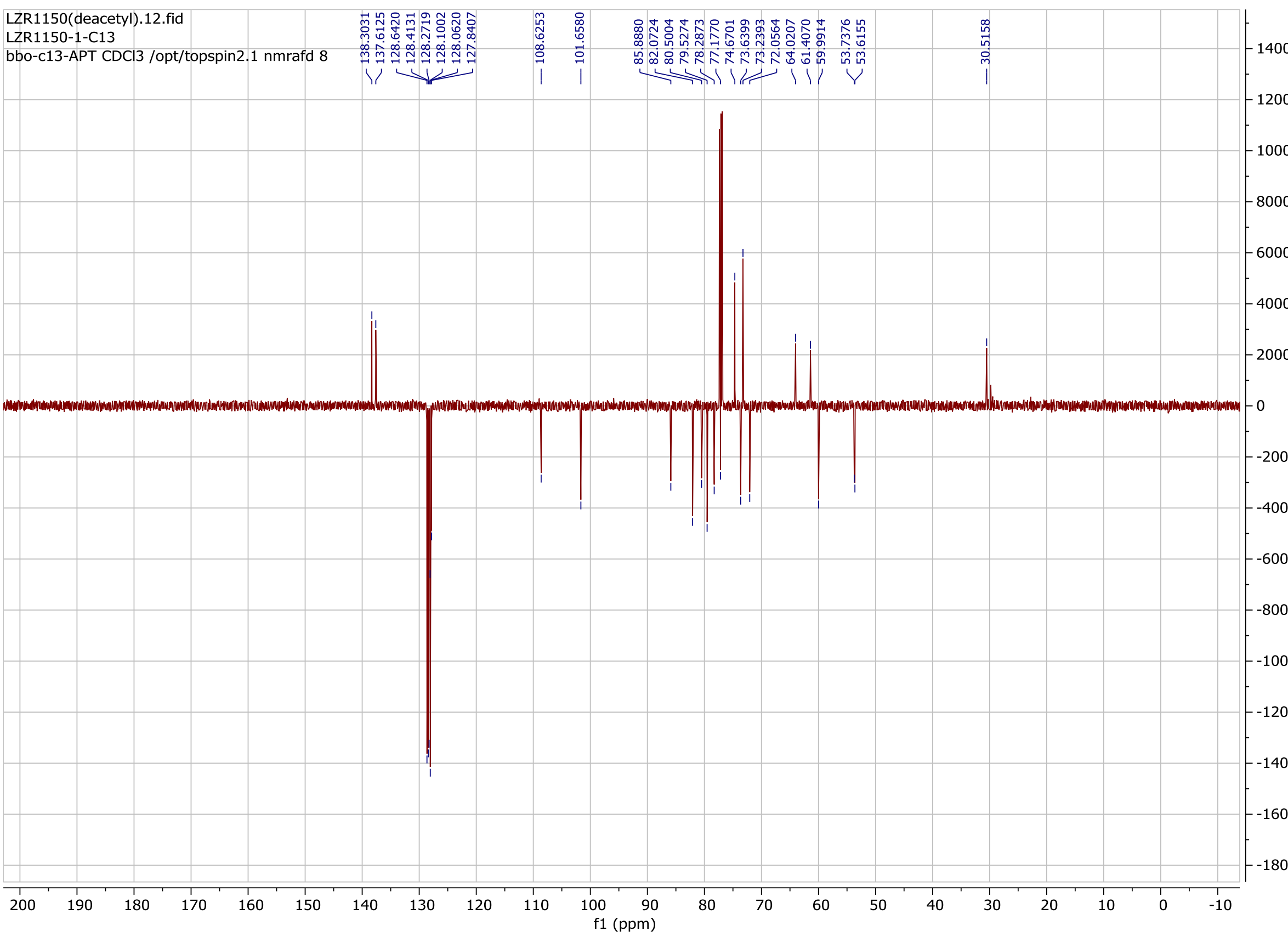

^{1}H and ^{13}C of compound **S12** in D_2O

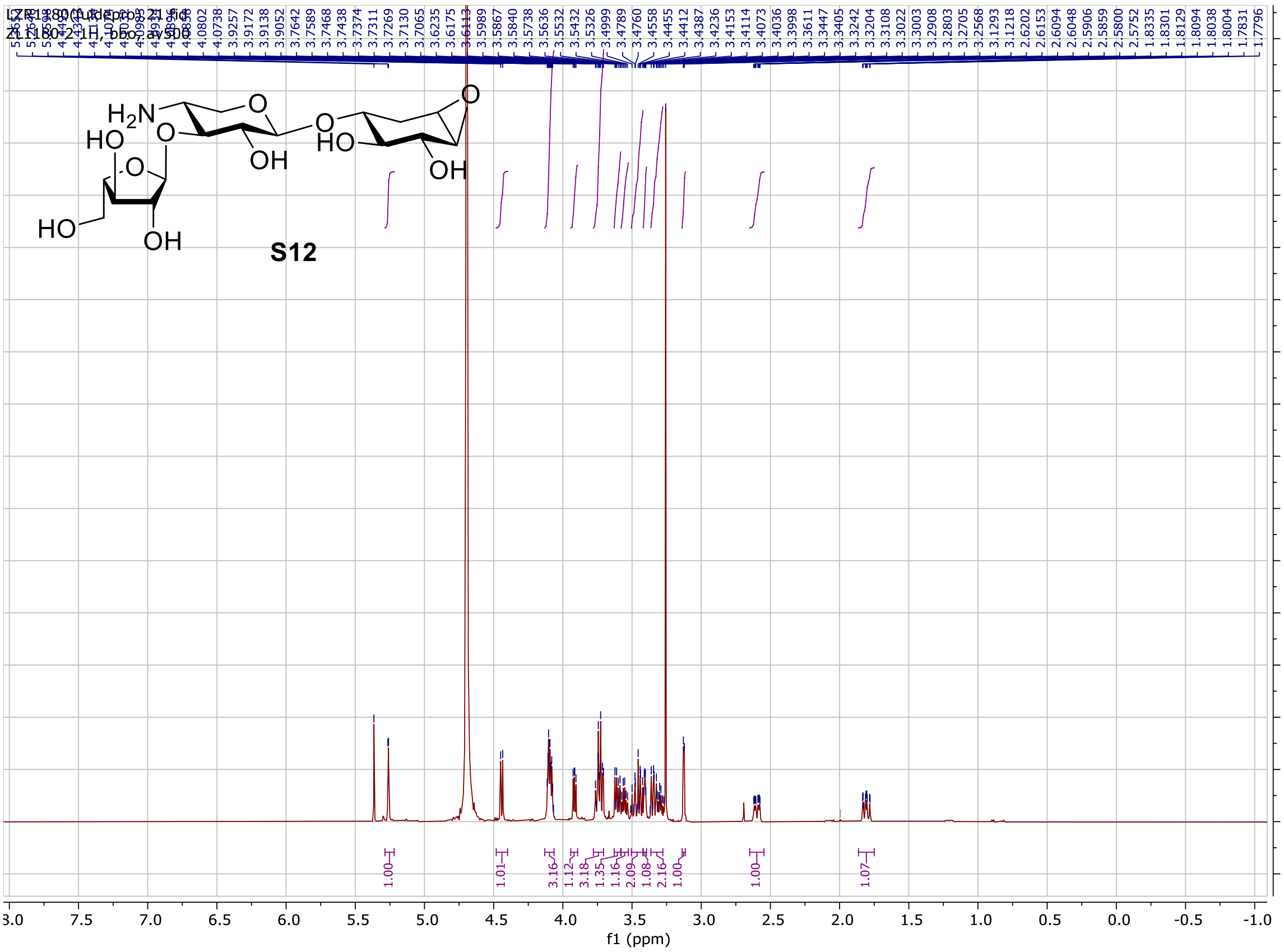

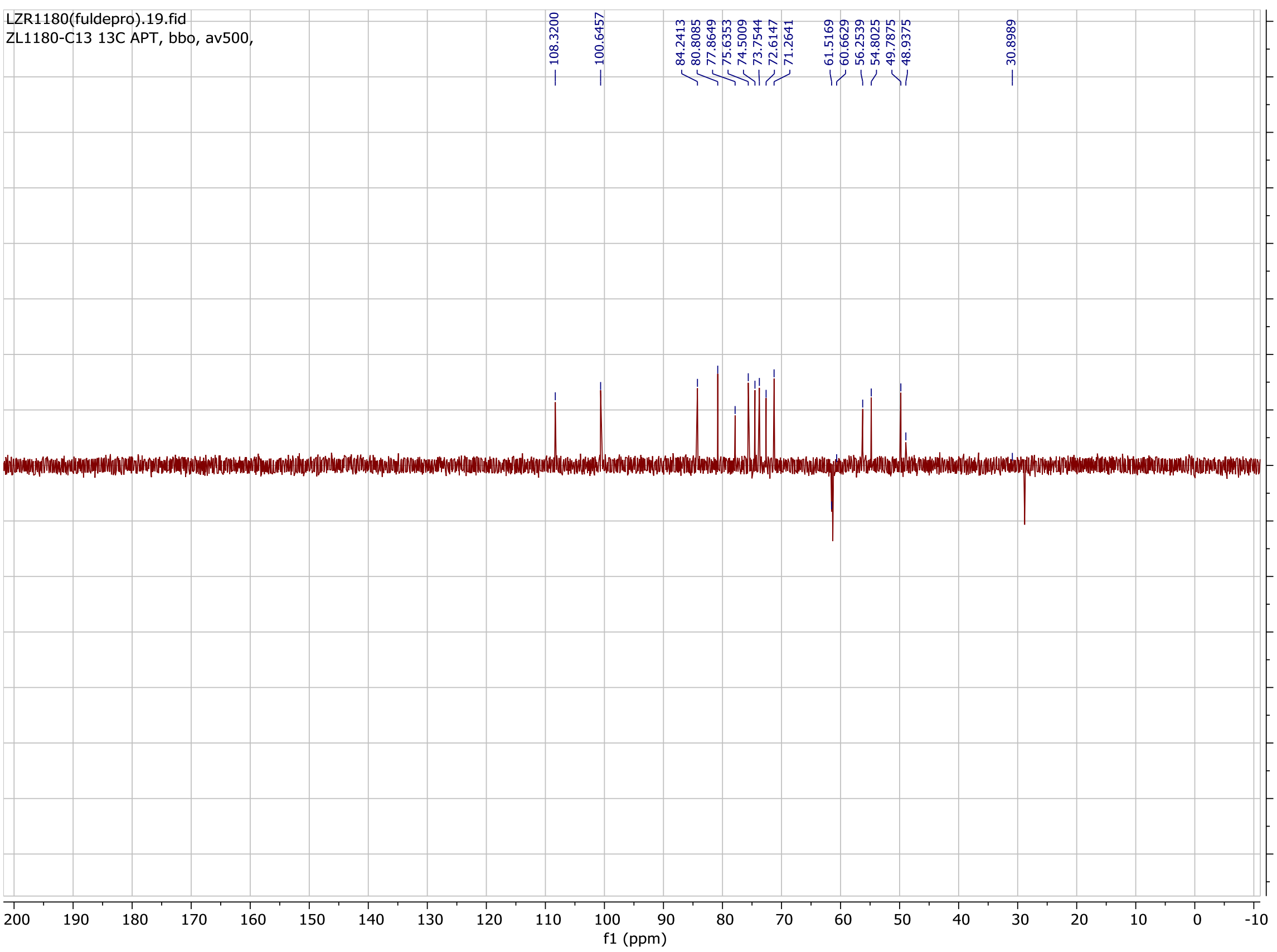

[1]H and ^{13}C of compound **3-Bio** in D_2O

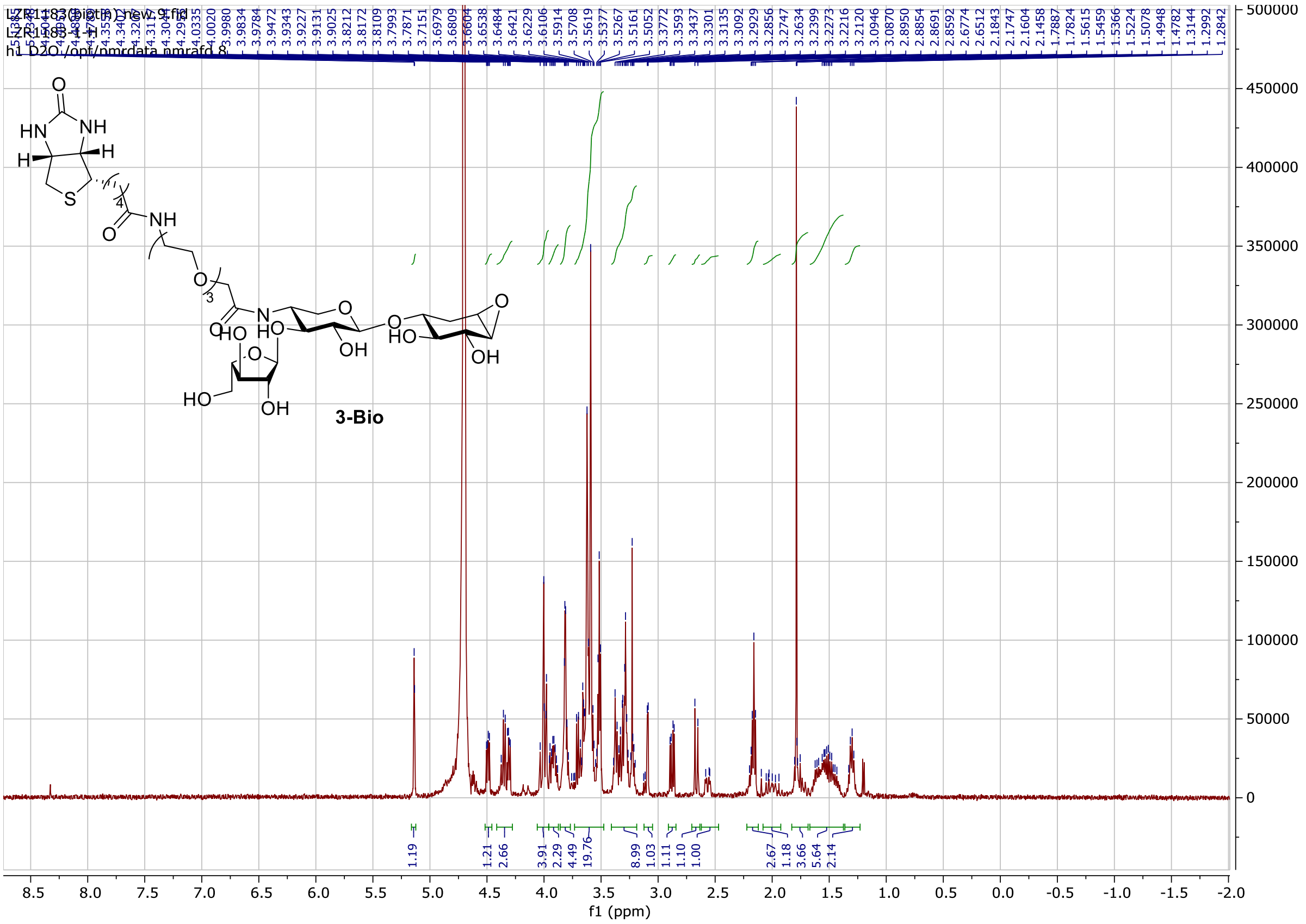

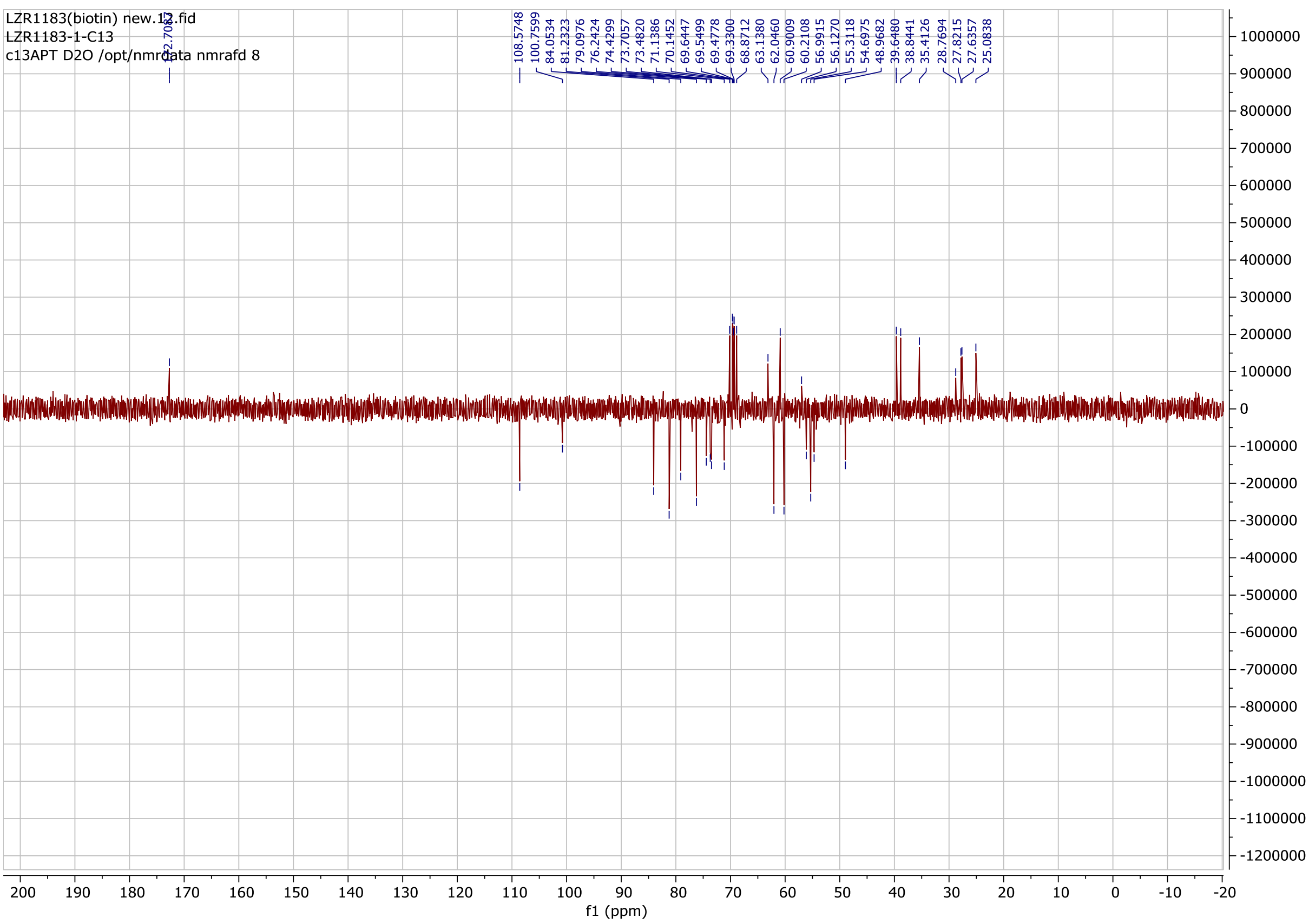

^{1}H of compound **3-Cy5** in D_2O and ^{13}C in MeOD

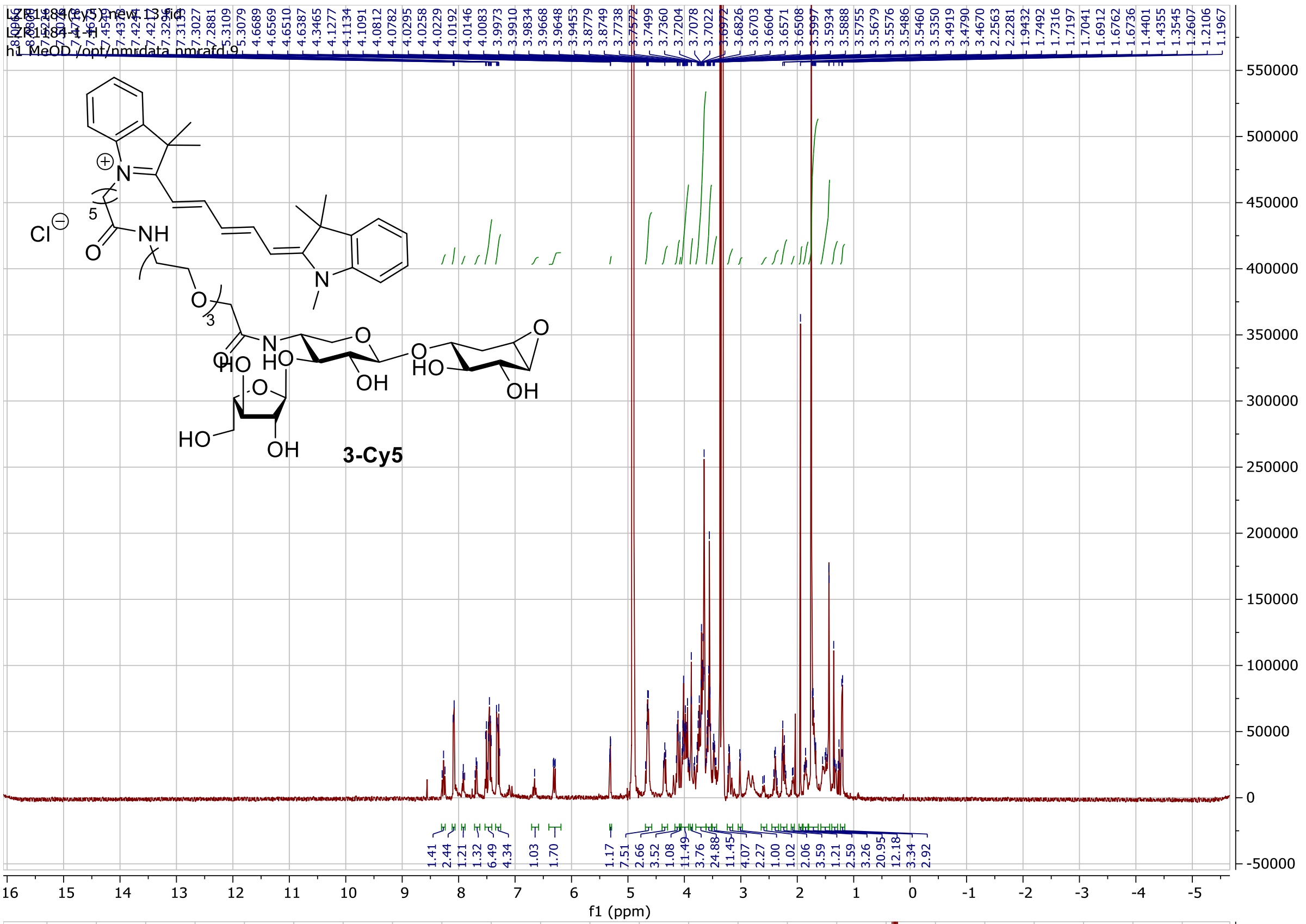

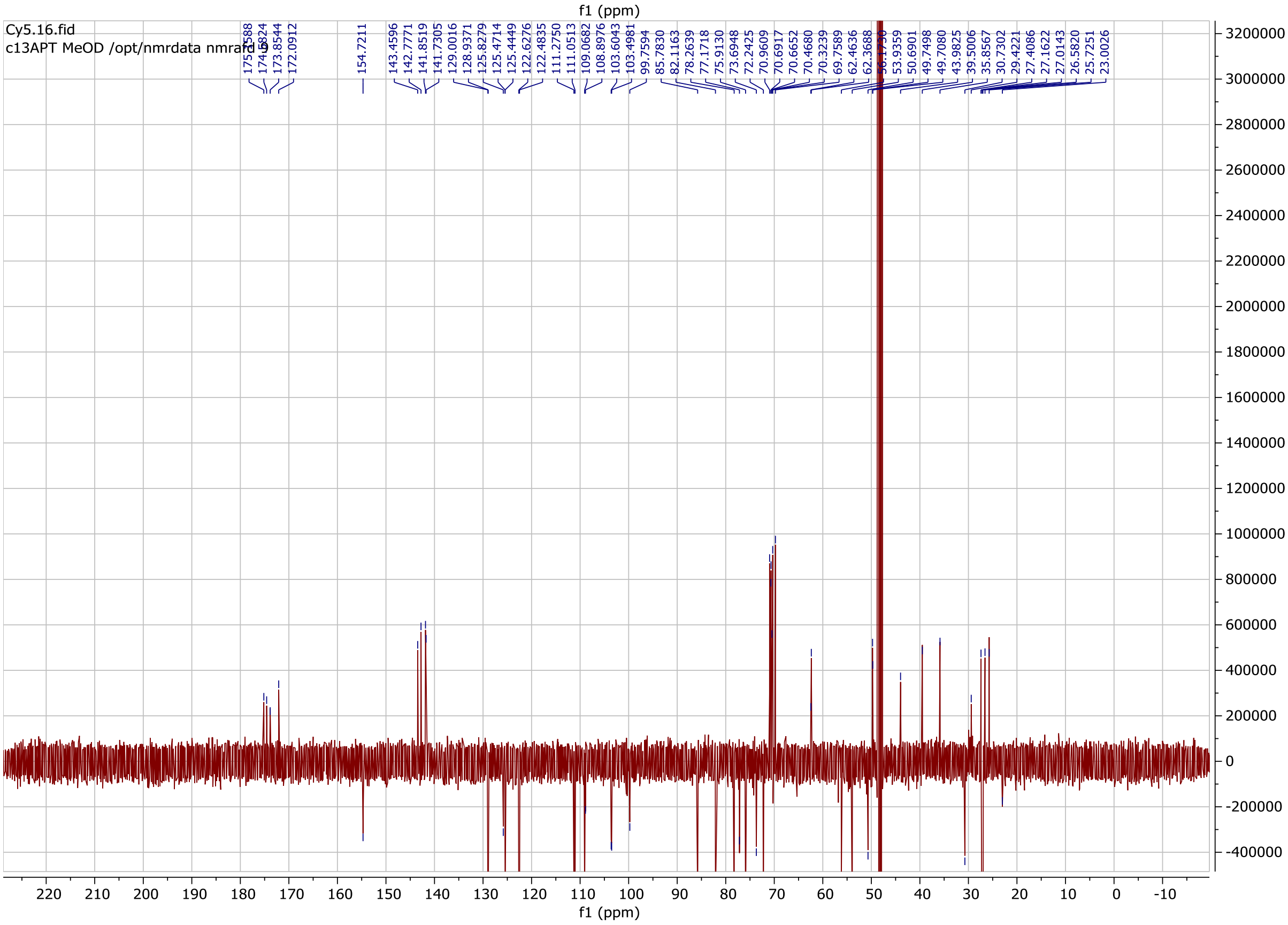

[1]H and ^{13}C of compound **S13** in $CDCl_3$

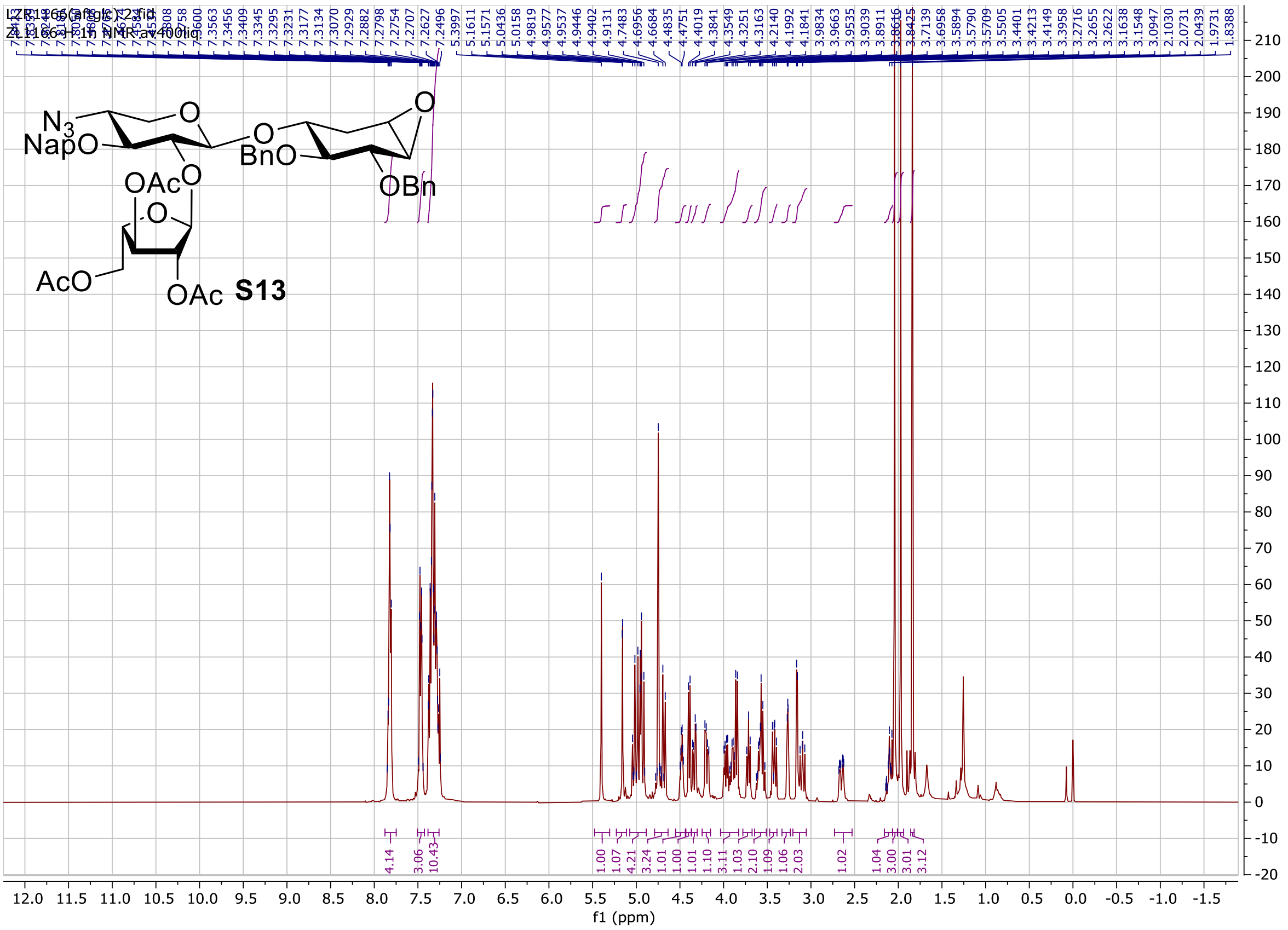

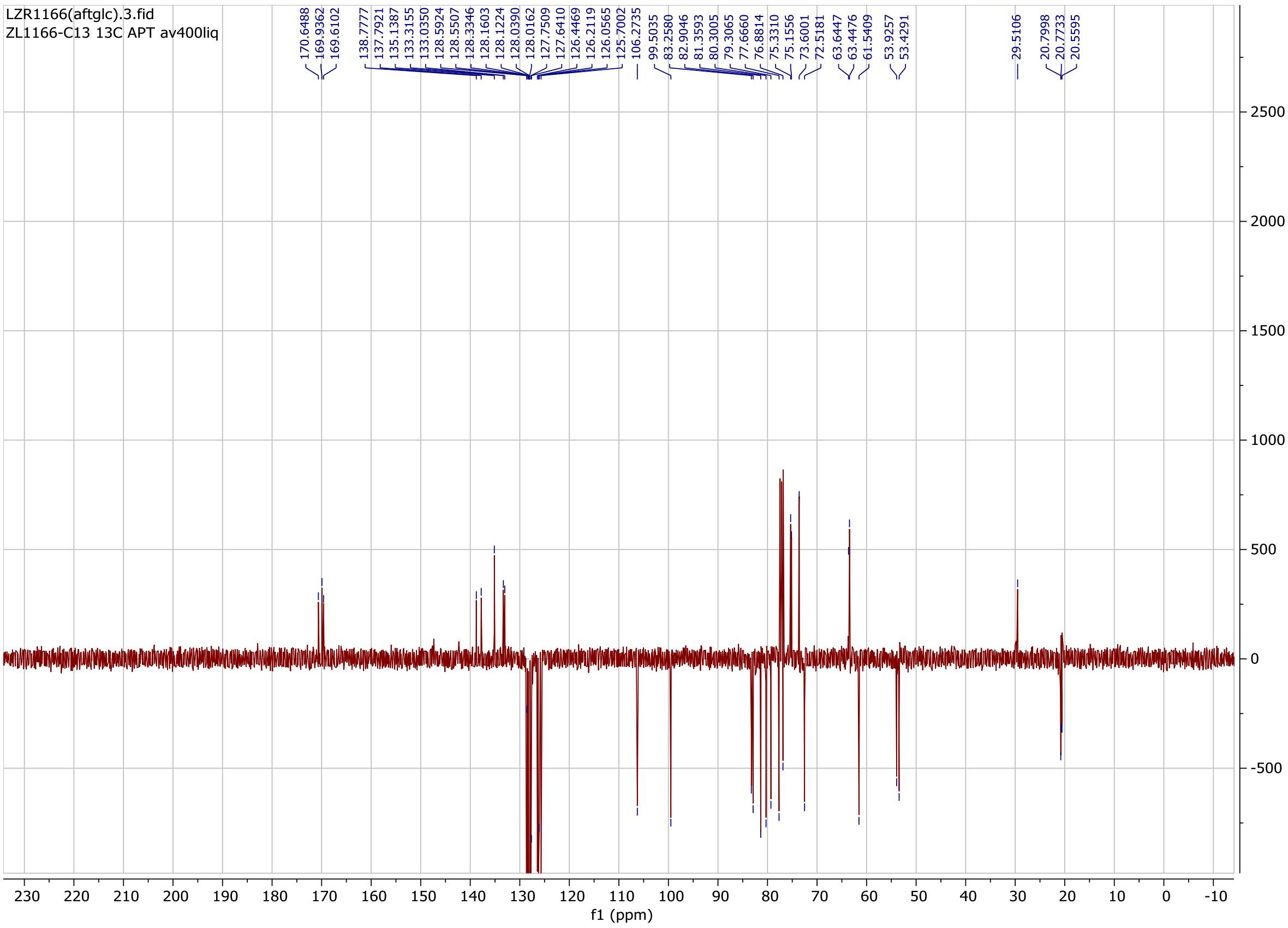

[1]H and ^{13}C of compound **S14** in $CDCl_3$

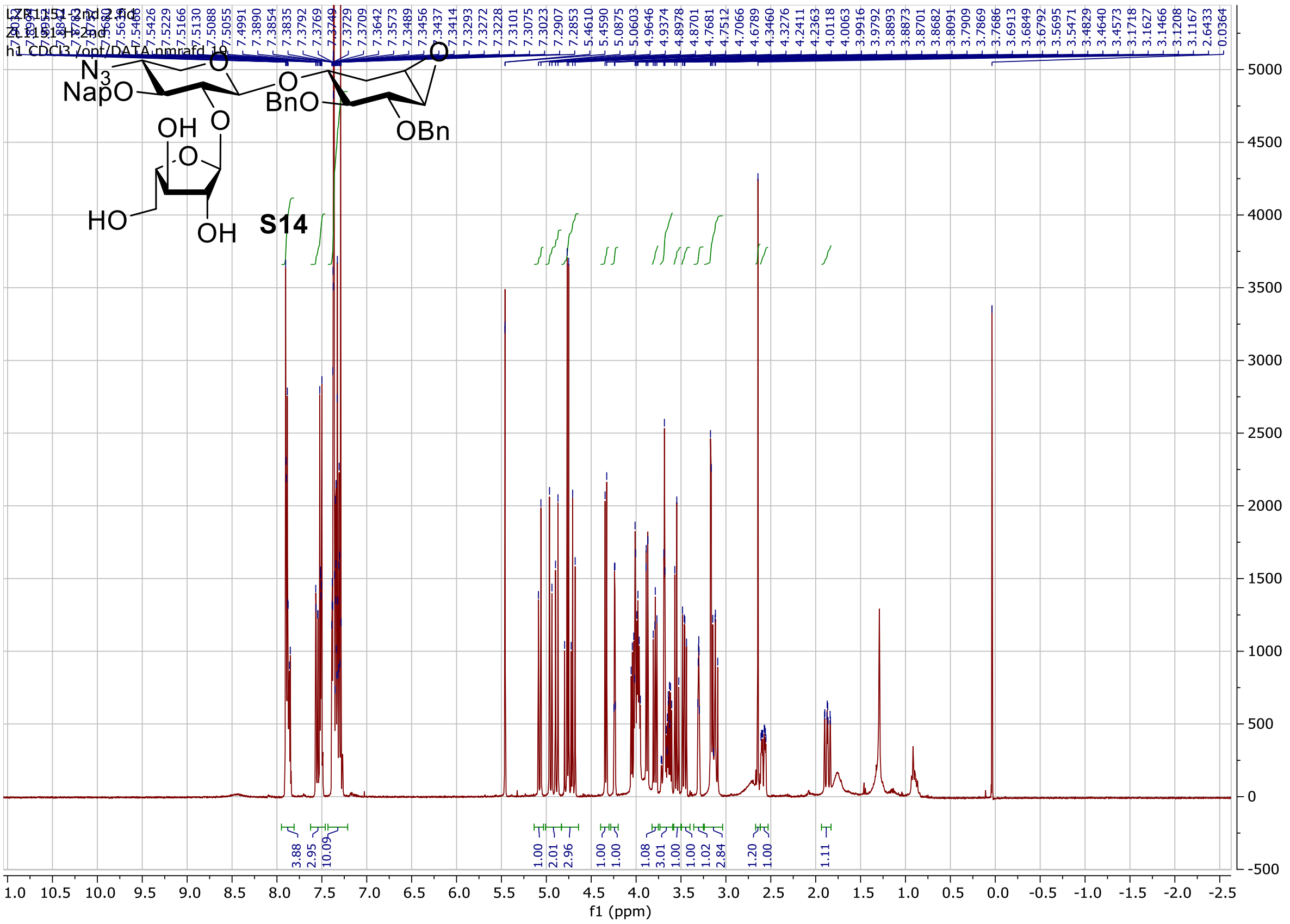

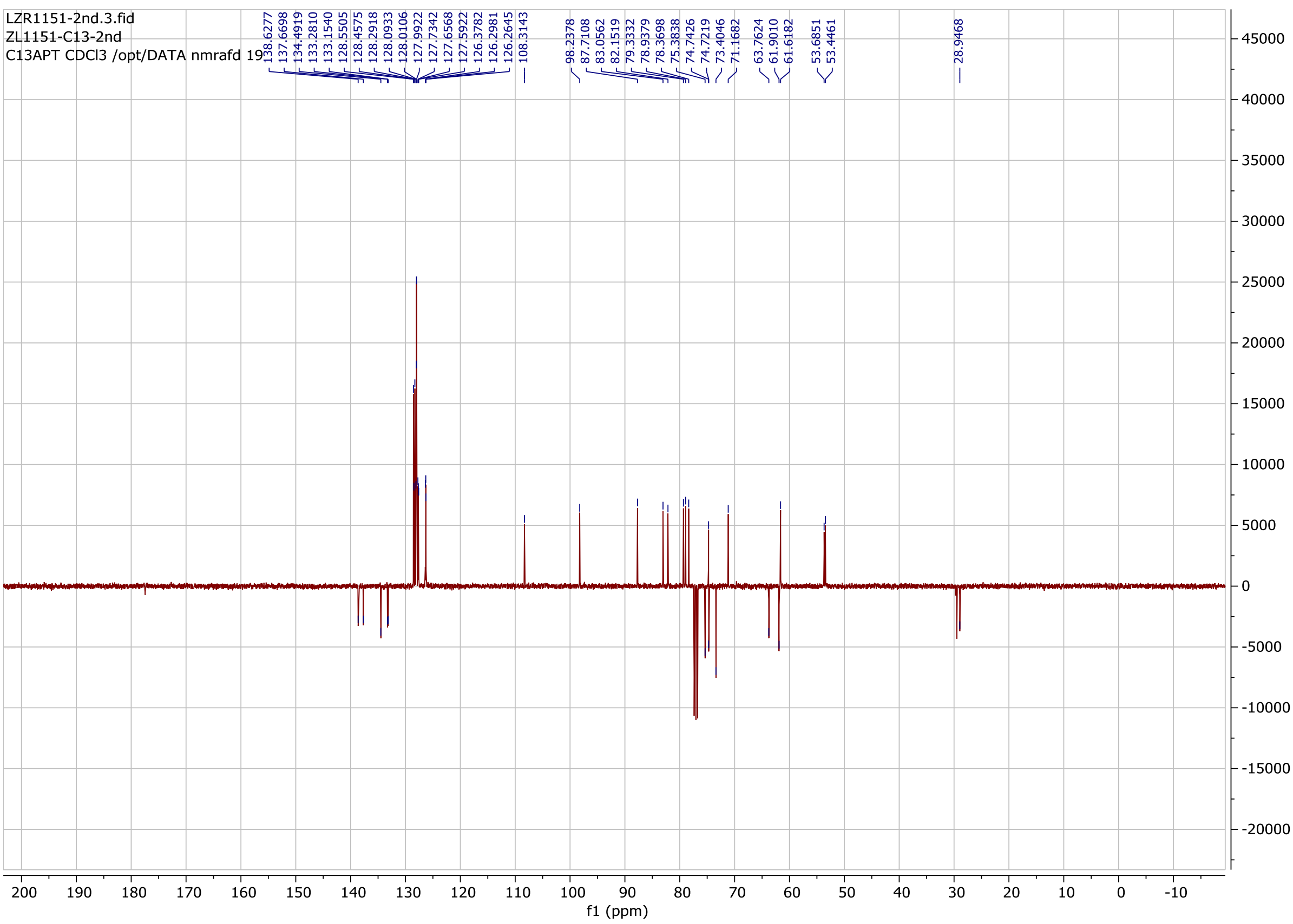

[1]H and ^{13}C of compound **S15** in D_2O

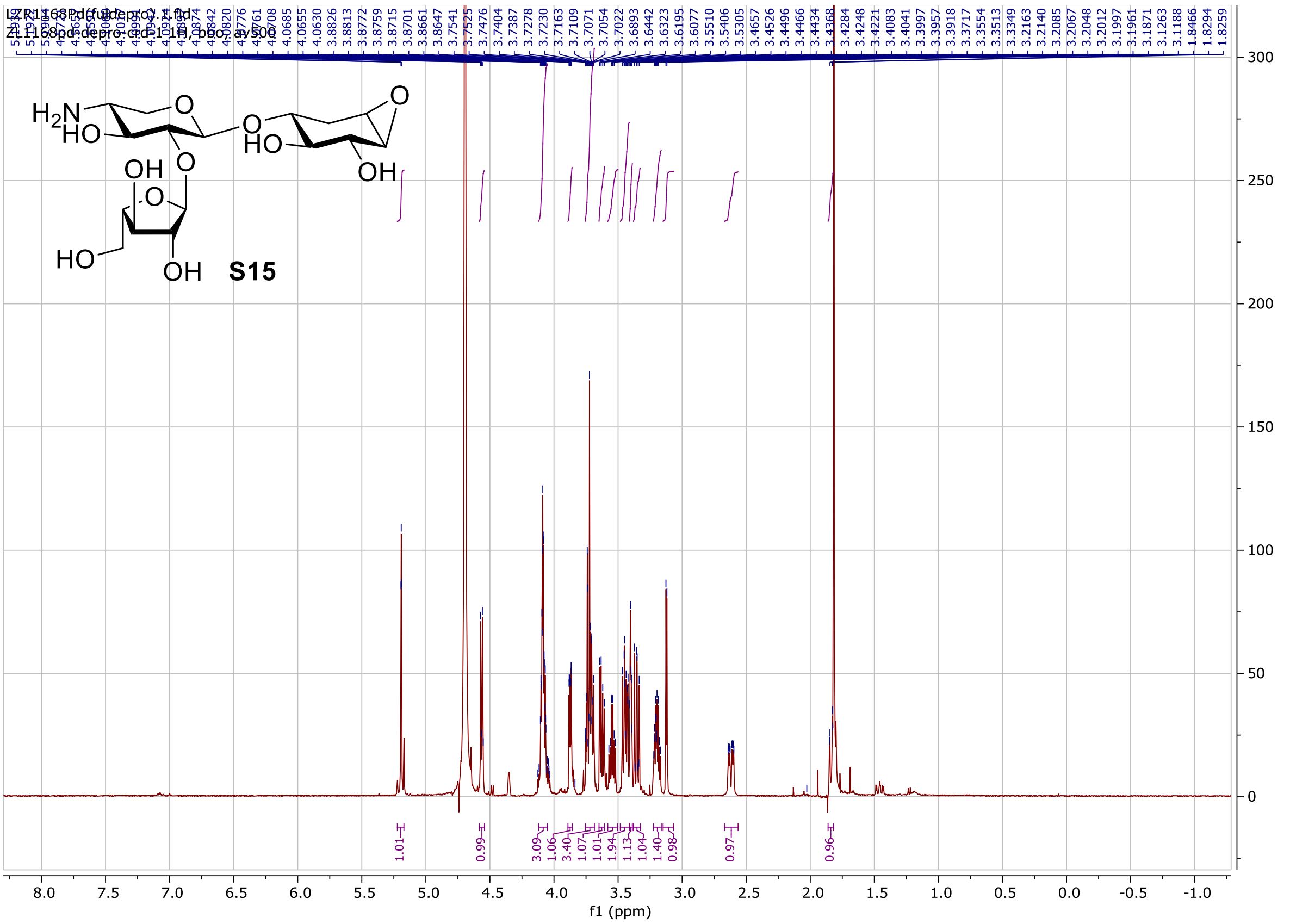

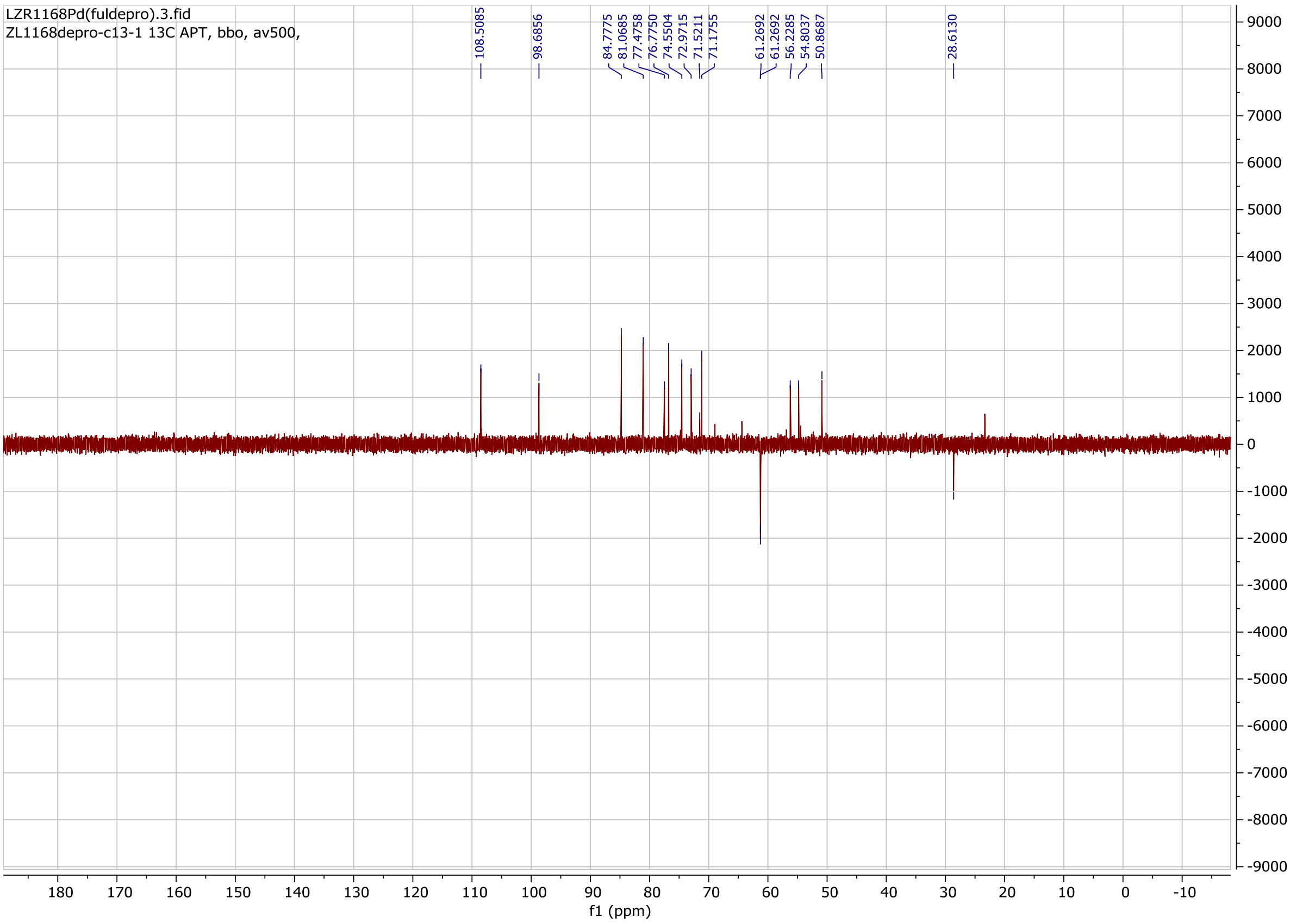

[1]H and ^{13}C of compound **2-Bio** in D_2O

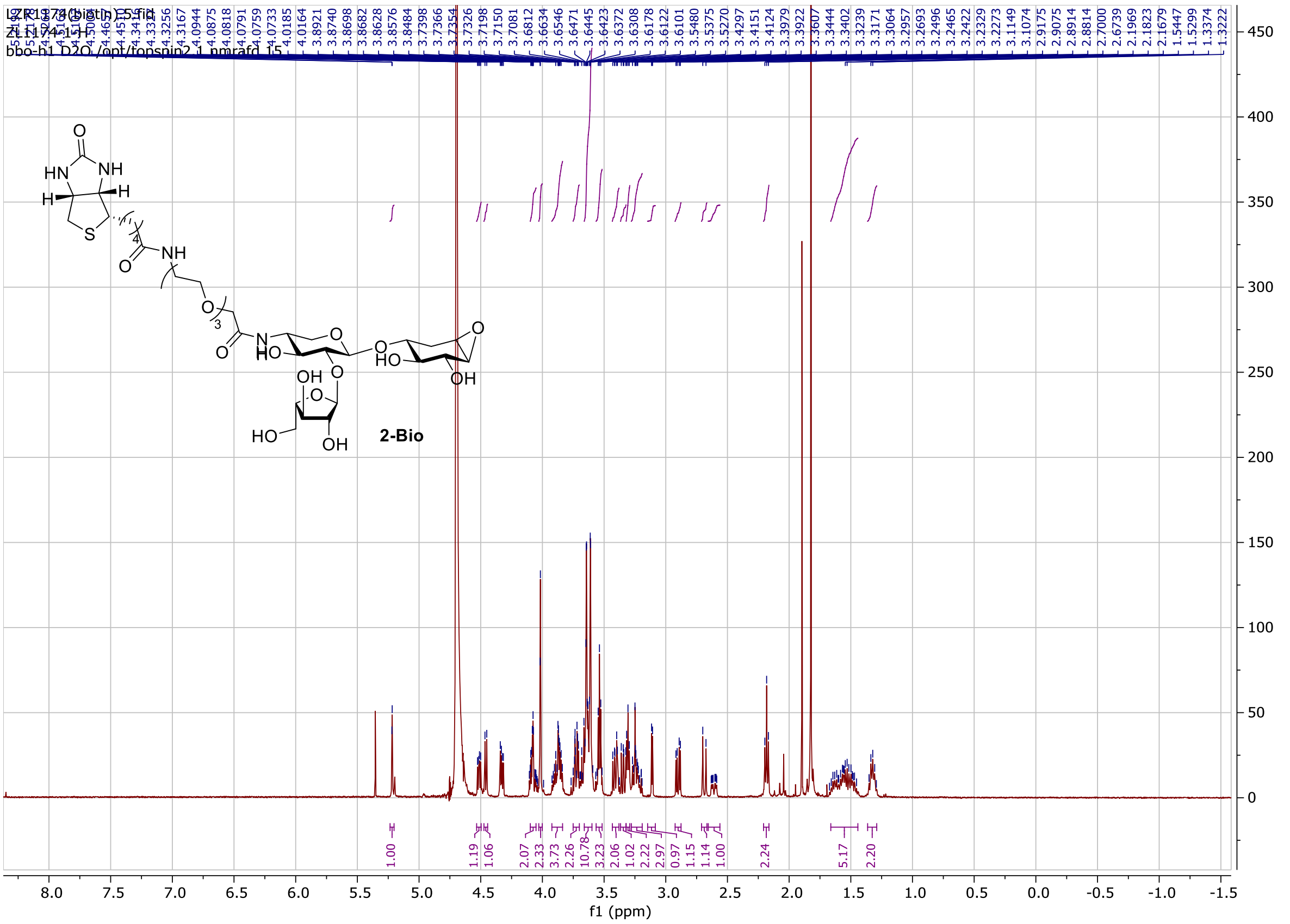

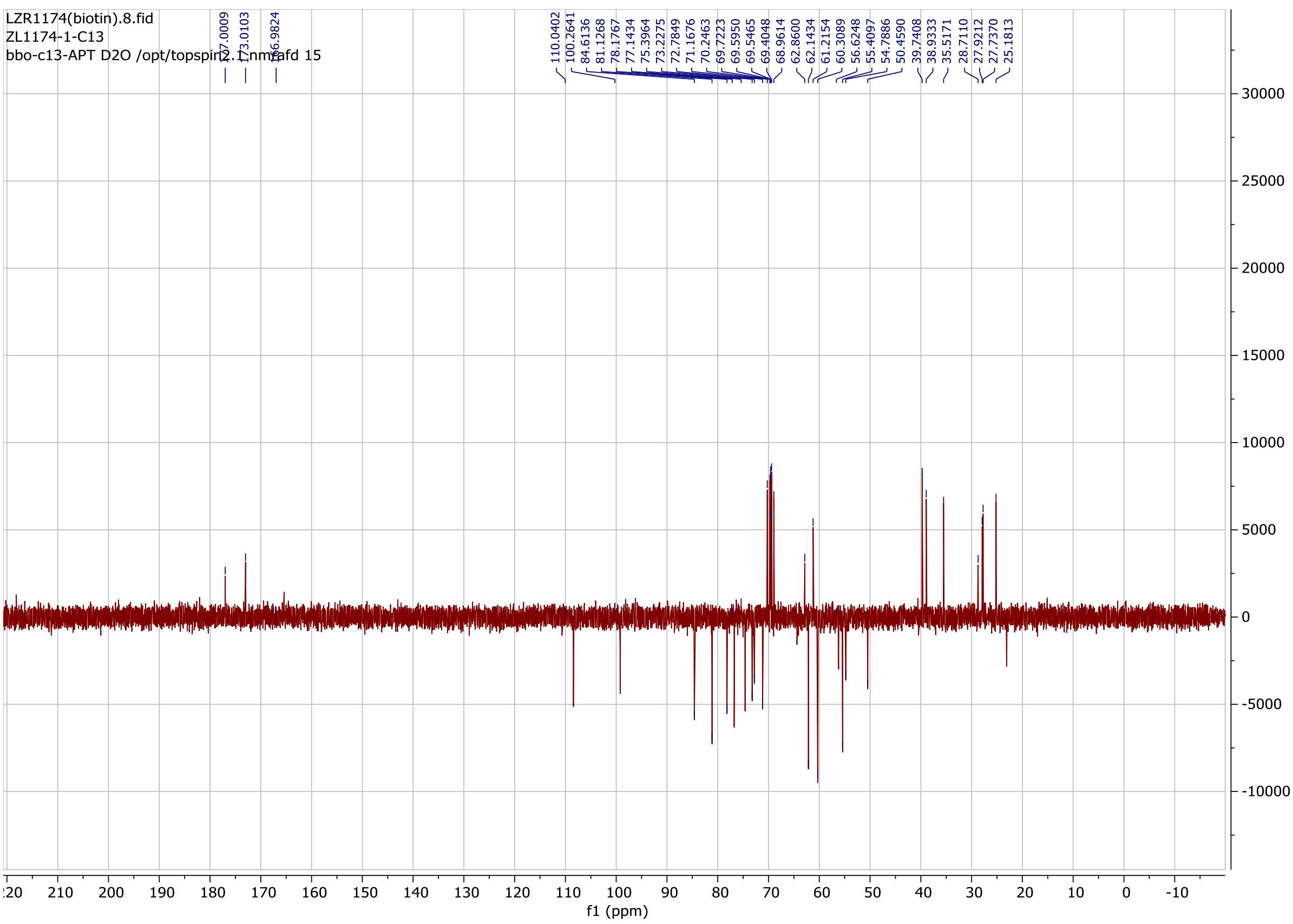

^{1}H of compound **2-Cy5** in D_2O and ^{13}C in MeOD

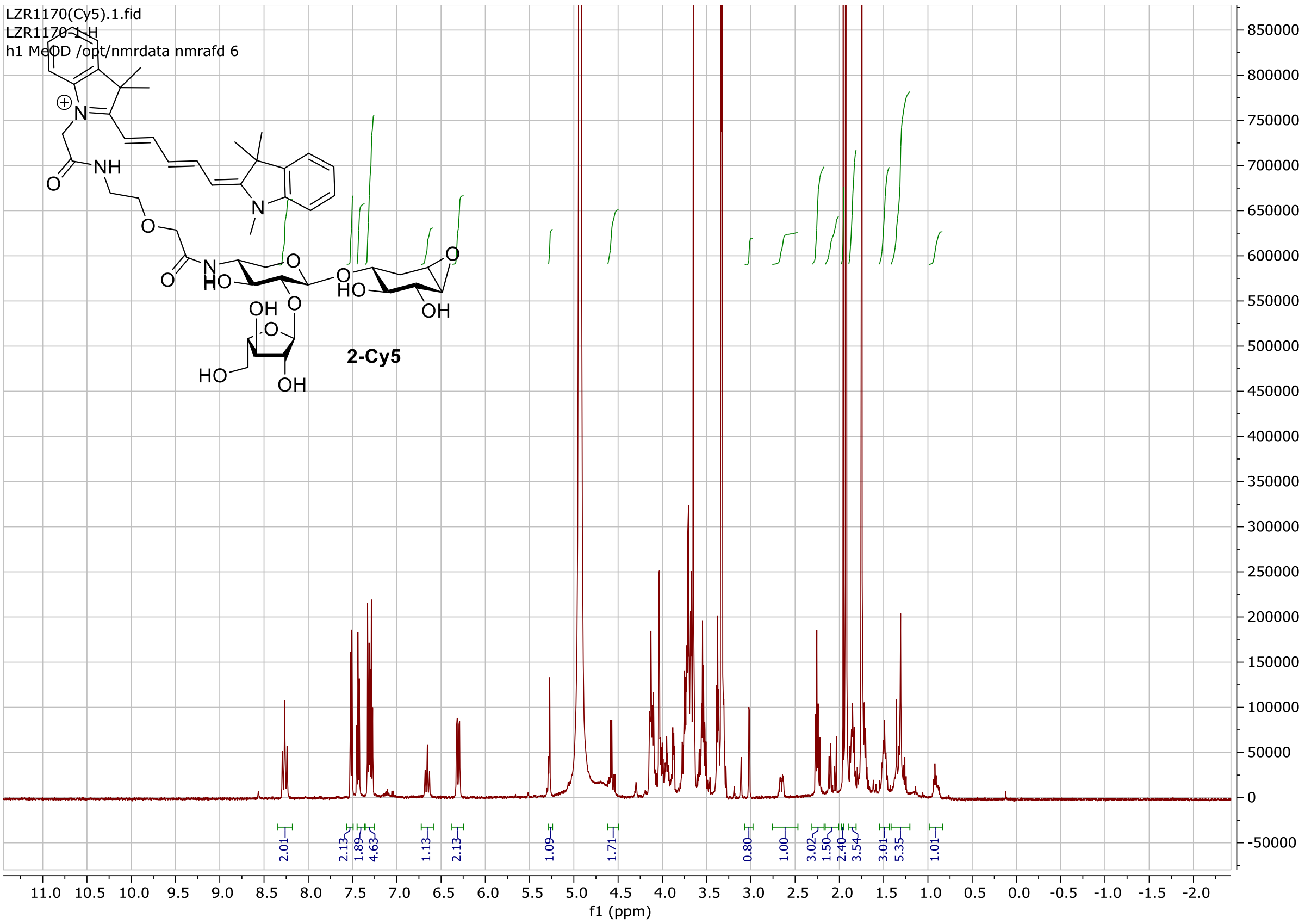

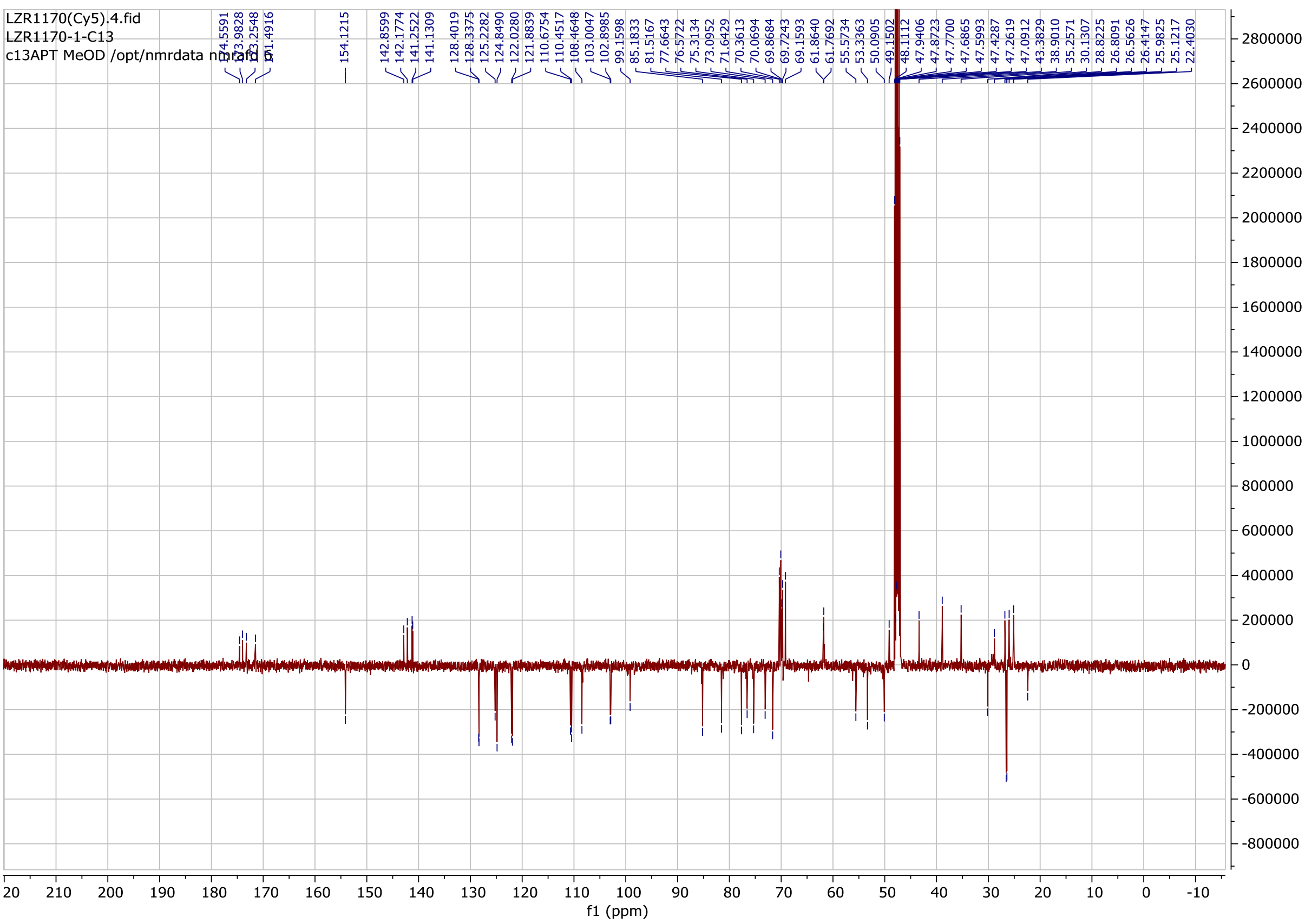

[1]H and ^{13}C of compound **S16** in $CDCl_3$

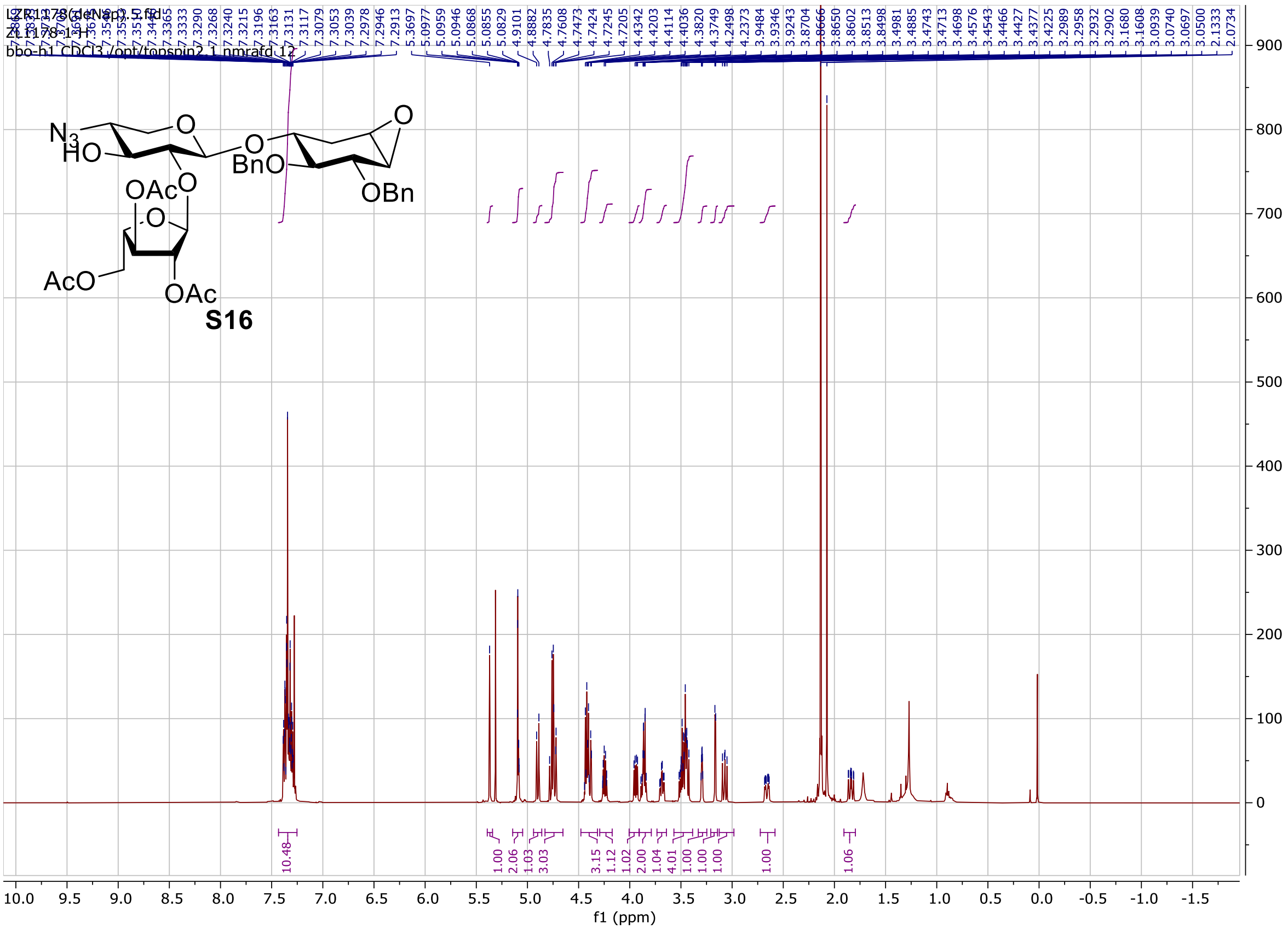

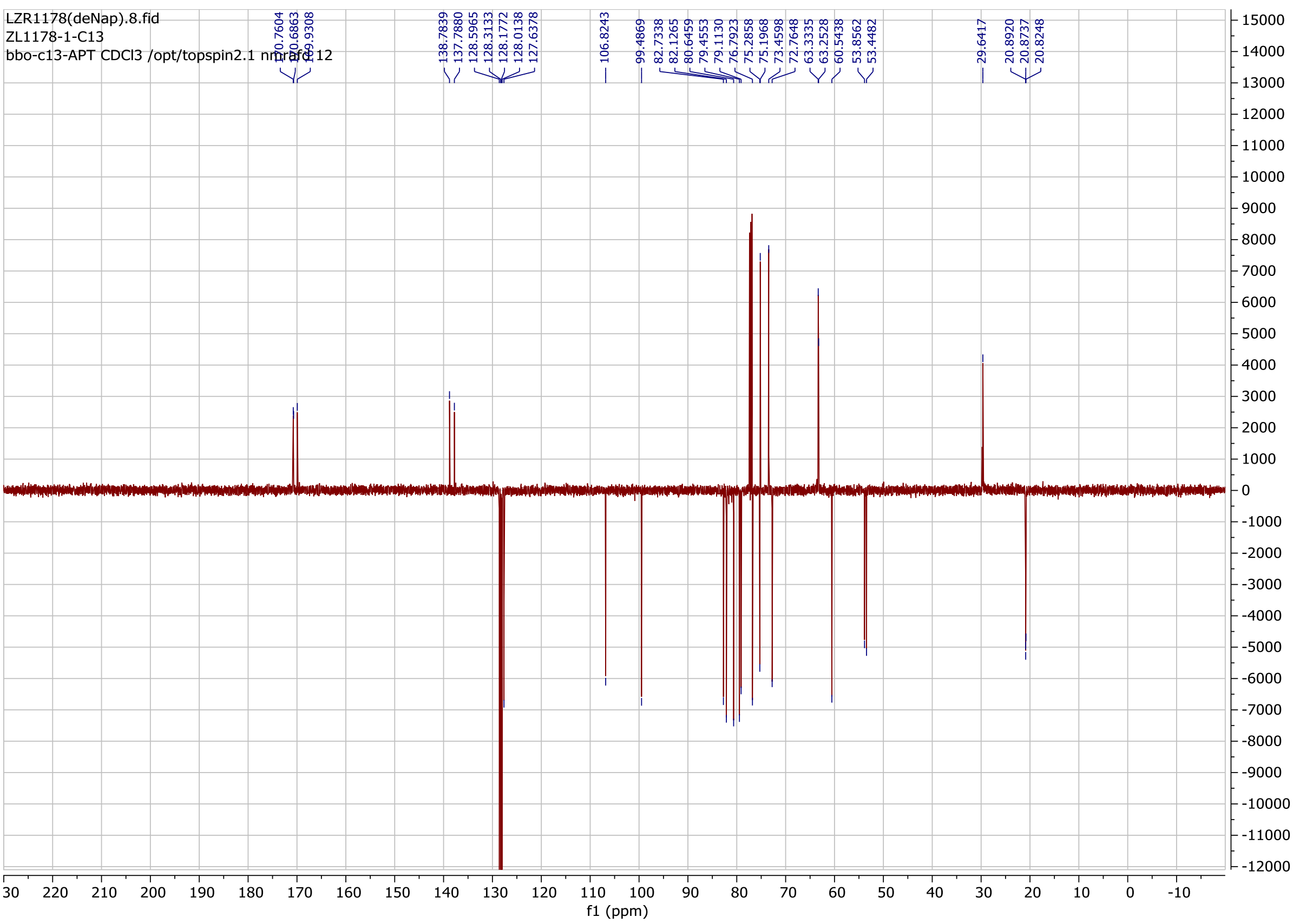

^{1}H and ^{13}C of compound **S17** in $CDCl_3$

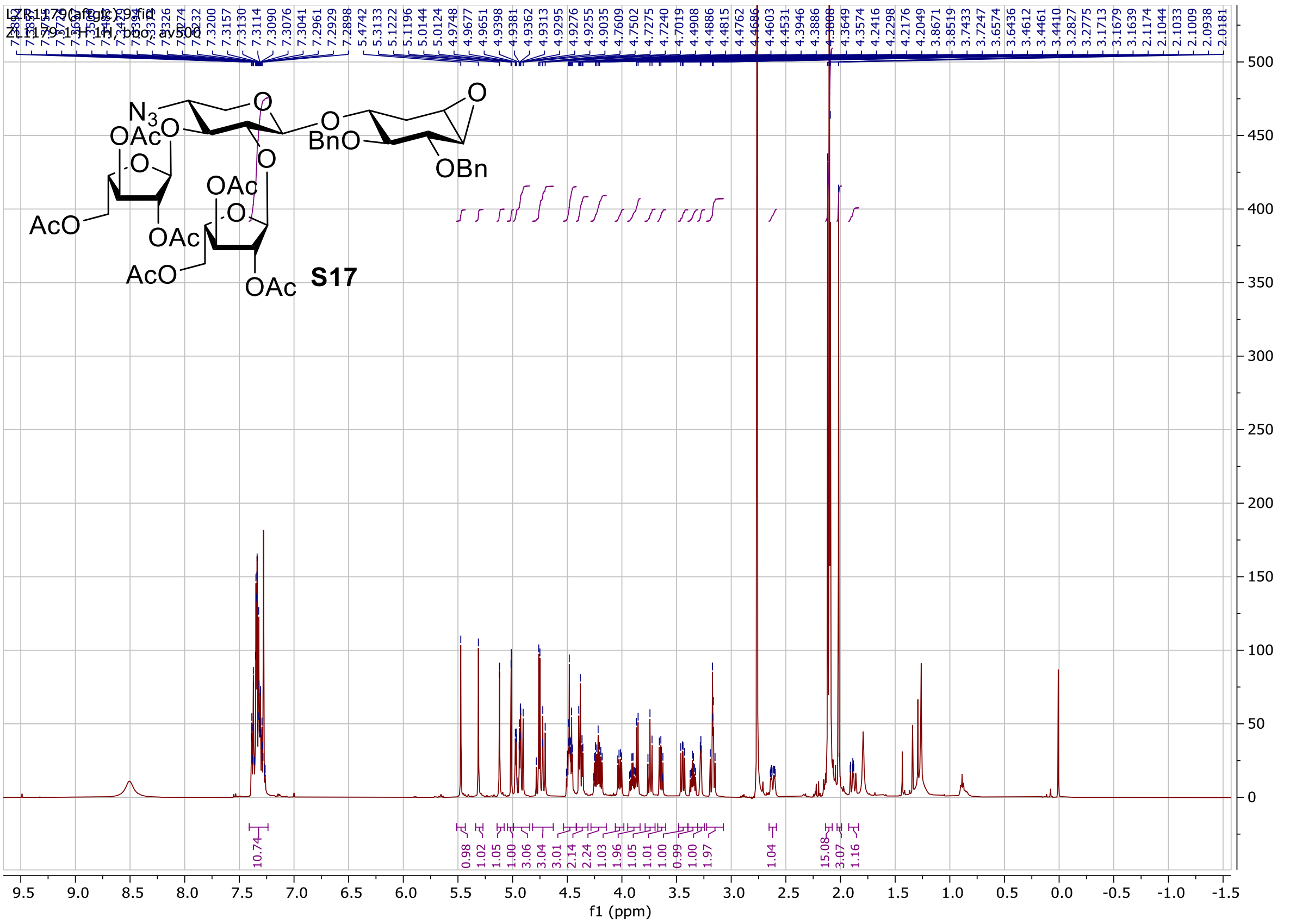

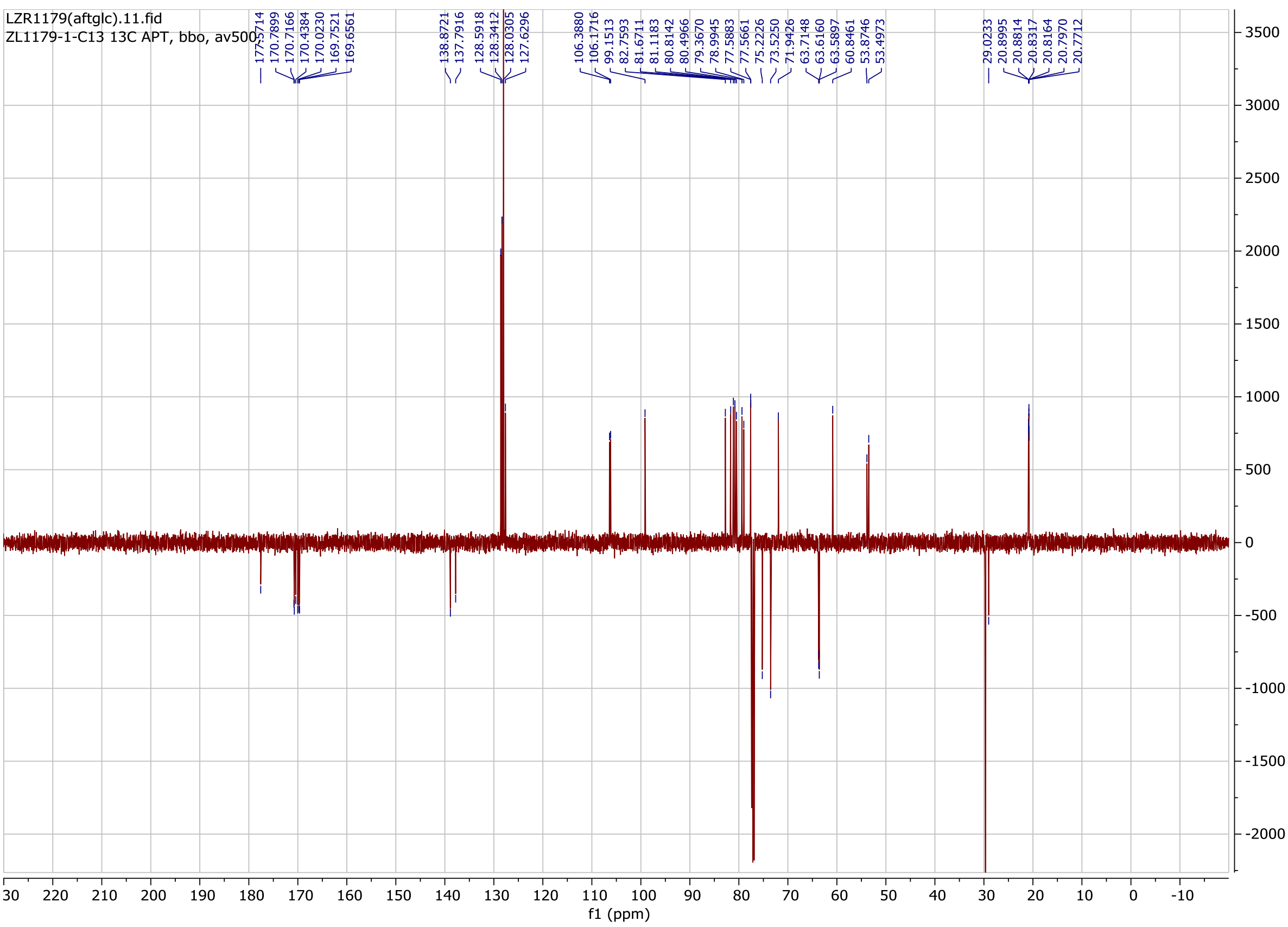

^{1}H and ^{13}C of compound **S18** in D_2O

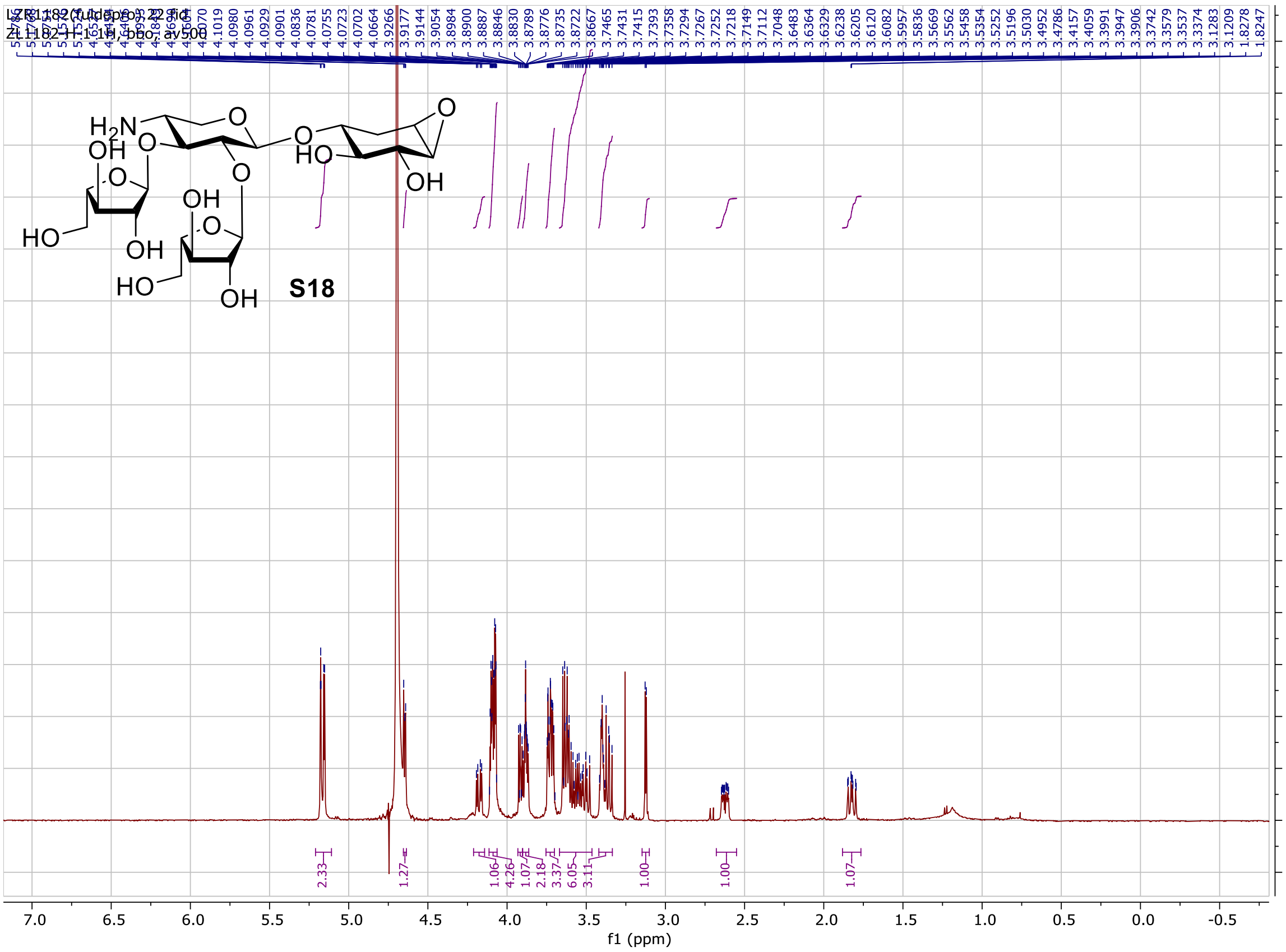

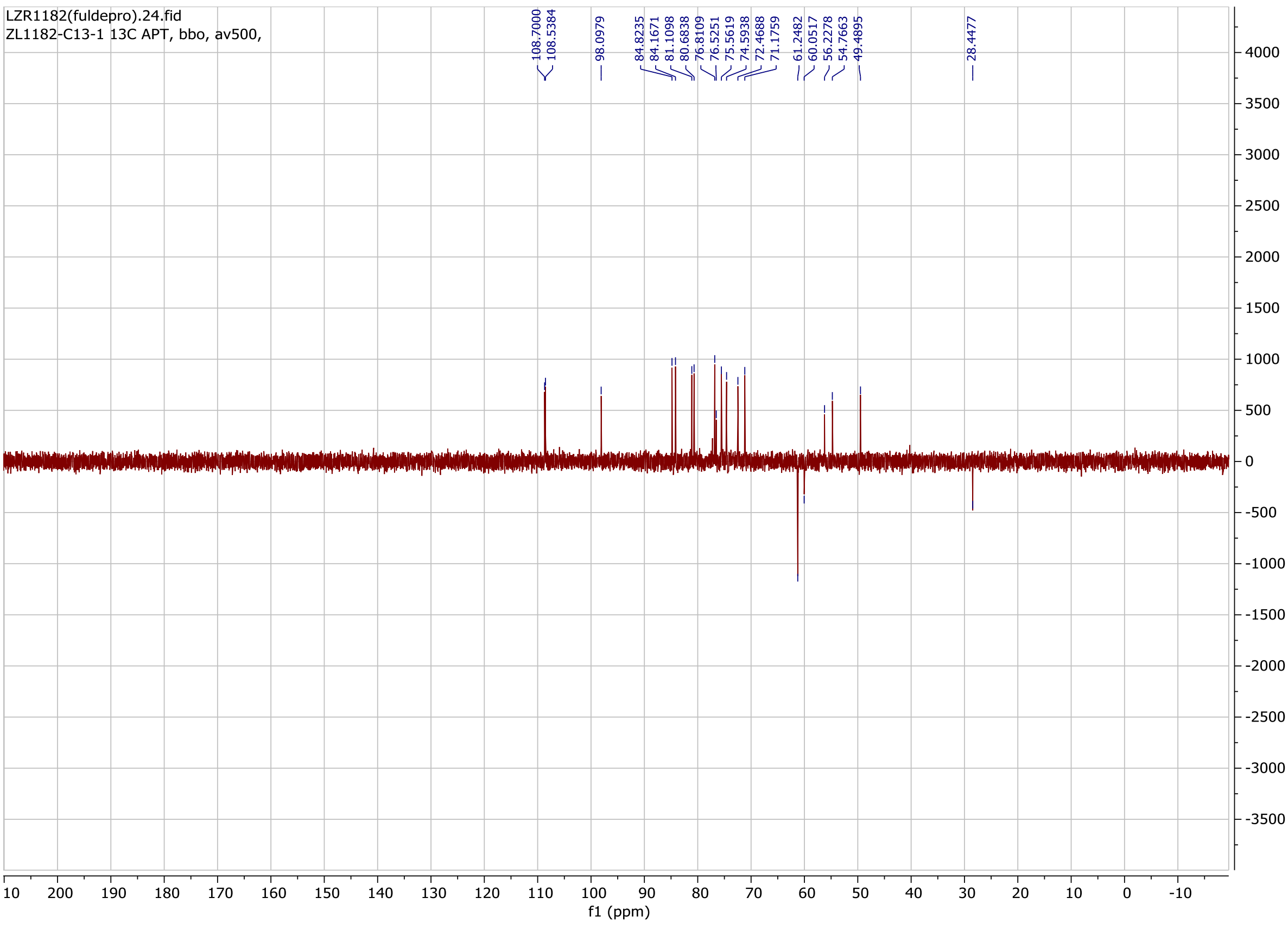

^{1}H and ^{13}C of compound **5-Bio** in D_2O

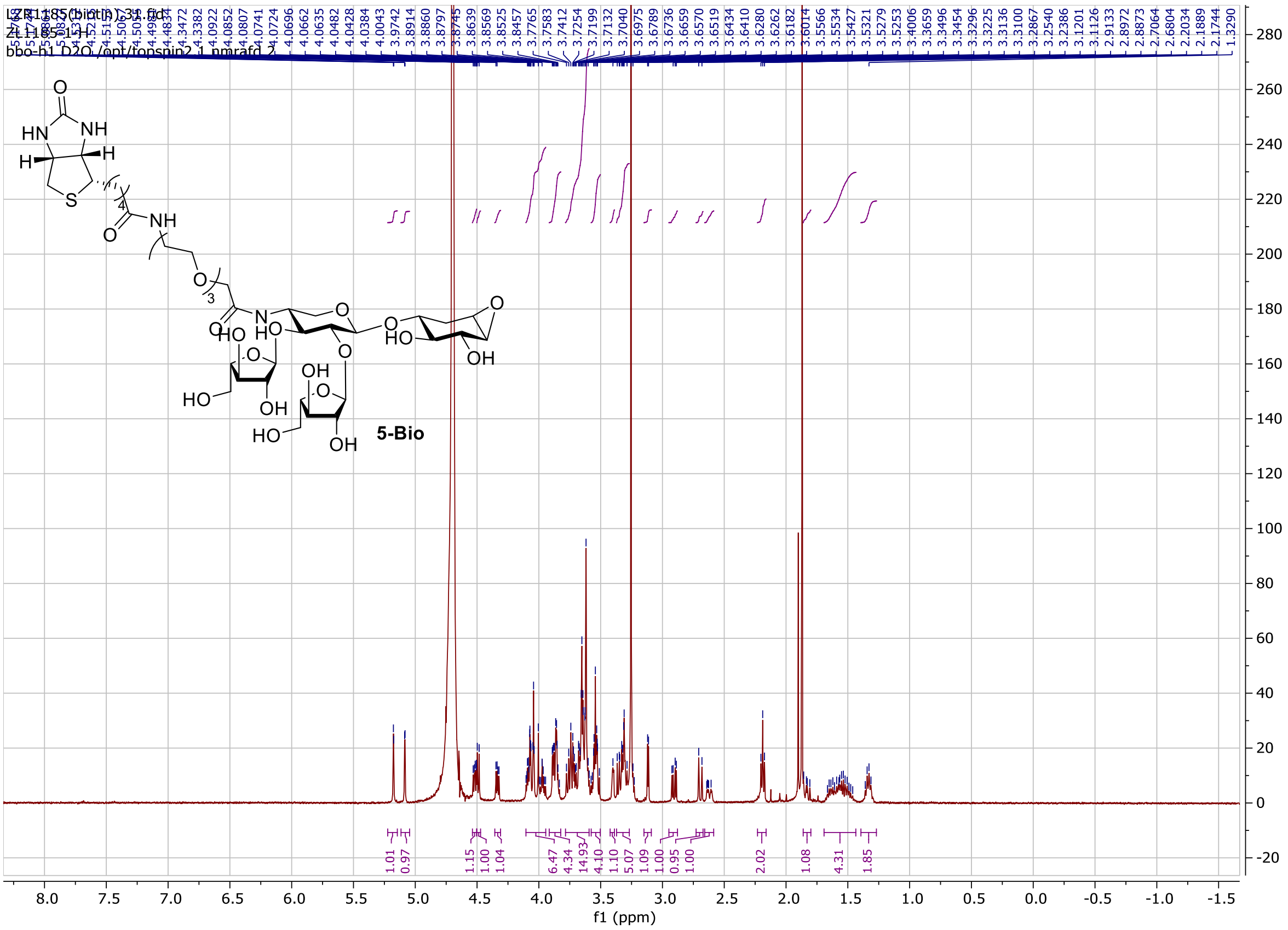

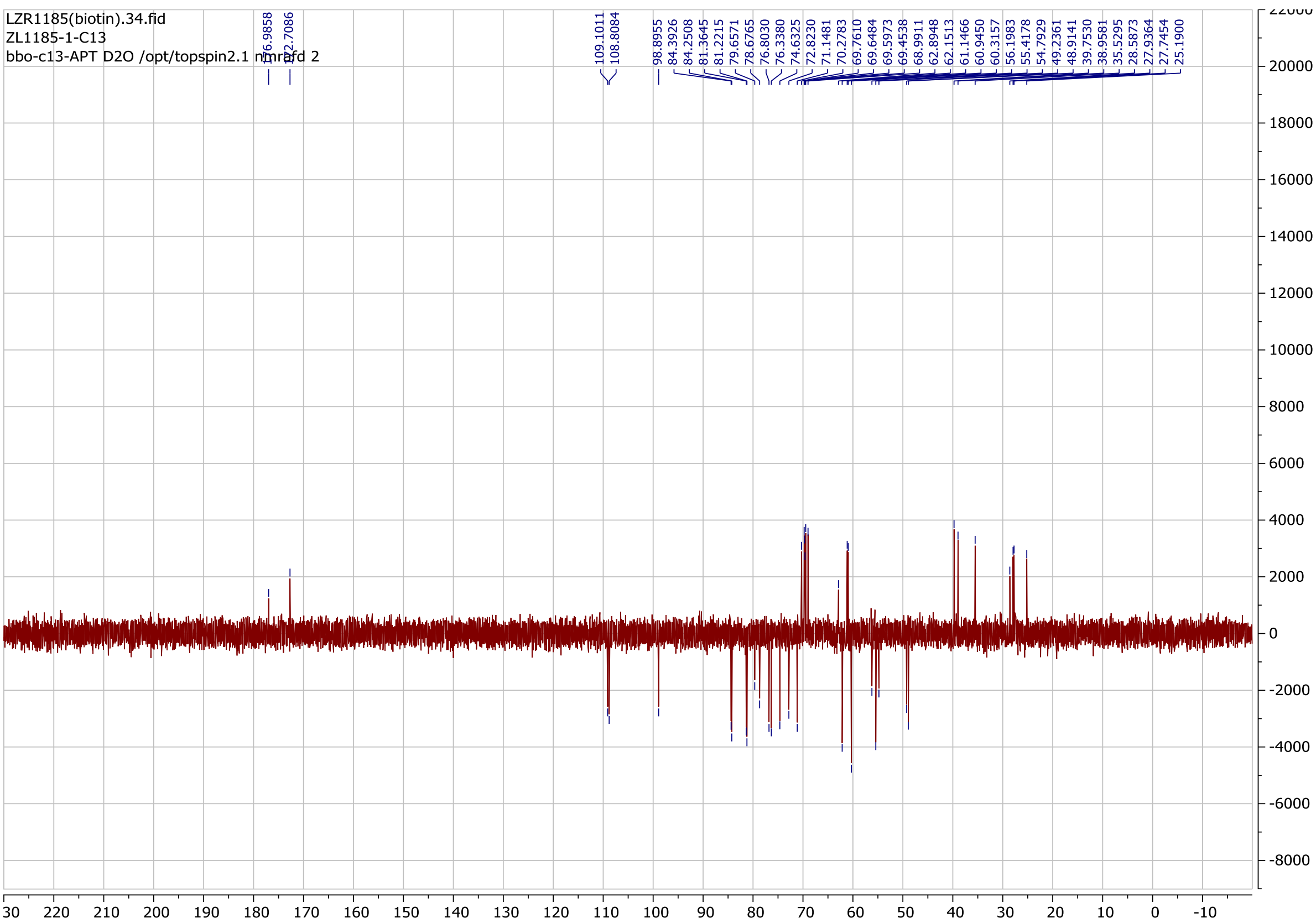

^{1}H and ^{13}C of compound **5-Cy5** in D_2O

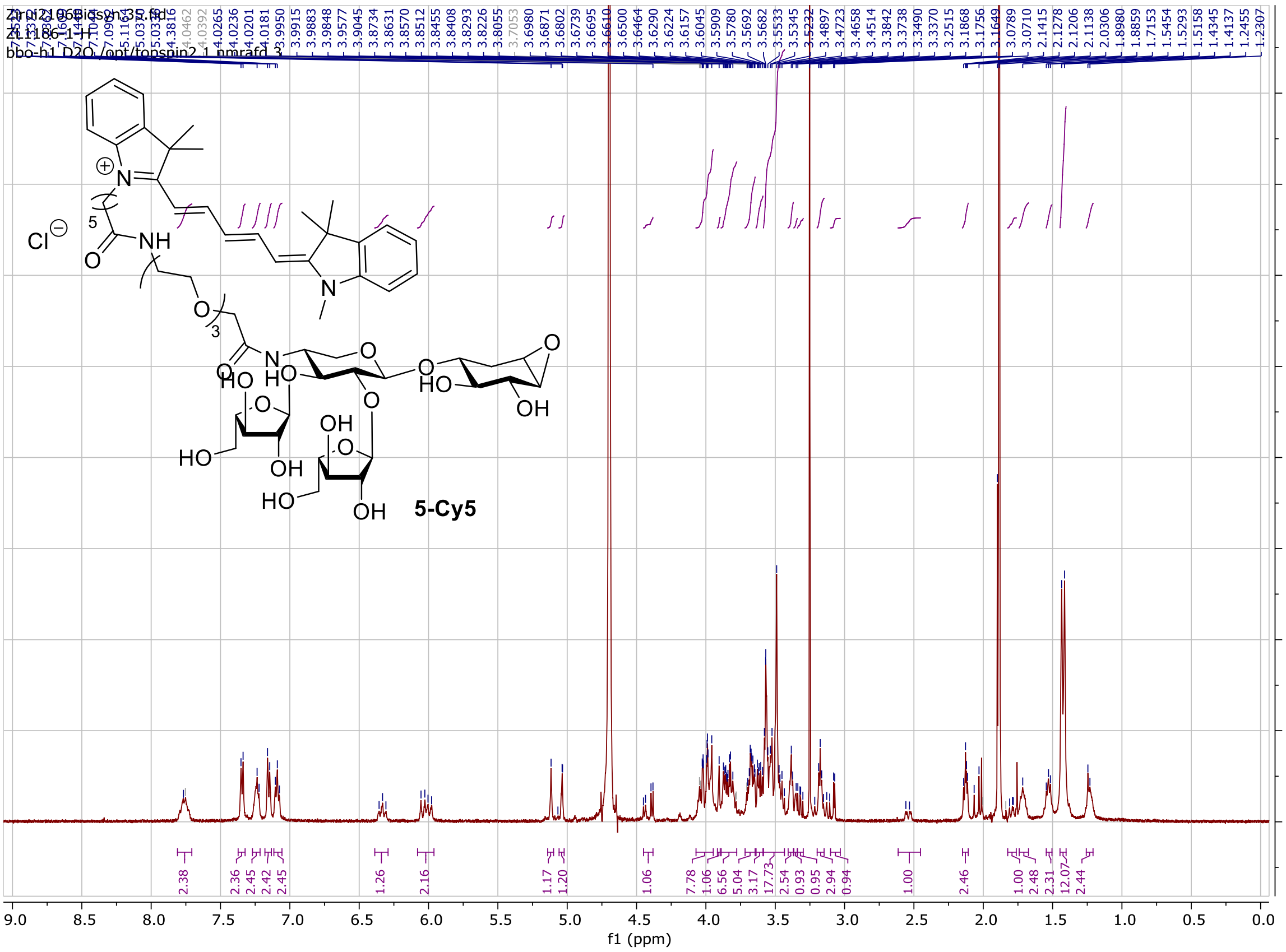

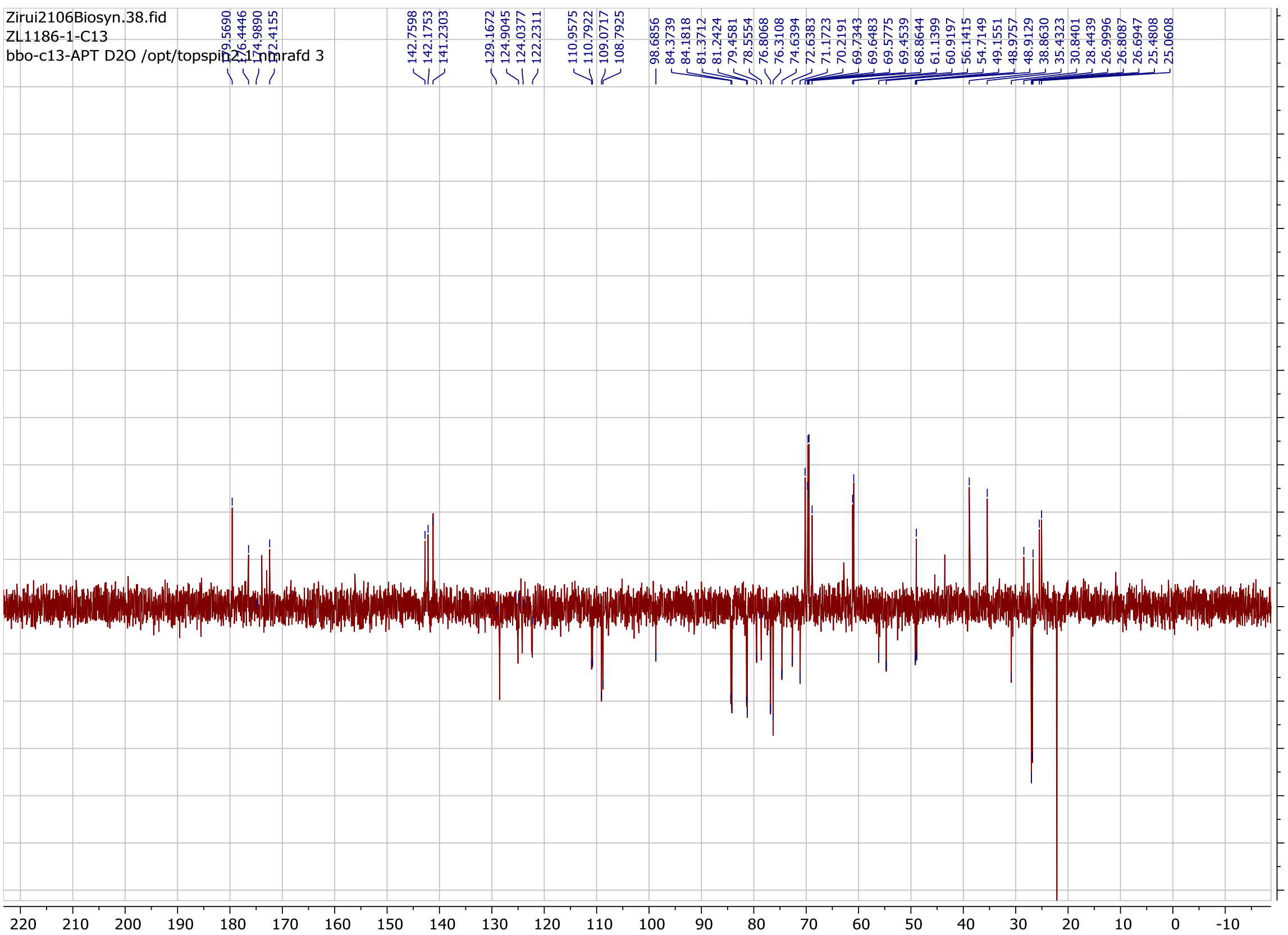

Supplementary Table 1. Primary sequences of the proteins used in this work

Protein	Source	CAZy	Mw (kDa)	ε (M^{-1} cm^{-1})	Sequence
*Ct*Xyn30A	*C. thermocellum*	GH30	46.8	91790	MGSSHHHHHHSSGLVPRGSHMASA<br>ETATINLSAEKQVIRGFGGMNHPV<br>WISDLTPQQRDTAFGNGEGQLGFT<br>ILRIHVDENRNNWSKEVATARRAI<br>ELGAIVFASPWNPPSNMVETFTRN<br>GVPNQKRLRYDKYGDYVQHLNDFV<br>AYMKSNGVDLYAISVQNEPDYAHE<br>WTWWTPQEMLRFMRDYAGQINCRV<br>MAPESFQYLKNMSDPILNDPQALA<br>NLDILGAHFYGTTVNNMPYPLFEQ<br>KGAGKELWMTEVYVPNSDSNSADR<br>WPEALEVAHNMHNALVEGNFQAYV<br>WWYIRRSYGPMKEDGTISKRGYMM<br>AHYSKFVRPGYVRVDATKNPTYNV<br>YLSAYKNKKDNSVVAVVINKSTEA<br>KTINISVPGTSIRKWERYVTTGSK<br>NLRKESDINASGTTFQVTLEPQSV<br>TTFV
*Ct*Xyl5A	*C. thermocellum*	GH5	54.1	135220	MASSPQRGRPRLNAARTTFVGDNG<br>QPLRGPYTSTEWTAAAPYDQIARV<br>KELGFNAVHLYAECFDPRYPAPGS<br>KAPGYAVNEIDKIVERTRELGLYL<br>VITIGNGANNGNHNAQWARDFWKF<br>YAPRYAKETHVLYEIHNEPVAWGP<br>PYSSSTANPPGAVDMEIDVYRIIR<br>TYAPETPVLLFSYAVFGGKGGAAE<br>ALKDIRAFNKAVFGNENAVWTNEA<br>VAFHGYAGWQETTIAVEELLKAGY<br>PCFMTEYAGGAWGSGMGGLDVELT<br>YELERLGVSWLTFQYIPPTGVSDD<br>VTKPEYFSALVENSGLSWTPDYGN<br>WPAARGVYGNGGLARETATWINNF<br>LTGTTRIEAEDFDWGGNGVSYYDT<br>DSVNVGGQYRPDEGVDIEKTSDTG<br>GGYNVGWISEGEWLEYTIRVRNPG<br>YYNLSLRVAGISGSRVQVSFGNQD<br>KTGVWELPATGGFQTWTTATRQVF<br>LGAGLQKLRINALSGGFNLNWIEL<br>SPILEHHHHHH
XynA	*Bacillus sp.* KW1	GH10	49.9	94880	MGSSHHHHHHSSGDVFRGSHMVKK<br>KEFMNHSSNGGSQEQEDQSWRKEA<br>NDRILQHRQRELVINVIDKEKKPL<br>AGIEVEIKQIRHEFAFGSAMNDQV<br>LFNQTYADFFVQHFNWAVFENEAK<br>WYANEPERGKITYEKADAMLNFAN<br>RHQIPVRGHALFWEVEDANPNWLK<br>SLPNHEVYEAMKRRLEHAGNHFKG<br>KFRHWDVNNEMMHGSFFKDRFGKQ<br>IWKWMYEETKKIDPQALLFVNDYN<br>VISYGEHHAYKAHINELRQLGAPV<br>EAIGVQGHFADRVDPVVVKERLDV<br>LAELGLPIWVTEYDSVHPDANRRA<br>DNLEALYRVAFSHPAVKGVLMWGF<br>WAGAHWRGEHAAIVNHDWSLNEAG<br>RRYEKLLQEWTTQRVEKTDANGQV<br>TCPAFHGTYEVRIGEESKMLQQQT<br>IELDSAKQTPLQLDIIVPVE
*Ta*Xyn10	*T. aurantiacus*	GH10	35.5	57410	QAAQSVDQLIKARGKVYFGVATDQ<br>NRLTTGKNAAIIQADFGQVTPENS<br>MKWDATEPSQGNFNFAGADYLVNW<br>AQQNGKLIRGHTLVWHSQLPSWVS<br>SITDKNTLTNVMKNHITTLMTRYK<br>GKIRAWDVVNEAFNEDGSLRQTVF<br>LNVIGEDYIPIAFQTARAADPNAK

					LYINDYNLDSASYPKTQAIVNRVK<br>QWRAAGVPIDGIGSQTHLSAGQGA<br>SVLQALPLLASAGTPEVAITELDV<br>AGASSTDYVNVVNACLNVQSCVGI<br>TVWGVADPDSWRASTTPLLFDGNF<br>NPKPAYNAIVQDLQQSRQKLISEE<br>DENSAVDHHHHHH
*So*Xyn10A	*S. olivaceoviridis E-86*	GH10	48.1	93850	MASESTLGAAAAQSGRYFGTAIAS<br>GKLGDSAYTTIASREFNMVTAENE<br>MKIDATEPQRGQFNFSAGDRVYNW<br>AVQNGKQVRGHTLAWHSQQPGWMQ<br>SLSGSTLRQAMIDHINGVMGHYKG<br>KIAQWDVVNEAFSDDGSGGRRDSN<br>LQRTGNDWIEVAFRTARAADPAAK<br>LCYNDYNIENWTWAKTQGVYNMVR<br>DFKQRGVPIDCVGFQSHFNSGSPY<br>NSNFRTTLQNFAALGVDVAITELD<br>IQGASSSTYAAVTNDCLAVSRCLG<br>ITVWGVRDTDSWRSGDTPLLFNGD<br>GSKKAAYTAVLNALNGGSSTPPPS<br>GGGQIKGVGSGRCLDVPNASTTDG<br>TQVQLYDCHSATNQQWTYTDAGEL<br>RVYGDKCLDAAGTGNGTKVQIYSC<br>WGGDNQKWRLNSDGSIVGVQSGLC<br>LDAVGGGTANGTLIQLYSCSNGSN<br>QRWTRTLEAHHHHHH

Mw, molecular weight; ε, molar extinction coefficient. The Mw and ε were predicted by ProtParam tool (https://web.expasy.org/protparam/).
The signal peptides were excluded from constructs.
Linker + CBM6 is highlighted in yellow for *Ct*Xyl5A.

Supplementary Table 2. XynA and *Ct*Xyn30A crystal data collection and statistics (numbers in brackets refer to data for highest resolution shells)

	Ct**Xyn30A**	**XynA**
Beamline	I03	I03
Wavelength (Å)	0.976	0.976
Space group	P1	$P4_122$
Unit cell (a, b, c)	51.07, 60.51, 83.88,	109.07, 109.07, 84.79
(α, β, γ)	98.40, 97.40,105.50	90, 90, 90
Total reflections	531746 (26941)	608504 (44430)
Unique reflections	147933 (7234)	34175 (2454)
Completeness (%)	98.00 (96.70)	100.00 (99.60)
Multiplicity	3.60 (3.70)	17.80 (18.10)
R_{meas} [5]	0.10 (0.92)	0.12 (4.17)
R_{pim} [6]	0.05 (0.47)	0.03 (0.97)
$\langle I / \sigma(I) \rangle$	7.40 (1.30)	14.20 (0.70)
Resolution range (Å)	48.44 - 1.50 (1.53-1.50)	57.05 - 2.02 (2.07-2.02)
$CC_{1/2}$ [7]	0.99 (0.63)	1.00 (0.31)
Wilson B-factor (Å^2)	12.17	46.80
**Refinement**		
Resolution (Å)	48.44 - 1.50	54.59 - 2.02
No. of reflections, working set	147844	34111
No. of reflections, test set	7296	1705
Final R_{cryst}	0.189	0.200
Final R_{free}	0.215	0.242
Coordinate error (Å) [8]	0.075	0.164
RMSD Bonds (Å)	0.012	0.0138
RMSD Angles (°)	1.950	2.495
Molprobity score	0.63	1.6
**Model properties**		
Number of chains (protein)	2	1
Overall number of atoms (non-H)	7326	3163
in macromolecules	6365	3080
in ligands	20 1T1 / 18 XYP / 26 GCV	10 NEL / 9 XYP / 9 AHR
in solvent	897	55
Average B-factor (Å^2)	16.0	64.2
protein; for chains	14.7	Catalytic domain 56.1 CBM 101.0
ligands, for chains	12.0 1T1 / 13.4 XYP / 14.4 GCV	60.4 NEL / 69.9 XYP / 108.6 AHR
solvent	25.3	53.7
**Ramachandran**		
Most favoured (%)	97.7	95.4
Allowed (%)	2.1	4.1
Outliers (%)	0.3	0.5
**PDB code**	**9RDG**	**9RHV**

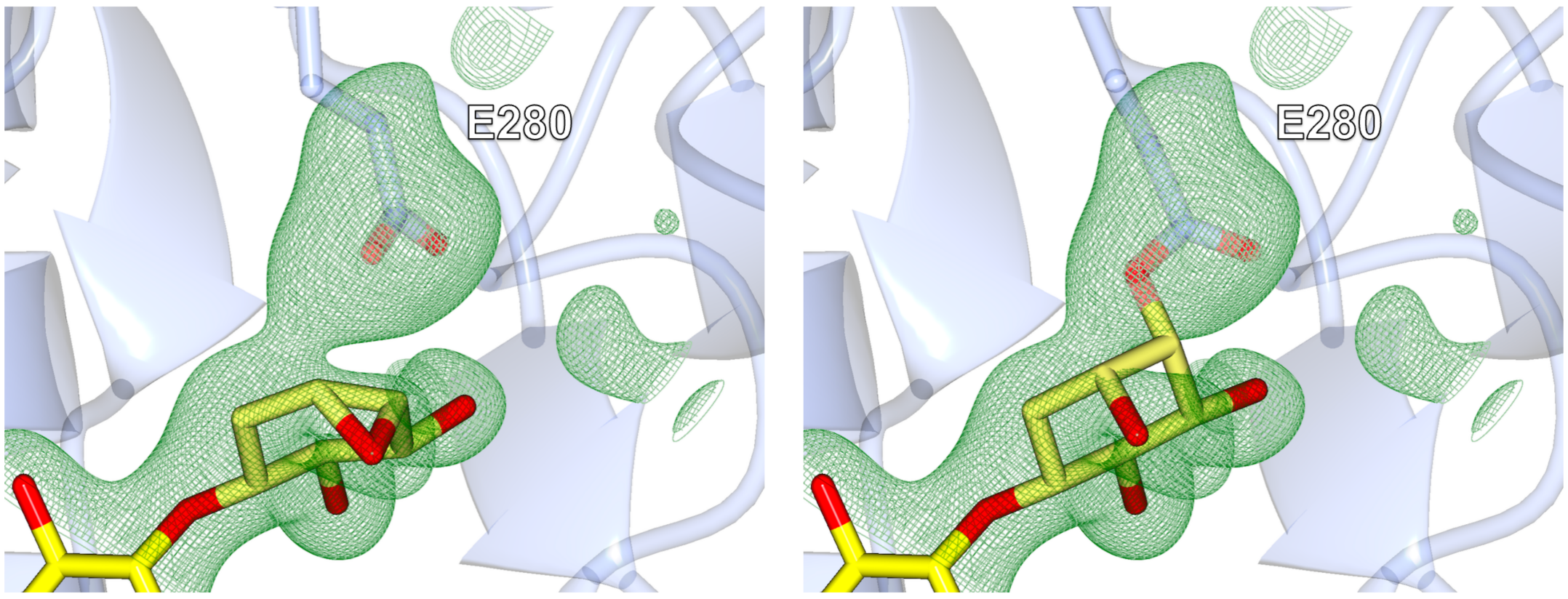

Supplementary Figure 4. Validation of dual-occupancy model for the XynA-3-Bio complex. A weighted difference omit map (mFo - DFc) was calculated after omission of the ligand and Glu280 side chain, followed by refinement. The resulting electron density is best explained by two ligand conformations corresponding to intact and reacted probe states. Both the ligand and Glu280 were modelled with alternative conformations and refined occupancies in the final structure.

Supplementary Table 3. Molecular interactions of **3-Bio** with XynA

Protein atom	Ligand atom/group	Distance (Å)	Type of interaction
Glu280 OE2	Cyclophellitol ring C1 (-1)	1.47	Covalent
Asn181 ND2	Cyclophellitol ring O2 (-1)	2.77	Hydrogen bond
His133 NE2	Cyclophellitol ring O3 (-1)	2.71	Hydrogen bond
Lys100 NZ	Cyclophellitol ring O3 (-1)	2.77	Hydrogen bond
Trp314 NE1	Cyclophellitol ring O4 (-1)	3.24	Hydrogen bond
Lys100 NZ	Cyclophellitol ring O4 (-1)	3.35	Hydrogen bond
Trp314 NE1	Xylobiosyl ring O2 (-2)	2.72	Hydrogen bond
Asn97 ND2	Xylobiosyl ring O3 (-2)	2.99	Hydrogen bond
Lys100 NZ	Xylobiosyl ring O5 (-2)	3.21	Hydrogen bond
Asn97 ND2	Ara*f* O3 (-2)	2.78	Hydrogen bond

Additionaly, hydrophobic contacts between the ligand and Trp137 (cyclophellitol), Trp322 (xylobiosyl), and Glu96 (Ara*f*) are noted.

The numbers between parenthesis correspond to the subsites.

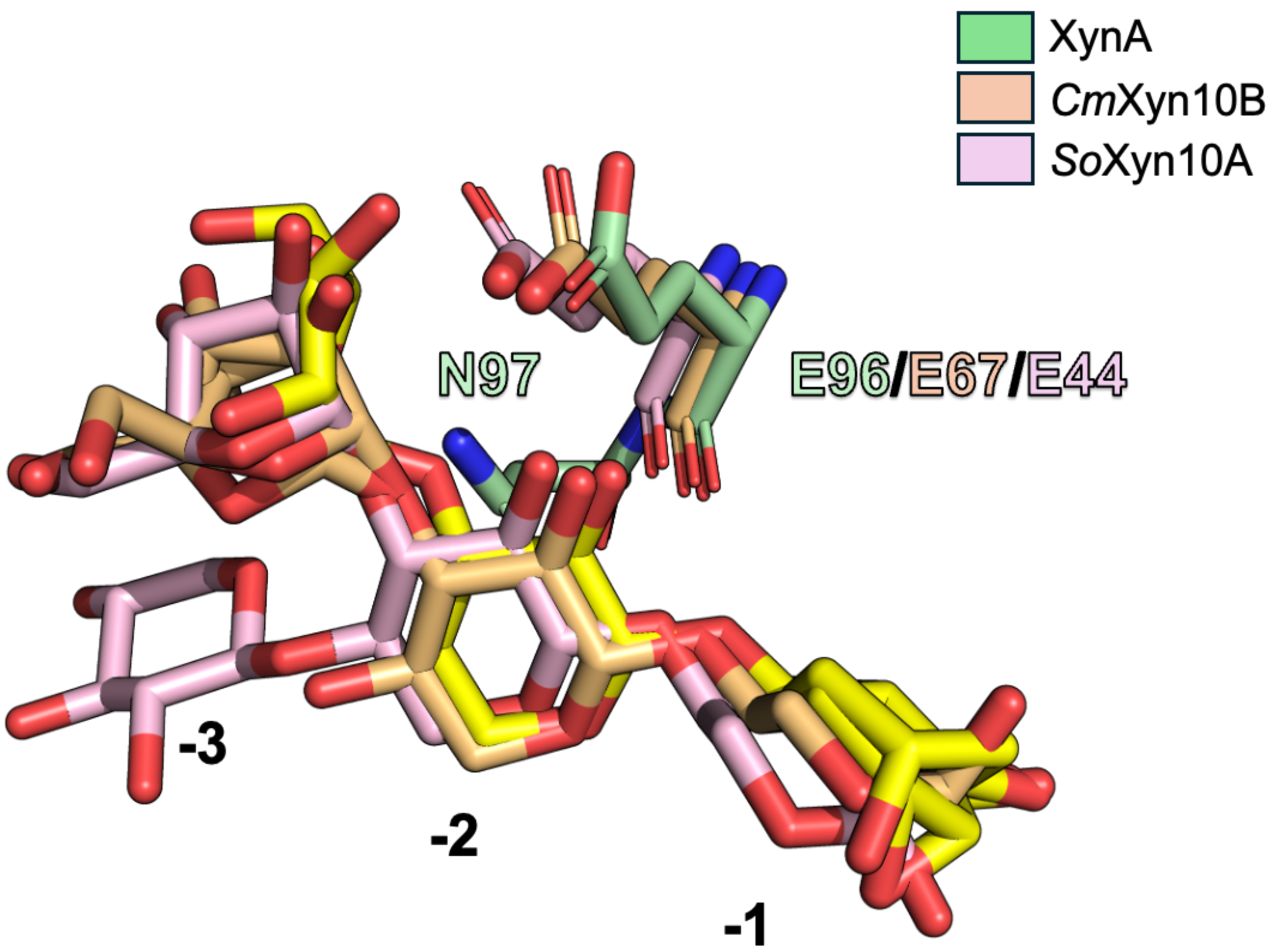

Supplementary Figure 5. Structural alignment of XynA and bacterial GH10s. Structural alignment of XynA (pale green/yellow ligand) with *Cm*Xyn10B-Ara*f*-X_2 (light orange, PDB:1UR1) and *So*Xyn10A-Ara*f*-X_3 (light pink, PDB: 1V6V) demonstrates the conserved mechanism of accommodation of the Ara*f* group by bacterial GH10s.

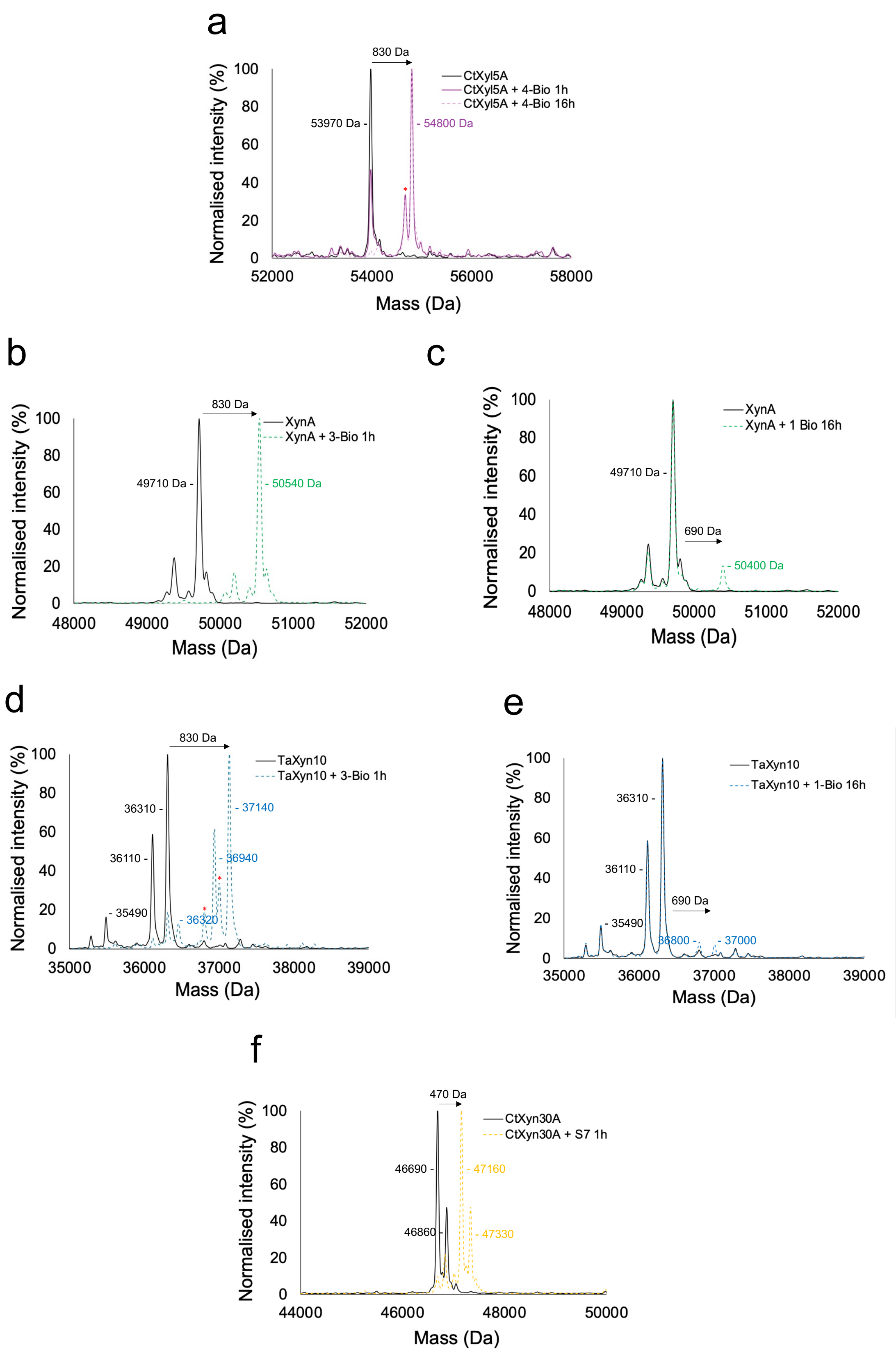

Supplementary Figure 6. Intact mass spectrometry of *Ct*Xyl5A, XynA, *Ta*Xyn10, and *Ct*Xyn30A. (a) *Ct*Xyn5A* shows partial conversion to *Ct*Xyn5A-**4-Bio** after 1 h and full conversion after 16 h incubation, with a mass shift consistent with formation of a single covalent adduct (predicted: +825 Da; observed: +830 Da). **(b-c)** XynA** shows full conversion to XynA-**3-Bio**, with formation of a single covalent adduct (predicted: +825 Da; observed:

+830 Da). In contrast, less than 10% of XynA was converted to XynA-**1-Bio** even after 16 h of incubation. **(d-e)** A similar profile was observed for *Ta*Xyn10***. **(f)** Full conversion of *Ct*Xyn30A**** to *Ct*Xyn30A-**S7** was observed, with a mass shift consistent with formation of a single covalent adduct (predicted: +469 Da; observed: +470 Da).

*The difference between the predicted (54,109 Da) and observed (53,970 Da) masses in *Ct*Xyl5A is due to loss of an "H" from the C-terminal His-tag;
** The difference between the predicted (49,896 Da) and observed (49,710 Da) masses in XynA is due to loss of "MG" from the N-terminus;
*** The 35,490 Da peak corresponds to unmodified *Ta*Xyn10 (predicted: 35,525 Da). The 36,110 Da and 36,310 Da peaks represent glycosylated proteoforms, consistent with mannosylation – addition of 4-5 sugar units;
**** The difference between the predicted (46,818 Da) and the observed masses (46,860 Da and 46,690 Da) are likely due to the presence of populations of N-terminal acetylated *Ct*Xyn30A and native protein lacking the N-terminal methionine, respectively.

Red asterisks in panels "a" and "d" indicate that subpopulations of *Ct*Xyl5A and *Ta*Xyn10 incubated with **3-Bio** are also modified with **1-Bio.** *Ct*Xyl5A and *Ta*Xyn10 are extensively characterised in the literature with respect to their substrate specificities, and none have been reported to exhibit arabinofuranosidase activity[9,10], which precludes a catalytic explanation for the observed loss of arabinose. In addition, LC–MS analysis with both probes revealed no detectable degradation (data not shown). We therefore attribute the loss of arabinose to the conditions employed in the intact mass spectrometry workflow, in which the acidification promoted by TFA under mild temperature can promote the cleavage of arabinose decorations in xylan-derived compounds[11,12].

Supplementary Table 4. Interactions of MeGlcA with *Ct*Xyn30A

Protein atom	Ligand atom/group	Distance (Å)	Type of interaction
Arg291 NH1	MeGlcA COOH	2.9	Salt bridge
Arg291NE	MeGlcA COOH	3.0	Salt bridge
Tyr293 OH	MeGlcA O6	2.6	Hydrogen bond
Ser254 OG	MeGlcA O3	2.7	Hydrogen bond
Tyr250 OH	MeGlcA O2	2.7	Hydrogen bond
Trp287 NE1	MeGlcA O5	3.4	Hydrogen bond
Tyr293 OH	MeGlcA O5	3.0	Hydrogen bond

Supplementary Table 5. *C. thermocellum* enzymes involved in xylan breakdown

Uniprot id.	Enzyme	Activity	CAZy	Mw (kDa)	Domains
A3DIL1	XynD	Endo-β-1,4-xylanase/ Acetyl xylan esterase	GH10	71.6	CBM22, GH10, Dockerin 1
A3DJP0	XynA	Endo-β-1,4-xylanase	GH11	74.4	CBM22, CBM6, CE4, Dockerin 1
A3DJS9	*Ct*Xyn30A	Endo-β-1,4-xylanase	GH30	70.3	GH30, CBM6, Dockerin1
A3DHG6	*Ct*Xyl5A	Endo-β-1,4-xylanase	GH5	103.1	GH5, CBM6, CBM13, CBM62, Dockerin 1
A3DGI0	XynC	Endo-β-1,4-xylanase	GH10	69.5	GH30, CBM6, Dockerin 1
A3DHG8	XynE	Endo-β-1,4-xylanase	GH141	105	GH141, CBM6, Dockerin 1
A3DDW7	XynY	Endo-β-1,4-xylanase/ Feruloyl esterase	GH10	119.7	CBM22, GH10, CBM22, CE1, Dockerin 1
P10478	XynZ	Endo-β-1,4-xylanase Feruloyl esterase	GH10	92.3	CE1, CBM6, GH10, Dockerin1
A3DEX4	*Ct*43Ara*f*	α-L-arabinofuranosidase	GH43	74.5	GH43, CBM6, CBM6, Dockerin 1
A3DHG9	AxB8	α-L-arabinofuranosidase	GH43	58.7	GH43, CBM6, Docherin1

Adapted from[13]

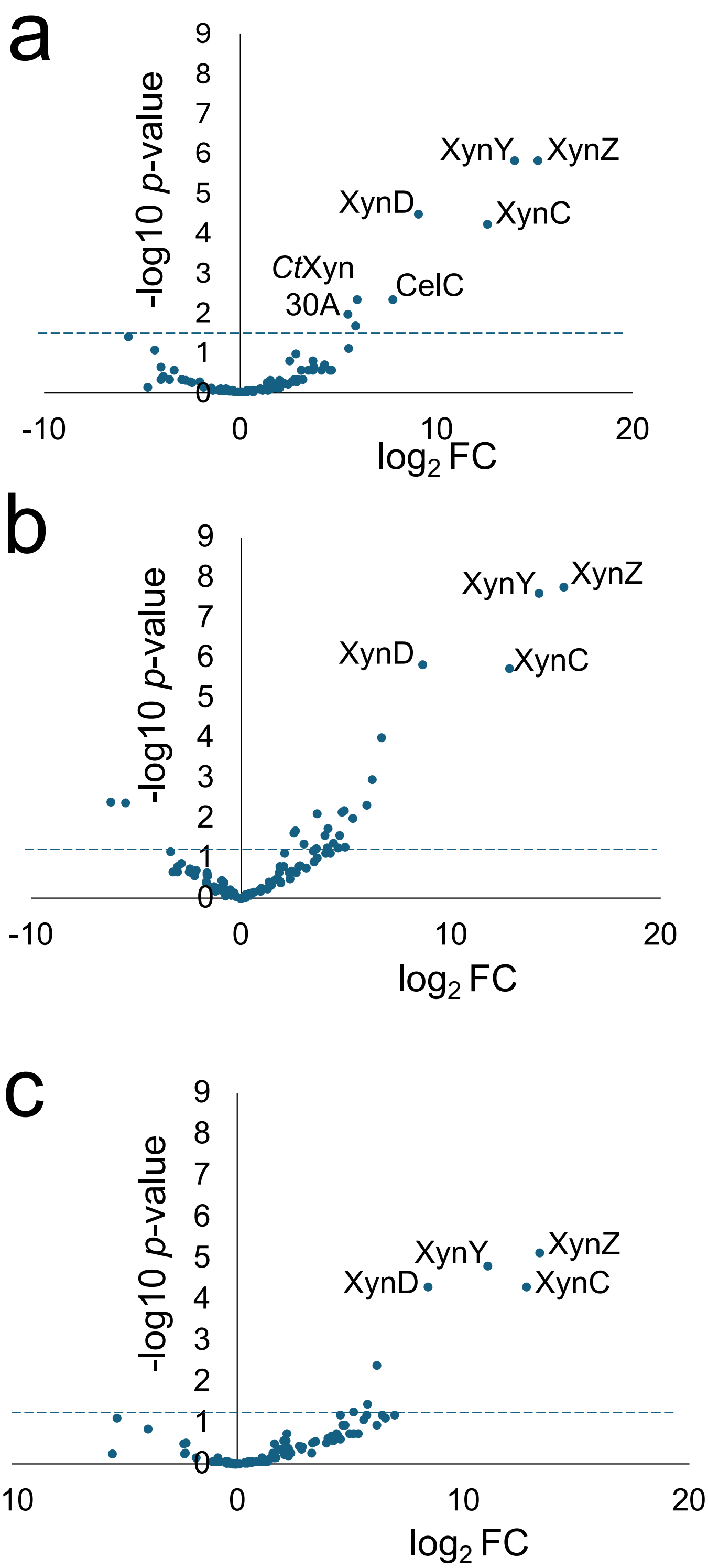

Supplementary Figure 7. ABPP of *C. thermocellum* proteome. Pull-down of the proteins labelled in Fig. 5a with **(a) 2-Bio**, **(b) 3-Bio**, and **(c) 5-Bio**. The profile of the enriched peptides is similar to that observed in **1-Bio** condition, as illustrated in Fig. 5b (main text). The threshold for protein enrichment significance (-$\log_2$ fold change, *x*-axis) was set at a Benjamini-Hochberg *p*-value of ≤ 0.05 (equivalent to a -$\log_{10}$value of 1.3, *y*-axis). The secretome of *C. thermocellum* grown on cellobiose for 72 h was incubated with **2-Bio**, **3-Bio**, and **5-Bio** at 30°C for 2 h.

Supplementary Table 6. *B. subtilis* subsp. *subtilis* str. 168 enzymes involved in xylan breakdown

Uniprot id.	Enzyme	Activity	CAZy	Mw (kDa)
Q59254	XynA	Endo- β-1,4- xylanase	GH11	23.3
P94489	XynB	Xylan β-1,4-xylosidase	GH43	61.4
H1ACZ7	XynC	Glucuronoxylan-specific endo-β-1,4-xylanase	GH30	47.2
Q45071	XynD	Arabinoxylan arabinofuranohydrolase	GH43	54.5

Source: https://rth.dk/resources/bsgatlas/

Supplementary Table 7. Annotation of CAZy families involved in xylan conversion in the genomes of C*. globosum, A. niger, and T. reesei*

**CAZy family**	***C. globosum* CBS 148.51**	***A. niger* CBS 513.88**	***T. reesei* RUT C-30**
GH3	13	17	13
GH5	10	10	12
GH10	7	1	1
GH11	9	4	4
GH30	2	1	6
GH43	17	10	3
**Total**	**58**	**43**	**39**

Source: https://mycocosm.jgi.doe.gov/mycocosm/home

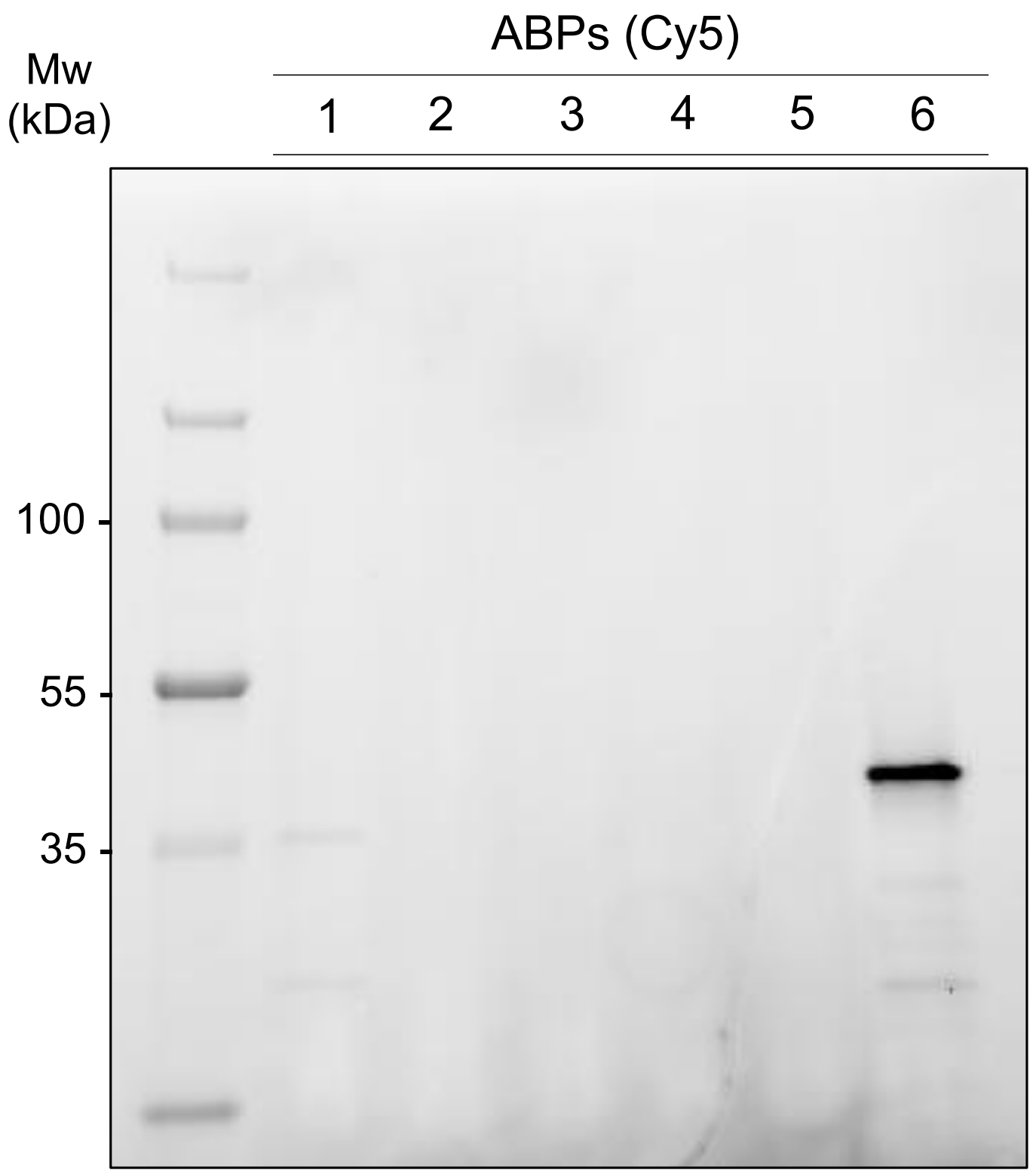

Supplementary Figure 8. ABPP of *B. subtilis* secretome. The secretome of *B. subtilis* grown on xylan for 48 h was incubated with **1-6-Cy5** at 30ºC for 1 h. Among all probes, ABP **6-Cy5** (MeGlcA-decorated) showed strong and selective labelling of a protein band in the 35–55 kDa range, suggesting that this protein corresponds to the *B. subtilis* GXX XynC, 47 kDa. Mw, molecular weight.

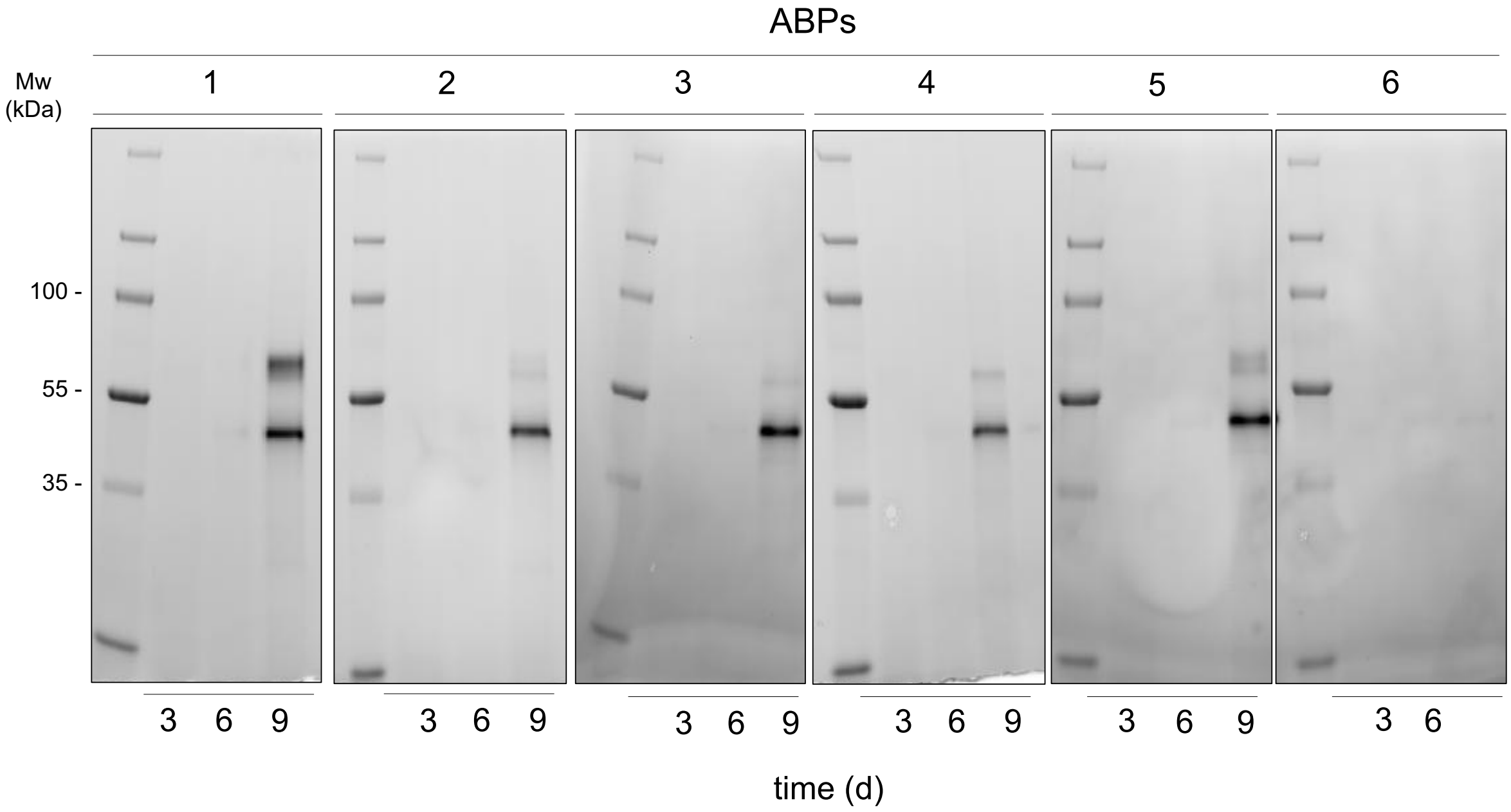

Supplementary Figure 9. ABPP of *C. globosum* secretome. The secretome of *C. globosum* cultivated on xylan for nine days was incubated with **1-6-Cy5** at 30°C for 1 h. The intense labelling of the band above 55 kDa by the non-decorated probe (**1-Cy5**) suggests that these proteins are xylanases that do not accept Ara*f* decorations. In contrast, the stronger labelling observed in the 35-55 kDa range with the Ara*f*-decorated (**2-5-Cy5**) probes suggest that these ABPs labels fungal AXXs. Mw, molecular weight.

Supplementary Table 8. GH10 and GH30 xylanases annotated in *C. globosum* genome

Uniprot id.	CAZy family	SP	Mw (kDa)	GS	Domain
Q2HIC4	GH10	1-17	44.3	1	CBM1
Q2HHK0	GH10	1-20	40.5	1	CBM1
Q2H7X4	GH10	1-18	35.1	1	x
Q2GM35	GH10	1-17	37.7	1	x
Q2GXB6	GH10	1-20	37.2	1	x
Q2GTY2	GH10	1-15	35.2	1	x
Q2GY31	GH10	x	16.5	x	x
Q2HA74	GH30	1-23	51.0	nd	x
Q2GUQ4	GH30	1-18	50.7	nd	x

Source: https://mycocosm.jgi.doe.gov/mycocosm/home

SP, signal peptide

MW, molecular weight

GS, glycosylation site

Data on glycosylation sites were acquired from https://services.healthtech.dtu.dk/services/NetNGlyc-1.0/

Sequences highlighted in grey were excluded from structural analysis

nd, not determined

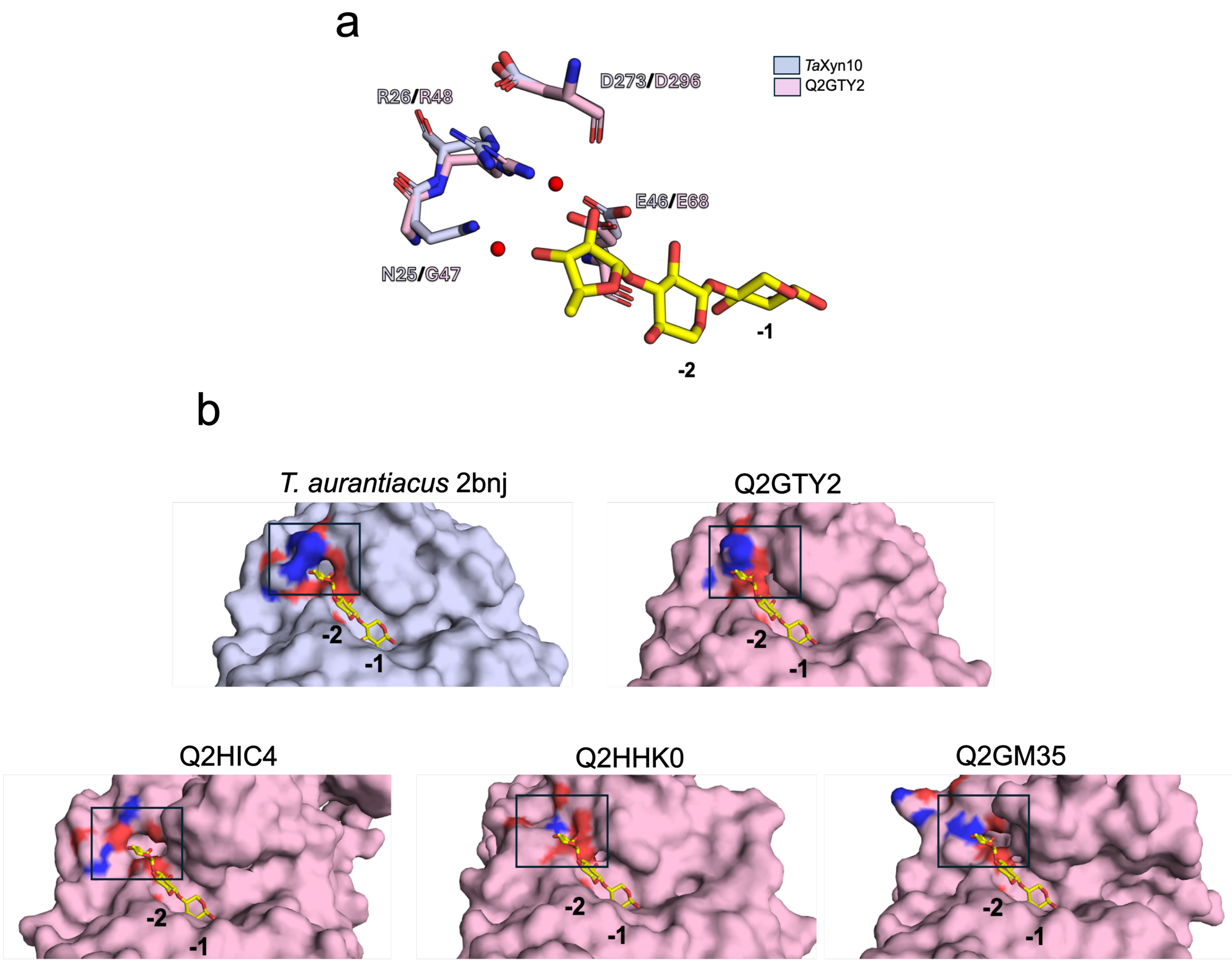

Supplementary Figure 10. Structural comparison of *C. globosum* putative xylanases with *Ta*Xyn10. **(a)** Structural alignment of *Ta*Xyn10 (light blue, PDB entry 2BNJ) and Q2GTY2 highlighting the residues that contact Ara*f* in both enzymes. Waters are represented by red circles. **(b)** Surface comparison of *C. globosum* GH10s and *Ta*Xyn10. The highlighted area (square) contains the residues hypothesised as crucial for the specificity of fungal AXXs towards Ara*f*-decorated parts of xylan.

Supplementary Table 9. Comparison of residues contacting Ara*f* in *Ta*Xyn10 and *C. globosum* GH10 xylanases

**Enzymes**	**Residues**			
*Ta*Xyn10	N25*	**R26***	**E46**	**D273**
Q2GTY2	G47	**R48**	**E68**	**D296**
Q2HIC4	G39	T40	E62	H302
Q2HHK0	Y99	H100	E120	D352
Q2GM35	R56	N57	E78	Y303

TaXyn10 from PDB entry 2bnj was used as reference

The residues highlighted in bold are conserved in Q2GTY2 and *Ta*Xyn10

The asterisks represent the residues that make indirect interaction with the ligand via water molecules

	25 26	46	273
***Bispora* sp.**	AADI**PG**LEQDDRY	GTTP**A**NIM	DDT**Y**SWVP
G. trabeum	ALGV**PH**LLNFTHD	GATP**E**NEM	TDL**Y**SWIP
F. oxysporum	ALTV**RN**DQ-GEID	SITP**E**NAM	SDK**Y**SWVP
H. chrysogenum	EIDH**YH**LN-NQPL	QVTH**E**NSM	SDK**D**SWRQ
T. reesei	ATDR**GL**LQ-REKN	QVTP**E**NSM	SDK**D**SWRA
C. globosum	CADQ**GR**LG-AGKN	QVTP**E**NSM	RDP**D**SWRP
A.nidulans	CSDQ**AL**L-QNSQN	VITP**E**NSM	SDK**D**SWRA
T. aurantiacus	ATDQ**NR**LTT-GKN	QVTP**E**NSM	ADP**D**SWRA
A.niger	IGDQ**YT**LTKNSKT	ALTP**E**NSM	ADP**D**SWRS
P. simplicissimum	IGDQ**YT**LTKNTKN	QLTP**E**NSM	ADP**D**SWRS

Supplementary Figure 11. Alignment of filamentous fungi GH10 xylanases with known crystallographic structures. Residues in bold are essential for interaction with Ara*f*. Residue numbers reflect their positions in *Ta*Xyn10, a xylanase from *T. aurantiacus* used as model in the establishment of an ABPP-SSN approach for functional annotation of xylanases.

Supplementary Table 10. Filamentous fungi GH10 xylanases used as input for SSN analysis

Microorganism	Uniprot id.
Gloeophyllum trabeum	S7Q6I2, U5HTZ3, A0A059P9J8
Podospora anserina	B2APG8, B2ADU0, B2AQD3, B2AMK1, B2AV20, B2B5D0, B2B789, B2AFS1
Neurospora crassa	Q7S3P8, Q7RW51, Q7S0Y0
Agaricus bisporus	O60206, Q9HGX1
Coprinopsis cinerea	A8P570, A8NBS6, A8P108, A8N539
Fusarium oxysporum	P46239, Q8TGC3, O93976, F9F6U4, B3A0S5, F9F9C7
Penicillium chrysogenum	P29417
Penicillium simplicissimum	B6H9S6, B6HPJ6, P56588
Aspergillus nidulans	Q00177, Q5BAS4, A1DNN0
Chaetomium globosum	Q2HIC4, Q2HHK0, Q2H7X4, Q2GXB6, Q2GM35, Q2GTY2
Ustilago maydis	Q4P902
Hapsidospora chrysogenum	A0A086SY89
Aspergillus niger	A2QFV7
Thermoascus aurantiacus	P23360
Trichoderma reesei	Q9P973

```
A8P108       -----KACVGINLASLTDK--YQILI---DAGPINPYPWDK-DLQK-KEHIYNAIRDGLV 367
Q8TGC3       ------GITDISFWGFTDKHAYTWLP------GAKPLMFNE-TYYP--KGAFYATHSALA 313
O93976       ------GITDISFWGFTDKHAYTWLP------GAKPLMFNE-TYYP--KGAFYATHSALA 312
B6H9S6       ------GITDISFWGFTDKHAYTWLP------GAKPLMFDE-CYNA--KGAFYATHAALT 312
A1DNN0       ------GVADISFWGFTDKHAYTWIE------GAKPLMFDE-YYNP--KGAFYATHTALT 311
Q5BAS4       ------GITDISFWGFTDKHAYTWVK------GAKPLMFDE-YYNP--KGAFYAIHSALT 312
Q2GXB6       -------------------------ARGRF-RGRGRRVCWTA-GMEK--KVAYARVVELLR 265
B2AQD3       -----PKCVGITVWDFTDQ--YSWIPQQY-PGEGEACLWDR-NYNK--KPAYHSIVGLLQ 318
A8N539       -----PQCVGITIWGVSDR--HTWVDGTF-PGDDSPLLWDD-NYRR--KPAYDGVNAALN 311
Q4P902.1     -----KNCVGITVWGVSDN--TSWRK------NANPLLWNS-SYQK--KPAYNAVLSTLN 319
Q7S0Y0       -----PKCVGITVWGVRDS--DSWRP------NGNPLLFDA-NWNP--KAAYNSIVQALQ 313
Q2GTY2       -----PKCVGITVWGVRDP--DSWRP------QGNPLLFDS-NYNP--KANYNAIVSLLS 310
B2ADU0       -----PKCRSITVWGVRDQ--DSWRP------QGNPLLFDS-NYNA--KANYNAIVQFLS 354
F9F9C7       -----PKCVGITVWGVSDK--DSWRP------GENPLLYDS-NYQP--KAAFNALVNALM 368
P46239       -----PKCVGITVWGVSDK--DSWRP------GDNPLLYDS-NYQP--KAAFNALANAL- 332
A0A086SY89   -----EKCVGITVWGVSDK--DSWRQ------GDNPLLFDN-NYQP--KAAYHALVNAL- 32
Q2HHK0       -----SKCVGITVWGVSDK--DSWRA------NDNPLLFDR-NYQP--KAAYNALVNAL- 324
B2AMK1       -----PKCVGITVWGVSDK--DSWRS------SDNPLLFDR-NYQP--KAAYNALMNAFV 332
A8NBS6       -----PLCIGITVWGVSDA--NSWRS------STNPLLFDR-NYQP--KAAYNAVSQML- 318
F9F6U4       -----PKCIGITVWGVSDK--NSWRK------EHDSLLFDA-NYNP--KAAYTAVVNALR 313
Q9P973       -----SKCVGITVWGISDK--DSWRA------STNPLLFDA-NFNP--KPAYNSIVGILQ 331
Q00177       -----QKCVGITVWGVSDK--DSWRA------SDSPLLFDG-NYQP--KDAYNAIVNALS 303
Q9HGX1       -----PACVSITTWGVADI--NSWRS------GSSPLLFDN-NYRP--KAAYNAVINA-- 277
P23360       -----QSCVGITVWGVADP--DSWRA------STTPLLFDG-NFNP--KPAYNAIVQDLQ 302
P29417       -----PKCVGITVWGVADP--DSWRA------DESPLLFDA-SYNP--KEAYNVSQLLSR 309
A2QFV7       -----PKCIGITVWGVADP--DSWRS------SSTPLLFDS-NYNP--KPAYTAIANAL- 308
P56588       -----AKCVGITVWGVADP--DSWRS------SSSPLLFDG-NYNP--KAAYNAIANAL- 302
S7Q6I2       ----GAACVGITTWGITDL--YSWIPSTY-PGEGYALLFDD-NYVP--HPAFNATIQALL 327
B2B789       ----KDVCVGITIWDFYDP--FSWVPHVF-PGNGASLLWFE-DFSK--HPAYDGMVNYFG 327
Q2H7X4       -----CGCIGVTIWDFYDP--FSWVPFVF-EGEGAALLWFE-DFSK--HPAYDGVVEALT 276
B2AV20       -----KGCIGVTIWDFYDP--FSWVPYVF-PGEGAPLLWFE-DWSK--HPAYYGVQEALK 337
Q7S3P8       -----DGCIGVTIWDFYDP--FSWVPAVF-PGEGAALLWFD-DFSR--HPAYEGVVEALT 345
B3A0S5       -----KRCVGITVWGISDK--YSWVPGTF-PGEGSALLWND-NFQK--KPSYTSTLNT-- 323
Q2GM35       -----PSCVGMTIWGVTDR--YSWVPNTF-QGEGDALLW-NSNYQ--KKAAYTSFLEG-- 318
B2APG8       -----PRCVGMTIWVRPLD--MTSLVLQLLTVRNRASVTGTRGFPRLSRVKVTRFFGTTT 341
B2B5D0       -----ERCVGVTVWQFTDR--YSWVPGTF-PGKGGACLWTE-DYQR--KPAYWAVKRFLE 329
Q7RW51       -----DGCVGVTIWGFIDK--YNWIQ------NGNAAIFDS-SFNK--KPAYSAISSVLA 316
Q2HIC4       -----DGCIGVTVWGFTDK--HSWIPSTF-PGEGDAMIYDS-NYNK--KPAWTSISSVLA 319
B2AFS1       -----PRCVGVTVWSFTDK--YSWVPSTF-NGAGDALIYDS-QFRK--KAAWTSISSVLA 319
B6HPJ6       -----TKCVGITIWDYTDK--YSWVPSTF-PGQGDACPWDK-NLVK--KPAYAGILAALG 318
A8P570       -----KRCVGITIWDWTDK--YSWVPGWF-EGEGAALPWDE-DFKK--KPAYYGIVEG-- 313
O60206.1     -----SRCVGVTVWDWTDR--YSWVPGVF-NGEGAACPWDE-NLAK--KPAYQGIVDGWS 315
U5HT23       -----SGCIGVTIWDYTDK--YSWVPSTF-SGQGAALPWDA-NLNK--KPAYDGIVAGFA 377
A0A059P9J8   -----SGCVGVTVWDFTDK--YSWVPSTF-SGQGAATPYDS-NLVK--KPAYDGIVAGWQ 333
```

Supplementary Figure 12. Alignment of filamentous fungi GH10 xylanases used as input in the SSN analysis. The Uniprot ids are colour-coded as the clusters observed in Fig. 6a. The residue highlighted in red corresponds to Asp273 in *Ta*Xyn10.

Supplementary Table 11. Purification steps used for each protein

Protein	Buffers – purification steps
*Ct*Xyn30A	Buffer A: 50 mM HEPES, pH 7.5; 1000 mM NaCl, 10 mM Imidazole, 3mM $CaCl_2$
	Buffer B: 50 mM HEPES, pH 7.5; 1000 mM NaCl, 300 mM Imidazole, 3mM $CaCl_2$
	Buffer GF: 50 mM HEPES, pH 7.5; 200 mM NaCl, 5mM $CaCl_2$
*Ct*Xyl5A	Buffer A: 20 mM Tris-HCl, pH 8.0; 300 mM NaCl, 5 mM Imidazole
	Buffer B: 20 mM Tris-HCl, pH 8.0; 300 mM NaCl, 500 mM Imidazole
	Buffer GF: 20 mM Tris-HCl, pH 8.0; 150 mM NaCl
XynA	Buffer A: 40 mM Tris-HCl, pH 8.0; 250 mM NaCl, 10 mM Imidazole
	Buffer B: 40 mM Tris-HCl, pH 8.0; 250 mM NaCl, 500 mM Imidazole
	Buffer GF: 40 mM Tris-HCl, pH 8.0; 150 mM NaCl, 1mM DTT
	*Buffer GF: 10 mM HEPES, pH 7.0; 150 mM NaCl
*Ta*Xyn10	Buffer A: 20 mM sodium phosphate, pH 7.4; 500 mM NaCl; 5 mM Imidazole
	Buffer B: 20 mM sodium phosphate, pH 7.4; 500 mM NaCl, 500 mM Imidazole
	Buffer GF: 20 mM sodium phosphate, pH 7.4; 150 mM NaCl
*So*Xyn10A	Buffer A: 20 mM sodium phosphate, pH 7.4; 500 mM NaCl; 5 mM Imidazole
	Buffer B: 20 mM sodium phosphate, pH 7.4; 500 mM NaCl, 200 mM Imidazole
	Buffer GF: 20 mM sodium phosphate, pH 7.4; 150 mM NaCl

Buffer A and buffer B are used for affinity purification step. GF, gel filtration.
*Buffer used for crystallization assays.

References

1. Schröder, S.P. et al. A Divergent Synthesis of l-arabino- and d-xylo-Configured Cyclophellitol Epoxides and Aziridines. *European Journal of Organic Chemistry* **2016**, 4787-4794 (2016).
2. de Boer, C. Doctoral, Leiden University (2021).
3. Crich, D., Pedersen, C.M., Bowers, A.A. & Wink, D.J. On the use of 3,5-O-benzylidene and 3,5-O-(di-tert-butylsilylene)-2-O-benzylarabinothiofuranosides and their sulfoxides as glycosyl donors for the synthesis of beta-arabinofuranosides: importance of the activation method. *J Org Chem* **72**, 1553-65 (2007).
4. de Boer, C. et al. Glycosylated cyclophellitol-derived activity-based probes and inhibitors for cellulases. *RSC Chem Biol* **1**, 148-155 (2020).
5. Diederichs, K. & Karplus, P.A. Improved R-factors for diffraction data analysis in macromolecular crystallography. *Nat Struct Biol* **4**, 269-75 (1997).
6. Weiss, M.S., Metzner, H.J. & Hilgenfeld, R. Two non-proline cis peptide bonds may be important for factor XIII function. *FEBS Lett* **423**, 291-6 (1998).
7. Karplus, P.A. & Diederichs, K. Linking Crystallographic Model and Data Quality. *Science* **336**, 1030-1033 (2012).
8. Cruickshank, D.W.J. Remarks about protein structure precision (vol 55, pg 583, 1999). *Acta Crystallographica Section D-Biological Crystallography* **55**, 1108-1108 (1999).
9. Labourel, A. et al. The Mechanism by Which Arabinoxylanases Can Recognize Highly Decorated Xylans. *J Biol Chem* **291**, 22149-22159 (2016).
10. Vardakou, M. et al. A family 10 Thermoascus aurantiacus xylanase utilizes arabinose decorations of xylan as significant substrate specificity determinants. *J Mol Biol* **352**, 1060-7 (2005).
11. Bowman, M.J., Dien, B.S., O'Bryan, P.J., Sarath, G. & Cotta, M.A. Selective chemical oxidation and depolymerization of switchgrass [corrected] (Panicum virgatum L.) xylan with [corrected] oligosaccharide product analysis by mass spectrometry. *Rapid Commun Mass Spectrom* **25**, 941-50 (2011).
12. Feijao, C. et al. Hydroxycinnamic acid-modified xylan side chains and their cross-linking products in rice cell walls are reduced in the Xylosyl arabinosyl substitution of xylan 1 mutant. *Plant J* **109**, 1152-1167 (2022).
13. Hamann, P.R.V. & Noronha, E.F. Xylan-breakdown apparatus of Clostridium thermocellum. *Cellulose* **29**, 7535-7553 (2022).

Raw data

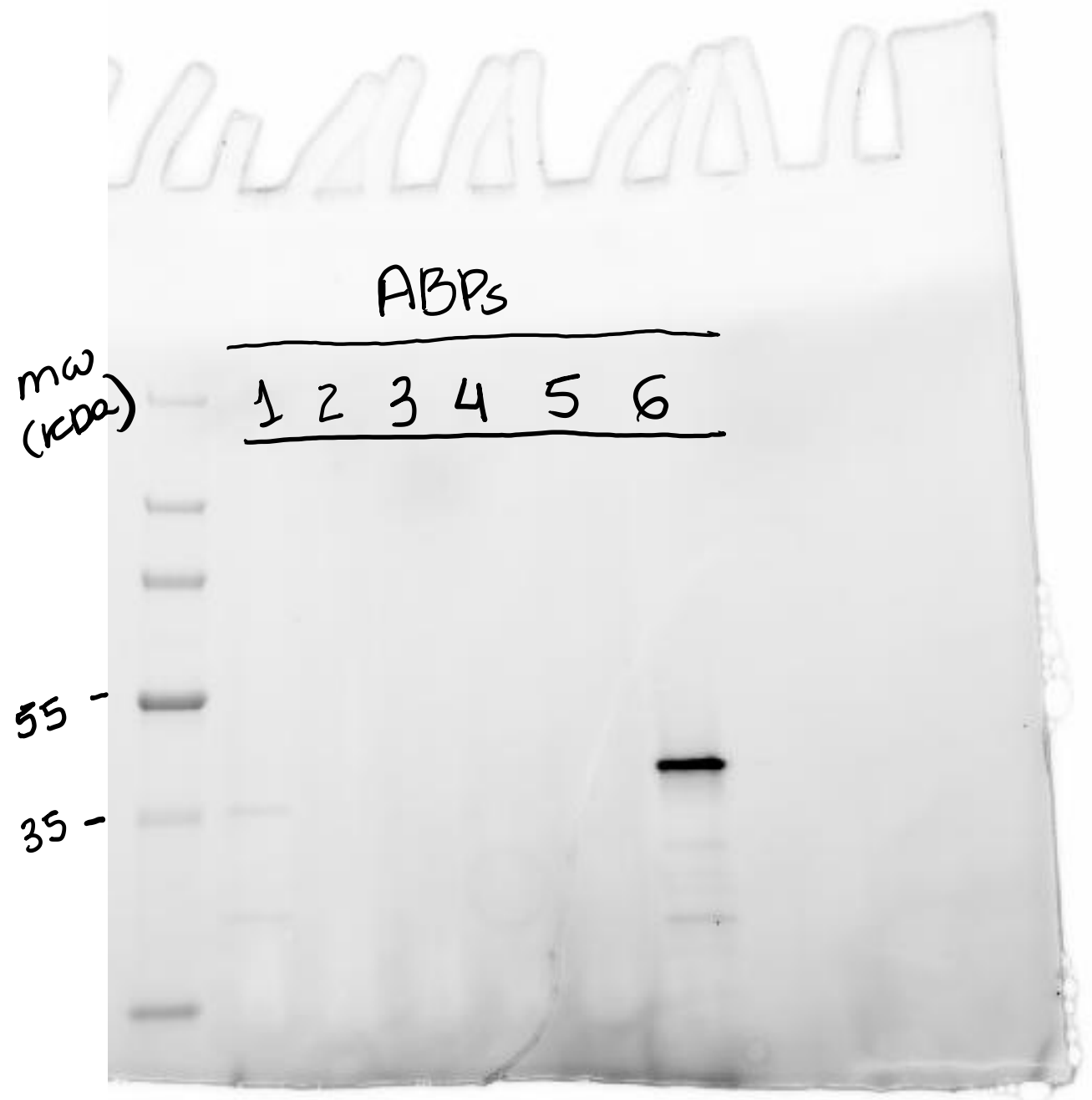

Supplementary figure 8

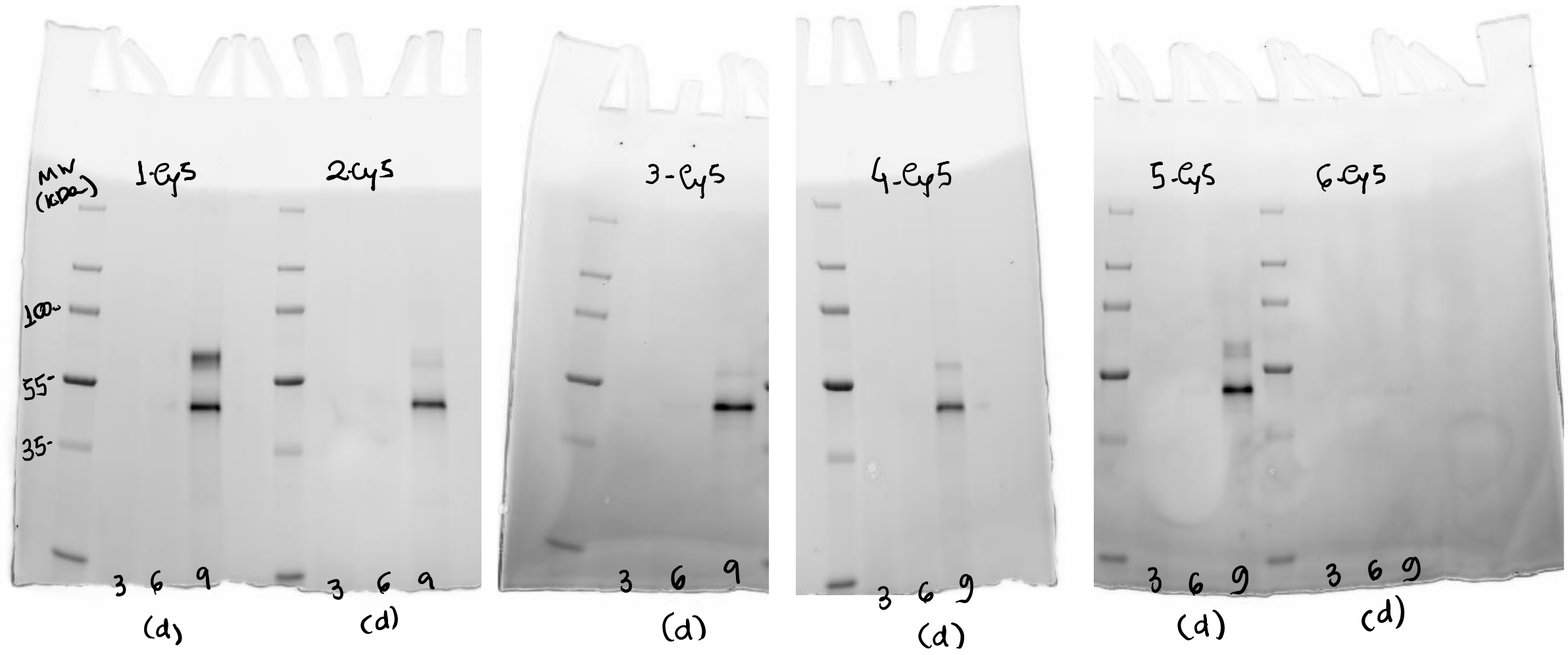

Supplementary Figure 9
