# Supplementary material for: A chemoproteomic biotechnological toolkit for resolving xylanase specificity in decorated xylan
Source: Nat Commun. 2026 Jun 22;17:7860. doi: 10.1038/s41467-026-74484-0 (PMC13444322; doi:10.1038/s41467-026-74484-0)
Supplement: Supplementary file 3 [file 41467_2026_74484_MOESM3_ESM.pdf]

nature portfolio

Peer Review File

A Chemoproteomic Biotechnological Toolkit for Resolving Xylanase Specificity in Decorated Xylan

Corresponding Author: Professor Gideon Davies

This file contains all reviewer reports in order by version, followed by all author rebuttals in order by version.

Version 0:

Reviewer comments:

Reviewer #1

(Remarks to the Author)
This study investigates a method of overcoming the limitations of annotating the specific activities of carbohydrate-active enzymes (CAZymes) using activity-based probes (ABPs), which target these glycan-processing enzymes. The authors focus on branched and decorated xylans. They designed a specific set of ABPs to mimic the molecular features of arabinoxylan and glucuronoxylan in order to determine the potential activities of xylanases specific to these substrates. The selective covalent labelling strategy presented in this study enables the specific xylan activities of enzymes to be distinguished within complex biological mixtures.
Gel-based analysis of bound, fluorescence-labelled probes was supported by LC-MS/MS analysis of ABP-labelled enzymes. The authors convincingly demonstrate that, when supported by a pull-down assay, the chemoproteomic method confirms the specific binding of the ABPs and enables specific enzymes to be analysed in biological cell samples.

The convincing approach taken in this study is of general interest and could also be applied to determine the activities of other CAZymes, as the authors have already suggested in previous studies.
The chemoproteomic approach presented could be useful for identifying new enzyme activities in unknown samples or analysing enzyme candidates where crystallographic data and/or overexpression and purification were unsuccessful.

Overall, this is a solid study. The data presented support the conclusions, the methodology is sound, and the work meets the expected standards.

Minor comments:

Abstract: define „ABPP“

Page 6, line 11: explain „catalytic TEMPO“

Legend Figure 2: In the legend text, you could indicate that the ABPs 1-6 are the ABPs with the corresponding numbers shown in Figure 1. Although obvious, you could also indicate that the control SDS-gel with loaded enzymes is shown on the right. Incidentally, the short name shown here for each individual enzyme is not very visible and could be improved.

Figure 4: Have you tried analysing the fluorescently labelled protein bands shown in figure 4a by mass spectrometry to identify the specific proteins to which they correspond?

Methods/ Protein production and purification:
Please provide the codon optimization details. Which of the the pET28a, pET21a, pET28a, and pET21a vectors were used for which enzyme?

Supplementary information, page 4: Move „General procedure A“ to the next page.
Supplementary information, page 5: Move „Compound S3“ to the next page
... and so on

Supplementary information, NMR spectra: It would be better to indicate the structures using the names given in the descriptions shown previously.

Supplementary Figure 4. How much B. subtilis supernatant was added on the SDS-gel?
In general, please provide this information also for all the other SDS-PAGE gel analyses, where secretomes were analysed.

Supplementary Table 10: Please give the genus names of the fungi.

Throughout the main text and supplementary information: Check space before „°"

Reviewer #2

(Remarks to the Author)
This research provides advancements in mechanism-based chemical probes to target endo-retaining glycoside hydrolases with different xylan-related specificities. It provides important developments for selectively identifying retaining xylanases in complex proteomes. There are limitations that need to be addressed in characterising probe mechanism, but once addressed, this study will be appropriate for publication in Nature Communications.

It would have been helpful to have a version with line numbering.

Define ABPP in the abstract.

Dynamic polysaccharide degradation. Not polysaccharide's.

Better define the xylanases you are targeting with these mechanistic probes. Endo-acting retaining xylanases at their -1, -2 subsites. Vs Exo or Inverting.

'A virtually unlimited source of energy for onward conversion into green chemicals' Unnecessarily grandiose and unclear. Remove.

'… via retaining double-displacement mechanism' Add period.

From the text it wasn't clearly explained why the glycosyl enzyme intermediate with the probes are stable and why they are not hydrolysed in the second step.

'…in which a glutamic acid residue' glutamate.

'… that selectively and covalently label AXXs and GXXs' change to '…that selectively and covalently label endo-retaining AXX and GXX specific xylanases'

Fig 1. Add a panel to show the reaction mechanism of probe inhibition/formation of 'stable' adduct glycosyl-enzyme intermediate and show why the reaction doesn't proceed after formation of glycosyl-enzyme intermediate.

Fig1a. what is going on with the glu acid/base arrows?

Fig1b. Put the chemical numbering in order on the polysaccharide. For example 1 and 2 on top 3,4 , 5, 6 on the bottom. Currently it reads 2,6 then 1, 4, 3, 5. There is no order.

Remove the n and brackets from the polysaccharide structure, this implies repeating motifs. Use dotted lines at each end.

Add the Me group to compound 6

Add compound S7 to fig1 as it is a complex shown in fig3.

Figure 1 title. Glutamate nucleophile, not glutamic acid.

Scheme 1. Show full chemical structure of the final product with the 6-Cy5 and the 4-Bio on the non-reducing ends of the probes with the simplified shape schematic beside it.

Have you encountered any off target labelling of your enzymes, for example labelling of the catalytic acid/base rather than the nucleophile or other residues? This would be interesting to note at least in the supplementary section for future reference.

From the Fig 3a mtz which was provided, the experimental electron density of the ABP-3Bio with XynA it doesn't look like there is a covalent bond with the catalytic nucleophile even though it is modelled as such. The electron density looks planar at the anomeric carbon and the xylose seems mis-modelled in a chair conformation. I am unconvinced this is a covalent bond from the data provided. Show the difference and weighted difference omit map for this complex, omitting both the

glutamate nucleophile and the probe from the refinement.

While some of the probes may be stably maintained in a covalent manner in some of the xylanases' active sites, at least with ABP-3Bio and XynA, this might not be the case. Mass spec analysis also showed only partial covalent labelling of CtXyl5A. Thus, the xylanase pull down assays may be due to probe covalently linked to the enzymes or they may be related to simple enzyme affinity for the product complex of the non-covalently-linked probe after hydrolysis of the enzyme-glycosyl intermediate or some combination of covalent and non-covalent interaction. It is difficult to distinguish the options as the study is missing inactivation kinetics. To confirm probe mechanism, probe inactivation kinetics (for Cy5 and Bio) should be demonstrated with the control xylanases (CtXyl5A, CtXyn30A, XynA, TaXyn10) where an important probe interaction is seen.

The data for the pull down for CtXyn30A by 2-bio and 4-bio is contradictory to the Fig2b Cy5 labelling data. Can you offer an explanation for this? Is it possible the probe tail for Cy5 is interfering with the interaction?

Fig4f title. By 6b do you mean 6-Bio?

'Notwithstanding the caveat that current ABP target only the negative subsites' of endo-retaining xylanases.

Conclusion: '…selective targeting' the negative subsites of endo-retaining AXX and GXX xylanases.

Reviewer #3

(Remarks to the Author)
The authors describe a biotechnological strategy toolkit for discovering and engineering substrate-specific Xylanases with applications in biomass utilisation and microbial ecology.

The activity-based probes developed alongside the use of structural, biochemical analysis and sequence similarity networks have not only enabled the precise mapping of substrate specificity of Xylanases but also revealed the determinant of probe selectivity. Through their combinatorial approach they were able to establish a predictive platform for the functional annotation of enzyme from sequence information.

As such, I believe this platform will be of great interest to the world of enzyme discovery, engineering for a number of biotechnological applications.

The authors describe the design and synthesis of the activity-based probes and provide useful structural and biochemical insights into the mechanism of recognition and selectivity. They validated their findings extensively using mass spectrometry. I felt that this was very important. They demonstrate that their probes are useful across complex bacterial and fungal systems as well as cellulosomal proteomes central in biomass degradation. Lastly, they show the reader that combining their probes with sequence similarity network analysis is a powerful approach to functionally annotate xylanases.

The manuscript is comprehensive and well-written - save for some minor points that I think should be addressed (see below), I have no hesitation in recommending that this paper be published.
Minor:
Figure 5. I found the sequence similarity network a little hard to read when printed out. I would recommend increasing the size of it by 20%.

Methods:
The two sections: Crystal plates set-up and Crystal structure solution and refinement ready a bit duplicated. Please move everything for crystallisation into the top section. The Crystal structure solution section should start with : Diffraction data collected at ….

Version 1:

Reviewer comments:

Reviewer #1

(Remarks to the Author)
The authors have satisfactorily addressed all critical points.

Reviewer #2

(Remarks to the Author)
The authors have adequately addressed my comments. This work provides significant advancement to the structural and biochemical understanding of mechanism-based probes and merits publication in Nature Communications.

Reviewer #3

(Remarks to the Author)
The authors have addressed the reviewers comments. The revised manuscript with figures reads very well now and this work will be of significance to the field.

Open Access This Peer Review File is licensed under a Creative Commons Attribution 4.0 International License, which permits use, sharing, adaptation, distribution and reproduction in any medium or format, as long as you give appropriate credit to the original author(s) and the source, provide a link to the Creative Commons license, and indicate if changes were made.
In cases where reviewers are anonymous, credit should be given to 'Anonymous Referee' and the source.
The images or other third party material in this Peer Review File are included in the article's Creative Commons license, unless indicated otherwise in a credit line to the material. If material is not included in the article's Creative Commons license and your intended use is not permitted by statutory regulation or exceeds the permitted use, you will need to obtain permission directly from the copyright holder.
To view a copy of this license, visit https://creativecommons.org/licenses/by/4.0/

Please find below our responses to the referee comments and suggestions.

We thank them for their hard work and time to help us produce a better submission.

REVIEWER COMMENTS

Reviewer #1 (Remarks to the Author):

We thank the reviewer for the general comments.

Minor comments:

- Abstract: define "ABPP"

RESPONSE: The definition of ABPP (activity-based protein profiling) has been added to the abstract (page 1, in red)

- Page 6, line 11: explain "catalytic TEMPO"

RESPONSE: TEMPO stands for 2,2,6,6-Tetramethylpiperidine 1-oxyl. This information has been added to the manuscript (page 7, in red).

- Legend Figure 2: In the legend text, you could indicate that the ABPs 1-6 are the ABPs with the corresponding numbers shown in Figure 1. Although obvious, you could also indicate that the control SDS-gel with loaded enzymes is shown on the right. Incidentally, the short name shown here for each individual enzyme is not very visible and could be improved.

RESPONSE: We have clarified that the ABPs shown in Fig. 2 follow the same numbering as adopted in Fig. 1, and that the Coomassie blue–stained gel is shown on the right. In addition, the protein names were moved to the upper part of the Coomassie gel to improve visualisation.

- Figure 4: Have you tried analysing the fluorescently labelled protein bands shown in figure 4a by mass spectrometry to identify the specific proteins to which they correspond?

RESPONSE: **Cy5**-tagged compounds **1-6** were used for fluorescent detection, while the corresponding biotinylated compounds were employed for enrichment and protein identification. The mass spectrometry-based identification of the proteins labelled in Fig. 4a is shown in Fig. 4b–d and Supplementary Fig. 4. The corresponding results are discussed on page 14-15, in red).

- Methods/ Protein production and purification:
Please provide the codon optimization details. Which of the pET28a, pET21a, pET28a, and pET21a vectors were used for which enzyme?

RESPONSE: Codon optimization has now been clearly specified for each enzyme, together with the target organism and expression vector used (page 23-24, in red).

- Supplementary information, page 4: Move "General procedure A" to the next page. Supplementary information, page 5: Move "Compound S3" to the next page
... and so on

RESPONSE: All of these have been moved as suggested.

- Supplementary information, NMR spectra: It would be better to indicate the structures using the names given in the descriptions shown previously.

RESPONSE: The structures are now identified using the nomenclature previously established in the manuscript.

- Supplementary Figure 4. How much B. subtilis supernatant was added on the SDS-gel? In general, please provide this information also for all the other SDS-PAGE gel analyses, where secretomes were analysed.

RESPONSE: A total of 10 uL of supernatant (0.5–1.0 mg/mL protein) was loaded on the SDS-PAGE gel in experiments with secretomes. This information has been added to the Materials and Methods section ("Production of secretomes"- page 28, in red).

- Supplementary Table 10: Please give the genus names of the fungi.

RESPONSE: The genus names have been included.

- Throughout the main text and supplementary information: Check space before "°"

RESPONSE: All of these have been corrected in the revised manuscript.

<u>Reviewer #2 (Remarks to the Author):</u>

- It would have been helpful to have a version with line numbering.

RESPONSE: Line numbers have been added to the revised version of the manuscript.

- Define ABPP in the abstract.

RESPONSE: The definition of ABPP (activity-based protein profiling) has been added to the abstract (page 1, in red).

- Dynamic polysaccharide degradation. Not polysaccharide's.

RESPONSE: This has now been corrected (page 2, in red).

- Better define the xylanases you are targeting with these mechanistic probes. Endo-acting retaining xylanases at their -1, -2 subsites. Vs Exo or Inverting.

RESPONSE: This information is now introduced in page 4 (3rd paragraph, in red). The target subsites are highlighted in the legend of Fig.1 (page 5-6, in red).

- 'A virtually unlimited source of energy for onward conversion into green chemicals' Unnecessarily grandiose and unclear. Remove.

RESPONSE: This, indeed inappropriate, sentence was removed.

- '… via retaining double-displacement mechanism' Add period.

RESPONSE: The missing period has been added (page 2, 3rd paragraph, in red).

- From the text it wasn't clearly explained why the glycosyl enzyme intermediate with the probes are stable and why they are not hydrolysed in the second step.

RESPONSE: We have added more details on why the probes are stable and not susceptible to hydrolysis: "Cyclophellitol epoxides and aziridines are weak electrophiles that are protonated by the catalytic acid/base residue. This protonation stabilises the developing positive charge and facilitates nucleophilic attack by the catalytic carboxylate. The resulting covalent adduct is not a productive glycosyl-enzyme intermediate and blocks water-mediated turnover, rendering these probes highly selective, mechanism-based irreversible inhibitors" (page 3, in red).

- '…in which a glutamic acid residue' glutamate.

RESPONSE: Glutamic acid has been changed to glutamate (page 4, 2nd paragraph, in red).

- '… that selectively and covalently label AXXs and GXXs' change to '…that selectively and covalently label endo-retaining AXX and GXX specific xylanases'

RESPONSE: The sentence has been modified (page 4, 3rd paragraph, in red).

- Fig 1. Add a panel to show the reaction mechanism of probe inhibition/formation of 'stable' adduct glycosyl-enzyme intermediate and show why the reaction doesn't proceed after formation of glycosyl-enzyme intermediate.

RESPONSE: The new panel was added to Fig. 1 as Fig. 1b. The legend was modified accordingly.

- Fig1a. what is going on with the glu acid/base arrows?

RESPONSE: The arrows have been corrected.

- Fig1b. Put the chemical numbering in order on the polysaccharide. For example 1 and 2 on top 3,4 , 5, 6 on the bottom. Currently it reads 2,6 then 1, 4, 3, 5. There is no order.

RESPONSE: Fig. 1b has been modified. Compounds **1, 3**, and **5** are displayed on the bottom, and **2, 4,** and **6** on the top.

- Remove the n and brackets from the polysaccharide structure, this implies repeating motifs. Use dotted lines at each end.

RESPONSE: Both "n" and brackets have been removed from the glucuronoarabinoxylan structure.

- Add the Me group to compound 6

RESPONSE: The CH3 group has been added to compound **6** – Fig. 1.

- Add compound S7 to fig1 as it is a complex shown in fig3.

RESPONSE: Compound **S7** has been added to Fig. 1. The legend was modified accordingly.

- Figure 1 title. Glutamate nucleophile, not glutamic acid.

RESPONSE: “Glutamic acid” has been changed to glutamate.

- Scheme 1. Show full chemical structure of the final product with the 6-Cy5 and the 4-Bio on the non-reducing ends of the probes with the simplified shape schematic beside it.

RESPONSE: Scheme 1 has been updated to include the **6-Cy5** and **4-Bio** compounds, each bearing tags at the non-reducing ends.

- Have you encountered any off-target labelling of your enzymes, for example labelling of the catalytic acid/base rather than the nucleophile or other residues? This would be interesting to note at least in the supplementary section for future reference.

RESPONSE: We thank the reviewer for this important point. Under the conditions used in this study, we did not observe evidence for covalent labelling of residues other than the catalytic nucleophile in either our mass spectrometry analyses or our structural data. This is consistent with the expected behaviour of cyclophellitol-derived mechanism-based probes for retaining glycosidases, theoretically and as observed in published studies. We have now added a clarifying sentence to the manuscript to make this explicit (Legend of Fig. 3, in red).

- From the Fig 3a mtz which was provided, the experimental electron density of the ABP-3Bio with XynA it doesn’t look like there is a covalent bond with the catalytic nucleophile even though it is modelled as such. The electron density looks planar at the anomeric carbon and the xylose seems mis-modelled in a chair conformation. I am unconvinced this is a covalent bond from the data provided. Show the difference and weighted difference omit map for this complex, omitting both the glutamate nucleophile and the probe from the refinement.

RESPONSE: We thank the reviewer for carefully examining the electron density in this region and for raising this important point.

We agree that the density around the anomeric carbon is not consistent with a single, fully occupied covalent adduct. In particular, the maps indicate partial planarity at C1 and do not support modelling the ligand solely as the reacted epoxide.

To address this directly, we calculated both standard omit maps and weighted difference omit maps after removing the ligand and the side chain of the catalytic

nucleophile (Glu280), followed by re-refinement. Refinement with the ligand modelled exclusively in either the unreacted or covalently linked state did not satisfactorily account for the observed density. In the fully unreacted model, the anomeric carbon approaches Glu280 at an implausibly short distance, whereas the fully covalent model leaves residual features unexplained.

Taken together, these observations are most consistent with a dual-occupancy model comprising both intact epoxide and covalently reacted glycosyl–enzyme states. We have therefore re-refined the structure accordingly, with both the ligand and Glu280 modelled in alternative conformations with refined occupancies.

The re-refined model has been deposited in the PDB under accession code 9RHV. Omit-map coefficients are provided in the Supplementary Information, representative maps are shown in Supplementary Fig. 1, and the main text and Fig. 3b have been revised to reflect the mixed-state interpretation.

- While some of the probes may be stably maintained in a covalent manner in some of the xylanases' active sites, at least with ABP-3Bio and XynA, this might not be the case. Mass spec analysis also showed only partial covalent labelling of CtXyl5A. Thus, the xylanase pull down assays may be due to probe covalently linked to the enzymes or they may be related to simple enzyme affinity for the product complex of the non-covalently-linked probe after hydrolysis of the enzyme-glycosyl intermediate or some combination of covalent and non-covalent interaction. It is difficult to distinguish the options as the study is missing inactivation kinetics. To confirm probe mechanism, probe inactivation kinetics (for Cy5 and Bio) should be demonstrated with the control xylanases (CtXyl5A, CtXyn30A, XynA, TaXyn10) where an important probe interaction is seen.

RESPONSE: We agree that defining the mechanism and extent of covalent modification is important for interpreting the pull-down experiments. We also agree that inactivation kinetics would, in principle, be informative. However, we have not included such experiments in the present revision because the required substrate series is not currently available. Specifically, these measurements would require the synthesis of panel of bespoke branched oligosaccharides bearing suitable leaving groups across the relevant substitution patterns, and to our knowledge such compounds have not been described. Their preparation would require a substantial synthetic effort beyond the scope of the present revision. Indeed, one of the motivations for the present probe-development study was the lack of suitable substrates for selectively interrogating these enzymes.

To address the reviewer's concern as directly as possible, we instead performed intact-mass spectrometry on the biotinylated probes for *Ct*Xyl5A, XynA, *Ta*Xyn10 and *Ct*Xyn30A. Because intact MS directly reports the expected mass shift of the enzyme–probe conjugate, it provides direct evidence for covalent adduct formation in solution. These new data are now included in Supplementary Fig. 3, page 12 (3rd paragraph, in red), and page 13, in red.

GH5:
*Ct*Xyn5A showed partial and full conversion to CtXyn5A-**4-Bio** after 1 h or 16 h of incubation, respectively.

GH10:
XynA reached full conversion after 1 h incubation with **3-Bio** (used for XynA crystallography). In contrast, less than 10% of XynA was converted to XynA-**1Bio** even after 16 h of incubation, suggesting that the decorated backbone favoured the catalytic site compared to the substituted xylobiose moiety of **1-Bio**.

These findings are consistent with the *C. thermocellum* pull-downs presented in this work (Fig. 4b-d), where AXX/GXX are not enriched under **1-Bio** conditions. Further corroboration comes from competition assays, in which the reference enzyme used for our SSN analysis (Tr-Q9P973, known to accept decorated substrates) required higher **1-Bio** loadings than **3-Bio** to achieve inhibition (Fig. 5c).

The same response was observed for *Ta*Xyn10.

To address the, apparent, disconnect between **Bio-** and **Cy5-**tagged probes, we incubated XynA and *Ta*Xyn10 with **1-Cy5** and **3-Cy5**. Under these conditions, full conversion was observed after 1 h incubation, agreeing with in-gel data:

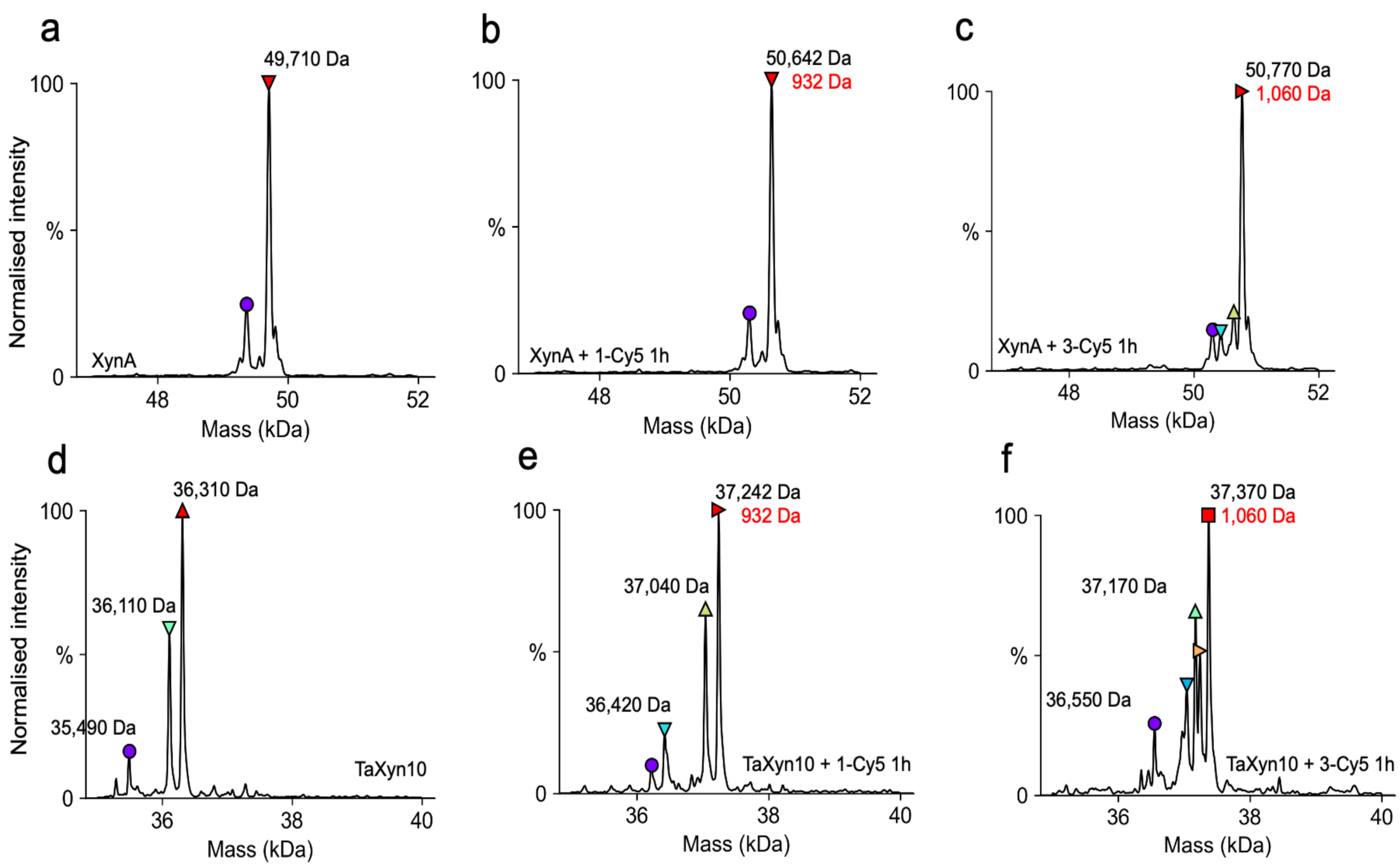

Figure 1. Intact mass spectrometry of XynA (a-c) and *Ta*Xyn10 (d-f). XynA (a) reveals full conversion to XynA-**1-Cy5** (b) and XynA-**3-Cy5** (c), with a mass shift consistent with formation of a single covalent adduct (**1-Cy5** predicted: +931 Da; observed: +932. **3-Cy5** predicted: +1,063 Da; observed: +1,060). The same procedure was adopted for *Ta*Xyn10 (a), *Ta*Xyn10-**1-Cy5** (b) and *Ta*Xyn10-**3-Cy5** (c).

These findings suggest that, in solution, contacts between the specificity motif and the enzyme are critical for accommodating the more rigid **biotin** linkers, whereas the longer and more flexible **Cy5** linker can promote accommodation of suboptimal configured sugars. This interpretation is consistent with the

crystallographic data: XynA-**3-Bio** yielded defined complexes, whereas no ligand density was observed for **1-Bio**. In contrast, neither **Cy5**-based probe produced crystals in co-crystallisation assays or ligand density upon soaking, likely due to fluorophore disorder and lattice constraints.

GH30:

In the GH30 family, full conversion of *Ct*Xyn30A was observed with **S7** (GXX inhibitor used to obtain CtXyn30A complexes)

Taken together, these intact-mass data show that the biotinylated probes form covalent adducts in solution and are consistent with the pull-down results. These findings indicate that covalent modification makes a substantial contribution to pull-down enrichment, while not excluding a minor contribution from non-covalent interactions in some cases.

- **The data for the pull down for CtXyn30A by 2-bio and 4-bio is contradictory to the Fig2b Cy5 labelling data. Can you offer an explanation for this? Is it possible the probe tail for Cy5 is interfering with the interaction?**

RESPONSE: This apparent discrepancy likely reflects the different experimental contexts of the two readouts. The in-gel labelling experiments were performed with purified *Ct*Xyn30A and therefore primarily report direct covalent labelling of the isolated enzyme. By contrast, the pull-down experiments were performed on *C. thermocellum* cellulosomal secretomes, in which enzymes are present within a tightly associated multi-enzyme complex linked by high-affinity cohesin-dockerin interactions.

Accordingly, enrichment in the pull-down experiment does not necessarily imply the same degree of direct labelling observed for the purified enzyme. We therefore consider it plausible that *Ct*Xyn30A was co-enriched under some probe conditions through association with other labelled cellulosomal xylanases. This interpretation is consistent with the stronger enrichment observed with **6-Bio**, which matches the purified-enzyme behaviour more closely, and the more moderate enrichment observed with **2-Bio** and **4-Bio**. We have now clarified this point in the manuscript (page 15, in red).

In summary, the purified-enzyme experiments indicate clear probe preferences at the level of covalent labelling, whereas the broader enrichment patterns observed in complex samples likely reflect the additional influence of sample context, including lower-efficiency interactions and possible co-enrichment.

- Fig4f title. By 6b do you mean 6-Bio?

RESPONSE: “6b” has been changed to 6-Bio in Fig. 4f title.

- 'Notwithstanding the caveat that current ABP target only the negative subsites' of endo-retaining xylanases.

RESPONSE: The sentence has been modified (page 20, in red).

- Conclusion: '…selective targeting' the negative subsites of endo-retaining AXX and GXX xylanases.

RESPONSE: The sentence has been modified (page 22, in red).

<u>Reviewer #3 (Remarks to the Author):</u>

I believe this platform will be of great interest to the world of enzyme discovery, engineering for a number of biotechnological applications.

The manuscript is comprehensive and well-written - save for some minor points that I think should be addressed (see below), I have no hesitation in recommending that this paper be published.

Many thanks for this generous summary of the work.

Minor:
- Figure 5. I found the sequence similarity network a little hard to read when printed out. I would recommend increasing the size of it by 20%.

RESPONSE: Figure 5 was revised to improve readability.

- Methods:
The two sections: Crystal plates set-up and Crystal structure solution and refinement ready a bit duplicated. Please move everything for crystallisation into the top section. The Crystal structure solution section should start with : Diffraction data collected at

RESPONSE: Thank you for pointing this out. The duplicated text has been moved to the "Crystal plates set-up" section (page 25-26, in red).

Please find below our responses to the referee comments and suggestions.

We thank them for their hard work and time to help us produce a better submission.

REVIEWER COMMENTS

Reviewer #1 (Remarks to the Author):

We thank the reviewer for the general comments.

Minor comments:

- Abstract: define "ABPP"

RESPONSE: The definition of ABPP (activity-based protein profiling) has been added to the abstract (page 1, in red)

- Page 6, line 11: explain "catalytic TEMPO"

RESPONSE: TEMPO stands for 2,2,6,6-Tetramethylpiperidine 1-oxyl. This information has been added to the manuscript (page 7, in red).

- Legend Figure 2: In the legend text, you could indicate that the ABPs 1-6 are the ABPs with the corresponding numbers shown in Figure 1. Although obvious, you could also indicate that the control SDS-gel with loaded enzymes is shown on the right. Incidentally, the short name shown here for each individual enzyme is not very visible and could be improved.

RESPONSE: We have clarified that the ABPs shown in Fig. 2 follow the same numbering as adopted in Fig. 1, and that the Coomassie blue–stained gel is shown on the right. In addition, the protein names were moved to the upper part of the Coomassie gel to improve visualisation.

- Figure 4: Have you tried analysing the fluorescently labelled protein bands shown in figure 4a by mass spectrometry to identify the specific proteins to which they correspond?

RESPONSE: Cy5-tagged compounds **1-6** were used for fluorescent detection, while the corresponding biotinylated compounds were employed for enrichment and protein identification. The mass spectrometry-based identification of the proteins labeled in Fig. 4a is shown in Fig. 4b–d and Supplementary Fig. 4. The corresponding results are discussed on page 14-15, in red).

- Methods/ Protein production and purification:
Please provide the codon optimization details. Which of the pET28a, pET21a, pET28a, and pET21a vectors were used for which enzyme?

RESPONSE: Codon optimization has now been clearly specified for each enzyme, together with the target organism and expression vector used (page 23-24, in red).

- Supplementary information, page 4: Move "General procedure A" to the next page. Supplementary information, page 5: Move "Compound S3" to the next page
... and so on

RESPONSE: All of these have been moved as suggested.

- Supplementary information, NMR spectra: It would be better to indicate the structures using the names given in the descriptions shown previously.

RESPONSE: The structures are now identified using the nomenclature previously established in the manuscript.

- Supplementary Figure 4. How much B. subtilis supernatant was added on the SDS-gel? In general, please provide this information also for all the other SDS-PAGE gel analyses, where secretomes were analysed.

RESPONSE: A total of 10 uL of supernatant (0.5–1.0 mg/mL protein) was loaded on the SDS-PAGE gel in experiments with secretomes. This information has been added to the Materials and Methods section ("Production of secretomes"- page 28, in red).

- Supplementary Table 10: Please give the genus names of the fungi.

RESPONSE: The genus names have been included.

- Throughout the main text and supplementary information: Check space before "°"

RESPONSE: All of these have been corrected in the revised manuscript.

<u>**Reviewer #2 (Remarks to the Author):**</u>

- It would have been helpful to have a version with line numbering.

RESPONSE: Line numbers have been added to the revised version of the manuscript.

- Define ABPP in the abstract.

RESPONSE: The definition of ABPP (activity-based protein profiling) has been added to the abstract (page 1, in red).

- Dynamic polysaccharide degradation. Not polysaccharide's.

RESPONSE: This has now been corrected (page 2, in red).

- Better define the xylanases you are targeting with these mechanistic probes. Endo-acting retaining xylanases at their -1, -2 subsites. Vs Exo or Inverting.

RESPONSE: This information is now introduced in page 4 (3rd paragraph, in red). The target subsites are highlighted in the legend of Fig.1 (page 5-6, in red).

- 'A virtually unlimited source of energy for onward conversion into green chemicals' Unnecessarily grandiose and unclear. Remove.

RESPONSE: This, indeed inappropriate, sentence was removed.

- '… via retaining double-displacement mechanism' Add period.

RESPONSE: The missing period has been added (page 2, 3rd paragraph, in red).

- From the text it wasn't clearly explained why the glycosyl enzyme intermediate with the probes are stable and why they are not hydrolysed in the second step.

RESPONSE: We have added more details on why the probes are stable and not susceptible to hydrolysis: "Cyclophellitol epoxides and aziridines are weak electrophiles that are protonated by the catalytic acid/base residue. This protonation stabilises the developing positive charge and facilitates nucleophilic attack by the catalytic carboxylate. The resulting covalent adduct is not a productive glycosyl-enzyme intermediate and blocks water-mediated turnover, rendering these probes highly selective, mechanism-based irreversible inhibitors" (page 3, in red).

- '…in which a glutamic acid residue' glutamate.

RESPONSE: Glutamic acid has been changed to glutamate (page 4, 2nd paragraph, in red).

- '… that selectively and covalently label AXXs and GXXs' change to '…that selectively and covalently label endo-retaining AXX and GXX specific xylanases'

RESPONSE: The sentence has been modified (page 4, 3rd paragraph, in red).

- Fig 1. Add a panel to show the reaction mechanism of probe inhibition/formation of 'stable' adduct glycosyl-enzyme intermediate and show why the reaction doesn't proceed after formation of glycosyl-enzyme intermediate.

RESPONSE: The new panel was added to Fig. 1 as Fig. 1b. The legend was modified accordingly.

- Fig1a. what is going on with the glu acid/base arrows?

RESPONSE: The arrows have been corrected.

- Fig1b. Put the chemical numbering in order on the polysaccharide. For

example 1 and 2 on top 3,4 , 5, 6 on the bottom. Currently it reads 2,6 then 1, 4, 3, 5. There is no order.

RESPONSE: Fig. 1b has been modified. Compounds **1, 3**, and **5** are displayed on the bottom, and **2, 4,** and **6** on the top.

- Remove the n and brackets from the polysaccharide structure, this implies repeating motifs. Use dotted lines at each end.

RESPONSE: Both "n" and brackets have been removed from the glucuronoarabinoxylan structure.

- Add the Me group to compound 6

RESPONSE: The CH3 group has been added to compound **6** – Fig. 1.

- Add compound S7 to fig1 as it is a complex shown in fig3.

RESPONSE: Compound **S7** has been added to Fig. 1. The legend was modified accordingly.

- Figure 1 title. Glutamate nucleophile, not glutamic acid.

RESPONSE: "Glutamic acid" has been changed to glutamate.

- Scheme 1. Show full chemical structure of the final product with the 6-Cy5 and the 4-Bio on the non-reducing ends of the probes with the simplified shape schematic beside it.

RESPONSE: Scheme 1 has been updated to include the **6-Cy5** and **4-Bio** compounds, each bearing tags at the non-reducing ends.

- Have you encountered any off-target labelling of your enzymes, for example labelling of the catalytic acid/base rather than the nucleophile or other residues? This would be interesting to note at least in the supplementary section for future reference.

RESPONSE: We thank the reviewer for this important point. Under the conditions used in this study, we did not observe evidence for covalent labelling of residues other than the catalytic nucleophile in either our mass spectrometry analyses or our structural data. This is consistent with the expected behaviour of cyclophellitol-derived mechanism-based probes for retaining glycosidases, theoretically and as observed in published studies. We have now added a clarifying sentence to the manuscript to make this explicit (Legend of Fig. 3, in red).

- From the Fig 3a mtz which was provided, the experimental electron density of the ABP-3Bio with XynA it doesn't look like there is a covalent bond with the catalytic nucleophile even though it is modelled as such. The electron density looks planar at the anomeric carbon and the xylose seems mis-modelled in a chair conformation. I am unconvinced this is a covalent bond from the data

provided. Show the difference and weighted difference omit map for this complex, omitting both the glutamate nucleophile and the probe from the refinement.

RESPONSE: We thank the reviewer for carefully examining the electron density in this region and for raising this important point.

We agree that the density around the anomeric carbon is not consistent with a single, fully occupied covalent adduct. In particular, the maps indicate partial planarity at C1 and do not support modelling the ligand solely as the reacted epoxide.

To address this directly, we calculated both standard omit maps and weighted difference omit maps after removing the ligand and the side chain of the catalytic nucleophile (Glu280), followed by re-refinement. Refinement with the ligand modelled exclusively in either the unreacted or covalently linked state did not satisfactorily account for the observed density. In the fully unreacted model, the anomeric carbon approaches Glu280 at an implausibly short distance, whereas the fully covalent model leaves residual features unexplained.

Taken together, these observations are most consistent with a dual-occupancy model comprising both intact epoxide and covalently reacted glycosyl–enzyme states. We have therefore re-refined the structure accordingly, with both the ligand and Glu280 modelled in alternative conformations with refined occupancies.

The re-refined model has been deposited in the PDB under accession code 9RHV. Omit-map coefficients are provided in the Supplementary Information, representative maps are shown in Supplementary Fig. 1, and the main text and Fig. 3b have been revised to reflect the mixed-state interpretation.

- While some of the probes may be stably maintained in a covalent manner in some of the xylanases' active sites, at least with ABP-3Bio and XynA, this might not be the case. Mass spec analysis also showed only partial covalent labelling of CtXyl5A. Thus, the xylanase pull down assays may be due to probe covalently linked to the enzymes or they may be related to simple enzyme affinity for the product complex of the non-covalently-linked probe after hydrolysis of the enzyme-glycosyl intermediate or some combination of covalent and non-covalent interaction. It is difficult to distinguish the options as the study is missing inactivation kinetics. To confirm probe mechanism, probe inactivation kinetics (for Cy5 and Bio) should be demonstrated with the control xylanases (CtXyl5A, CtXyn30A, XynA, TaXyn10) where an important probe interaction is seen.

RESPONSE: We agree that defining the mechanism and extent of covalent modification is important for interpreting the pull-down experiments. We also agree that inactivation kinetics would, in principle, be informative. However, we have not included such experiments in the present revision because the required substrate series is not currently available. Specifically, these measurements would require a panel of branched oligosaccharides bearing suitable leaving groups across the relevant substitution patterns, and to our knowledge such compounds have not been

described. Their preparation would require a substantial synthetic effort beyond the scope of the present revision. Indeed, one of the motivations for the present probe-development study was the lack of suitable substrates for selectively interrogating these enzymes.

To address the reviewer's concern as directly as possible, we instead performed intact-mass spectrometry on the biotinylated probes for *Ct*Xyl5A, XynA, *Ta*Xyn10 and *Ct*Xyn30A. Because intact MS directly reports the expected mass shift of the enzyme–probe conjugate, it provides direct evidence for covalent adduct formation in solution. These new data are now included in Supplementary Fig. 3, page 12 (3rd paragraph, in red), and page 13, in red.

GH5:
*Ct*Xyn5A showed partial and full conversion to CtXyn5A-**4-Bio** after 1 h or 16 h of incubation, respectively.

GH10:
XynA reached full conversion after 1 h incubation with **3-Bio** (used for XynA crystallography). In contrast, less than 10% of XynA was converted to XynA-**1Bio** even after 16 h of incubation, suggesting that the decorated backbone favoured the catalytic site compared to the substituted xylobiose moiety of **1-Bio**.

These findings are consistent with the *C. thermocellum* pull-downs presented in this work (Fig. 4b-d), where AXX/GXX are not enriched under **1-Bio** conditions. Further corroboration comes from competition assays, in which the reference enzyme used for our SSN analysis (Tr-Q9P973, known to accept decorated substrates) required higher **1-Bio** loadings than **3-Bio** to achieve inhibition (Fig. 5c).

The same response was observed for *Ta*Xyn10.

To address the, apparent, disconnect between **Bio-** and **Cy5-**tagged probes, we incubated XynA and *Ta*Xyn10 with **1-Cy5** and **3-Cy5**. Under these conditions, full conversion was observed after 1 h incubation, agreeing with in-gel data:

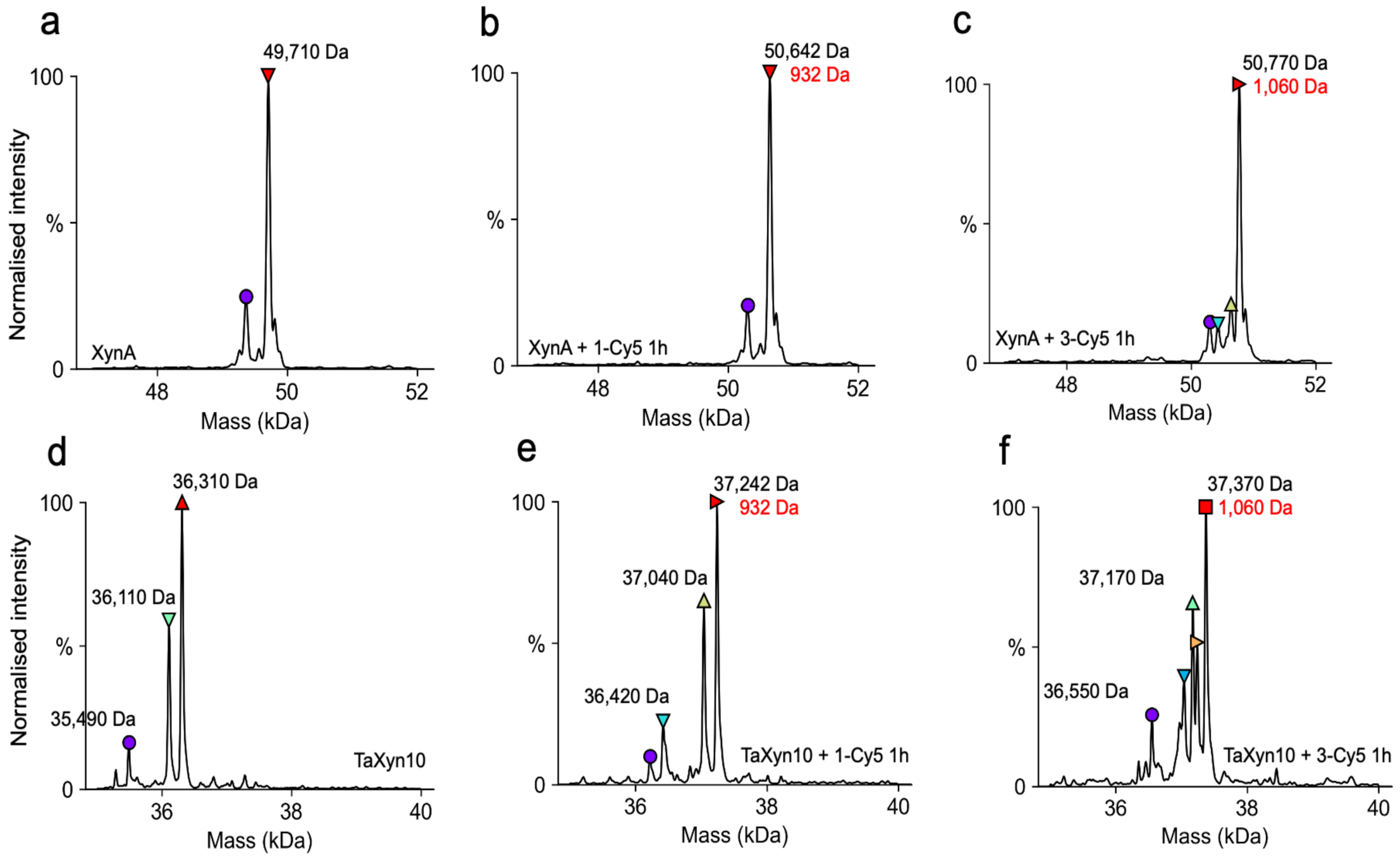

Figure 1. Intact mass spectrometry of XynA (a-c) and *Ta*Xyn10 (d-f). XynA (a) reveals full conversion to XynA-**1-Cy5** (b) and XynA-**3-Cy5** (c), with a mass shift consistent with formation of a single covalent adduct (**1-Cy5** predicted: +931 Da; observed: +932. **3-Cy5** predicted: +1,063 Da; observed: +1,060). The same procedure was adopted for *Ta*Xyn10 (a), *Ta*Xyn10-**1-Cy5** (b) and *Ta*Xyn10-**3-Cy5** (c).

These findings suggest that, in solution, contacts between the specificity motif and the enzyme are critical for accommodating the more rigid **biotin** linkers, whereas the longer and more flexible **Cy5** linker can promote accommodation of suboptimal configured sugars. This interpretation is consistent with the crystallographic data: XynA-**3-Bio** yielded defined complexes, whereas no ligand density was observed for **1-Bio**. In contrast, neither **Cy5**-based probe produced crystals in co-crystallisation assays or ligand density upon soaking, likely due to fluorophore disorder and lattice constraints.

GH30:

In the GH30 family, full conversion of *Ct*Xyn30A was observed with **S7** (GXX inhibitor used to obtain CtXyn30A complexes)

Taken together, these intact-mass data show that the biotinylated probes form covalent adducts in solution and are consistent with the pull-down results. These findings indicate that covalent modification makes a substantial contribution to pull-down enrichment, while not excluding a minor contribution from non-covalent interactions in some cases.

- The data for the pull down for CtXyn30A by 2-bio and 4-bio is contradictory to the Fig2b Cy5 labelling data. Can you offer an explanation for this? Is it possible the probe tail for Cy5 is interfering with the interaction?

RESPONSE: This apparent discrepancy likely reflects the different experimental contexts of the two readouts. The in-gel labelling experiments were performed with

purified *Ct*Xyn30A and therefore primarily report direct covalent labelling of the isolated enzyme. By contrast, the pull-down experiments were performed on *C. thermocellum* cellulosomal secretomes, in which enzymes are present within a tightly associated multi-enzyme complex linked by high-affinity cohesin-dockerin interactions.

Accordingly, enrichment in the pull-down experiment does not necessarily imply the same degree of direct labelling observed for the purified enzyme. We therefore consider it plausible that *Ct*Xyn30A was co-enriched under some probe conditions through association with other labelled cellulosomal xylanases. This interpretation is consistent with the stronger enrichment observed with **6-Bio**, which matches the purified-enzyme behaviour more closely, and the more moderate enrichment observed with **2-Bio** and **4-Bio**. We have now clarified this point in the manuscript (page 15, in red).

In summary, the purified-enzyme experiments indicate clear probe preferences at the level of covalent labelling, whereas the broader enrichment patterns observed in complex samples likely reflect the additional influence of sample context, including lower-efficiency interactions and possible co-enrichment.

- Fig4f title. By 6b do you mean 6-Bio?

RESPONSE: “6b” has been changed to 6-Bio in Fig. 4f title.

- ‘Notwithstanding the caveat that current ABP target only the negative subsites’ of endo-retaining xylanases.

RESPONSE: The sentence has been modified (page 20, in red).

- Conclusion: ‘…selective targeting’ the negative subsites of endo-retaining AXX and GXX xylanases.

RESPONSE: The sentence has been modified (page 22, in red).

<u>Reviewer #3 (Remarks to the Author):</u>

I believe this platform will be of great interest to the world of enzyme discovery, engineering for a number of biotechnological applications.

The manuscript is comprehensive and well-written - save for some minor points that I think should be addressed (see below), I have no hesitation in recommending that this paper be published.

Minor:
- Figure 5. I found the sequence similarity network a little hard to read when printed out. I would recommend increasing the size of it by 20%.

RESPONSE: Figure 5 was revised to improve readability.

- Methods:

The two sections: Crystal plates set-up and Crystal structure solution and refinement ready a bit duplicated. Please move everything for crystallisation into the top section. The Crystal structure solution section should start with : Diffraction data collected at

RESPONSE: Thank you for pointing this out. The duplicated text has been moved to the “Crystal plates set-up” section (page 25-26, in red).
